# Natural active ingredients targeted inflammatory cytokines and major blinding eye diseases: a two-sample Mendelian randomization and molecular docking analysis

**DOI:** 10.3389/fmed.2025.1427144

**Published:** 2025-07-04

**Authors:** Ying Shao, Yicheng Lu, Yue Gu, Yujia Chen, Chen Li

**Affiliations:** ^1^Department of Ophthalmology, Kunshan Rehabilitation Hospital, Suzhou, Jiangsu, China; ^2^Department of Ophthalmology, The First Affiliated Hospital of Soochow University, Suzhou, Jiangsu, China; ^3^School of Clinical Medicine, Nantong University, NanTong, Jiangsu, China; ^4^School of Clinical Medicine, Nanjing Medical University, NanJing, Jiangsu, China

**Keywords:** eye disease, GWAS, inflammatory cytokines, Mendelian randomization, molecular docking

## Abstract

**Objectives:**

Previous studies have reported that a few inflammatory cytokines have associations with ocular diseases. The objective of this study is to explore the causal relationship between 41 inflammatory cytokines and five ocular diseases using Mendelian randomization (MR) method and study the interaction between five natural active ingredients and inflammatory cytokines through molecular docking.

**Methods:**

The two-sample MR study employed genetic variances related to age-related macular degeneration (AMD), glaucoma, diabetic retinopathy (DR), myopia, and cataract. These variances were sourced from a comprehensive, publicly accessible genome-wide association study (GWAS). Additionally, inflammatory cytokines were derived from a GWAS summary that included 8,293 healthy individuals. The study primarily used the inverse variance weighted (IVW) method to investigate the causality between exposures and outcomes. To further bolster the final results, a variety of methods were concurrently used, including MR-Egger, weighted median, simple mode, weighted mode, and MR-PRESSO. A protein-protein interaction (PPI) network was constructed, and corresponding protein interaction relationships were analyzed utilizing the STRING database. Molecular docking served as an evaluation tool, confirming the binding between components and targets. This process was performed using AutoDock and PyMOL software.

**Results:**

The results indicated that IL-18 (OR: 1.134, 95% CI: 1.009–1.275, *P* = 0.034) and PDGF-BB (OR: 0.804, 95% CI: 0.678–0.954, *P* = 0.012) had protective effect on AMD; Genetically predicted RANTES had protective effect on glaucoma (OR: 0.886, 95% CI: 0.810–0.969, *P* = 0.008); IL-10 had protective effect on DR (OR: 0.871, 95% CI: 0.759–0.999, *P* = 0.048); GROa may be associated with increased myopia risk (OR: 1.230, 95% CI: 1.046–1.446, *P* = 0.012); Eotaxin (OR: 1.089, 95% CI: 1.018–1.165, *P* = 0.013), FGF2 (OR: 1.183, 95% CI: 1.004–1.393, *P* = 0.045) and GROa (OR: 1.053, 95% CI: 1.000–1.109, *P* = 0.049) were associated with increased cataract risk, while IL-1RA may be associated with decreased cataract risk. PPI network showed GROa, FGF2, IL-18, IL-1RA, IL-10, and Eotaxin interact closely. Molecular docking simulation showed that most of the compounds have good binding activities with critical targets.

**Conclusion:**

The present study identified inflammatory cytokines with causal relationships to five ocular diseases, revealing potential compounds for treating these diseases, providing a theoretical basis for further clinical practice.

## Introduction

Inflammatory response is extensively participating in the development and progression of many ocular diseases, and research on treating related ocular diseases by regulating inflammatory response has now attracted the attention of ophthalmologists worldwide. Recent studies have found inflammatory response also plays an important role in traditionally non-inflammatory ophthalmic diseases, such as age-related macular degeneration (AMD), glaucoma, diabetic retinopathy (DR), myopia, and cataract (1–3). AMD is a progressive retinal degenerative disease, and the important role of inflammatory response and oxidative stress in its pathogenesis and the inflammatory response process of neovascular AMD cannot be ignored. Retinal pigment epithelial cells, microglial cells and other intraocular immune surveillance cells gradually cause an imbalance between pro-oxidation and anti-oxidation signals in the series of reactions induced by external antigens and the process of self-aging, leading to excessive tissue oxidative stress, related inflammation processes, immune response disorders, and damage to the blood-retinal barrier, triggering the adaptive response of the immune system. At the same time, aging cells secrete a variety of pro-inflammatory cytokines and chemokines, which further stimulate microglial cells or macrophages and the tissue complement system (4). Interleukin-18 (IL-18), as a pro-inflammatory cytokine, plays an important role in the pathological process of AMD. Studies have shown that IL-18 is closely related to the activation of the NOD-like receptor thermal protein domain associated protein 3 inflammatome, leading to inflammatory response and oxidative stress in retinal pigment epithelium (RPE) cells (5). Certain compounds such as cis-urocanic acid can inhibit the release of IL-18. Thus, the inflammatory response and phototoxicity of RPE cells can be reduced, showing potential in the treatment of AMD (6). Glaucoma is an irreversible blinding ocular disease caused by secondary optic nerve structure and function damage involved in immune inflammation, and alleviating local immune inflammation can promote partial vision recovery. Glial cells first participate in the neurodegenerative process of glaucoma, and mechanically sensitive ion channels perceive stress, and adenosine receptor inflammasomes are activated. Peripapillary retinal glial cells resist the mechanical stress caused by increased intraocular pressure, rebuild the metabolic homeostasis of the retina and optic nerve, chronic mechanical stimulation, vascular stress stimulation, and complement cascade activation led to glial cell decompensation, eventually progressing to glaucoma optic neuropathy (7). Studies have found that the concentration of RANTES in aqueous humor of primary congenital glaucoma patients with primary congenital glaucoma is significantly different from that of primary open-angle glaucoma patients and healthy controls, suggesting that RANTES may play an important role in the pathogenesis of primary congenital glaucoma (8). DR is a foremost cause of irreversible vision impairment in the elderly, and its pathogenesis includes vascular changes causing intraocular immune inflammation activation stimulating neovascularization, macular edema formation, etc. Chronic low-grade inflammation processes have been widely detected in different stages of DR animal models and DR patients (9). IL-10 significantly alleviates the outer blood-retinal barrier damage and vascular leakage in early DR through regulation of macrophage polarization (from pro-inflammatory M1 to anti-inflammatory M2) (10). In patients with type 1 diabetes, peripheral blood mononuclear cells of patients with DR Exhibit higher levels of IL-10, suggesting that IL-10 may play an important role in the regulation of inflammation in DR (11). Myopia is a public health problem whose cause and mechanism are not fully understood. It was previously thought that myopia was mostly caused by factors such as form deprivation, peripheral defocus, genetic variation, etc., but recent research shows that immune inflammation is closely related to the progression of myopia. Significant changes in inflammatory factors have been detected in the intraocular fluid of simple myopia animal models, and cyclosporine can down-regulate inflammatory factors and inhibit the progression of myopia. In addition, there may be a connection between myopia and congenital subclinical inflammation of the conjunctiva, sclera, retina or choroid. The concentrations of IL-6 in aqueous humor were found to be positively correlated with the axial length in high myopia patients (12). Cataract is caused by lens protein denaturation leading to lens turbidity, and ocular inflammation and local immune response are vital risk factors for the occurrence of cataracts. The immune cells of the lens, which are produced during embryonic development, are activated during events such as ocular surgery, trauma, or local metabolic changes. These cells produce an adaptive immune response to lens denaturation and initiate repair. However, this immune response alters the transparent state of the lens (13). The interaction of IL-1RA with other inflammatory factors plays an important role in the pathogenesis of cataract. The decrease of IL-1RA may exacerbate the inflammatory response and thus promote the development of cataracts (14). Although current research is more focused on the regulation of post-cataract surgery inflammation, the existence of immune inflammation mechanisms undoubtedly becomes an important breakthrough in cataract prevention, delaying development, and regulating the lens regeneration process.

MR is an epidemiological method based on genetic variation. It used genetic variation as instrumental variables (IVs) to establish evidence of causality between exposures and outcomes (15). Because allelic variants are randomly assigned during meiosis, this approach is not subject to the bias of confusion and reverse causation (16). Two-sample MR analysis extracts genetic effects estimates from two sets of non-overlapping individuals, which will effectively enhance causal inference (17). In the present study, first we extracted summary statistics from published genome-wide association study (GWAS) of 41 inflammatory cytokines to analyze the causal relationship between these inflammatory cytokines with five ocular diseases. Then, by establishing the protein-protein interaction (PPI) network, the interaction between inflammatory cytokines was explored. Finally, the potential natural active ingredients for treating these ocular diseases were investigated by molecular docking analysis.

## Methods

This two-sample MR analysis was performed based on published GWAS summary data. Each institutional review committee approved the study, and all participants gave informed written consent. No additional informed consent and ethics approval were necessitated. This study was conducted in accordance with the guidelines for Strengthening the Reporting of Observational Studies in Epidemiology-Mendelian randomization (STROBE-MR) (18). Supplementary Table S1 contains the STROBE statement.

### MR assumptions

**Figure 1:**
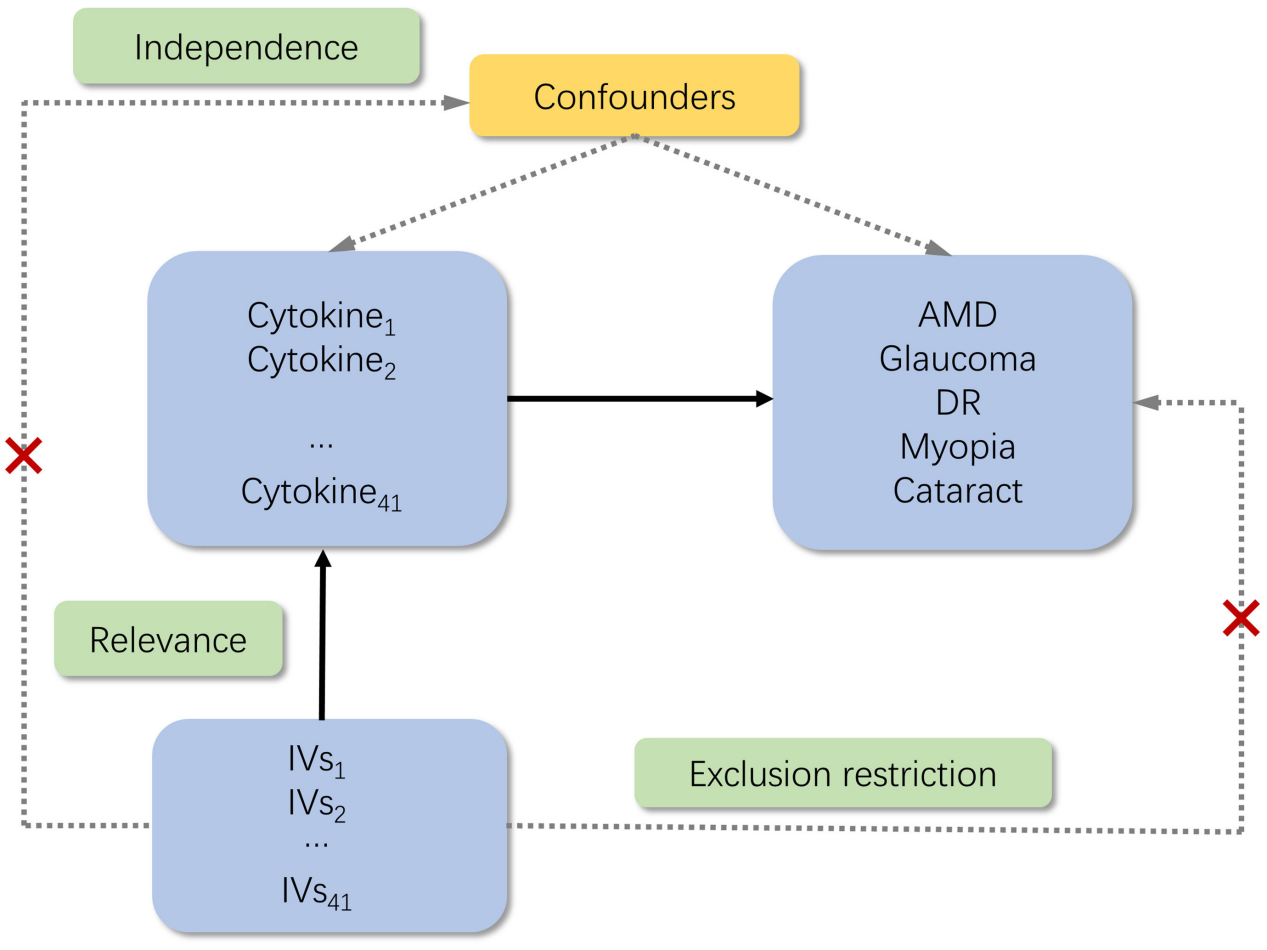
Schematic of the study design in this MR analysis. Significant instrumental variables (IVs) were selected for 41 inflammatory cytokines, and then the causal relationship between inflammatory cytokines and five ocular diseases was investigated. Three basic assumptions of MR analysis were illustrated in this causal directed acyclic graph, namely, relevance, independence, and exclusion restrictions. IVs, instrumental variables; AMD, age-related macular degeneration; DR, diabetic retinopathy.

For the genetic instrument to be valid, three key assumptions must be met: (1) relevance assumption: the genetic variants are associated with the exposure; (2) independence assumption: no unmeasured confounders of the associations between genetic variants and outcome; (3) exclusion restriction: the genetic variants influence the outcome only via the exposure (19). In the present study, six GWASs were employed to identify genetically significant Single Nucleotide Polymorphisms (SNPs) associated with 41 inflammatory cytokines and five ocular diseases (Figure 1).

### IVs selection

Initially, we established 5 × 10^−6^ as a genome-wide significance threshold to identify SNPs that are strongly linked with AMD, glaucoma, DR, myopia, cataracts, and inflammatory cytokines. Subsequently, to circumvent linkage disequilibrium, we grouped these SNPs (kb = 10,000, *r*^2^ = 0.001). We discarded SNPs with medium-frequency palindromic structures due to the uncertainty of their alignment in the same direction in GWASs of inflammatory cytokines. Lastly, we utilized the *R*^2^ value of each SNP to compute the variance proportion of exposure, in order to eliminate the effect of linkage disequilibrium. The *F*-statistic was employed to gauge the tool's strength, thus mitigating the risk of weak instruments bias (20, 21). We employed IVW method, MR-Egger (testing level pleipotency) and weighted median method (robust for invalid instrumental variables) to calculate β-value (22, 23).

### Data source

This MR analysis utilized six datasets, all of which were derived from the open-access GWAS dataset at https://gwas.mrcieu.ac.uk/. Inclusion criteria: all GWAS data were based on populations of European descent, and case and control definitions closely followed the original study. Control group excluded any history of eye disease. Exclusion criteria: malignancies, acute and chronic infections (e.g., HIV, tuberculosis), autoimmune diseases (e.g., rheumatoid arthritis), and other systemic diseases that may affect cytokine levels were excluded in the original study. The AMD dataset was procured from a study that included 3,763 cases and 205,359 controls of European ancestry. Similarly, the glaucoma dataset was sourced from a meta-analysis study that encompassed 8,591 cases and 210,201 controls of European ancestry. The DR dataset, also of European ancestry, was derived from a study comprising 3,646 cases and 203,018 controls. The myopia dataset was derived from a meta-analysis study that included 1,640 cases and 210,931 controls of European ancestry. The cataract dataset was sourced from a meta-analysis study that encompassed 26,758 cases and 189,604 controls of European ancestry. For inflammatory cytokines, the data was obtained from a study that provided genome variant associations with 41 inflammatory cytokines in 8,293 Finnish participants (24). The present study amalgamated results from two sources, which are the FINRISK surveys and the Cardiovascular Risk in Young Finns Study. Participants had a mean age of 60 years for the FINRISK survey and 37 years for the YFS study. There was no overlap in population selection between the exposure group and the outcome group. Table 1 displayed the 41 inflammatory cytokines which was investigated in this study.

**Table 1:** The 41 inflammatory cytokines upon which the effect of five ocular diseases was investigated.

**Inflammatory cytokines**
Beta-nerve growth factor (B-NGF)
Cutaneous T-cell-attracting chemokine (CTACK)
Eotaxin
Fibroblast growth factor 2 (FGF2)
Granulocyte-colony stimulating factor (G-CSF)
Growth regulated oncogene alpha (GROa)
Hepatocyte growth factor (HGF)
Interferon gamma (IFN-γ)
Interleukin 1 beta (IL-1B)
Interleukin 1 receptor alpha (IL-1RA)
Interleukin-2 (IL-2)
Interleukin-2 receptor alpha (IL-2RA)
Interleukin-4 (IL-4)
Interleukin-5 (IL-5)
Interleukin-6 (IL-6)
Interleukin-7 (IL-7)
Interleukin-8 (IL-8)
Interleukin-9 (IL-9)
Interleukin-10 (IL-10)
Interleukin-12-P70 (IL-12-P70)
Interleukin-13 (IL-13)
Interleukin-16 (IL-16)
Interleukin-17 (IL-17)
Interleukin-18 (IL-18)
Interferon gamma-induced protein (IP-10)
Macrophage colony-stimulating factor (M-CSF)
Monocyte chemoattractant protein-1/monocyte chemotactic and activating factor (MCP-1/MCAF)
Monocyte chemotactic protein-3 (MCP-3)
Macrophage migration inhibitory factor (MIF)
Mitogen-inducible-gene (MIG)
Macrophage inflammatory protein-1 alpha (MIP-1A)
Macrophage inflammatory protein-1 beta (MIP-1B)
Platelet-derived growth factor (PDGF-BB)
Chemokine ligand 5 (RANTES)
Stem cell factor (SCF)
Stem cell growth factor-beta (SCGF-B)
Stromal cell-derived factor-1 alpha (SDF-1A)
Tumor necrosis factor-alpha (TNF-A)
Tumor necrosis factor-beta (TNF-B)
Tumor necrosis factor-related apoptosis-inducing ligand (TRAIL)
Vascular endothelial growth factor (VEGF)

### Statistical analysis

The causal effect estimation in the present study primarily employs the Inverse Variance Weighted (IVW) method. The IVW method is an ideal state estimation with a strong ability to detect causal relationships. However, the major drawback is that it assumes all IVs are valid, and this assumption cannot be guaranteed in practice (25). Therefore, robust test methods that do not require all IVs to be valid are also used to provide assessments of causal relationships. Such as MR-Egger (26) and weighted median (27), which supplement the IVW, can more thoroughly estimate the causality between exposures and outcomes. In addition, the MR-PRESSO method could detect and correct potentially pleiotropic outliers (28). To ensure a strong association with exposures, we choose SNPs with an *F*-statistic >10 as IVs. We also determined potential heterogeneity in the results by calculating the *p*-value from the Cochran Q test derived from IVW. The MR-Egger regression method was used to test for horizontal pleiotropy. Furthermore, to assess the influence of individual SNPs, a leave-one-out method was conducted. The packages of R used in this study included “TwoSample,” “data.table,” “tidyverse,” “readxl,” “writexl,” “magrittr.”

*F*-statistic and IVW is calculated as follows:


$$\begin{array}{l} F=\frac{\beta^{2}}{SE^{2}} \end{array}$$

β: effect value of SNP on the exposure factor, SE: standard error.


$$\begin{array}{l} \beta_{IVW}=\frac{\sum \left(\beta_{X_{i}}\cdot \beta_{Y_{i}}/\sigma_{Y_{i}}^{2}\right)}{\sum \left(\beta_{X_{i}}^{2}/\sigma_{X_{i}}^{2}\right)}. \end{array}$$

### PPI data

We further analyzed the protein interactions among eight inflammatory cytokines that were selected by MR analysis to have significant causal relationship with five ocular diseases. We searched the database STRING (29) to exhibite a PPI network after importing the eight inflammatory cytokines (IL-18, PDGF-BB, RANTES, IL-10, GROa, Eotaxin, FGF2, and IL-1RA). Cytoscape 3.8.1 was then utilized to draw the interaction network and analyzed.

### Molecular docking

Molecular docking was carried out to further identify the potential interaction between the above eight inflammatory cytokines and natural active ingredients with anti-inflammatory and eye-protective effects. We downloaded the protein crystal structure by searching the Research of Cooperative Organization for structural bioinformatics protein database (http://www.pdb.org/). However, after searching, only the protein structures of IL-18 and RANTES were found and downloaded in PDB website for subsequent analysis. Structural files of the key components of these natural active compounds are then downloaded through TCMSP, and with Chem3D software, their energy is minimized. We use PyMOL software to find potential combination pockets and remove original ligands from protein molecules. The AutoDock tools were used to remove water molecules, add polar hydrogen atoms, merge nonpolar hydrogen atoms, define rotatable bonds. Binding energy of the docking results < -5 indicates a good binding interaction between the compound and target. All docking results were evaluated by the root-mean-square deviation (RMSD) value, and an RMSD value of < 2 was deemed reliable. Finally, visualization of molecular structures was carried out using the Discovery studio tool.

## Results

### Influence of 41 inflammatory cytokines on five ocular diseases

**Figure 2:**
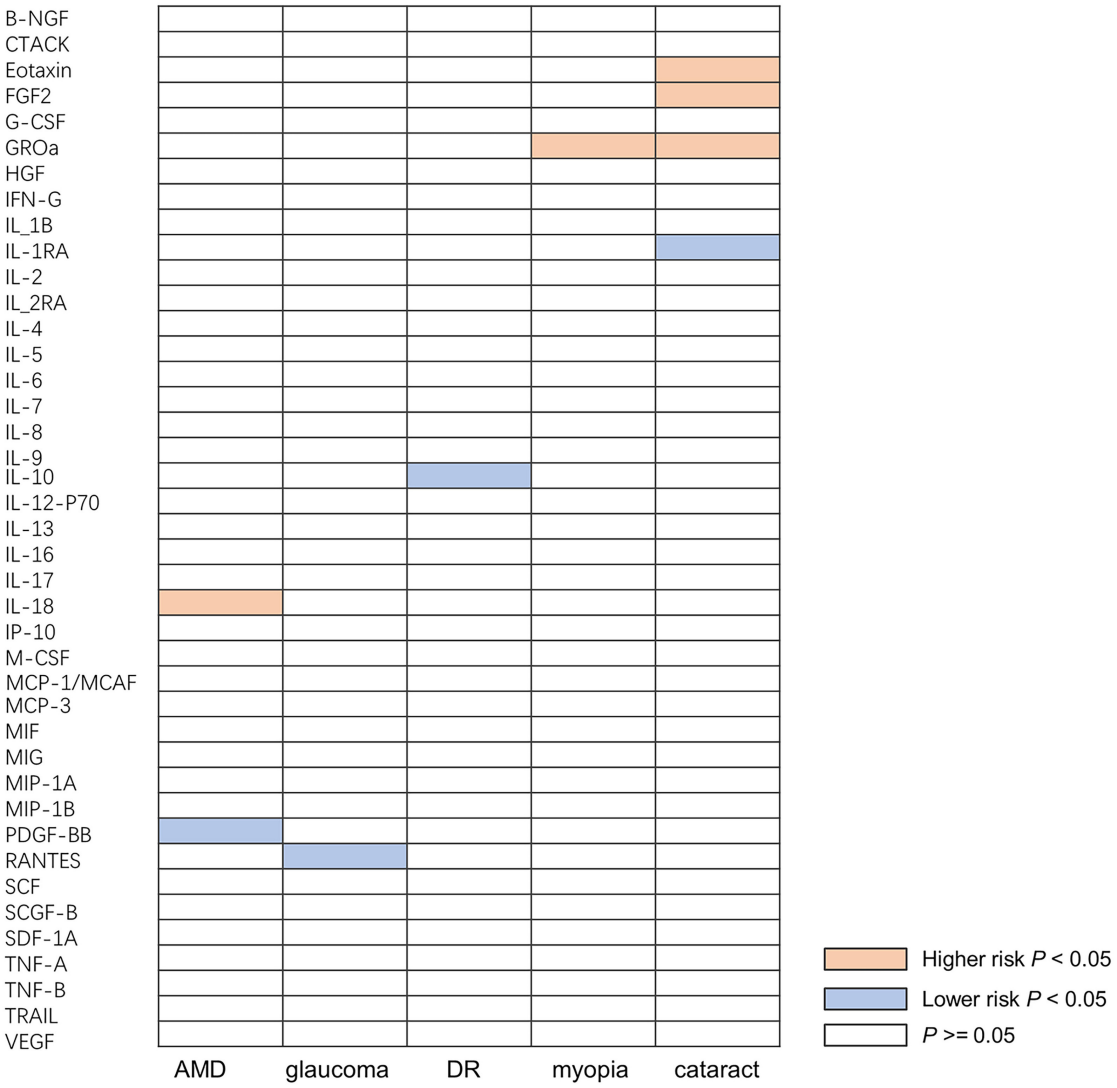
Causal correlations of inflammatory cytokines on five ocular diseases. The orange boxes represent the inflammatory cytokine may increase the risk of ocular disease. The blue boxes represent the inflammatory cytokine may reduce the risk of ocular disease. The white boxes represent that the difference is not significant. B-NGF, beta-nerve growth factor; CTACK, cutaneous T-cell-attracting chemokine; FGF, fibroblast growth factor; CSF, granulocyte-colony stimulating factor; GROa, growth regulated oncogene alpha; HGF, hepatocyte growth factor; IFN-γ, interferon gamma; IL, interleukin; IL-1B, interleukin 1 beta; IL-1RA, interleukin 1 receptor alpha; IL-2RA, interleukin 2 receptor alpha; IP-10, interferon gamma-induced protein; M-CSF, macrophage colony-stimulating factor; MCP-1, monocyte chemoattractant protein-1; MCAF, monocyte chemotactic and activating factor; MCP-3, monocyte chemotactic protein-3; MIF, macrophage migration inhibitory factor; MIG, mitogen-inducible-gene; MIP-1A, macrophage inflammatory protein-1 alpha; MIP-1B, macrophage inflammatory protein-1 beta; PDGF-BB, platelet-derived growth factor; RANTES, chemokine ligand 5; SCF, stem cell factor; SCGF-B, stem cell growth factor-beta; SDF-1A, stromal cell-derived factor-1 alpha; TNF-A, tumor necrosis factor-alpha; TNF-B, tumor necrosis factor-beta; TRAIL, tumor necrosis factor-related apoptosis-inducing ligand; VEGF, vascular endothelial growth factor; AMD, age-related macular degeneration; DR, diabetic retinopathy.

When the threshold of genome-wide significance was set at 5 × 10^−6^, all of 41 inflammatory cytokines had 3 or more valid genetic variants, and the F statistic for each inflammatory cytokine was >10, indicating that the weak tool bias was not significant (Supplementary Tables S2–S16). After selected the instruments, we used them to analyze the causal inference of 41 inflammatory cytokines and five ocular diseases, which include AMD, glaucoma, DR, myopia, and cataract. The IVW method was selected as the main analysis method, and no indications of heterogeneity, weak IVs, or horizontal pleiotropy were found in any of the MR analyses conducted in this study. A summary of the IVW test results is presented in Figure 2.

**Figure 3:**
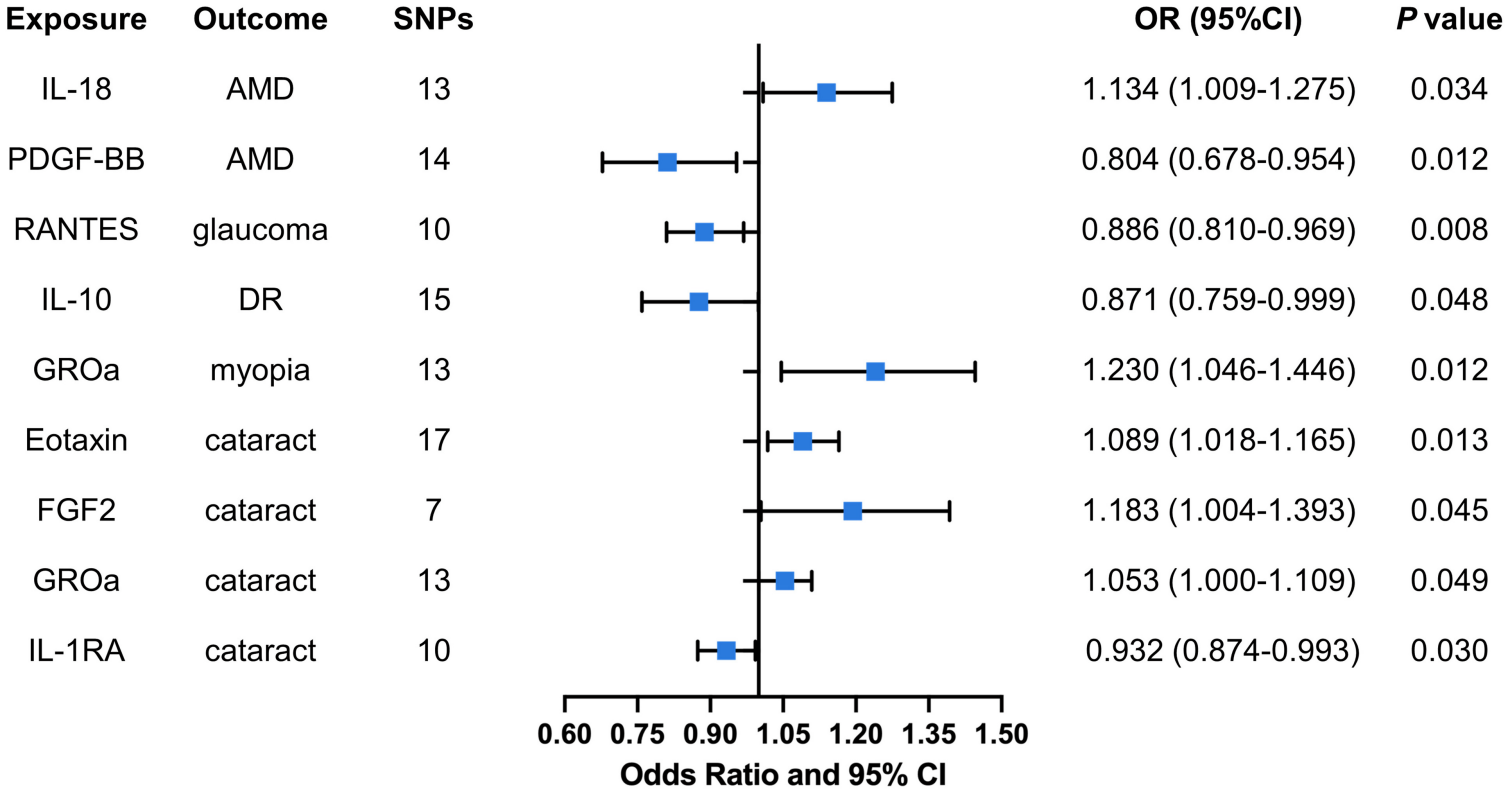
Causal correlations of inflammatory cytokines on five ocular diseases. The change in the OR of ocular diseases per 1-SD rise in the cytokine level is shown by OR and 95% CI. The results from IVW method were shown for all cytokines. IL, interleukin; PDGF, platelet-derived growth factor; RANTES, regulated upon activation normal T cell expressed and secreted factor; GROa, growth regulated oncogene alpha; FGF, fibroblast growth factor; AMD, age-related macular degeneration; DR, diabetic retinopathy; OR, odds ratio; CI, confidence internal.

A total of nine significant associations, including eight inflammatory cytokines were identified. For every 1-SD increase in interleukin 18 (IL-18) levels, the risk of developing AMD increased correspondingly [odds ratio (OR): 1.134, 95% confidence internal (CI): 1.009–1.275, *P* = 0.034], while inverse associations between genetically platelet-derived growth factor (PDGF-BB) concentrations and AMD (OR: 0.804, 95% CI: 0.678–0.954, *P* = 0.012) was found by using IVW method. Using the IVW method, an inverse association between genetically proxied circulating concentrations of chemokine ligand 5 (RANTES) and glaucoma risk (OR: 0.886, 95% CI: 0.810–0.969, *P* = 0.008) was found. The same causal relationship was observed in IL-10 with DR (OR: 0.871, 95% CI: 0.759–0.999, *P* = 0.048). Positive associations were observed for genetically proxied growth regulated oncogene alpha (GROa; OR: 1.230, 95% CI: 1.046–1.446, *P* = 0.012) in relation to myopia. For cataract, positive associations were observed for genetically proxied Eotaxin (OR: 1.089, 95% CI: 1.018–1.165, *P* = 0.013), fibroblast growth factor 2 (FGF2; OR: 1.183, 95% CI: 1.004–1.393, *P* = 0.045), and GROa (OR: 1.053, 95% CI: 1.000–1.109, *P* = 0.049) in relation to cataract. Furthermore, an inverse association for genetically proxied concentrations of Interleukin 1 receptor alpha (IL-1RA) in relation to cataract (OR: 0.932, 95% CI: 0.874–0.993, *P* = 0.030) was found. Figure 3 illustrates all relationships with a significance level of *P* < 0.05 in IVW models.

**Figure 4:**
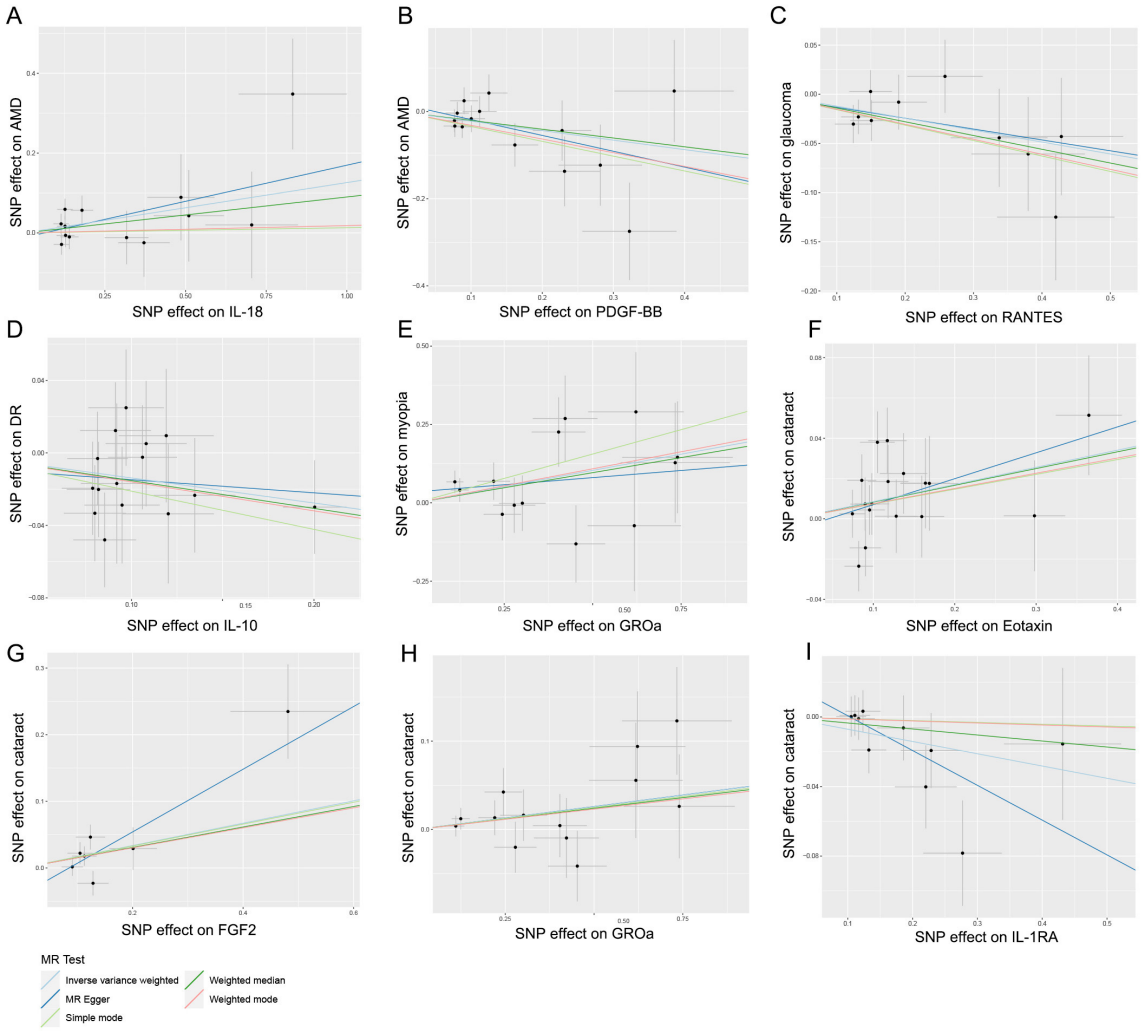
Scatter plots for the causal association between inflammatory cytokines and five ocular diseases. **(A)** Individual inverse variance (IV) associations with IL-18 risk are displayed vs. individual IV associations with AMD in black dots. **(B)** Individual IV associations with PDGF-BB risk are displayed vs. individual IV associations with AMD in black dots. **(C)** Individual IV associations with RANTES risk are displayed vs. individual IV associations with glaucoma in black dots. **(D)** Individual IV associations with IL-10 risk are displayed vs. individual IV associations with DR in black dots. **(E)** Individual IV associations with GROa risk are displayed vs. individual IV associations with myopia in black dots. **(F)** Individual IV associations with Eotaxin risk are displayed vs. individual IV associations with cataract in black dots. **(G)** Individual IV associations with FGF2 risk are displayed vs. individual IV associations with cataract in black dots. **(H)** Individual IV associations with GROa risk are displayed vs. individual IV associations with cataract in black dots. **(I)** Individual IV associations with IL-1RA risk are displayed vs. individual IV associations with cataract in black dots. The 95% CI of odd ratio for each IV is shown by vertical and horizontal lines. The slope of the lines represents the estimated causal effect of the MR methods. IL, interleukin; PDGF, platelet-derived growth factor; RANTES, regulated upon activation normal T cell expressed and secreted factor; GROa, growth regulated oncogene alpha; FGF, fibroblast growth factor; AMD, age-related macular degeneration; DR, diabetic retinopathy.

**Figure 5:**
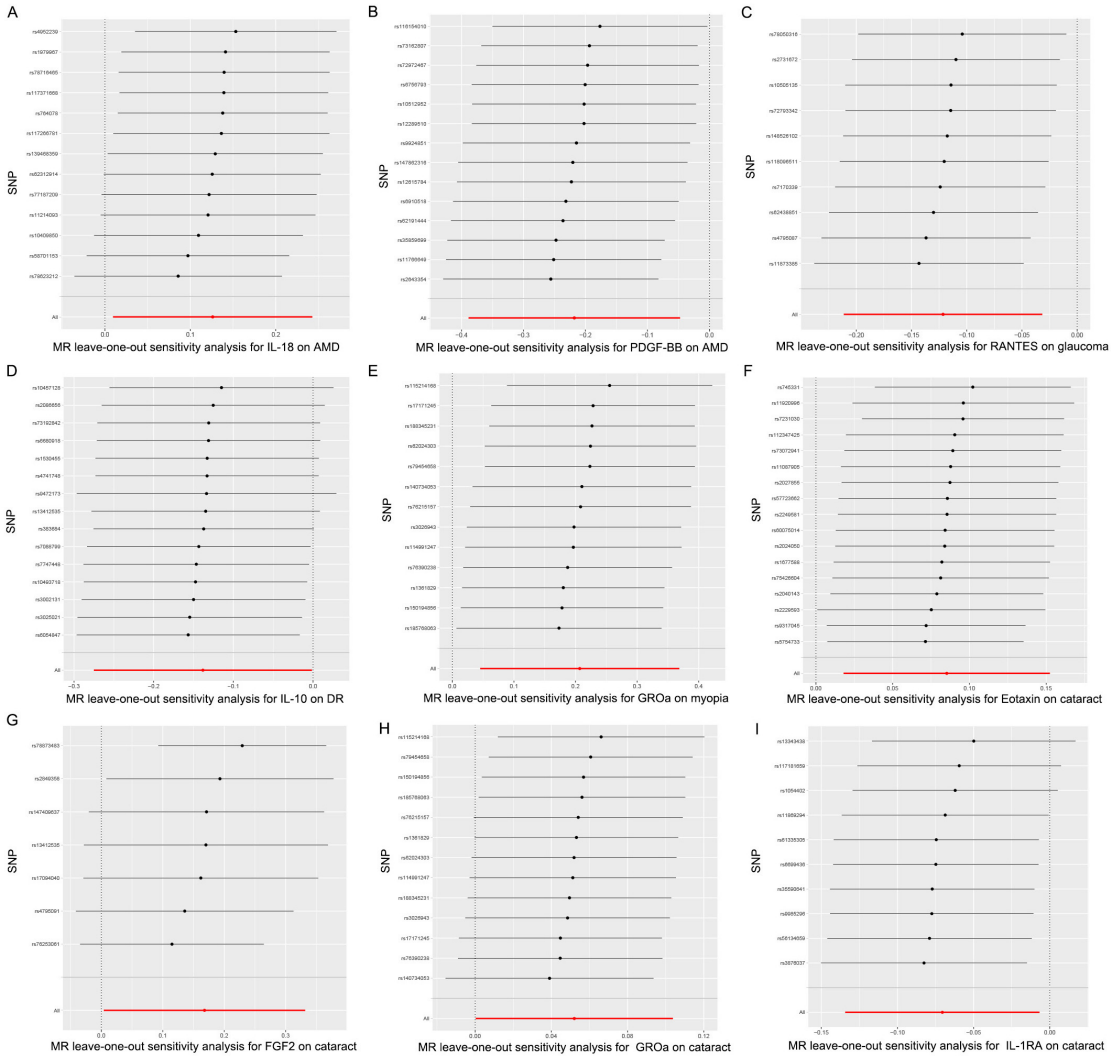
Leave-one-out plots for the causal association between inflammatory cytokines and five ocular diseases. **(A)** leave-one-out plots for the causal association between IL-18 and AMD; **(B)** leave-one-out plots for the causal association between PDGF-BB and AMD; **(C)** leave-one-out plots for the causal association between RANTES and glaucoma; **(D)** leave-one-out plots for the causal association between IL-10 and DR; **(E)** leave-one-out plots for the causal association between GROa and myopia; **(F)** leave-one-out plots for the causal association between Eotaxin and cataract; **(G)** leave-one-out plots for the causal association between FGF2 and cataract; **(H)** leave-one-out plots for the causal association between GROa and cataract; **(I)** leave-one-out plots for the causal association between IL-1RA and cataract. IL, interleukin; PDGF, platelet-derived growth factor; RANTES, regulated upon activation normal T cell expressed and secreted factor; GROa, growth regulated oncogene alpha; FGF, fibroblast growth factor; AMD, age-related macular degeneration; DR, diabetic retinopathy.

Hence, IL-18, PDGF-BB, RANTES, IL-10, GROa, Eotaxin, FGF2, and IL-1RA may be considered as therapeutic targets for these ocular diseases. The scatter plots of MR analyses for these inflammatory cytokines in five ocular diseases are exhibited in Figure 4. The leave-one-out method was implemented to ascertain the influence of each SNP on the overall causal estimate. Following the removal of each SNP, the MR analysis was meticulously carried out anew on the remaining SNPs (Figure 5). The findings remained consistent, highlighting a significant causal connection among the computed results of all SNPs.

### Analysis of PPI network

**Figure 6:**
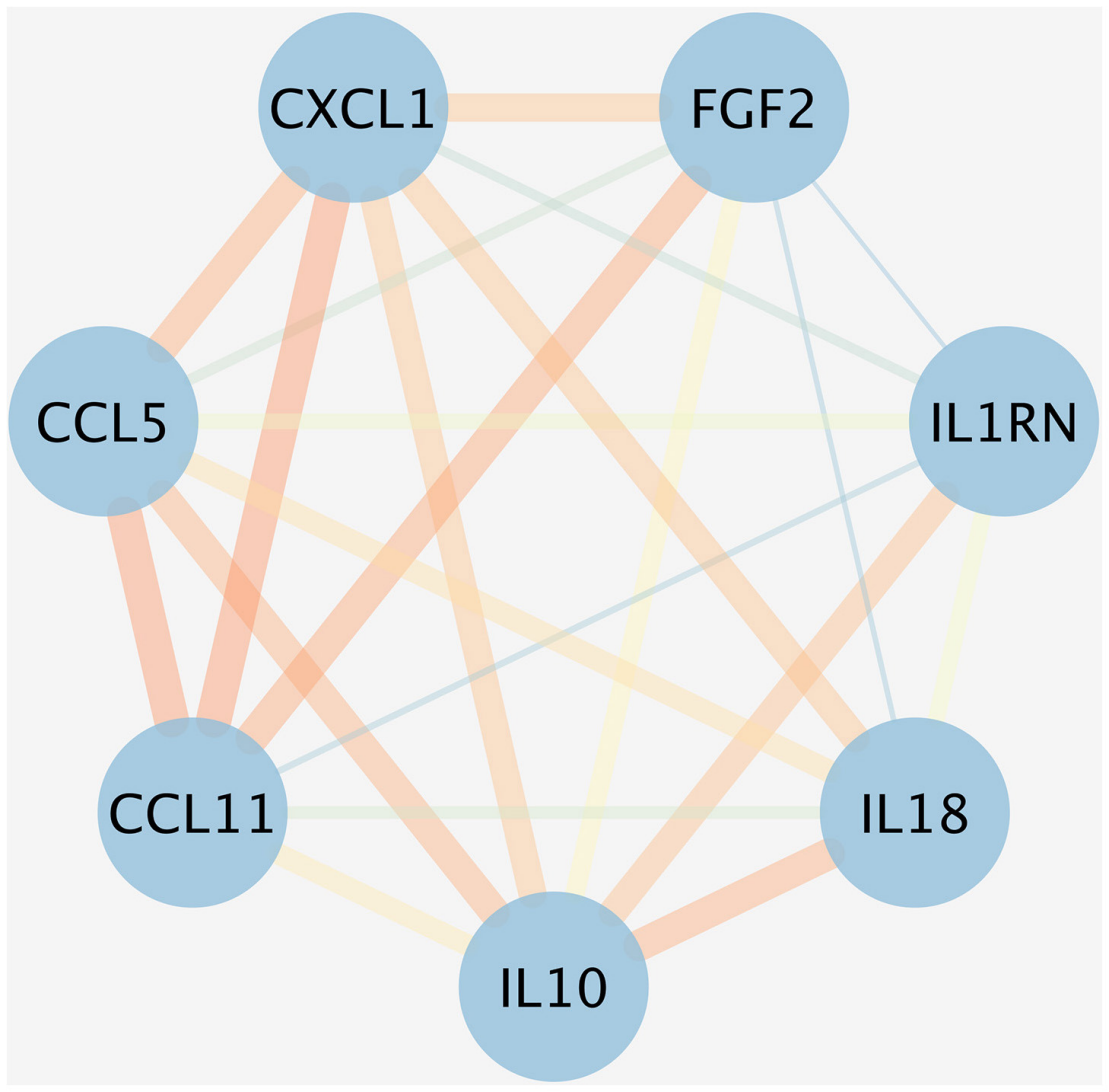
PPI network for the inflammatory cytokines associated with five ocular diseases. The nodes represented the proteins, and the different colored edges represented the interaction relationships of different proteins. The thicker the edge, the greater the combination score value. CXCL1, CXC motif chemokine ligand 1; FGF, fibroblast growth factor; IL, interleukin; IL-1RN, IL-1 receptor antagonist; CCL11, chemokine eotaxin-1.

An initial PPI network was constructed using the STRING database, centered around eight inflammatory cytokines. As depicted in Figure 6, the network comprised eight nodes and 21 edges, with an average node degree of 5.25 and a local clustering coefficient of 0.875. The thickness of each edge represented the combination score, with a thicker edge indicating a higher combination score value. In this network, the nodes symbolized proteins, while the edges denoted the interaction relationships among these proteins. Figure 6 showed that GROa [C-X-C motif chemokine ligand 1 (CXCL1)], FGF2, IL-18, IL-1RA (IL-1RN), IL-10, and Eotaxin (CCL11) are the six inflammatory cytokine that interact most closely.

### Molecular docking analysis

**Figure 7:**
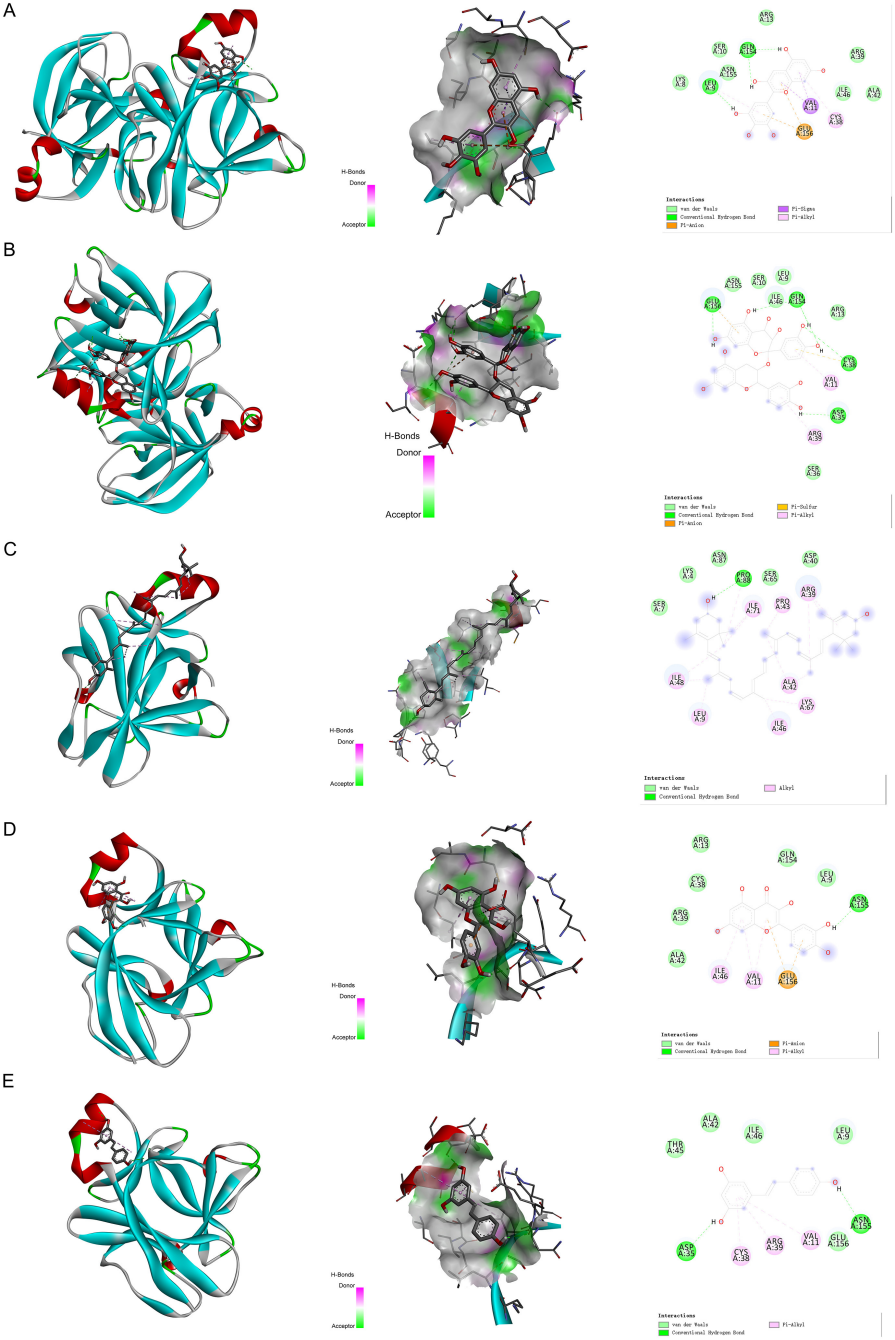
Molecular docking. Binding mode of **(A)** IL-18 with anthocyanin; **(B)** IL-18 with procyanidin; **(C)** IL-18 with lutein; **(D)** IL-18 with quercetin; and **(E)** IL-18 with resveratrol. IL, interleukin.

**Figure 8:**
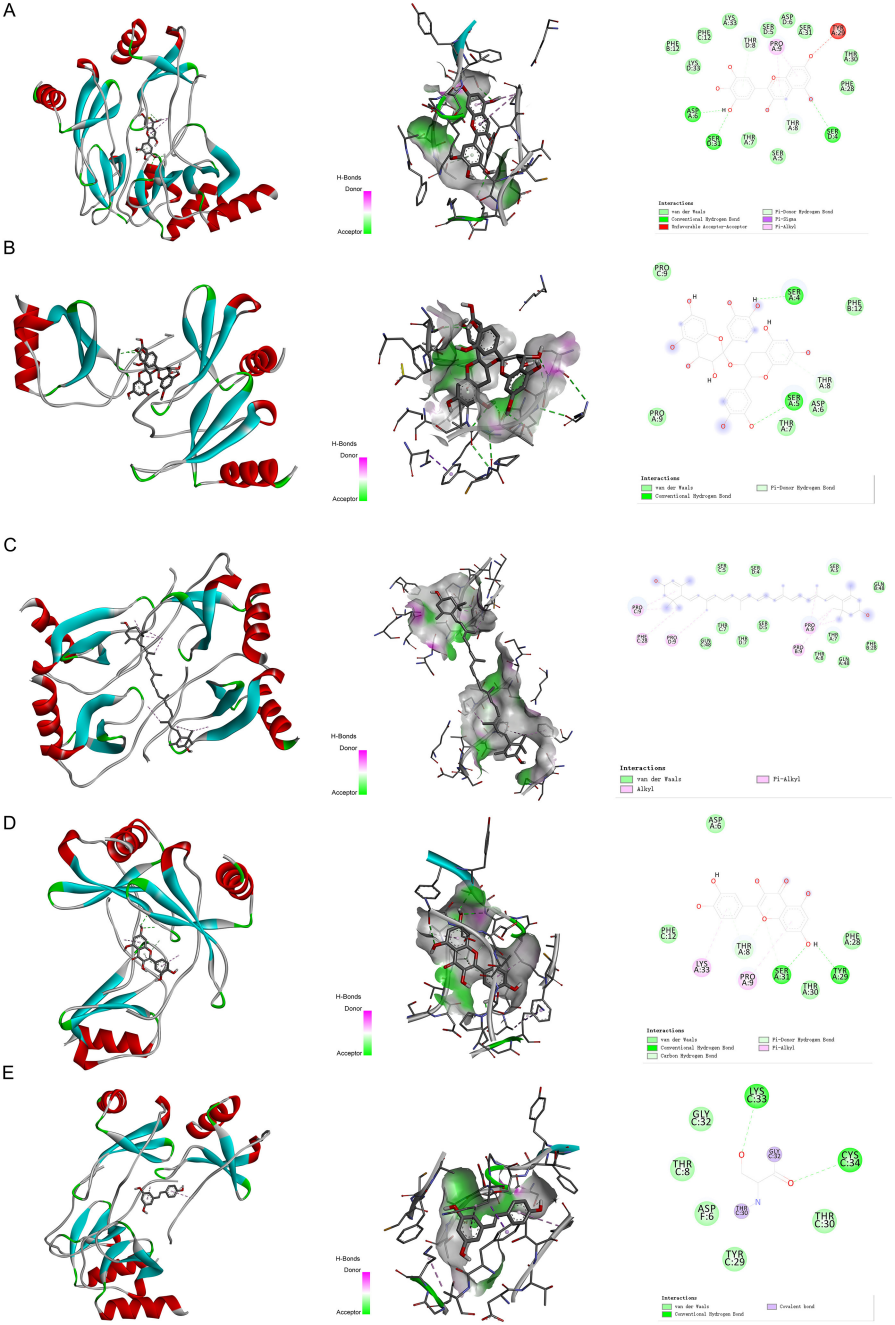
Molecular docking. Binding mode of **(A)** RANTES with anthocyanin; **(B)** RANTES with procyanidin; **(C)** RANTES with lutein; **(D)** RANTES with quercetin; and **(E)** RANTES with resveratrol. IL, interleukin.

We have selected the following natural active ingredients with anti-inflammatory and eye-protective effects: anthocyanins, procyanidin, lutein, quercetin, and resveratrol (Table 2) to perform molecular docking with IL-18 and RANTES to screen potential therapeutic drugs. The docking results are shown in Table 3. The binding energy between the ligand and the receptor serves as a crucial measure of their binding capacity, with a standard of ≤ -5.0 kcal/mol (30). From the docking results, the binding free energy of the active ingredients docked with the target proteins are all < -5.0 kcal/mol (Table 3), indicating that the active ingredients bind well with these target proteins. Figures 7, 8 showed the binding modes of the key active components docked with the target. Hydrogen bonds, van der Waals forces, and pi-alkyl are the primary forms of interaction. The compound with the lowest binding energy is anthocyanin, suggesting that this compound may have a good therapeutic effect on glaucoma.

**Table 2:** Basic information of key components.

**Component**	**Chemical formula**	**Molecular weight (g/mol)**	**Structure**
Anthocyanin	C_78_H_89_O_44_+	1,730.5	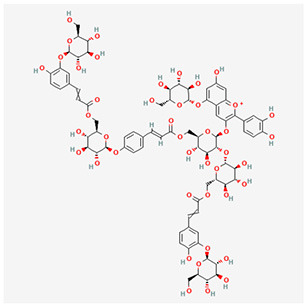
Procyanidin	C_30_H_26_O_13_	594.5	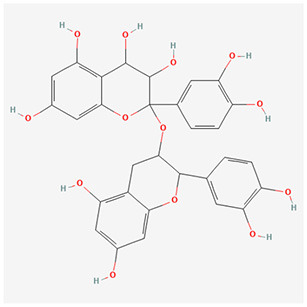
Lutein	C_40_H_56_O_2_	568.9	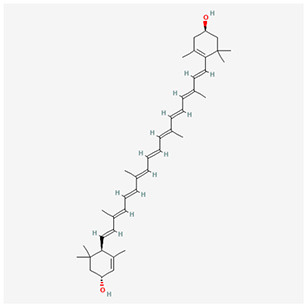
Quercetin	C_15_H_10_O_7_	302.23	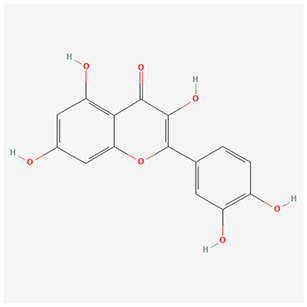
Resveratrol	C_14_H_12_O_3_	228.24	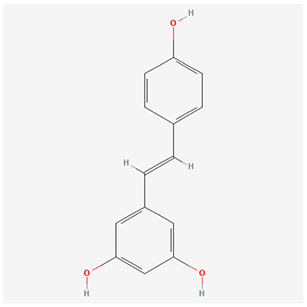

**Table 3:** Free binding energy of the key active components and key targets.

**Component**	**Target**	**Free binding energy (kcal/mol)**	**RMSD**
Anthocyanin	IL-18	−6.1	1.364
	RANTES	−8.2	0.122
Procyanidin	IL-18	−6.1	1.339
	RANTES	−7.2	1.970
Lutein	IL-18	−6.4	1.317
	RANTES	−5.4	1.410
Quercetin	IL-18	−5.5	1.775
	RANTES	−7.9	1.803
Resveratrol	IL-18	−5.5	1.332
	RANTES	−7.4	1.463

## Discussion

In this two-sample MR analysis, we first studied the causal relationship between 41 inflammatory factors as exposures and five ocular diseases as outcomes. The results suggest that IL-18 and PDGF-BB may be upstream causes of AMD; Genetically predicted RANTES had protective effect on glaucoma; IL-10 had protective effect on DR. GROa may be associated with increased myopia risk; FGF2, GROa, and IL-1RA may be upstream causes of cataract. The PPI results show that these six inflammatory factors, GROa, FGF2, IL-18, IL-1RA, IL-10, and Eotaxin, interact closely with each other. By further selecting five natural active ingredients with anti-inflammatory and eye-protective effects (anthocyanin, procyanidin, lutein, quercetin, resveratrol) for molecular docking with IL-18 and RANTES, the results showed that anthocyanin may have a good therapeutic effect on glaucoma. The comprehensive analysis method in this study provides a new perspective for understanding the potential mechanisms of five ocular diseases and identifying potential drug targets.

So far, many studies have explored the correlation between ocular diseases and inflammatory cytokines, such as classic immune-inflammatory ocular diseases including allergic conjunctivitis, immune corneal lesions, scleritis, non-infectious uveitis, optic neuritis, and thyroid associated ophthalmopathy (31–35). Recent studies have found that immune inflammation is also involved in the occurrence and development of some previously considered non-inflammatory ocular diseases, such as AMD, glaucoma, DR, myopia, and cataract (1–3). However, even with a large number of observational study results, the accidental correlation between these ocular diseases and important inflammatory cytokines cannot be explained as causal relationships between exposures and outcomes in observational data, and the possibility of reverse causality and residual confounding cannot be ruled out (36). Therefore, the changes in the levels of inflammatory cytokines in these patients may represent the pathogenesis of the disease, side effects of treatment, potential infection status, which may indistinguishable in observational study.

AMD is a progressive sight-threatening disease that results in the loss of photoreceptors in the macula. Clinically, AMD is classified into dry (non-neovascular) and wet (neovascular) AMD. Inflammatory responses play a significant role in both types of AMD pathological changes. Some researchers believe that IL-18 has a therapeutic effect on wet AMD (37). However, recent studies have overturned this conclusion. Experimental data from five different laboratories indicate that IL-18 has no protective effect in choroidal neovascularization-induced wet AMD models, and intraocular injection of IL-18 can even cause retinal toxicity (38). Furthermore, Yerramothu (2) found that Alu RNA activates NLRP3 inflammasomes to produce IL-18, leading to the apoptosis of human and mouse RPE cells. Our study concludes through MR analysis that IL-18 may be a risk factor for the occurrence and progression of AMD. Further research is needed to clarify the interaction between IL-18 and AMD. PDGF-BB has been reported to be involved in many pathological processes. In wet AMD, VEGF promotes the growth of neovascularization while PDGF-BB maintains the interaction between pericytes and endothelial cells in mature vessels (39). Although VEGF antagonists can prevent the angiogenesis of wet AMD, studies in other systems such as skeletal muscle splitting vessels have found that PDGF-BB limits the extent of vascular expansion by regulating VEGFR2 signaling and endothelial cell proliferation to prevent VEGF-induced abnormal angiogenesis, thus enabling the vessels to effectively split into a normal capillary network, with PDGF-BB seemingly playing a protective role (40). Our research using the MR method indicates that PDGF-BB has a protective effect on AMD, which may not be consistent with some existing research results on the effect of PDGF-BB on wet AMD. This discrepancy may be attributed to the inclusion of both wet and dry AMD in this study. The role of PDGF-BB in different types of AMD needs further research. Many studies have highlighted the elevated levels of various inflammatory cytokines in the aqueous humor and tear of POAG, in comparison to healthy individuals. RANTES, also known as CCL5, is a chemokine that is produced by T lymphocytes, endothelial cells, and platelets. It functions as a chemotactic factor, primarily facilitating T lymphocytes and activation basophil degranulation (41). An upsurge in RANTES expression has been linked to a broad spectrum of inflammatory pathologies. In the context of ophthalmology, several studies have correlated increased intraocular concentrations of RANTES with chronic inflammation conditions such as severe non-proliferative DR, pan-retinal photocoagulation, and uveal melanoma (42–44). However, there is limited research on the association between RANTES and glaucoma. In this study, we used MR method to explore the potential protective role of RANTES in glaucoma. These findings highlight the potential of early intervention targeting RANTES in preventing glaucoma outcomes. An important pathophysiological process in DR is the inflammatory response (45). IL-10 inhibits the infiltration of various inflammatory cells in the eye micro-vessels and the release of inflammatory mediators by these inflammatory cells, thereby reducing local inflammatory responses (46). Studies have shown that the lower the level of IL-10 in the human body, the higher the probability of DR occurrence and the more severe the condition (47). In this study, we used MR method to provide compelling evidence supporting the connection between DR and IL-10, suggesting that IL-10 may play a potential role in inhibiting the occurrence of DR. GROa, also known as CXCL1, is a member of the CXC chemokine family. The relationship between GROa and myopia is not clear, and research on this topic is limited. Our study found that GROa may be an upstream cause of myopia, but further research is needed to clarify the interaction between them. The MR analysis results for cataract showed that FGF2, Eotaxin, GROa, and IL-1RA may play a role in the occurrence and progression of the disease. It is known that FGF2 protein has different roles in various tissues of the eye, and the concentration gradient of FGF2 protein is considered to be the reason for the establishment of cell migration patterns in the lens (48). FGF2 is considered to be a growth factor that induces the differentiation of mammalian lens epithelial cells in lens. Besides, the differentiation of lens epithelial cells to fibroblasts requires FGF2 (49, 50). However, there is limited research on the relationship between Eotaxin, GROa, IL-1RA and cataract. We used MR method to explore the potential protective role of FGF2, Eotaxin, and GROa in cataract. IL-1RA may increase the risk of cataract. How these cytokines function in immune initiation and trigger a series of subsequent events in the context of disease is still a meaningful question to be resolved. The findings in this paper may provide clues to the pathway of clinical drugs.

In recent years, an increasing number of studies have focused on the therapeutic effects of natural active ingredients with anti-inflammatory properties on ocular diseases. In this study, we selected five natural active ingredients (anthocyanins, procyanidin, lutein, quercetin, and resveratrol) for molecular docking analysis with IL-18 and RANTES, two inflammation factors related to ocular diseases, aiming to enrich the treatment options for these diseases. Simona et al. searched and reviewed studies on the benefits of different natural active ingredients for some ocular diseases. The results showed that several polyphenols (such as anthocyanins, procyanidin, quercetin, and resveratrol) and carotenoids (such as lutein) have significant preventive and therapeutic effects on cataracts, glaucoma, DR, and AMD. The mechanisms involved include reducing the production of reactive oxygen species, inhibiting the tumor necrosis factor-α and VEGF pathways, inhibiting p53-dependent cell apoptosis, and inhibiting the production of inflammatory markers. Consuming products containing these natural active ingredients may have a protective effect on these diseases (24). The molecular docking analysis results in this study show that anthocyanins may have a good therapeutic effect on glaucoma. The specific mechanism needs further research.

The protective effect of RANTES on glaucoma in this study suggests that it may serve as a biomarker for early diagnosis and provide a basis for the development of neuroprotective therapies targeting the CCL5 pathway. In addition, the protective effect of IL-10 against diabetic retinopathy supports further development of recombinant IL-10 analogs (such as Pegilodecakin) that delay disease progression by regulating inflammation of the retinal microenvironment. The high affinity binding of natural active ingredients (such as anthocyanins) to the target provides a molecular basis for their conversion into topical eye drops or oral supplements, which may be a safe adjunct strategy for existing therapies.

Although the MR approach reduces confounding bias through genetic instrumental variables, there are potential limitations. First, genetic instrumental variables may affect outcomes through pleiotropic pathways (for example, SNPs of IL-18 may also regulate other inflammatory pathways), and residual pleiotropy may affect outcomes even though we have corrected outliers with MR-PRESSO. In addition, the MR analysis assumes that instrumental variables are independent of confounders, but certain unmeasured environmental factors (such as smoking or diet) may influence both inflammatory factor levels and eye disease risk. Because of the raw data not contained CCI scores, this study did not make adjustment comorbidities. Last, the possibility of reverse causality (such as the disease itself altering the expression of inflammatory factors), although reduced by the fixed nature of the genetic variation, needs to be further verified by two-way MR.

This study reveals the potential causal association between inflammatory factors and various eye diseases through two-sample Mendelian randomization analysis, but it should be noted that these results are highly sensitive to the screening threshold of instrumental variables. When a more stringent genome-wide significance threshold (*P* < 5 × 10^−8^) was applied, the association between AMD, glaucoma, diabetic retinopathy, myopia, and cataract with inflammatory factors was no longer statistically significant. This phenomenon may stem from a significant reduction in the number of SNPs available under strict thresholds (some inflammatory factors even have no eligible instrumental variables), leading to an increased risk of statistical underpotency or weak instrumental bias. Although this study validated the robustness of the original results through sensitivity analysis and multivariate models, these limitations suggest that the current conclusions may be constrained by race-specific genetic structure or the statistical power of GWAS. In the future, we need to further validate the biological mechanisms of key inflammatory factors through large cross-ethnic cohort, fine-mapping or functional experiments to clarify their causal role in the development of eye diseases.

## Data Availability

The original contributions presented in the study are included in the article/Supplementary material, further inquiries can be directed to the corresponding author.
