# Supplementary material for: Natural active ingredients targeted inflammatory cytokines and major blinding eye diseases: a two-sample Mendelian randomization and molecular docking analysis
Source: Front Med (Lausanne). 2025 Jul 4;12:1427144. doi: 10.3389/fmed.2025.1427144 (PMC12271126; doi:10.3389/fmed.2025.1427144)
Supplement: Supplementary file 1 [file Data_Sheet_1.docx]

| **Table S1. STROBE-MR checklist of the study.** | | | |
| --- | --- | --- | --- |
| Section | Item No. | Checklist item | Page No. |
| **Title and abstract** | 1 | Investigating the molecular mechanism of five natural active ingredients and inflammatory cytokines for the treatment of five ocular diseases by Mendelian randomization and molecular docking technology | 1-3 |
| **Introduction** |  |  |  |
| Background | 2 | Explain the scientific background and rationale for the reported study. What is the exposure? Is a potential causal relationship between exposure and outcome plausible? Justify why MR is a helpful method to address the study question | 4, 5 |
| Objectives | 3 | State specific objectives clearly, including pre-specified causal hypotheses (if any). State that MR is a method that, under specific assumptions, intends to estimate causal effects | 5 |
| **Methods** | | | |
| Study design and data sources | 4 | Present key elements of the study design early in the article. Consider including a table listing sources of data for all phases of the study. For each data source contributing to the analysis, describe the following: |  |
|  |  | a) Setting: Describe the study design and the underlying population, if possible. Describe the setting, locations, and relevant dates, including periods of recruitment, exposure, follow-up, and data collection, when available. | 6,7  Figure 1 |
|  |  | b) Participants: Give the eligibility criteria, and the sources and methods of selection of participants. Report the sample size, and whether any power or sample size calculations were carried out prior to the main analysis | 6,7  Table 1 |
|  |  | c) Describe measurement, quality control and selection of genetic variants | 6,7 |
|  |  | d) For each exposure, outcome, and other relevant variables, describe methods of assessment and diagnostic criteria for diseases | 6,7 |
|  |  | e) Provide details of ethics committee approval and participant informed consent, if relevant | 6 |
| Assumptions | 5 | Explicitly state the three core IV assumptions for the main analysis (relevance, independence and exclusion restriction) as well assumptions for any additional or sensitivity analysis | 6 |
| Statistical methods: main analysis | 6 | Describe statistical methods and statistics used |  |
|  |  | a) Describe how quantitative variables were handled in the analyses (i.e., scale, units, model) | 6-8 |
|  |  | b) Describe how genetic variants were handled in the analyses and, if applicable, how their weights were selected | 6-8 |
|  |  | c) Describe the MR estimator (e.g. two-stage least squares, Wald ratio) and related statistics. Detail the included covariates and, in case of two-sample MR, whether the same covariate set was used for adjustment in the two samples | 6-8 |
|  |  | d) Explain how missing data were addressed | N/A |
|  |  | e) If applicable, indicate how multiple testing was addressed | 6-8 |
| Assessment of assumptions | 7 | Describe any methods or prior knowledge used to assess the assumptions or justify their validity | 6-8 |
| Sensitivity analyses and additional analyses | 8 | Describe any sensitivity analyses or additional analyses performed (e.g. comparison of effect estimates from different approaches, independent replication, bias analytic techniques, validation of instruments, simulations) | 6-8 |
| Software and pre-registration | 9 | a) Name statistical software and package(s), including version and settings used | 8 |
|  |  | b) State whether the study protocol and details were pre-registered (as well as when and where) | N/A |
| **Results** | | | |
| Descriptive data | 10 | a) Report the numbers of individuals at each stage of included studies and reasons for exclusion. Consider use of a flow diagram | 9  Figure 1 |
|  |  | b) Report summary statistics for phenotypic exposure(s), outcome(s), and other relevant variables (e.g. means, SDs, proportions) | Table S2-16 |
|  |  | c) If the data sources include meta-analyses of previous studies, provide the assessments of heterogeneity across these studies | N/A |
|  |  | d) For two-sample MR:  i. Provide justification of the similarity of the genetic variant-exposure associations between the exposure and outcome samples  ii. Provide information on the number of individuals who overlap between the exposure and outcome studies | N/A |
| Main results | 11 | a) Report the associations between genetic variant and exposure, and between genetic variant and outcome, preferably on an interpretable scale | 9,10,  Figure 2-5 |
|  |  | b) Report MR estimates of the relationship between exposure and outcome, and the measures of uncertainty from the MR analysis, on an interpretable scale, such as odds ratio or relative risk per SD difference | 9,10,  Figure 2-5 |
|  |  | c) If relevant, consider translating estimates of relative risk into absolute risk for a meaningful time period | NA |
|  |  | d) Consider plots to visualize results (e.g. forest plot, scatterplot of associations between genetic variants and outcome versus between genetic variants and exposure) | Figure 2-5 |
| Assessment of assumptions | 12 | a) Report the assessment of the validity of the assumptions | 9,10 |
|  |  | b) Report any additional statistics (e.g., assessments of heterogeneity across genetic variants, such as I2, Q statistic or E-value) | 9,10, Table S2-16 |
| Sensitivity analyses and additional analyses | 13 | a) Report any sensitivity analyses to assess the robustness of the main results to violations of the assumptions | 9,10, Table S2-16 |
|  |  | b) Report results from other sensitivity analyses or additional analyses | 9,10, Table S2-16 |
|  |  | c) Report any assessment of direction of causal relationship (e.g., bidirectional MR) | N/A |
|  |  | d) When relevant, report and compare with estimates from non-MR analyses | N/A |
|  |  | e) Consider additional plots to visualize results (e.g., leave-one-out analyses) | Figure 5 |
| **Discussion** | | | |
| Key results | 14 | Summarize key results with reference to study objectives | 11 |
| Limitations | 15 | Discuss limitations of the study, taking into account the validity of the IV assumptions, other sources of potential bias, and imprecision. Discuss both direction and magnitude of any potential bias and any efforts to address them | 14 |
| Interpretation | 16 | a) Meaning: Give a cautious overall interpretation of results in the context of their limitations in comparison with other studies | 11-14 |
|  |  | b) Mechanism: Discuss underlying biological mechanisms that could drive a potential causal relationship between the investigated exposure and the outcome, and whether the gene-environment equivalence assumption is reasonable. Use causal language carefully, clarifying that IV estimates may provide causal effects only under certain assumptions | 11-14 |
|  |  | c) Clinical relevance: Discuss whether the results have clinical or public policy relevance, and to what extent they inform effect sizes of possible interventions | 11-14 |
| Generalizability | 17 | Discuss the generalizability of the study results (a) to other populations, (b) across other exposure periods/timings, and (c) across other levels of exposure | 11-14 |
| **Other information** | | | |
| Funding | 18 | Describe sources of funding and the role of funders in the present study and, if applicable, sources of funding for the databases and original study or studies on which the present study is based | 15 |
| Data and data sharing | 19 | Provide the data used to perform all analyses or report where and how the data can be accessed, and reference these sources in the article. Provide the statistical code needed to reproduce the results in the article, or report whether the code is publicly accessible and if so, where | 15 |
| Conflicts of Interest | 20 | All authors should declare all potential conflicts of interest | 15 |

**Table S2. MR estimates of 41 inflammatory cytokines on AMD.**

|  | **IVW** | | | | **MR-Egger** | | | | **Weighted Median** | | | | **Simple Mode** | | | | **Weighted Mode** | | | |
| --- | --- | --- | --- | --- | --- | --- | --- | --- | --- | --- | --- | --- | --- | --- | --- | --- | --- | --- | --- | --- |
| **Exposures** | **No.of SNPs** | **OR** | **95% CI** | **pval** | **No.of SNPs** | **OR** | **95% CI** | **pval** | **No.of SNPs** | **OR** | **95% CI** | **pval** | **No.of SNPs** | **OR** | **95% CI** | **pval** | **No.of SNPs** | **OR** | **95% CI** | **pval** |
| CTACK | 12 | 0.933 | (0.823-1.056) | 0.272 | 12 | 1.019 | (0.798-1.302) | 0.884 | 12 | 0.954 | (0.817-1.113) | 0.547 | 12 | 0.962 | (0.748-1.239) | 0.771 | 12 | 0.967 | (0.742-1.261) | 0.809 |
| Eotaxin | 17 | 1.040 | (0.909-1.190) | 0.567 | 17 | 1.122 | (0.808-1.558) | 0.503 | 17 | 1.060 | (0.876-1.281) | 0.551 | 17 | 1.134 | (0.816-1.575) | 0.465 | 17 | 1.072 | (0.825-1.391) | 0.611 |
| GROa | 13 | 1.069 | (0.956-1.195) | 0.241 | 13 | 1.066 | (0.877-1.297) | 0.534 | 13 | 1.057 | (0.910-1.227) | 0.534 | 13 | 1.019 | (0.791-1.313) | 0.888 | 13 | 1.039 | (0.808-1.336) | 0.772 |
| IP-10 | 12 | 1.030 | (0.873-1.214) | 0.728 | 12 | 1.068 | (0.726-1.571) | 0.745 | 12 | 0.967 | (0.809-1.157) | 0.745 | 12 | 0.886 | (0.650-1.207) | 0.458 | 12 | 0.886 | (0.661-1.187) | 0.434 |
| MCP-1 | 16 | 0.990 | (0.884-1.160) | 0.897 | 16 | 1.069 | (0.680-1.679) | 0.778 | 16 | 0.977 | (0.788-1.211) | 0.830 | 16 | 1.008 | (0.679-1.495) | 0.971 | 16 | 0.968 | (0.694-1.351) | 0.852 |
| MCP-3 | 6 | 1.012 | (0.918-1.116) | 0.811 | 6 | 1.010 | (0.780-1.308) | 0.943 | 6 | 0.979 | (0.866-1.107) | 0.738 | 6 | 0.937 | (0.760-1.156) | 0.571 | 6 | 0.936 | (0.788-1.112) | 0.488 |
| MIG | 13 | 0.998 | (0.892-1.117) | 0.977 | 13 | 1.089 | (0.860-1.379) | 0.495 | 13 | 0.991 | (0.845-1.163) | 0.916 | 13 | 1.008 | (0.755-1.346) | 0.958 | 13 | 0.929 | (0.700-1.233) | 0.620 |
| MIP-1A | 4 | 1.016 | (0.816-1.265) | 0.885 | 4 | 1.079 | (0.548-2.125) | 0.846 | 4 | 1.017 | (0.789-1.310) | 0.897 | 4 | 1.012 | (0.727-1.410) | 0.947 | 4 | 1.018 | (0.724-1.432) | 0.924 |
| MIP-1B | 22 | 0.995 | (0.881-1.123) | 0.929 | 22 | 1.061 | (0.754-1.493) | 0.736 | 22 | 0.971 | (0.822-1.148) | 0.733 | 22 | 0.879 | (0.643-1.201) | 0.427 | 22 | 0.917 | (0.689-1.221) | 0.560 |
| RANTES | 10 | 1.089 | (0.953-1.246) | 0.210 | 10 | 0.843 | (0.604-1.178) | 0.346 | 10 | 1.067 | (0.895-1.271) | 0.469 | 10 | 0.974 | (0.735-1.292) | 0.859 | 10 | 0.972 | (0.741-1.275) | 0.842 |
| SDF-1A | 9 | 0.899 | (0.736-1.098) | 0.297 | 9 | 0.772 | (0.519-1.148) | 0.242 | 9 | 0.966 | (0.747-1.249) | 0.792 | 9 | 1.004 | (0.670-1.505) | 0.986 | 9 | 1.006 | (0.675-1.501) | 0.976 |
| SCGF-B | 21 | 1.06 | (0.965-1.161) | 0.229 | 21 | 1.060 | (0.870-1.290) | 0.571 | 21 | 1.123 | (0.998-1.264) | 0.054 | 21 | 1.145 | (0.930-1.410) | 0.216 | 21 | 1.136 | (0.948-1.361) | 0.183 |
| B-NGF | 4 | 1.200 | (0.919-1.569) | 0.181 | 4 | 3.205 | (1.131-9.079) | 0.160 | 4 | 1.224 | (0.948-1.581) | 0.122 | 4 | 1.275 | (0.857-1.897) | 0.317 | 4 | 1.246 | (0.852-1.823) | 0.340 |
| FGF2 | 7 | 0.991 | (0.783-1.254) | 0.941 | 7 | 1.209 | (0.608-2.404) | 0.612 | 7 | 0.922 | (0.679-1.254) | 0.606 | 7 | 0.909 | (0.577-1.430) | 0.693 | 7 | 0.909 | (0.582-1.419) | 0.688 |
| G-CSF | 9 | 1.109 | (0.915-1.346) | 0.292 | 9 | 1.146 | (0.824-1.592) | 0.445 | 9 | 0.988 | (0.767-1.273) | 0.928 | 9 | 0.945 | (0.611-1.460) | 0.805 | 9 | 0.936 | (0.620-1.414) | 0.763 |
| HGF | 9 | 1.037 | (0.843-1.275) | 0.730 | 9 | 1.243 | (0.789-1.959) | 0.380 | 9 | 0.968 | (0.734-1.276) | 0.815 | 9 | 0.923 | (0.613-1.391) | 0.712 | 9 | 0.943 | (0.674-1.319) | 0.740 |
| M-CSF | 12 | 1.031 | (0.937-1.135) | 0.527 | 12 | 1.070 | (0.876-1.307) | 0.521 | 12 | 1.050 | (0.916-1.203) | 0.483 | 12 | 1.123 | (0.894-1.410) | 0.342 | 12 | 1.073 | (0.880-1.310) | 0.500 |
| PDGF-BB | 14 | 0.804 | (0.678-0.954) | 0.012 | 14 | 0.695 | (0.461-1.047) | 0.108 | 14 | 0.817 | (0.649-1.030) | 0.087 | 14 | 0.711 | (0.463-1.094) | 0.145 | 14 | 0.730 | (0.499-1.069) | 0.130 |
| SCF | 10 | 1.053 | (0.863-1.283) | 0.612 | 10 | 1.126 | (0.742-1.709) | 0.593 | 10 | 1.111 | (0.856-1.442) | 0.427 | 10 | 1.086 | (0.749-1.575) | 0.673 | 10 | 1.082 | (0.758-1.543) | 0.673 |
| VEGF | 18 | 0.997 | (0.865-1.150) | 0.968 | 18 | 1.046 | (0.762-1.435) | 0.784 | 18 | 0.934 | (0.781-1.116) | 0.451 | 18 | 0.852 | (0.602-1.206) | 0.380 | 18 | 0.817 | (0.580-1.152) | 0.266 |
| IL-10 | 15 | 1.099 | (0.959-1.260) | 0.173 | 15 | 1.446 | (0.928-2.255) | 0.127 | 15 | 1.107 | (0.909-1.350) | 0.312 | 15 | 1.026 | (0.714-1.475) | 0.892 | 15 | 1.340 | (1.043-1.722) | 0.038 |
| IL-12-p70 | 15 | 1.041 | (0.890-1.218) | 0.614 | 15 | 1.199 | (0.681-2.111) | 0.540 | 15 | 1.064 | (0.854-1.326) | 0.579 | 15 | 0.947 | (0.633-1.416) | 0.793 | 15 | 1.080 | (0.752-1.550) | 0.684 |
| IL-13 | 14 | 1.030 | (0.929-1.142 | 0.578 | 14 | 1.072 | (0.884-1.301 | 0.492 | 14 | 1.055 | (0.919-1.211) | 0.446 | 14 | 1.064 | (0.838-1.351) | 0.621 | 14 | 1.077 | (0.864-1.343 | 0.519 |
| IL-16 | 10 | 0.937 | (0.837-1.049) | 0.256 | 10 | 1.049 | (0.873-1.261) | 0.625 | 10 | 0.886 | (0.759-1.035) | 0.127 | 10 | 0.844 | (0.665-1.071) | 0.195 | 10 | 0.862 | (0.689-1.078) | 0.224 |
| IL-17 | 8 | 1.029 | (0.854-1.239) | 0.764 | 8 | 1.061 | (0.751-1.499) | 0.749 | 8 | 0.992 | (0.775-1.269) | 0.947 | 8 | 0.991 | (0.677-1.449) | 0.964 | 8 | 0.951 | (0.710-1.275) | 0.748 |
| IL-18 | 13 | 1.134 | (1.009-1.275) | 0.034 | 13 | 1.198 | (0.952-1.509) | 0.152 | 13 | 1.094 | (0.931-1.286) | 0.274 | 13 | 1.013 | (0.756-1.358) | 0.932 | 13 | 1.019 | (0.754-1.375) | 0.907 |
| IL-1B | 3 | 1.108 | (0.788-1.558) | 0.555 | 3 | 0.943 | (0.386-2.302) | 0.918 | 3 | 1.226 | (0.901-1.668) | 0.196 | 3 | 1.226 | (0.848-1.772) | 0.392 | 3 | 1.226 | (0.899-1.673) | 0.327 |
| IL-1RA | 10 | 1.104 | (0.962-1.267) | 0.157 | 10 | 1.117 | (0.761-1.637) | 0.588 | 10 | 1.133 | (0.950-1.350) | 0.165 | 10 | 1.176 | (0.870-1.589) | 0.319 | 10 | 1.173 | (0.883-1.559) | 0.299 |
| IL-2 | 8 | 1.026 | (0.896-1.174) | 0.714 | 8 | 1.016 | (0.778-1.329) | 0.909 | 8 | 1.016 | (0.853-1.211) | 0.855 | 8 | 1.183 | (0.903-1.548) | 0.262 | 8 | 1.003 | (0.801-1.257) | 0.978 |
| IL-2RA | 9 | 0.941 | (0.817-1.084) | 0.821 | 9 | 0.771 | (0.614-0.969) | 0.061 | 9 | 0.949 | (0.804-1.122) | 0.541 | 9 | 0.966 | (0.726-1.287) | 0.821 | 9 | 0.955 | (0.756-1.206) | 0.707 |
| IL-4 | 14 | 0.986 | (0.834-1.165) | 0.868 | 14 | 1.143 | (0.810-1.613) | 0.461 | 14 | 1.031 | (0.825-1.288) | 0.791 | 14 | 0.996 | (0.683-1.452) | 0.983 | 14 | 1.031 | (0.729-1.458) | 0.866 |
| IL-5 | 8 | 0.922 | (0.800-1.063) | 0.266 | 8 | 0.889 | (0.652-1.214) | 0.488 | 8 | 0.938 | (0.786-1.120) | 0.480 | 8 | 0.958 | (0.736-1.247) | 0.760 | 8 | 0.966 | (0.742-1.259) | 0.807 |
| IL-6 | 11 | 1.004 | (0.827-1.221) | 0.963 | 11 | 1.038 | (0.655-1.645) | 0.878 | 11 | 1.000 | (0.771-1.297) | 0.999 | 11 | 0.977 | (0.637-1.497) | 0.916 | 11 | 0.965 | (0.635-1.466) | 0.871 |
| IL-7 | 12 | 1.064 | (0.958-1.181) | 0.246 | 12 | 0.946 | (0.741-1.206) | 0.662 | 12 | 1.043 | (0.904-1.204) | 0.563 | 12 | 1.091 | (0.880-1.351) | 0.445 | 12 | 1.058 | (0.883-1.267) | 0.555 |
| IL-8 | 8 | 0.936 | (0.808-1.085) | 0.382 | 8 | 0.880 | (0.670-1.157) | 0.396 | 8 | 0.928 | (0.777-1.108) | 0.407 | 8 | 0.924 | (0.705-1.210) | 0.583 | 8 | 0.917 | (0.691-1.218) | 0.570 |
| IL-9 | 6 | 0.866 | (0.719-1.045) | 0.133 | 6 | 1.018 | (0.647-1.602) | 0.942 | 6 | 0.809 | (0.641-1.020) | 0.073 | 6 | 0.792 | (0.592-1.059) | 0.176 | 6 | 0.792 | (0.594-1.056) | 0.173 |
| IFN-G | 12 | 1.005 | (0.846-1.193) | 0.959 | 12 | 1.054 | (0.731-1.519) | 0.783 | 12 | 1.066 | (0.844-1.345) | 0.593 | 12 | 1.216 | (0.813-1.818) | 0.362 | 12 | 1.192 | (0.858-1.656) | 0.316 |
| MIF | 10 | 0.987 | (0.860-1.132 | 0.849 | 10 | 1.054 | (0.805-1.380) | 0.711 | 10 | 0.935 | (0.773-1.130) | 0.487 | 10 | 0.926 | (0.710-1.207) | 0.582 | 10 | 0.935 | (0.693-1.261) | 0.669 |
| TNF-A | 4 | 1.071 | (0.815-1.408) | 0.624 | 4 | 0.856 | (0.612-1.197) | 0.460 | 4 | 1.083 | (0.841-1.396) | 0.535 | 4 | 1.096 | (0.734-1.635) | 0.686 | 4 | 1.084 | (0.747-1.574) | 0.700 |
| TNF-B | 5 | 0.935 | (0.815-1.072) | 0.334 | 5 | 1.135 | (0.897-1.437) | 0.370 | 5 | 0.939 | (0.813-1.085) | 0.393 | 5 | 0.816 | (0.634-1.050) | 0.189 | 5 | 1.027 | (0.828-1.273) | 0.821 |
| TRAIL | 16 | 1.136 | (0.989-1.305) | 0.072 | 16 | 1.086 | (0.882-1.336) | 0.449 | 16 | 1.063 | (0.919-1.230) | 0.411 | 16 | 1.125 | (0.889-1.425) | 0.343 | 16 | 1.065 | (0.929-1.221) | 0.380 |

Abbreviations: AMD, age-related macular degeneration; CI, Confidence interval; OR, Odds Ratio; pval, P-value; SNPs, single nucleotide polymorphisms. OR and 95% CI represent the change in the odds ratio of AMD per 1 SD increase in inflammatory cytokines.

**Table S3. Heterogeneity and horizontal pleiotropy tests of 41 inflammatory cytokines on AMD**

| **Exposures** | **Q_1_ pval (IVW)** | **Q_2_ pval (MR-Egger)** | **I^2^** | **intercept** | **intercept pval** |
| --- | --- | --- | --- | --- | --- |
| B-NGF | 0.152 | 0.418 | 43.3% | -0.140 | 0.200 |
| CTACK | 0.843 | 0.836 | 0.0% | -0.022 | 0.429 |
| Eotaxin | 0.745 | 0.699 | 0.0% | -0.010 | 0.628 |
| FGF2 | 0.788 | 0.730 | 0.0% | -0.026 | 0.573 |
| G-CSF | 0.686 | 0.587 | 0.0% | -0.005 | 0.821 |
| GROa | 0.888 | 0.838 | 0.0% | 0.001 | 0.976 |
| HGF | 0.503 | 0.477 | 0.0% | -0.026 | 0.410 |
| IFN-G | 0.413 | 0.337 | 3.2% | -0.007 | 0.773 |
| IL-1B | 0.196 | 0.095 | 38.7% | 0.033 | 0.753 |
| IL-1RA | 0.846 | 0.772 | 0.0% | -0.002 | 0.952 |
| IL-2 | 0.671 | 0.556 | 0.0% | 0.002 | 0.942 |
| IL-2RA | 0.228 | 0.490 | 24.2% | 0.044 | 0.082 |
| IL-4 | 0.567 | 0.563 | 0.0% | -0.021 | 0.355 |
| IL-5 | 0.955 | 0.918 | 0.0% | 0.008 | 0.804 |
| IL-6 | 0.395 | 0.311 | 5.0% | -0.004 | 0.881 |
| IL-7 | 0.903 | 0.926 | 0.0% | 0.026 | 0.318 |
| IL-8 | 0.959 | 0.943 | 0.0% | 0.013 | 0.618 |
| IL-9 | 0.563 | 0.506 | 0.0% | -0.004 | 0.486 |
| IL-10 | 0.764 | 0.820 | 0.0% | -0.031 | 0.226 |
| IL-12p70 | 0.563 | 0.505 | 0.0% | -0.015 | 0.619 |
| IL-13 | 0.931 | 0.909 | 0.0% | -0.010 | 0.635 |
| IL-16 | 0.521 | 0.669 | 0.0% | -0.036 | 0.165 |
| IL-17 | 0.889 | 0.819 | 0.0% | -0.006 | 0.843 |
| IL-18 | 0.399 | 0.344 | 4.8% | -0.011 | 0.595 |
| IP-10 | 0.049 | 0.033 | 44.2% | -0.007 | 0.839 |
| M-CSF | 0.615 | 0.541 | 0.0% | -0.013 | 0.689 |
| MCP-1 | 0.358 | 0.300 | 8.4% | -0.010 | 0.726 |
| MCP-3 | 0.690 | 0.548 | 0.0% | 0.001 | 0.989 |
| MIF | 0.526 | 0.456 | 0.0% | -0.014 | 0.591 |
| MIG | 0.444 | 0.417 | 0.1% | -0.022 | 0.428 |
| MIP-1A | 0.957 | 0.869 | 0.0% | -0.010 | 0.871 |
| MIP-1B | 0.807 | 0.768 | 0.0% | -0.009 | 0.694 |
| PDGF-BB | 0.418 | 0.387 | 2.9% | 0.018 | 0.457 |
| RANTES | 0.793 | 0.948 | 0.0% | 0.052 | 0.139 |
| SCF | 0.502 | 0.415 | 0.0% | -0.010 | 0.728 |
| SCGF-B | 0.320 | 0.266 | 10.6% | -0.001 | 0.989 |
| SDF-1A | 0.874 | 0.880 | 0.0% | 0.021 | 0.413 |
| TNF-A | 0.144 | 0.330 | 44.6% | 0.067 | 0.231 |
| TNF-B | 0.232 | 0.545 | 28.4% | -0.060 | 0.160 |
| TRAIL | 0.047 | 0.039 | 40.5% | 0.010 | 0.569 |
| VEGF | 0.161 | 0.128 | 24.9% | -0.008 | 0.742 |

Q1 pval: P-value of Q test from IVW method; Q2 pval: P-value of Q test from MR-Egger method.

Abbreviations: AMD, age-related macular degeneration; pval, P-value; Q, Cochran Q statistics; SNPs, single nucleotide polymorphisms; IVW, the inverse variance weighted method.

**Table S4. SNPs information of 41 inflammation cytokines with AMD**

|  | **Inflammatory cytokines (exposure)** | | | | | **AMD (outcome)** | | |
| --- | --- | --- | --- | --- | --- | --- | --- | --- |
| **SNP** | **effect allele** | **other allele** | **Beta** | **se** | **pval** | **F** | **se** | **pval** |
| **B-NGF** | | | | | | | | |
| rs28637706 | T | G | -0.1554 | 0.0261 | 2.717E-09 | 35.4305296 | 0.0272 | 0.2881 |
| rs4767014 | T | C | -0.1211 | 0.0264 | 4.518E-06 | 21.029893 | 0.0268 | 0.287 |
| rs71641308 | T | C | 0.1969 | 0.0429 | 4.424E-06 | 21.0539447 | 0.0429 | 0.0209701 |
| rs73472576 | T | C | -0.1146 | 0.0251 | 4.813E-06 | 20.8342764 | 0.0253 | 0.355 |
| **CTACK** | | | | | | | | |
| rs10854859 | A | G | -0.1498 | 0.0293 | 3.049E-07 | 26.1247203 | 0.0301 | 0.6766 |
| rs116303454 | A | G | 0.3754 | 0.081 | 3.579E-06 | 21.4675143 | 0.0781 | 0.2903 |
| rs116871507 | A | T | -0.2086 | 0.0448 | 0.00000316 | 21.668849 | 0.049 | 0.8519 |
| rs116943377 | A | G | 0.2878 | 0.0611 | 2.496E-06 | 22.1749384 | 0.0661 | 0.3534 |
| rs117932939 | T | C | 0.1969 | 0.0422 | 3.089E-06 | 21.7585421 | 0.0457 | 0.0634001 |
| rs118084576 | A | G | 0.5675 | 0.1226 | 0.00000366 | 21.4148015 | 0.1432 | 0.3429 |
| rs184329319 | T | G | -0.3069 | 0.0648 | 2.173E-06 | 22.4185246 | 0.0714 | 0.792 |
| rs55764737 | T | C | 0.5424 | 0.0967 | 2.012E-08 | 31.4448608 | 0.1022 | 0.5943 |
| rs57338032 | A | G | 0.1443 | 0.0316 | 4.831E-06 | 20.8411539 | 0.0333 | 0.4075 |
| rs57789542 | T | C | -0.7687 | 0.1659 | 3.575E-06 | 21.4577535 | 0.1486 | 0.95 |
| rs60247384 | T | C | 0.1128 | 0.0245 | 4.302E-06 | 21.1860159 | 0.0263 | 0.9845 |
| rs76395525 | A | G | 0.5193 | 0.1081 | 1.553E-06 | 23.0647377 | 0.1232 | 0.8083 |
| **Eotaxin** | | | | | | | | |
| rs11087905 | A | C | 0.0954 | 0.0188 | 4.07E-07 | 25.7439435 | 0.0266 | 0.3035 |
| rs112347425 | T | C | 0.1595 | 0.0276 | 7.771E-09 | 33.388525 | 0.0438 | 0.695401 |
| rs11920996 | T | C | 0.2979 | 0.0377 | 2.919E-15 | 62.4241056 | 0.0594 | 0.7514 |
| rs1677588 | T | G | 0.1181 | 0.025 | 2.223E-06 | 22.310731 | 0.0396 | 0.5518 |
| rs2024050 | A | G | 0.164 | 0.0302 | 5.467E-08 | 29.4827394 | 0.0485 | 0.2788 |
| rs2027855 | T | C | 0.0743 | 0.0162 | 4.272E-06 | 21.0301137 | 0.0257 | 0.771401 |
| rs2040143 | A | G | -0.0858 | 0.0178 | 1.333E-06 | 23.2288973 | 0.0279 | 0.683501 |
| rs2229593 | T | C | 0.3647 | 0.0406 | 2.838E-19 | 80.6702648 | 0.0637 | 0.9212 |
| rs2249581 | T | C | -0.0899 | 0.018 | 5.912E-07 | 24.9383891 | 0.0287 | 0.3582 |
| rs5754733 | A | C | -0.105 | 0.0213 | 8.196E-07 | 24.2948048 | 0.0332 | 0.7201 |
| rs57723662 | C | G | -0.0982 | 0.0213 | 3.878E-06 | 21.2499441 | 0.033 | 0.0701908 |
| rs60075014 | T | C | -0.1688 | 0.0356 | 2.078E-06 | 22.4770293 | 0.0511 | 0.2675 |
| rs7231030 | A | C | 0.0903 | 0.0193 | 2.709E-06 | 21.885421 | 0.0304 | 0.8647 |
| rs73072941 | A | T | -0.1281 | 0.0263 | 1.113E-06 | 23.7181486 | 0.0393 | 0.5396 |
| rs745331 | A | G | -0.0821 | 0.0176 | 3.036E-06 | 21.7547953 | 0.0271 | 0.2661 |
| rs75426604 | A | C | -0.1371 | 0.0291 | 2.397E-06 | 22.1913107 | 0.0434 | 0.2298 |
| rs9317045 | A | C | 0.1172 | 0.0236 | 6.954E-07 | 24.6561487 | 0.0355 | 0.4252 |
| **FGF2** | | | | | | | | |
| rs13412535 | A | G | -0.1129 | 0.0224 | 4.763E-07 | 25.3967228 | 0.0315 | 0.727601 |
| rs147409637 | T | C | 0.201 | 0.0431 | 3.077E-06 | 21.7431973 | 0.0697 | 0.4377 |
| rs17094040 | T | C | 0.1051 | 0.0229 | 4.308E-06 | 21.0581541 | 0.0361 | 0.3893 |
| rs2849358 | A | G | 0.0911 | 0.0193 | 2.321E-06 | 22.2745002 | 0.0289 | 0.6236 |
| rs4795091 | A | G | 0.1239 | 0.0266 | 3.055E-06 | 21.6902807 | 0.0404 | 0.81 |
| rs76253061 | T | C | -0.4811 | 0.1041 | 3.807E-06 | 21.3528125 | 0.1534 | 0.8445 |
| rs78873483 | A | G | 0.1286 | 0.0282 | 0.00000498 | 20.7907211 | 0.0395 | 0.2423 |
| **G-CSF** | | | | | | | | |
| rs10939033 | A | G | -0.0775 | 0.0163 | 2.074E-06 | 22.6005443 | 0.0255 | 0.9879 |
| rs117261691 | T | C | 0.1318 | 0.0288 | 4.669E-06 | 20.9380652 | 0.046 | 0.271 |
| rs183023730 | T | G | 0.7898 | 0.1677 | 2.471E-06 | 22.1734096 | 0.1913 | 0.1128 |
| rs586802 | A | G | 0.0882 | 0.0187 | 2.398E-06 | 22.2405058 | 0.029 | 0.9435 |
| rs6740648 | T | C | 0.0818 | 0.0172 | 0.0000019 | 22.6120749 | 0.0269 | 0.5332 |
| rs74148555 | T | C | -0.3771 | 0.0753 | 5.591E-07 | 25.0734338 | 0.0877 | 0.6489 |
| rs76287671 | T | C | 0.0894 | 0.0189 | 2.191E-06 | 22.3687714 | 0.0296 | 0.1471 |
| **GROa** | | | | | | | | |
| rs114991247 | T | C | -0.2202 | 0.0463 | 1.971E-06 | 22.6061961 | 0.0427 | 0.0863694 |
| rs115214168 | T | C | 0.4528 | 0.0828 | 4.479E-08 | 29.8886987 | 0.087 | 0.9988 |
| rs1361829 | A | G | -0.1106 | 0.0241 | 4.576E-06 | 21.0489779 | 0.0252 | 0.2877 |
| rs140734053 | A | G | 0.7333 | 0.1545 | 2.069E-06 | 22.514445 | 0.13 | 0.2646 |
| rs150194856 | T | C | -0.4223 | 0.0914 | 3.857E-06 | 21.3355879 | 0.0985 | 0.9127 |
| rs17171245 | T | G | 0.2446 | 0.053 | 3.932E-06 | 21.287076 | 0.059 | 0.7687 |
| rs185768063 | A | G | 0.4038 | 0.076 | 1.055E-07 | 28.2137159 | 0.077 | 0.3762 |
| rs188345231 | T | C | 0.6177 | 0.1322 | 2.968E-06 | 21.8196176 | 0.1412 | 0.9537 |
| rs3026943 | A | C | -0.1246 | 0.0256 | 1.084E-06 | 23.6761454 | 0.0265 | 0.5403 |
| rs62024303 | A | G | -0.3013 | 0.066 | 4.908E-06 | 20.8288473 | 0.0628 | 0.408 |
| rs76215157 | C | G | -0.7398 | 0.1564 | 2.226E-06 | 22.3619669 | 0.1253 | 0.7582 |
| rs76390238 | C | G | 0.6223 | 0.1352 | 4.141E-06 | 21.17391 | 0.1356 | 0.5639 |
| rs79454658 | T | C | 0.2784 | 0.0596 | 3.017E-06 | 21.8072442 | 0.0622 | 0.6584 |
| **HGF** | | | | | | | | |
| rs11060254 | A | G | -0.0765 | 0.0166 | 3.974E-06 | 21.2325661 | 0.0146 | 0.5866 |
| rs11129909 | T | C | -0.0738 | 0.0161 | 4.457E-06 | 21.0066482 | 0.0299 | 0.2485 |
| rs13412535 | A | G | -0.1043 | 0.0213 | 9.671E-07 | 23.9720514 | 0.011 | 0.727601 |
| rs2003620 | T | C | 0.2277 | 0.0487 | 2.978E-06 | 21.8556602 | 0.0765 | 0.3083 |
| rs362307 | T | C | 0.1511 | 0.0328 | 0.00000421 | 21.2166607 | 0.0643 | 0.1954 |
| rs4245058 | T | C | -0.1552 | 0.0331 | 2.683E-06 | 21.9797747 | -0.0018 | 0.9723 |
| rs57146176 | A | G | -0.0987 | 0.0208 | 2.184E-06 | 22.5114477 | -0.0857 | 0.0886094 |
| rs5745687 | T | C | -0.3008 | 0.0404 | 9.922E-14 | 55.4228333 | 0.0071 | 0.9093 |
| rs80051150 | T | C | 0.198 | 0.0413 | 1.684E-06 | 22.9787324 | -0.0159 | 0.8173 |
| **IFN-G** | | | | | | | | |
| rs113399544 | A | G | -0.0849 | 0.0183 | 3.323E-06 | 21.5179571 | 0.0277 | 0.8581 |
| rs113600793 | A | C | 0.1871 | 0.0371 | 4.426E-07 | 25.4265591 | 0.0538 | 0.296 |
| rs115729819 | A | G | 0.2511 | 0.0514 | 1.045E-06 | 23.8591531 | 0.0784 | 0.4346 |
| rs117046255 | T | C | -0.0968 | 0.0207 | 2.787E-06 | 21.8624013 | 0.0323 | 0.3103 |
| rs11843756 | T | G | 0.1812 | 0.0391 | 3.622E-06 | 21.4709227 | 0.0622 | 0.7668 |
| rs12420286 | T | C | 0.2357 | 0.05 | 2.452E-06 | 22.2160576 | 0.0754 | 0.5326 |
| rs147378920 | A | G | -0.384 | 0.0751 | 3.195E-07 | 26.1378831 | 0.0892 | 0.675601 |
| rs1867282 | T | C | 0.0781 | 0.0166 | 2.478E-06 | 22.1296084 | 0.0254 | 0.5162 |
| rs2073438 | A | G | 0.092 | 0.0188 | 9.551E-07 | 23.9413036 | 0.0287 | 0.0148898 |
| rs7088799 | T | G | -0.0805 | 0.0166 | 1.274E-06 | 23.5105843 | 0.0257 | 0.2846 |
| rs73479333 | C | G | -0.1123 | 0.024 | 2.816E-06 | 21.8889468 | 0.0375 | 0.6244 |
| rs74148555 | T | C | -0.3771 | 0.077 | 9.858E-07 | 23.9783586 | 0.0877 | 0.6489 |
| **IL-1B** | | | | | | | | |
| rs143319329 | T | C | 0.4357 | 0.093 | 2.835E-06 | 21.9356097 | 0.1558 | 0.1666 |
| rs4786740 | A | C | 0.1264 | 0.0265 | 0.00000182 | 22.7375092 | 0.0256 | 0.3118 |
| rs61335305 | A | C | 0.4333 | 0.0928 | 3.015E-06 | 21.7882281 | 0.0928 | 0.344 |
| **IL-1RA** | | | | | | | | |
| rs1054402 | T | C | 0.1325 | 0.0269 | 8.201E-07 | 24.2488368 | 0.0289 | 0.0676208 |
| rs117181659 | A | G | -0.2204 | 0.0478 | 0.00000392 | 21.2486381 | 0.0519 | 0.642899 |
| rs11869294 | C | G | -0.2286 | 0.047 | 1.128E-06 | 23.6439727 | 0.0468 | 0.9797 |
| rs13343438 | A | G | 0.2771 | 0.0607 | 4.973E-06 | 20.8285666 | 0.0648 | 0.4482 |
| rs35590641 | C | G | -0.1167 | 0.025 | 3.041E-06 | 21.7783718 | 0.0264 | 0.4739 |
| rs3876037 | A | G | 0.1234 | 0.027 | 4.733E-06 | 20.8769237 | 0.0261 | 0.8399 |
| rs56134659 | A | G | -0.1109 | 0.0236 | 2.564E-06 | 22.0700237 | 0.025 | 0.3839 |
| rs61335305 | A | C | 0.4315 | 0.0904 | 1.812E-06 | 22.7713329 | 0.0928 | 0.344 |
| rs6699436 | A | G | -0.1858 | 0.0404 | 4.365E-06 | 21.1393876 | 0.0403 | 0.4877 |
| rs9985296 | T | C | 0.1053 | 0.0231 | 4.952E-06 | 20.7680871 | 0.0251 | 0.8412 |
| **IL-2** | | | | | | | | |
| rs13412535 | A | G | 0.174 | 0.0331 | 1.447E-07 | 27.6181929 | 0.0315 | 0.727601 |
| rs16836080 | A | G | 0.1158 | 0.0253 | 4.841E-06 | 20.9376929 | 0.0268 | 0.2943 |
| rs2690020 | A | G | 0.1158 | 0.0245 | 2.273E-06 | 22.3273767 | 0.0252 | 0.3009 |
| rs4479767 | A | G | 0.1821 | 0.0392 | 3.444E-06 | 21.5675348 | 0.0432 | 0.4419 |
| rs4634519 | A | G | -0.1249 | 0.0268 | 0.00000318 | 21.7074115 | 0.028 | 0.6856 |
| rs61335305 | A | C | 0.4439 | 0.0913 | 1.157E-06 | 23.6255259 | 0.0928 | 0.344 |
| rs62124990 | T | G | -0.7013 | 0.149 | 2.502E-06 | 22.1405238 | 0.0916 | 0.8917 |
| rs7615304 | A | G | -0.1139 | 0.024 | 2.161E-06 | 22.5101151 | 0.0251 | 0.2989 |
| **IL-2RA** | | | | | | | | |
| rs11241559 | T | G | -0.124 | 0.0264 | 0.00000275 | 22.0496505 | 0.0288 | 0.2242 |
| rs117244812 | A | G | -0.7187 | 0.1493 | 1.474E-06 | 23.1601767 | 0.148 | 0.02476 |
| rs12789243 | T | C | 0.1263 | 0.0276 | 4.608E-06 | 20.9293013 | 0.0291 | 0.1369 |
| rs17147986 | A | C | -0.2976 | 0.0337 | 1.069E-18 | 77.9421257 | 0.0357 | 0.1162 |
| rs17624670 | A | G | -0.125 | 0.0273 | 4.639E-06 | 20.9537099 | 0.0289 | 0.8423 |
| rs34037190 | A | G | 0.4784 | 0.0935 | 3.134E-07 | 26.1652756 | 0.0778 | 0.9988 |
| rs56213152 | T | C | 0.1269 | 0.0271 | 2.937E-06 | 21.9154734 | 0.0292 | 0.8633 |
| rs759244 | A | T | -0.1094 | 0.0238 | 4.257E-06 | 21.117715 | 0.0256 | 0.8375 |
| rs79100208 | C | G | 0.8345 | 0.1758 | 2.052E-06 | 22.5206611 | 0.1566 | 0.669501 |
| **IL-4** | | | | | | | | |
| rs116705532 | T | G | -0.4675 | 0.0978 | 1.727E-06 | 22.844369 | 0.1561 | 0.4351 |
| rs117146485 | T | C | -0.2856 | 0.0625 | 4.945E-06 | 20.8761312 | 0.0943 | 0.8441 |
| rs12238729 | T | C | 0.5271 | 0.1096 | 1.505E-06 | 23.116432 | 0.1965 | 0.8052 |
| rs12640583 | T | G | -0.1104 | 0.0214 | 2.451E-07 | 26.6075108 | 0.0337 | 0.8543 |
| rs17713451 | A | G | 0.1255 | 0.0252 | 6.41E-07 | 24.795908 | 0.0394 | 0.347 |
| rs1867282 | T | C | 0.0808 | 0.0162 | 5.822E-07 | 24.8706044 | 0.0254 | 0.5162 |
| rs2073438 | A | G | 0.0847 | 0.0183 | 3.725E-06 | 21.4169828 | 0.0287 | 0.0148898 |
| rs2346020 | A | G | 0.079 | 0.0169 | 2.843E-06 | 21.8461253 | 0.0267 | 0.7873 |
| rs2708586 | T | C | -0.0767 | 0.0166 | 3.588E-06 | 21.3436258 | 0.0264 | 0.7696 |
| rs56408830 | A | G | -0.1794 | 0.0365 | 9.075E-07 | 24.151983 | 0.0611 | 0.3859 |
| rs7613691 | A | G | 0.1787 | 0.0382 | 2.962E-06 | 21.8784235 | 0.0578 | 0.8387 |
| rs79597994 | T | C | -0.5855 | 0.1271 | 4.056E-06 | 21.2156501 | 0.1443 | 0.5104 |
| rs9506111 | A | G | -0.1446 | 0.0314 | 4.081E-06 | 21.2017123 | 0.0498 | 0.1411 |
| rs9941733 | A | G | 0.1156 | 0.0229 | 4.331E-07 | 25.4764171 | 0.033 | 0.8733 |
| **IL-5** | | | | | | | | |
| rs10178043 | T | G | 0.2579 | 0.0553 | 3.126E-06 | 21.7368748 | 0.0556 | 0.8229 |
| rs148634917 | A | G | -0.517 | 0.1087 | 1.974E-06 | 22.6082361 | 0.0935 | 0.3673 |
| rs28793375 | T | C | 0.1697 | 0.0362 | 2.746E-06 | 21.962978 | 0.037 | 0.8501 |
| rs72831687 | A | G | -0.5337 | 0.1104 | 1.324E-06 | 23.3561331 | 0.1001 | 0.8979 |
| rs73040118 | T | C | 0.2294 | 0.049 | 2.903E-06 | 21.9048029 | 0.0514 | 0.673899 |
| rs74811276 | A | G | 0.217 | 0.0471 | 4.082E-06 | 21.2139889 | 0.0455 | 0.2238 |
| rs7739450 | A | G | -0.1295 | 0.0256 | 4.05E-07 | 25.5743314 | 0.0254 | 0.734 |
| rs9309063 | T | G | -0.1119 | 0.0245 | 4.866E-06 | 20.8483979 | 0.0253 | 0.4235 |
| **IL-6** | | | | | | | | |
| rs10910395 | A | T | -0.108 | 0.0235 | 4.348E-06 | 21.1157384 | 0.0367 | 0.9699 |
| rs10982193 | A | G | -0.0793 | 0.0174 | 4.816E-06 | 20.7654986 | 0.028 | 0.5199 |
| rs113098456 | A | G | -0.1553 | 0.0339 | 4.641E-06 | 20.9815709 | 0.0482 | 0.0363103 |
| rs113600793 | A | C | 0.1736 | 0.0359 | 1.291E-06 | 23.3778749 | 0.0538 | 0.296 |
| rs114373846 | T | C | 0.4196 | 0.0905 | 3.568E-06 | 21.4915771 | 0.1434 | 0.9797 |
| rs11732981 | A | C | 0.0722 | 0.0156 | 3.793E-06 | 21.4150792 | 0.025 | 0.3539 |
| rs1333040 | T | C | 0.0747 | 0.0157 | 1.993E-06 | 22.632701 | 0.025 | 0.6239 |
| rs13412535 | A | G | -0.1186 | 0.0214 | 3.141E-08 | 30.7069242 | 0.0315 | 0.727601 |
| rs4684700 | T | C | -0.0747 | 0.0162 | 3.912E-06 | 21.2571805 | 0.0253 | 0.9779 |
| rs73273528 | T | C | 0.268 | 0.0553 | 0.00000125 | 23.4808499 | 0.0815 | 0.0644303 |
| rs76856708 | T | C | 0.336 | 0.0697 | 1.427E-06 | 23.2331173 | 0.1106 | 0.9568 |
| **IL-7** | | | | | | | | |
| rs117509142 | T | C | -0.3213 | 0.0684 | 2.599E-06 | 22.0524712 | 0.0702 | 0.3889 |
| rs11757972 | T | C | 0.121 | 0.0257 | 2.529E-06 | 22.1540183 | 0.0253 | 0.6053 |
| rs1374279 | A | T | 0.1625 | 0.0347 | 2.792E-06 | 21.9177413 | 0.0355 | 0.3323 |
| rs142397827 | A | C | 0.4592 | 0.0994 | 3.822E-06 | 21.329418 | 0.0893 | 0.9797 |
| rs17091524 | T | C | 0.5092 | 0.1015 | 5.244E-07 | 25.1531697 | 0.0965 | 0.407 |
| rs2006957 | T | C | 0.2557 | 0.0262 | 1.434E-22 | 95.1934111 | 0.0266 | 0.6863 |
| rs218238 | A | T | 0.1319 | 0.0284 | 3.277E-06 | 21.5576257 | 0.0299 | 0.9593 |
| rs28793375 | T | C | 0.1644 | 0.036 | 4.866E-06 | 20.8423479 | 0.037 | 0.8501 |
| rs62006410 | T | C | -0.1492 | 0.0302 | 7.588E-07 | 24.3933685 | 0.0293 | 0.1086 |
| rs7155170 | A | T | -0.1236 | 0.027 | 4.787E-06 | 20.9438939 | 0.0278 | 0.53 |
| rs77318030 | T | C | -0.2966 | 0.0631 | 2.639E-06 | 22.0816535 | 0.0622 | 0.609399 |
| rs77981494 | T | C | -0.5201 | 0.1055 | 8.225E-07 | 24.289401 | 0.0903 | 0.3714 |
| **IL-8** | | | | | | | | |
| rs113487695 | A | C | -0.6129 | 0.1292 | 2.092E-06 | 22.49109 | 0.1358 | 0.6705 |
| rs116726256 | T | C | -0.2247 | 0.0489 | 4.261E-06 | 21.1030251 | 0.0539 | 0.611 |
| rs12075 | A | G | 0.1148 | 0.0235 | 9.969E-07 | 23.8508763 | 0.0251 | 0.7618 |
| rs12912642 | A | G | 0.1168 | 0.0251 | 3.212E-06 | 21.6418574 | 0.0263 | 0.9183 |
| rs183628733 | T | C | 0.6547 | 0.1417 | 3.821E-06 | 21.3354236 | 0.12 | 0.3272 |
| rs2673604 | A | C | -0.118 | 0.0254 | 3.289E-06 | 21.5701353 | 0.0269 | 0.726501 |
| rs3786107 | A | G | 0.2463 | 0.0517 | 1.935E-06 | 22.6831845 | 0.0483 | 0.729699 |
| rs75840288 | A | C | 0.5125 | 0.1121 | 4.846E-06 | 20.8897288 | 0.1074 | 0.3113 |
| **IL-9** | | | | | | | | |
| rs117807175 | C | G | -0.5225 | 0.1106 | 2.327E-06 | 22.3062103 | 0.1102 | 0.9667 |
| rs1259728 | A | G | -0.2381 | 0.0507 | 2.599E-06 | 22.0427781 | 0.0535 | 0.3232 |
| rs3736858 | C | G | -0.1351 | 0.0291 | 3.373E-06 | 21.5421069 | 0.0312 | 0.4176 |
| rs41294750 | T | C | 0.3442 | 0.0736 | 2.916E-06 | 21.8589632 | 0.0746 | 0.2596 |
| rs4880409 | T | C | -0.3552 | 0.0716 | 6.952E-07 | 24.5970671 | 0.1234 | 0.229 |
| rs73443903 | A | C | 0.2162 | 0.046 | 2.569E-06 | 22.0779717 | 0.0494 | 0.1792 |
| **IL-10** | | | | | | | | |
| rs10457128 | A | G | -0.0854 | 0.0172 | 6.956E-07 | 24.6459963 | 0.0261 | 0.741199 |
| rs10493718 | A | C | -0.1081 | 0.0222 | 1.068E-06 | 23.7046186 | 0.0342 | 0.745399 |
| rs13412535 | A | G | -0.1347 | 0.0224 | 1.798E-09 | 36.1515304 | 0.0315 | 0.727601 |
| rs1530455 | T | C | 0.082 | 0.0174 | 2.527E-06 | 22.2032598 | 0.0262 | 0.9887 |
| rs2086656 | T | C | -0.08 | 0.017 | 2.589E-06 | 22.1395946 | 0.0263 | 0.683501 |
| rs3002131 | C | G | 0.1191 | 0.026 | 4.592E-06 | 20.9780134 | 0.0365 | 0.2986 |
| rs3025021 | T | C | 0.0913 | 0.0194 | 2.609E-06 | 22.1424477 | 0.0267 | 0.4341 |
| rs383684 | A | G | 0.092 | 0.0197 | 3.168E-06 | 21.8037269 | 0.0438 | 0.3955 |
| rs4741748 | A | G | -0.0788 | 0.0169 | 3.202E-06 | 21.735346 | 0.0257 | 0.262 |
| rs6054847 | T | C | 0.0971 | 0.0207 | 2.752E-06 | 21.9981065 | 0.0318 | 0.9584 |
| rs6680918 | T | C | -0.1202 | 0.025 | 1.591E-06 | 23.1108783 | 0.0383 | 0.5097 |
| rs7088799 | T | G | -0.0815 | 0.0166 | 9.352E-07 | 24.0983093 | 0.0257 | 0.2846 |
| rs73192842 | A | G | 0.0949 | 0.0206 | 4.031E-06 | 21.2170752 | 0.0321 | 0.9608 |
| rs7747448 | A | G | -0.1061 | 0.0189 | 1.998E-08 | 31.5061032 | 0.0286 | 0.9998 |
| rs9472173 | T | C | -0.2004 | 0.0174 | 1.256E-30 | 132.612502 | 0.0258 | 0.0138 |
| **IL-12p70** |  |  |  |  |  |  |  |  |
| rs113600793 | A | C | 0.1832 | 0.0359 | 3.351E-07 | 26.0349984 | 0.0538 | 0.296 |
| rs12969892 | T | C | 0.1227 | 0.0267 | 4.194E-06 | 21.1135916 | 0.0414 | 0.1849 |
| rs2123852 | T | C | 0.0942 | 0.0204 | 0.00000373 | 21.317535 | 0.0315 | 0.1279 |
| rs273702 | A | G | -0.127 | 0.027 | 2.522E-06 | 22.1195062 | 0.0419 | 0.7854 |
| rs282258 | T | C | 0.0726 | 0.0156 | 3.282E-06 | 21.6530739 | 0.0252 | 0.3077 |
| rs34322762 | T | C | 0.0953 | 0.0199 | 1.708E-06 | 22.9267052 | 0.0264 | 0.1138 |
| rs34826779 | T | G | -0.0884 | 0.019 | 3.327E-06 | 21.6417733 | 0.0304 | 0.731799 |
| rs41282644 | A | G | 0.1401 | 0.0303 | 3.737E-06 | 21.3740356 | 0.0467 | 0.9045 |
| rs4530855 | T | G | 0.0861 | 0.0184 | 2.872E-06 | 21.8910287 | 0.0277 | 0.6285 |
| rs4741748 | A | G | -0.0799 | 0.0163 | 9.162E-07 | 24.0222601 | 0.0257 | 0.262 |
| rs6532374 | T | C | -0.1033 | 0.0226 | 4.613E-06 | 20.8871545 | 0.0356 | 0.559399 |
| rs7754905 | A | G | -0.1005 | 0.019 | 1.14E-07 | 27.9718014 | 0.0299 | 0.4878 |
| rs782111 | A | C | -0.0765 | 0.0156 | 9.247E-07 | 24.0419222 | 0.025 | 0.6532 |
| rs865585 | A | C | -0.1654 | 0.0237 | 2.732E-12 | 48.69337 | 0.0354 | 0.7201 |
| rs9381249 | T | C | -0.1788 | 0.0367 | 1.125E-06 | 23.730037 | 0.0622 | 0.2709 |
| **IL-13** | | | | | | | | |
| rs10995604 | A | G | -0.1571 | 0.0343 | 4.482E-06 | 20.966335 | 0.0349 | 0.5236 |
| rs117795020 | A | G | -0.3584 | 0.0716 | 5.479E-07 | 25.0419617 | 0.0739 | 0.1736 |
| rs12623722 | A | G | -0.1189 | 0.0257 | 3.614E-06 | 21.3922198 | 0.0273 | 0.8282 |
| rs138854806 | A | G | -0.4204 | 0.0839 | 5.449E-07 | 25.0934216 | 0.0753 | 0.3311 |
| rs139083458 | T | C | 0.9995 | 0.211 | 2.165E-06 | 22.4263748 | 0.21 | 0.9261 |
| rs147747784 | C | G | 0.369 | 0.0765 | 1.435E-06 | 23.2534958 | 0.0584 | 0.1799 |
| rs150836197 | T | C | 0.3283 | 0.0713 | 4.136E-06 | 21.1895189 | 0.0644 | 0.741401 |
| rs27949 | T | C | -0.1144 | 0.025 | 4.827E-06 | 20.9281299 | 0.0268 | 0.4848 |
| rs28442067 | A | G | -0.1379 | 0.0286 | 1.413E-06 | 23.2356516 | 0.0306 | 0.6506 |
| rs7073807 | T | C | 0.1618 | 0.0354 | 0.00000477 | 20.8789618 | 0.037 | 0.6528 |
| rs75383097 | C | G | -0.5369 | 0.116 | 3.702E-06 | 21.4106188 | 0.1009 | 0.7824 |
| rs76339001 | A | T | -0.4375 | 0.0886 | 7.915E-07 | 24.3695248 | 0.0758 | 0.9948 |
| rs76975337 | T | C | -0.1211 | 0.0265 | 4.921E-06 | 20.8715608 | 0.0284 | 0.7133 |
| rs77955971 | A | C | 0.4408 | 0.0868 | 3.756E-07 | 25.775204 | 0.0696 | 0.5197 |
| **IL-16** | | | | | | | | |
| rs117217798 | T | C | -0.2064 | 0.044 | 2.772E-06 | 21.992129 | 0.0449 | 0.35 |
| rs12577604 | T | C | 0.4335 | 0.0941 | 4.083E-06 | 21.2105631 | 0.0985 | 0.3859 |
| rs142034902 | A | G | -0.4367 | 0.0925 | 2.327E-06 | 22.2759462 | 0.0993 | 0.1382 |
| rs142332135 | A | G | -0.7646 | 0.1082 | 1.581E-12 | 49.9076715 | 0.0957 | 0.3791 |
| rs144691581 | A | G | 0.4929 | 0.0958 | 2.668E-07 | 26.4569551 | 0.0865 | 0.5086 |
| rs35834666 | T | C | -0.1729 | 0.0348 | 6.57E-07 | 24.6708857 | 0.0357 | 0.4051 |
| rs4778640 | A | G | 0.7189 | 0.0983 | 2.552E-13 | 53.4543648 | 0.0987 | 0.626 |
| rs4976691 | C | G | 0.1254 | 0.026 | 1.473E-06 | 23.2488577 | 0.0269 | 0.4977 |
| rs7097884 | T | C | -0.1193 | 0.0243 | 8.809E-07 | 24.0891559 | 0.0253 | 0.1245 |
| rs78042619 | A | G | 0.55 | 0.1158 | 2.023E-06 | 22.5455847 | 0.1109 | 0.425 |
| **IL-17** | | | | | | | | |
| rs11985957 | A | G | 0.1511 | 0.0329 | 4.363E-06 | 21.0875351 | 0.0546 | 0.4943 |
| rs12735700 | T | G | -0.0943 | 0.0206 | 0.0000045 | 20.9496914 | 0.0307 | 0.8783 |
| rs145006174 | C | G | -0.2266 | 0.0473 | 1.645E-06 | 22.9449024 | 0.0706 | 0.3038 |
| rs17282552 | T | C | -0.2026 | 0.0403 | 4.876E-07 | 25.2672208 | 0.057 | 0.3957 |
| rs3792369 | A | G | 0.0941 | 0.0166 | 1.46E-08 | 32.1256376 | 0.0257 | 0.723199 |
| rs61990749 | C | G | 0.1124 | 0.0226 | 6.569E-07 | 24.728879 | 0.0352 | 0.4691 |
| rs78296352 | T | G | 0.2949 | 0.0645 | 4.809E-06 | 20.8986774 | 0.1092 | 0.912 |
| rs9519328 | A | G | 0.5256 | 0.1101 | 0.00000179 | 22.7767495 | 0.083 | 0.7268 |
| **IL-18** | | | | | | | | |
| rs10409850 | A | G | 0.1791 | 0.0347 | 2.44E-07 | 26.6253795 | 0.0368 | 0.1228 |
| rs11214093 | T | C | 0.1143 | 0.0238 | 1.544E-06 | 23.0516561 | 0.0252 | 0.3679 |
| rs117266781 | T | C | 0.7051 | 0.1436 | 9.176E-07 | 24.0966203 | 0.1333 | 0.8806 |
| rs117371668 | T | G | 0.3712 | 0.0799 | 3.357E-06 | 21.571779 | 0.0854 | 0.773101 |
| rs139468359 | T | C | 0.5101 | 0.1088 | 2.737E-06 | 21.9693112 | 0.1145 | 0.707301 |
| rs1979967 | T | C | 0.14 | 0.0285 | 8.719E-07 | 24.1173694 | 0.0306 | 0.742499 |
| rs4952239 | A | T | -0.1156 | 0.0242 | 1.809E-06 | 22.8059686 | 0.0259 | 0.2657 |
| rs58701153 | A | T | -0.1265 | 0.0242 | 1.807E-07 | 27.3095098 | 0.0263 | 0.0244 |
| rs62312914 | T | C | -0.1265 | 0.025 | 4.215E-07 | 25.5896661 | 0.0259 | 0.5196 |
| rs764078 | A | T | 0.1283 | 0.0278 | 4.075E-06 | 21.2876296 | 0.0296 | 0.8353 |
| rs77187209 | T | C | -0.4859 | 0.1041 | 3.082E-06 | 21.7748877 | 0.108 | 0.4086 |
| rs78623212 | T | C | 0.8322 | 0.1676 | 6.82E-07 | 24.6417178 | 0.1387 | 0.0120901 |
| rs78716465 | A | G | 0.3173 | 0.0679 | 2.981E-06 | 21.8254989 | 0.0669 | 0.8612 |
| **IP-10** | | | | | | | | |
| rs113183470 | A | T | -0.2414 | 0.0524 | 4.147E-06 | 21.2118552 | 0.0535 | 0.2096 |
| rs12714300 | A | T | -0.1573 | 0.0338 | 3.297E-06 | 21.6466523 | 0.0356 | 0.501 |
| rs143799975 | A | G | -0.7551 | 0.1638 | 4.012E-06 | 21.2396738 | 0.1475 | 0.5857 |
| rs34383175 | T | C | -0.3196 | 0.0653 | 9.904E-07 | 23.9416388 | 0.0688 | 0.780899 |
| rs397816 | T | C | 0.1211 | 0.0248 | 1.026E-06 | 23.8315133 | 0.0261 | 0.506499 |
| rs4859940 | C | G | -0.1204 | 0.0258 | 3.228E-06 | 21.7660819 | 0.0275 | 0.5191 |
| rs4862110 | T | C | -0.1453 | 0.0318 | 4.909E-06 | 20.8662153 | 0.0291 | 0.2232 |
| rs75970138 | A | G | -0.4845 | 0.1037 | 2.994E-06 | 21.8170861 | 0.1102 | 0.0882897 |
| rs7645625 | T | G | -0.1116 | 0.0236 | 2.192E-06 | 22.3496682 | 0.0256 | 0.7443 |
| rs78077394 | T | C | -0.3486 | 0.0701 | 6.474E-07 | 24.716412 | 0.0704 | 0.3257 |
| rs79848609 | A | C | 0.2514 | 0.0535 | 2.637E-06 | 22.069357 | 0.0581 | 0.00611294 |
| rs8112618 | A | G | 0.1388 | 0.0297 | 3.047E-06 | 21.8289442 | 0.0312 | 0.0536599 |
| **M-CSF** | | | | | | | | |
| rs116274860 | T | G | 0.8262 | 0.1739 | 2.029E-06 | 22.553989 | 0.1116 | 0.8216 |
| rs116887628 | A | G | -0.2741 | 0.0598 | 4.626E-06 | 20.9926783 | 0.0492 | 0.4604 |
| rs117867915 | T | C | 0.5224 | 0.1096 | 1.874E-06 | 22.700602 | 0.0911 | 0.6033 |
| rs11963606 | C | G | -0.5353 | 0.117 | 4.731E-06 | 20.9158207 | 0.0972 | 0.187 |
| rs12962919 | T | C | 0.3025 | 0.0659 | 4.394E-06 | 21.0538767 | 0.0497 | 0.5818 |
| rs139457375 | A | C | -0.4047 | 0.0854 | 2.142E-06 | 22.4389646 | 0.0723 | 0.579001 |
| rs147378920 | A | G | -0.6064 | 0.1318 | 4.177E-06 | 21.1514289 | 0.0892 | 0.675601 |
| rs34089869 | T | C | 0.2194 | 0.0462 | 2.078E-06 | 22.5341636 | 0.0403 | 0.9107 |
| rs62294910 | A | G | 0.3472 | 0.0687 | 4.378E-07 | 25.5210246 | 0.0517 | 0.1488 |
| rs72723242 | T | G | -0.4969 | 0.1083 | 4.434E-06 | 21.0345501 | 0.0996 | 0.265 |
| rs9387100 | T | C | -0.135 | 0.029 | 3.341E-06 | 21.6532798 | 0.0253 | 0.2125 |
| rs9626985 | T | C | 0.2277 | 0.0496 | 4.482E-06 | 21.0578901 | 0.0424 | 0.2769 |
| **MCP-1** | | | | | | | | |
| rs111995966 | T | G | 0.1428 | 0.0309 | 3.788E-06 | 21.3518377 | 0.0472 | 0.0537106 |
| rs11920996 | T | C | 0.1805 | 0.0376 | 1.604E-06 | 23.0395783 | 0.0594 | 0.7514 |
| rs12062235 | T | G | 0.1477 | 0.032 | 3.836E-06 | 21.2988834 | 0.0518 | 0.1157 |
| rs143815843 | A | G | -0.2049 | 0.0447 | 4.609E-06 | 21.0070308 | 0.0728 | 0.3673 |
| rs16837903 | A | G | -0.1104 | 0.0238 | 3.352E-06 | 21.5119627 | 0.0369 | 0.2802 |
| rs2201150 | T | C | 0.0916 | 0.016 | 1.044E-08 | 32.7677623 | 0.0259 | 0.9412 |
| rs2229593 | T | C | 0.2624 | 0.0405 | 9.246E-11 | 41.9675308 | 0.0637 | 0.9212 |
| rs56212190 | T | C | 0.1799 | 0.0372 | 1.318E-06 | 23.3814936 | 0.0589 | 0.2376 |
| rs62245103 | T | G | 0.2433 | 0.0416 | 4.989E-09 | 34.1974213 | 0.0613 | 0.2019 |
| rs7197349 | A | G | 0.0971 | 0.0206 | 2.399E-06 | 22.2126218 | 0.0316 | 0.4489 |
| rs72705803 | A | G | -0.2188 | 0.047 | 3.222E-06 | 21.6667974 | 0.079 | 0.249 |
| rs7978037 | A | T | 0.0746 | 0.016 | 3.043E-06 | 21.7336912 | 0.0258 | 0.2398 |
| rs79939301 | A | G | 0.1449 | 0.0255 | 1.356E-08 | 32.281389 | 0.0397 | 0.8869 |
| rs856100 | A | G | 0.0899 | 0.0191 | 2.545E-06 | 22.1487108 | 0.0305 | 0.3169 |
| rs862990 | T | C | -0.089 | 0.0183 | 1.153E-06 | 23.6468685 | 0.0287 | 0.710901 |
| rs9317045 | A | C | 0.1157 | 0.0235 | 8.425E-07 | 24.2340944 | 0.0355 | 0.4252 |
| **MCP-3** | | | | | | | | |
| rs10892381 | T | C | 0.2432 | 0.0473 | 2.693E-07 | 26.3884447 | 0.0267 | 0.5586 |
| rs117286643 | A | G | 0.6934 | 0.1474 | 2.542E-06 | 22.0892842 | 0.0867 | 0.356 |
| rs28394764 | A | T | 0.597 | 0.1282 | 3.194E-06 | 21.646194 | 0.0715 | 0.4844 |
| rs3129806 | T | C | -0.1975 | 0.0433 | 4.978E-06 | 20.766693 | 0.0254 | 0.6383 |
| rs6993671 | T | C | 0.2041 | 0.0443 | 4.061E-06 | 21.1878794 | 0.0257 | 0.5101 |
| rs7275485 | T | C | -0.2218 | 0.0481 | 3.986E-06 | 21.2247149 | 0.0281 | 0.3755 |
| **MIF** | | | | | | | | |
| rs1007888 | T | C | -0.1275 | 0.0245 | 1.915E-07 | 27.0671345 | 0.0257 | 0.9498 |
| rs113218956 | A | G | -0.8789 | 0.1876 | 2.815E-06 | 21.9365002 | 0.1931 | 0.693499 |
| rs11551183 | C | G | 0.3666 | 0.0795 | 3.999E-06 | 21.2522416 | 0.0858 | 0.3571 |
| rs12594190 | A | G | 0.1321 | 0.0266 | 6.85E-07 | 24.6488375 | 0.0274 | 0.6024 |
| rs141009259 | T | C | -0.6194 | 0.1285 | 1.444E-06 | 23.2214977 | 0.1188 | 0.0774194 |
| rs2294689 | C | G | -0.1338 | 0.0287 | 3.043E-06 | 21.7221353 | 0.1026 | 0.435 |
| rs35792361 | A | G | -0.2586 | 0.0527 | 9.003E-07 | 24.065223 | 0.0538 | 0.1296 |
| rs35890933 | T | G | 0.1676 | 0.0365 | 4.458E-06 | 21.0725154 | 0.0356 | 0.4927 |
| rs3814097 | A | G | -0.1163 | 0.0251 | 3.548E-06 | 21.456855 | 0.0255 | 0.651 |
| rs78098071 | T | C | -0.4583 | 0.0915 | 5.509E-07 | 25.0733063 | 0.0973 | 0.712801 |
| **MIG** |  |  |  |  |  |  |  |  |
| rs10266753 | T | C | -0.2016 | 0.0397 | 3.769E-07 | 25.7731047 | 0.0449 | 0.478 |
| rs111607343 | A | G | -0.5235 | 0.1119 | 2.928E-06 | 21.8745887 | 0.1064 | 0.5888 |
| rs11177248 | A | G | 0.3157 | 0.0667 | 2.222E-06 | 22.3905207 | 0.0638 | 0.9073 |
| rs113302091 | T | C | 0.2537 | 0.0553 | 4.402E-06 | 21.0357194 | 0.0603 | 0.9159 |
| rs13143163 | C | G | 0.2735 | 0.0582 | 2.622E-06 | 22.0716799 | 0.0542 | 0.0338197 |
| rs139010077 | T | C | 0.4337 | 0.0943 | 4.193E-06 | 21.1408359 | 0.099 | 0.4156 |
| rs191555775 | A | T | 0.2279 | 0.0412 | 3.278E-08 | 30.5816501 | 0.0414 | 0.4901 |
| rs192433162 | A | G | -0.8045 | 0.1676 | 1.594E-06 | 23.0287712 | 0.1639 | 0.5716 |
| rs3733233 | T | C | 0.1223 | 0.025 | 1.049E-06 | 23.9188113 | 0.0263 | 0.622801 |
| rs62562991 | A | G | 0.6239 | 0.1259 | 7.237E-07 | 24.5439924 | 0.1056 | 0.184 |
| rs6679677 | A | C | 0.1628 | 0.0327 | 6.514E-07 | 24.7730793 | 0.0355 | 0.7565 |
| rs8127917 | T | G | 0.2382 | 0.0492 | 1.278E-06 | 23.4271795 | 0.0523 | 0.3091 |
| rs816960 | T | C | -0.1179 | 0.0242 | 0.00000115 | 23.7226704 | 0.0261 | 0.1477 |
| **MIP-1A** | | | | | | | | |
| rs117506943 | T | C | 0.0108 | 0.0682 | 4.484E-06 | 21.0242617 | 0.0628 | 0.8634 |
| rs12159394 | A | G | 0.0008 | 0.0366 | 3.112E-06 | 21.7655465 | 0.0393 | 0.9847 |
| rs57786342 | A | G | 0.0149 | 0.0283 | 8.909E-07 | 24.1108624 | 0.033 | 0.6507 |
| rs6956239 | T | C | -0.0088 | 0.026 | 4.583E-06 | 20.9364595 | 0.0276 | 0.7488 |
| **MIP-1B** | | | | | | | | |
| rs111721971 | T | G | -0.227 | 0.047 | 1.387E-06 | 23.321215 | 0.0689 | 0.3631 |
| rs116237296 | A | G | 0.5284 | 0.1115 | 2.153E-06 | 22.4528284 | 0.176 | 0.6669 |
| rs11651720 | T | C | -0.1189 | 0.0219 | 5.688E-08 | 29.4693566 | 0.0349 | 0.579399 |
| rs11716293 | C | G | 0.0986 | 0.0189 | 1.689E-07 | 27.2098029 | 0.0293 | 0.1779 |
| rs117657747 | A | G | 0.2089 | 0.0453 | 4.013E-06 | 21.2589533 | 0.0545 | 0.8592 |
| rs145526037 | T | G | -0.1863 | 0.0406 | 4.473E-06 | 21.0508102 | 0.0651 | 0.5366 |
| rs17138331 | A | G | -0.1434 | 0.0295 | 1.125E-06 | 23.623783 | 0.0451 | 0.583201 |
| rs17661219 | C | G | 0.0872 | 0.0172 | 3.888E-07 | 25.6963388 | 0.0267 | 0.666 |
| rs2314809 | T | C | -0.0735 | 0.0157 | 2.904E-06 | 21.9114212 | 0.0251 | 0.3167 |
| rs2742396 | T | C | 0.1071 | 0.0168 | 1.832E-10 | 40.6308167 | 0.0256 | 0.4604 |
| rs281728 | A | C | -0.079 | 0.0171 | 3.891E-06 | 21.3381683 | 0.0272 | 0.6814 |
| rs28393318 | A | G | -0.1076 | 0.0235 | 4.616E-06 | 20.9596484 | 0.0377 | 0.616799 |
| rs57893487 | C | G | -0.1075 | 0.0214 | 4.916E-07 | 25.2281007 | 0.031 | 0.0727495 |
| rs6802288 | A | G | -0.1623 | 0.0173 | 6.574E-21 | 87.9913554 | 0.0281 | 0.770699 |
| rs6806860 | A | C | -0.1008 | 0.0188 | 8.575E-08 | 28.7409116 | 0.03 | 0.1896 |
| rs6908843 | A | G | 0.0997 | 0.0209 | 1.779E-06 | 22.7506033 | 0.0334 | 0.3244 |
| rs72791296 | T | C | 0.2364 | 0.0466 | 3.968E-07 | 25.7287262 | 0.0686 | 0.441 |
| rs72799710 | T | C | -0.1037 | 0.0217 | 1.792E-06 | 22.8314355 | 0.0346 | 0.7415 |
| rs74979864 | A | T | -0.3184 | 0.0613 | 2.025E-07 | 26.9724467 | 0.096 | 0.7962 |
| rs76582507 | A | G | 0.3259 | 0.0676 | 1.421E-06 | 23.2364888 | 0.1446 | 0.7985 |
| rs772112 | A | T | -0.142 | 0.0216 | 5.007E-11 | 43.2080195 | 0.0322 | 0.3271 |
| rs9916627 | T | C | -0.096 | 0.0198 | 1.244E-06 | 23.5021319 | 0.032 | 0.2137 |
| **PDGF-BB** | | | | | | | | |
| rs10512952 | T | C | -0.2816 | 0.0587 | 1.636E-06 | 23.0083196 | 0.093 | 0.1861 |
| rs116154010 | T | C | 0.3225 | 0.0662 | 1.113E-06 | 23.726805 | 0.1118 | 0.0139601 |
| rs11766649 | A | G | 0.0902 | 0.0196 | 3.964E-06 | 21.173699 | 0.0308 | 0.4178 |
| rs12289510 | A | G | -0.0772 | 0.0158 | 1.001E-06 | 23.868011 | 0.0251 | 0.1881 |
| rs12615784 | T | C | -0.1003 | 0.0193 | 1.986E-07 | 27.0011991 | 0.0308 | 0.5963 |
| rs147862316 | T | C | 0.2279 | 0.0411 | 2.987E-08 | 30.7397838 | 0.0691 | 0.5302 |
| rs2643354 | A | G | 0.1251 | 0.0261 | 1.633E-06 | 22.9683294 | 0.0426 | 0.315 |
| rs35859699 | A | G | -0.3854 | 0.0838 | 4.223E-06 | 21.146144 | 0.1171 | 0.6856 |
| rs62191444 | T | G | -0.112 | 0.0239 | 2.678E-06 | 21.9551317 | 0.035 | 0.9842 |
| rs6756793 | T | C | 0.0876 | 0.0157 | 2.684E-08 | 31.1246668 | 0.0253 | 0.1679 |
| rs6910518 | T | G | 0.0806 | 0.0162 | 6.005E-07 | 24.7477578 | 0.0258 | 0.8936 |
| rs72972467 | C | G | -0.1616 | 0.0328 | 8.286E-07 | 24.2678235 | 0.0498 | 0.1245 |
| rs73162807 | A | C | -0.2313 | 0.0499 | 3.548E-06 | 21.4805787 | 0.0802 | 0.0875004 |
| rs9924851 | C | G | 0.0767 | 0.0163 | 2.702E-06 | 22.1366206 | 0.0261 | 0.4297 |
| **RANTES** | | | | | | | | |
| rs10505135 | T | C | 0.035 | 0.0252 | 1.899E-07 | 27.2144071 | 0.0261 | 0.1804 |
| rs118096511 | T | C | -0.0676 | 0.0709 | 1.965E-06 | 22.6332093 | 0.0744 | 0.363 |
| rs11873385 | A | G | 0.0096 | 0.0552 | 2.927E-06 | 21.8666762 | 0.0555 | 0.8633 |
| rs148526102 | T | C | -0.002 | 0.083 | 4.793E-06 | 20.9267695 | 0.0866 | 0.9818 |
| rs2731672 | T | C | -0.0476 | 0.0272 | 4.827E-06 | 20.8378946 | 0.0288 | 0.0984396 |
| rs4795087 | C | G | -0.0065 | 0.0312 | 1.629E-06 | 22.9160869 | 0.0325 | 0.8414 |
| rs62438851 | A | G | -0.024 | 0.0413 | 4.009E-06 | 21.241356 | 0.0411 | 0.559899 |
| rs7170339 | C | G | 0.0645 | 0.0904 | 2.187E-06 | 22.434053 | 0.09 | 0.4739 |
| rs72793342 | A | G | -0.0251 | 0.0307 | 9.08E-07 | 24.0184348 | 0.0309 | 0.4175 |
| rs78050316 | A | C | -0.0279 | 0.0859 | 9.946E-07 | 23.9152113 | 0.0948 | 0.7689 |
| **SCF** | | | | | | | | |
| rs10800449 | A | C | 0.0851 | 0.0179 | 1.962E-06 | 22.5968979 | 0.0277 | 0.9676 |
| rs11244035 | T | C | -0.1296 | 0.0279 | 3.501E-06 | 21.5723452 | 0.045 | 0.0322901 |
| rs113127926 | A | C | 0.1974 | 0.0418 | 2.337E-06 | 22.2965412 | 0.0642 | 0.8279 |
| rs117721699 | C | G | -0.2392 | 0.0484 | 7.514E-07 | 24.4189729 | 0.0773 | 0.7605 |
| rs12345108 | T | C | -0.0772 | 0.0167 | 3.731E-06 | 21.3647307 | 0.0262 | 0.6376 |
| rs13412535 | A | G | -0.1065 | 0.0213 | 5.586E-07 | 24.9940005 | 0.0315 | 0.727601 |
| rs138538809 | T | C | -0.5788 | 0.1139 | 3.757E-07 | 25.8169755 | 0.1684 | 0.7003 |
| rs72678285 | A | T | 0.1062 | 0.0231 | 0.00000443 | 21.1310384 | 0.0342 | 0.1785 |
| rs78666213 | T | G | -0.2845 | 0.0574 | 7.152E-07 | 24.5604614 | 0.0847 | 0.2331 |
| rs8045376 | A | G | -0.3126 | 0.068 | 4.268E-06 | 21.1278783 | 0.1092 | 0.7115 |
| **SCGF-B** | | | | | | | | |
| rs11111869 | A | G | 0.1621 | 0.0311 | 1.861E-07 | 27.1526211 | 0.0337 | 0.8953 |
| rs112346514 | T | C | -0.3261 | 0.0703 | 3.543E-06 | 21.5058918 | 0.0645 | 0.8068 |
| rs1149926 | T | C | -0.3458 | 0.0749 | 3.917E-06 | 21.3035927 | 0.0805 | 0.1454 |
| rs118003677 | T | C | -0.3654 | 0.0786 | 3.348E-06 | 21.6002363 | 0.0848 | 0.0687797 |
| rs12118918 | A | G | -0.1631 | 0.035 | 3.208E-06 | 21.7039281 | 0.0355 | 0.3499 |
| rs12480722 | T | C | 0.1654 | 0.0353 | 2.812E-06 | 21.9426011 | 0.0372 | 0.5163 |
| rs13287050 | A | T | -0.121 | 0.0263 | 4.118E-06 | 21.1556197 | 0.0279 | 0.2025 |
| rs13866 | T | C | -0.1647 | 0.028 | 3.773E-09 | 34.5810077 | 0.0287 | 0.3178 |
| rs139413256 | A | G | -0.5174 | 0.1076 | 1.532E-06 | 23.109727 | 0.0971 | 0.8075 |
| rs143829871 | T | C | -0.1866 | 0.0399 | 2.852E-06 | 21.8596898 | 0.0433 | 0.8139 |
| rs144724875 | T | C | 0.5381 | 0.0829 | 8.645E-11 | 42.1098245 | 0.0764 | 0.3326 |
| rs149009264 | A | G | 0.4551 | 0.0985 | 3.793E-06 | 21.3357404 | 0.1013 | 0.321 |
| rs150733161 | T | C | -0.5255 | 0.112 | 2.687E-06 | 22.0026963 | 0.1081 | 0.4726 |
| rs151194174 | A | G | 0.4536 | 0.0941 | 1.454E-06 | 23.2238038 | 0.0667 | 0.4599 |
| rs264157 | A | G | 0.1079 | 0.0233 | 3.685E-06 | 21.4337201 | 0.025 | 0.3306 |
| rs34911860 | A | G | -0.3674 | 0.0787 | 3.002E-06 | 21.7818793 | 0.0975 | 0.9854 |
| rs3817303 | T | G | 0.1362 | 0.0294 | 3.602E-06 | 21.4499391 | 0.0318 | 0.223 |
| rs4737731 | T | C | 0.1146 | 0.0251 | 4.871E-06 | 20.8347503 | 0.0276 | 0.1895 |
| rs4976691 | C | G | -0.1484 | 0.0253 | 4.438E-09 | 34.3869192 | 0.0269 | 0.4977 |
| rs77954165 | T | C | 0.2631 | 0.0562 | 2.867E-06 | 21.9046124 | 0.058 | 0.181 |
| rs78217154 | T | C | 0.3942 | 0.0861 | 4.722E-06 | 20.9504543 | 0.0892 | 0.02427 |
| **SDF-1A** | | | | | | | | |
| rs10474392 | A | G | 0.0934 | 0.0177 | 1.376E-07 | 27.8378865 | 0.0288 | 0.9958 |
| rs10516368 | A | C | -0.4268 | 0.0883 | 1.356E-06 | 23.3569674 | 0.1377 | 0.4752 |
| rs12141941 | T | C | -0.0881 | 0.0186 | 2.263E-06 | 22.4292612 | 0.0279 | 0.9609 |
| rs149893336 | A | G | -0.494 | 0.1082 | 0.00000493 | 20.8395539 | 0.1163 | 0.2252 |
| rs1600396 | A | G | -0.0933 | 0.0204 | 4.939E-06 | 20.9118278 | 0.0316 | 0.9337 |
| rs62194946 | T | G | -0.0849 | 0.0185 | 4.552E-06 | 21.0552772 | 0.0287 | 0.8592 |
| rs6586903 | T | C | -0.1264 | 0.0268 | 2.421E-06 | 22.2389153 | 0.0397 | 0.1182 |
| rs76766406 | A | G | 0.4642 | 0.1012 | 4.489E-06 | 21.0347952 | 0.1483 | 0.7877 |
| rs78883416 | C | G | -0.0871 | 0.0182 | 1.755E-06 | 22.8972104 | 0.027 | 0.5525 |
| **TNF-A** | | | | | | | | |
| rs10767536 | A | G | 0.118 | 0.0253 | 3.146E-06 | 21.7407357 | 0.0264 | 0.0561604 |
| rs115018697 | C | G | -0.9542 | 0.197 | 1.273E-06 | 23.4475589 | 0.1885 | 0.677699 |
| rs116736594 | T | C | 0.3407 | 0.0702 | 1.223E-06 | 23.5408048 | 0.067 | 0.6891 |
| rs79105320 | A | G | 0.5573 | 0.1177 | 2.207E-06 | 22.4066239 | 0.1208 | 0.1706 |
| **TNF-B** | | | | | | | | |
| rs10925040 | T | C | 0.1738 | 0.0372 | 2.929E-06 | 21.8001085 | 0.0262 | 0.1127 |
| rs2420873 | T | G | 0.1673 | 0.0365 | 4.513E-06 | 20.9822059 | 0.0257 | 0.1158 |
| rs62284710 | A | G | 0.3702 | 0.0782 | 2.183E-06 | 22.3822794 | 0.0523 | 0.3184 |
| rs75240021 | C | G | 0.3713 | 0.0772 | 1.489E-06 | 23.1025697 | 0.0482 | 0.3469 |
| rs76225863 | A | G | 0.7534 | 0.1217 | 5.982E-10 | 38.2749883 | 0.0799 | 0.8893 |
| **TRAIL** | | | | | | | | |
| rs113057689 | A | G | -0.2625 | 0.0489 | 7.972E-08 | 28.8094751 | 0.0605 | 0.8344 |
| rs11875481 | T | C | -0.0969 | 0.0211 | 4.601E-06 | 21.0851692 | 0.0337 | 0.9657 |
| rs12458564 | A | T | -0.1002 | 0.0175 | 1.1E-08 | 32.7758372 | 0.0278 | 0.2291 |
| rs13115587 | A | C | 0.101 | 0.0217 | 3.244E-06 | 21.657969 | 0.0359 | 0.7331 |
| rs13278062 | T | G | 0.08 | 0.0157 | 3.326E-07 | 25.9582324 | 0.0251 | 0.00025 |
| rs139958028 | A | G | 0.1803 | 0.0395 | 4.992E-06 | 20.830117 | 0.0598 | 0.4134 |
| rs183815186 | A | T | -0.3499 | 0.0602 | 6.341E-09 | 33.7745328 | 0.0929 | 0.0181999 |
| rs550057 | T | C | -0.0783 | 0.0169 | 3.707E-06 | 21.4607336 | 0.0272 | 0.1326 |
| rs558572 | T | C | 0.1351 | 0.0265 | 3.419E-07 | 25.9844422 | 0.0419 | 0.3612 |
| rs57396456 | T | C | -0.5641 | 0.0516 | 7.71E-28 | 119.48331 | 0.0834 | 0.8476 |
| rs616114 | T | C | -0.1033 | 0.0162 | 1.715E-10 | 40.6504223 | 0.0258 | 0.1551 |
| rs62093482 | T | C | 0.9827 | 0.0529 | 6.123E-77 | 345.004703 | 0.0867 | 0.5401 |
| rs747324 | T | C | -0.0826 | 0.0178 | 3.338E-06 | 21.528538 | 0.0279 | 0.0928197 |
| rs75928541 | A | G | 0.2784 | 0.0591 | 2.442E-06 | 22.1849241 | 0.0837 | 0.6009 |
| rs7599203 | T | C | 0.0918 | 0.02 | 4.333E-06 | 21.0629802 | 0.0322 | 0.7784 |
| rs78682108 | A | G | -0.2383 | 0.0394 | 1.435E-09 | 36.572116 | 0.0573 | 0.3918 |
| **VEGF** | | | | | | | | |
| rs10411345 | C | G | -0.1041 | 0.0218 | 1.733E-06 | 22.7964477 | 0.0299 | 0.3761 |
| rs10757514 | C | G | -0.1024 | 0.0222 | 4.169E-06 | 21.2702537 | 0.0326 | 0.501601 |
| rs10822118 | T | C | -0.0797 | 0.0168 | 2.211E-06 | 22.499703 | 0.0251 | 0.2833 |
| rs10934631 | T | C | -0.1132 | 0.0244 | 3.607E-06 | 21.5175047 | 0.0356 | 0.415 |
| rs114773511 | T | C | 0.2187 | 0.0441 | 6.971E-07 | 24.5866349 | 0.0623 | 0.1836 |
| rs12456390 | T | C | -0.0818 | 0.0179 | 4.882E-06 | 20.8775365 | 0.0269 | 0.9152 |
| rs1730969 | C | G | -0.7811 | 0.1696 | 4.106E-06 | 21.2027728 | 0.2901 | 0.9261 |
| rs181031888 | A | T | 0.3767 | 0.0498 | 4.014E-14 | 57.2020062 | 0.0692 | 0.5095 |
| rs2039420 | C | G | 0.0975 | 0.0175 | 2.744E-08 | 31.0321481 | 0.026 | 0.2704 |
| rs56071907 | T | C | 0.126 | 0.027 | 0.00000301 | 21.7716963 | 0.036 | 0.6307 |
| rs60013354 | A | G | -0.2497 | 0.0521 | 1.662E-06 | 22.9636196 | 0.0737 | 0.9066 |
| rs62401205 | A | C | -0.2015 | 0.0412 | 9.946E-07 | 23.9130171 | 0.0509 | 0.5739 |
| rs6496613 | A | C | -0.2359 | 0.0515 | 4.701E-06 | 20.9758771 | 0.0778 | 0.9771 |
| rs7356919 | A | G | -0.1694 | 0.02 | 2.846E-17 | 71.7208662 | 0.0292 | 0.0603698 |
| rs73872715 | T | C | -0.6079 | 0.1299 | 2.864E-06 | 21.8939787 | 0.1625 | 0.1863 |
| rs76458389 | T | G | -0.1786 | 0.0363 | 8.573E-07 | 24.2007243 | 0.0545 | 0.00468198 |
| rs77961527 | A | G | 0.2289 | 0.0457 | 5.525E-07 | 25.0805982 | 0.0588 | 0.2903 |
| rs9381249 | T | C | -0.2414 | 0.0396 | 1.038E-09 | 37.1503462 | 0.0622 | 0.2709 |

**Table S5. MR estimates of 41 inflammatory cytokines on glaucoma.**

|  | **IVW** | | | | **MR-Egger** | | | | **Weighted Median** | | | | **Simple Mode** | | | | **Weighted Mode** | | | |
| --- | --- | --- | --- | --- | --- | --- | --- | --- | --- | --- | --- | --- | --- | --- | --- | --- | --- | --- | --- | --- |
| **Exposures** | **No.of SNPs** | **OR** | **95% CI** | **pval** | **No.of SNPs** | **OR** | **95% CI** | **pval** | **No.of SNPs** | **OR** | **95% CI** | **pval** | **No.of SNPs** | **OR** | **95% CI** | **pval** | **No.of SNPs** | **OR** | **95% CI** | **pval** |
| CTACK | 12 | 1.040 | (0.957-1.131) | 0.356 | 12 | 1.040 | (0.881-1.227) | 0.654 | 12 | 1.005 | (0.901-1.120) | 0．934 | 12 | 0.983 | (0.829-1.164) | 0.843 | 12 | 0.991 | (0.832-1.181) | 0.924 |
| Eotaxin | 17 | 1.009 | (0.921-1.105) | 0.847 | 17 | 1.194 | (0.957-1.489) | 0.138 | 17 | 0.993 | (0.873-1.129) | 0.911 | 17 | 0.967 | (0.789-1.210) | 0.772 | 17 | 0.977 | (0.789-1.210) | 0.832 |
| GROa | 13 | 1.038 | (0.931-1.157) | 0.499 | 13 | 0.961 | (0.793-1.166) | 0.696 | 13 | 0.948 | (0.851-1.056) | 0.334 | 13 | 0.939 | (0.806-1.094) | 0.437 | 13 | 0.943 | (0.805-1.104) | 0.480 |
| IP-10 | 12 | 0.999 | (0.919-1.087) | 0.987 | 12 | 1.017 | (0.834-1.241) | 0.867 | 12 | 0.994 | (0.884-1.157) | 0.923 | 12 | 0.961 | (0.779-1.186) | 0.718 | 12 | 0.970 | (0.787-1.196) | 0.781 |
| MCP-1 | 16 | 1.059 | (0.956-1.174) | 0.269 | 16 | 1.035 | (0.780-1.373) | 0.816 | 16 | 0.992 | (0.862-1.142) | 0.911 | 16 | 0.941 | (0.739-1.200) | 0.632 | 16 | 0.964 | (0.777-1.195) | 0.743 |
| MCP-3 | 6 | 0.974 | (0.907-1.046) | 0.471 | 6 | 0.856 | (0.720-1.018) | 0.153 | 6 | 0.976 | (0.900-1.060) | 0.570 | 6 | 0.903 | (0.775-1.053) | 0.250 | 6 | 1.045 | (0.903-1.208) | 0.582 |
| MIG | 13 | 0.990 | (0.915-1.072) | 0.276 | 13 | 1.096 | (0.937-1.283) | 0.276 | 13 | 0.960 | (0.864-1.068) | 0.455 | 13 | 0.940 | (0.797-1.110) | 0.481 | 13 | 0.942 | (0.792-1.121) | 0.514 |
| MIP-1A | 4 | 0.939 | (0.780-1.130) | 0.505 | 4 | 0.787 | (0.407-1.523) | 0.551 | 4 | 0.937 | (0.783-1.121) | 0.478 | 4 | 1.092 | (0.775-1.537) | 0.650 | 4 | 0.826 | (0.597-1.144) | 0.333 |
| MIP-1B | 22 | 1.056 | (0.961-1.161) | 0.256 | 22 | 1.164 | (0.890-1.523) | 0.281 | 22 | 1.019 | (0.907-1.144) | 0.757 | 22 | 1.305 | (1.001-1.701) | 0.063 | 22 | 1.289 | (1.006-1.651) | 0.058 |
| RANTES | 10 | 0.886 | (0.810-0.969) | 0.008 | 10 | 0.895 | (0.716-1.120) | 0.361 | 10 | 0.870 | (0.771-0.981) | 0.023 | 10 | 0.855 | (0.709-1.031) | 0.135 | 10 | 0.858 | (0.706-1.044) | 0.160 |
| SDF-1A | 7 | 0.997 | (0.817-1.217) | 0.976 | 7 | 1.007 | (0.681-1.489) | 0.973 | 7 | 0.930 | (0.752-1.150) | 0.504 | 7 | 0.928 | (0.693-1.243) | 0.636 | 7 | 0.926 | (0.718-1.195) | 0.577 |
| SCGF-B | 21 | 0.982 | (0.913-1.057) | 0.633 | 21 | 1.008 | (0.863-1.177) | 0.923 | 21 | 0.991 | (0.911-1.078) | 0.831 | 21 | 0.959 | (0.824-1.116) | 0.596 | 21 | 0.983 | (0.871-1.110) | 0.783 |
| B-NGF | 4 | 0.966 | (0.805-1.158) | 0.706 | 4 | 2.118 | (1.054-4.255) | 0.170 | 4 | 0.989 | (0.832-1.175) | 0.900 | 4 | 1.013 | (0.753-1.364) | 0.937 | 4 | 1.028 | (0.821-1.287) | 0.824 |
| FGF2 | 7 | 1.113 | (0.882-1.404) | 0.366 | 7 | 1.129 | (0.532-2.396) | 0.764 | 7 | 0.977 | (0.771-1.238) | 0.848 | 7 | 0.883 | (0.617-1.263) | 0.665 | 7 | 0.883 | (0.617-1.263) | 0.521 |
| G-CSF | 9 | 1.009 | (0.885-1.150) | 0.895 | 9 | 1.004 | (0.801-1.260) | 0.972 | 9 | 1.015 | (0.861-1.196) | 0.860 | 9 | 0.954 | (0.733-1.241) | 0.735 | 9 | 1.084 | (0.733-1.241) | 0.536 |
| HGF | 9 | 0.941 | (0.776-1.142) | 0.538 | 9 | 0.863 | (0.551-1.354) | 0.543 | 9 | 0.863 | (0.714-1.043) | 0.128 | 9 | 0.864 | (0.678-1.101) | 0.270 | 9 | 0.832 | (0.659-1.052) | 0.163 |
| M-CSF | 12 | 1.064 | (0.977-1.158) | 0.155 | 12 | 1.131 | (0.944-1.355) | 0.210 | 12 | 1.079 | (0.979-1.189) | 0.127 | 12 | 1.173 | (0.990-1.389) | 0.092 | 12 | 1.173 | (0.990-1.389) | 0.133 |
| PDGF-BB | 14 | 1.027 | (0.880-0.198) | 0.739 | 14 | 0.940 | (0.645-1.369) | 0.751 | 14 | 1.037 | (0.877-1.226) | 0.670 | 14 | 1.007 | (0.765-1.326) | 0.959 | 14 | 1.016 | (0.786-1.312) | 0.908 |
| SCF | 10 | 1.069 | (0.935-1.222) | 0.331 | 10 | 1.241 | (0.937-1.645) | 0.171 | 10 | 1.182 | (0.937-1.645) | 0.171 | 10 | 1.207 | (0.896-1.625) | 0.247 | 10 | 1.209 | (0.892-1.639) | 0.253 |
| VEGF | 18 | 0.984 | (0.902-1.072) | 0.706 | 18 | 1.100 | (0.916-1.321) | 0.321 | 18 | 0.973 | (0.864-1.095) | 0.649 | 18 | 1.006 | (0.821-1.234) | 0.952 | 18 | 0.966 | (0.813-1.147) | 0.696 |
| IL-10 | 15 | 0.965 | (0.852-1.093) | 0.573 | 15 | 1.121 | (0.743-1.690) | 0.595 | 15 | 0.972 | (0.743-1.690) | 0.688 | 15 | 0.921 | (0.738-1.149) | 0.478 | 15 | 0.952 | (0.813-1.115) | 0.551 |
| IL-12-p70 | 15 | 1.002 | (0.877-1.145) | 0.973 | 15 | 1.436 | (0.911-2.265) | 0.144 | 15 | 1.032 | (0.878-1.213) | 0.705 | 15 | 1.090 | (0.838-1.419) | 0.531 | 15 | 1.041 | (0.805-1.345) | 0.765 |
| IL-13 | 14 | 1.040 | (0.970-1.115) | 0.265 | 14 | 1.031 | (0.905-1.175) | 0.652 | 14 | 1.027 | (0.935-1.128) | 0.581 | 14 | 1.007 | (0.860-1.180) | 0.930 | 14 | 1.018 | (0.878-1.179) | 0.820 |
| IL-16 | 10 | 1.048 | (0.971-1.131) | 0.227 | 10 | 1.019 | (0.900-1.154) | 0.771 | 10 | 1.045 | (0.944-1.156) | 0.395 | 10 | 1.123 | (0.944-1.335) | 0.223 | 10 | 1.078 | (0.925-1.256) | 0.363 |
| IL-17 | 8 | 0.970 | (0.846-1.114) | 0.673 | 8 | 1.047 | (0.801-1.368) | 0.747 | 8 | 0.960 | (0.807-1.141) | 0.641 | 8 | 0.953 | (0.720-1.260) | 0.744 | 8 | 0.960 | (0.784-1.176) | 0.708 |
| IL-18 | 13 | 1.087 | (0.964-1.225) | 0.173 | 13 | 1.186 | (0.940-1.498) | 0.179 | 13 | 1.029 | (0.913-1.161) | 0.639 | 13 | 1.004 | (0.828-1.217) | 0.970 | 13 | 1.001 | (0.821-1.221) | 0.991 |
| IL-1B | 3 | 0.884 | (0.703-1.111) | 0.291 | 3 | 0.673 | (0.469-0.966) | 0.277 | 3 | 0.896 | (0.702-1.145) | 0.381 | 3 | 0.833 | (0.581-1.194) | 0.424 | 3 | 1.013 | (0.788-1.301) | 0.327 |
| IL-1RA | 10 | 1.008 | (0.919-1.182) | 0.859 | 10 | 1.042 | (0.919-1.182) | 0.517 | 10 | 1.042 | (0.919-1.182) | 0.517 | 10 | 1.076 | (0.879-1.318) | 0.496 | 10 | 1.072 | (0.885-1.230) | 0.494 |
| IL-2 | 8 | 0.968 | (0.867-1.080) | 0.555 | 8 | 0.870 | (0.702-1.078) | 0.250 | 8 | 0.966 | (0.846-1.103) | 0.613 | 8 | 0.963 | (0.806-1.151) | 0.612 | 8 | 0.963 | (0.806-1.151) | 0.693 |
| IL-2RA | 9 | 0.929 | (0.844-1.021) | 0.126 | 9 | 0.739 | (0.739-1.068) | 0.247 | 9 | 0.907 | (0.807-1.020) | 0.102 | 9 | 0.904 | (0.729-1.121) | 0.386 | 9 | 0.865 | (0.715-1.047) | 0.175 |
| IL-4 | 14 | 1.018 | (0.890-1.165) | 0.790 | 14 | 1.155 | (0.871-1.529) | 0.336 | 14 | 1.005 | (0.855-1.182) | 0.948 | 14 | 0.929 | (0.702-1.230) | 0.617 | 14 | 0.938 | (0.709-1.241) | 0.661 |
| IL-5 | 8 | 0.951 | (0.864-1.047) | 0.304 | 8 | 1.009 | (0.819-1.243) | 0.937 | 8 | 0.942 | (0.837-1.059) | 0.315 | 8 | 0.926 | (0.771-1.113) | 0.442 | 8 | 0.933 | (0.783-1.112) | 0.464 |
| IL-6 | 11 | 1.064 | (0.921-1.229) | 0.398 | 11 | 0.956 | (0.685-1.334) | 0.798 | 11 | 1.077 | (0.903-1.284) | 0.412 | 11 | 1.106 | (0.805-1.519) | 0.549 | 11 | 1.081 | (0.785-1.491) | 0.643 |
| IL-7 | 12 | 0.994 | (0.927-1.066) | 0.867 | 12 | 1.083 | (0.920-1.275) | 0.360 | 12 | 0.953 | (0.864-1.052) | 0.339 | 12 | 0.933 | (0.791-1.100) | 0.424 | 12 | 0.941 | (0.823-1.075) | 0.389 |
| IL-8 | 8 | 1.001 | (0.906-1.106) | 0.985 | 8 | 1.090 | (0.906-1.313) | 0.396 | 8 | 1.007 | (0.885-1.146) | 0.911 | 8 | 0.970 | (0.785-1.198) | 0.785 | 8 | 0.988 | (0.809-1.206) | 0.907 |
| IL-9 | 6 | 0.946 | (0.830-1.079) | 0.407 | 6 | 1.058 | (0.757-1.479) | 0.757 | 6 | 0.927 | (0.792-1.085) | 0.345 | 6 | 0.850 | (0.655-1.105) | 0.280 | 6 | 0.805 | (0.651-1.112) | 0.289 |
| IFN-G | 12 | 1.027 | (0.861-1.226) | 0.764 | 12 | 1.523 | (0.181-1.962) | 0.009 | 12 | 1.076 | (0.908-1.274) | 0.400 | 12 | 1.030 | (0.784-1.354) | 0.835 | 12 | 1.056 | (0.802-1.391) | 0.704 |
| MIF | 10 | 0.987 | (0.896-1.086 | 0.784 | 10 | 0.939 | (0.772-1.143) | 0.550 | 10 | 1.009 | (0.884-1.151) | 0.894 | 10 | 1.044 | (0.852-1.278) | 0.690 | 10 | 1.057 | (0.873-1.281) | 0.584 |
| TNF-A | 4 | 0.943 | (0.821-1.083) | 0.404 | 4 | 0.930 | (0.740-1.169) | 0.597 | 4 | 0.966 | (0.818-1.140) | 0.681 | 4 | 0.977 | (0.771-1.238) | 0.861 | 4 | 0.983 | (0.781-1.238) | 0.895 |
| TNF-B | 5 | 1.046 | (0.960-1.140) | 0.305 | 5 | 1.090 | (0.897-1.324) | 0.449 | 5 | 1.042 | (0.942-1.153) | 0.421 | 5 | 1.043 | (0.886-1.228) | 0.638 | 5 | 1.053 | (0.901-1.231) | 0.552 |
| TRAIL | 16 | 1.017 | (0.935-1.107) | 0.697 | 16 | 0.976 | (0.862-1.106) | 0.709 | 16 | 0.980 | (0.886-1.084) | 0.698 | 16 | 1.031 | (0.853-1.247) | 0.755 | 16 | 1.981 | (0.887-1.085) | 0.716 |

Abbreviations: CI, Confidence interval; OR, Odds Ratio; pval, P-value; SNPs, single nucleotide polymorphisms. OR and 95% CI represent the change in the odds ratio of glaucoma per 1 SD increase in inflammatory cytokines.

**Table S6. Heterogeneity and horizontal pleiotropy tests of 41 inflammatory cytokines on glaucoma**

| **Exposures** | **Q_1_ pval (IVW)** | **Q_2_ pval (MR-Egger)** | **I^2^** | **intercept** | **intercept pval** |
| --- | --- | --- | --- | --- | --- |
| B-NGF | 0.144 | 0.840 | 48.2% | -0.112 | 0.153 |
| CTACK | 0.778 | 0.701 | 0.0% | 0.0001 | 0.995 |
| Eotaxin | 0.474 | 0.599 | 0.0% | -0.022 | 0.123 |
| FGF2 | 0.043 | 0.023 | 54.1% | -0.002 | 0.970 |
| G-CSF | 0.978 | 0.954 | 0.0% | -0.001 | 0.963 |
| GROa | 0.014 | 0.016 | 52.6% | 0.023 | 0.363 |
| HGF | 0.051 | 0.035 | 48.2% | 0.012 | 0.686 |
| IFN-G | 0.005 | 0.281 | 62.6% | -0.057 | 0.006 |
| IL-1B | 0.197 | 0.566 | 0.0% | 0.056 | 0.337 |
| IL-1RA | 0.426 | 0.751 | 0.0% | 0.040 | 0.078 |
| IL-2 | 0.185 | 0.216 | 30.4% | 0.020 | 0.304 |
| IL-2RA | 0.233 | 0.187 | 30.2% | 0.010 | 0.590 |
| IL-4 | 0.139 | 0.146 | 29.8% | -0.017 | 0.339 |
| IL-5 | 0.957 | 0.948 | 0.0% | -0.013 | 0.556 |
| IL-6 | 0.245 | 0.215 | 24.9% | 0.245 | 0.500 |
| IL-7 | 0.449 | 0.473 | 0.0% | -0.019 | 0.280 |
| IL-8 | 0.472 | 0.488 | 0.0% | -0.018 | 0.325 |
| IL-9 | 0.376 | 0.317 | 6.3% | -0.017 | 0.465 |
| IL-10 | 0.027 | 0.025 | 45.7% | -0.031 | 0.226 |
| IL-12p70 | 0.069 | 0.131 | 37.7% | -0.039 | 0.131 |
| IL-13 | 0.719 | 0.645 | 0.0% | 0.002 | 0.878 |
| IL-16 | 0.667 | 0.602 | 0.0% | 0.009 | 0.593 |
| IL-17 | 0.283 | 0.237 | 18.6% | -0.014 | 0.534 |
| IL-18 | 0.004 | 0.004 | 58.9% | -0.018 | 0.407 |
| IP-10 | 0.421 | 0.340 | 2.3% | -0.003 | 0.846 |
| M-CSF | 0.057 | 0.052 | 42.8% | -0.021 | 0.463 |
| MCP-1 | 0.642 | 0.570 | 0.0% | 0.003 | 0.864 |
| MCP-3 | 0.306 | 0.475 | 16.7% | 0.036 | 0.190 |
| MIF | 0.367 | 0.308 | 8.2% | 0.011 | 0.585 |
| MIG | 0.365 | 0.446 | 8.1% | -0.025 | 0.176 |
| MIP-1A | 0.194 | 0.129 | 36.4% | 0.030 | 0.637 |
| MIP-1B | 0.135 | 0.125 | 25.4% | -0.013 | 0.457 |
| PDGF-BB | 0.028 | 0.021 | 46.7% | 0.011 | 0.620 |
| RANTES | 0.802 | 0.719 | 0.0% | -0.002 | 0.920 |
| SCF | 0.545 | 0.593 | 0.0% | -0.022 | 0.271 |
| SCGF-B | 0.053 | 0.041 | 35.8% | -0.006 | 0.717 |
| SDF-1A | 0.114 | 0.070 | 41.5% | -0.002 | 0.954 |
| TNF-A | 0.520 | 0.327 | 0.0% | 0.004 | 0.891 |
| TNF-B | 0.294 | 0.205 | 18.9% | -0.013 | 0.666 |
| TRAIL | 0.148 | 0.145 | 27.4% | 0.010 | 0.392 |
| VEGF | 0.368 | 0.422 | 7.3% | -0.019 | 0.194 |

Q1 pval: P-value of Q test from IVW method; Q2 pval: P-value of Q test from MR-Egger method.

Abbreviations: pval, P-value; Q, Cochran Q statistics; SNPs, single nucleotide polymorphisms; IVW, the inverse variance weighted method.

**Table S7. SNPs information of 41 inflammation cytokines with glaucoma**

|  | **Inflammatory cytokines (exposure)** | | | | | **glaucoma (outcome)** | | |
| --- | --- | --- | --- | --- | --- | --- | --- | --- |
| **SNP** | **effect allele** | **other allele** | **Beta** | **se** | **pval** | **F** | **se** | **pval** |
| **B-NGF** | | | | | | | | |
| rs28637706 | T | G | -0.1554 | 0.0261 | 2.717E-09 | 35.4305296 | 0.0183 | 0.7388 |
| rs4767014 | T | C | -0.1211 | 0.0264 | 4.518E-06 | 21.029893 | 0.0179 | 0.4519 |
| rs71641308 | T | C | 0.1969 | 0.0429 | 4.424E-06 | 21.0539447 | 0.0287 | 0.2523 |
| rs73472576 | T | C | -0.1146 | 0.0251 | 4.813E-06 | 20.8342764 | 0.017 | 0.0552904 |
| **CTACK** | | | | | | | | |
| rs10854859 | A | G | -0.1498 | 0.0293 | 3.049E-07 | 26.1247203 | 0.0202 | 0.9448 |
| rs116303454 | A | G | 0.3754 | 0.081 | 3.579E-06 | 21.4675143 | 0.0528 | 0.4537 |
| rs116871507 | A | T | -0.2086 | 0.0448 | 0.00000316 | 21.668849 | 0.0331 | 0.774599 |
| rs116943377 | A | G | 0.2878 | 0.0611 | 2.496E-06 | 22.1749384 | 0.0437 | 0.537 |
| rs117932939 | T | C | 0.1969 | 0.0422 | 3.089E-06 | 21.7585421 | 0.0303 | 0.631899 |
| rs118084576 | A | G | 0.5675 | 0.1226 | 0.00000366 | 21.4148015 | 0.0946 | 0.0571702 |
| rs184329319 | T | G | -0.3069 | 0.0648 | 2.173E-06 | 22.4185246 | 0.0472 | 0.9846 |
| rs55764737 | T | C | 0.5424 | 0.0967 | 2.012E-08 | 31.4448608 | 0.0695 | 0.9976 |
| rs57338032 | A | G | 0.1443 | 0.0316 | 4.831E-06 | 20.8411539 | 0.0225 | 0.744201 |
| rs57789542 | T | C | -0.7687 | 0.1659 | 3.575E-06 | 21.4577535 | 0.1034 | 0.6546 |
| rs60247384 | T | C | 0.1128 | 0.0245 | 4.302E-06 | 21.1860159 | 0.0176 | 0.1219 |
| rs76395525 | A | G | 0.5193 | 0.1081 | 1.553E-06 | 23.0647377 | 0.0833 | 0.4671 |
| **Eotaxin** | | | | | | | | |
| rs11087905 | A | C | 0.0954 | 0.0188 | 4.07E-07 | 25.7439435 | 0.0178 | 0.5212 |
| rs112347425 | T | C | 0.1595 | 0.0276 | 7.771E-09 | 33.388525 | 0.0294 | 0.0353403 |
| rs11920996 | T | C | 0.2979 | 0.0377 | 2.919E-15 | 62.4241056 | 0.0398 | 0.0287303 |
| rs1677588 | T | G | 0.1181 | 0.025 | 2.223E-06 | 22.310731 | 0.0265 | 0.8797 |
| rs2024050 | A | G | 0.164 | 0.0302 | 5.467E-08 | 29.4827394 | 0.0326 | 0.2434 |
| rs2027855 | T | C | 0.0743 | 0.0162 | 4.272E-06 | 21.0301137 | 0.0172 | 0.8243 |
| rs2040143 | A | G | -0.0858 | 0.0178 | 1.333E-06 | 23.2288973 | 0.0187 | 0.9266 |
| rs2229593 | T | C | 0.3647 | 0.0406 | 2.838E-19 | 80.6702648 | 0.0431 | 0.955 |
| rs2249581 | T | C | -0.0899 | 0.018 | 5.912E-07 | 24.9383891 | 0.0192 | 0.9823 |
| rs5754733 | A | C | -0.105 | 0.0213 | 8.196E-07 | 24.2948048 | 0.0224 | 0.792899 |
| rs57723662 | C | G | -0.0982 | 0.0213 | 3.878E-06 | 21.2499441 | 0.0222 | 0.3149 |
| rs60075014 | T | C | -0.1688 | 0.0356 | 2.078E-06 | 22.4770293 | 0.0344 | 0.746 |
| rs7231030 | A | C | 0.0903 | 0.0193 | 2.709E-06 | 21.885421 | 0.0204 | 0.3395 |
| rs73072941 | A | T | -0.1281 | 0.0263 | 1.113E-06 | 23.7181486 | 0.0263 | 0.5282 |
| rs745331 | A | G | -0.0821 | 0.0176 | 3.036E-06 | 21.7547953 | 0.0182 | 0.1525 |
| rs75426604 | A | C | -0.1371 | 0.0291 | 2.397E-06 | 22.1913107 | 0.0292 | 0.7062 |
| rs9317045 | A | C | 0.1172 | 0.0236 | 6.954E-07 | 24.6561487 | 0.0238 | 0.9711 |
| **FGF2** | | | | | | | | |
| rs13412535 | A | G | -0.1129 | 0.0224 | 4.763E-07 | 25.3967228 | 0.0211 | 0.272 |
| rs147409637 | T | C | 0.201 | 0.0431 | 3.077E-06 | 21.7431973 | 0.0455 | 0.0287899 |
| rs17094040 | T | C | 0.1051 | 0.0229 | 4.308E-06 | 21.0581541 | 0.024 | 0.0767397 |
| rs2849358 | A | G | 0.0911 | 0.0193 | 2.321E-06 | 22.2745002 | 0.0194 | 0.8803 |
| rs4795091 | A | G | 0.1239 | 0.0266 | 3.055E-06 | 21.6902807 | 0.0271 | 0.402 |
| rs76253061 | T | C | -0.4811 | 0.1041 | 3.807E-06 | 21.3528125 | 0.1053 | 0.6131 |
| rs78873483 | A | G | 0.1286 | 0.0282 | 0.00000498 | 20.7907211 | 0.0266 | 0.0298202 |
| **G-CSF** | | | | | | | | |
| rs10939033 | A | G | -0.0775 | 0.0163 | 2.074E-06 | 22.6005443 | 0.0172 | 0.8912 |
| rs117261691 | T | C | 0.1318 | 0.0288 | 4.669E-06 | 20.9380652 | 0.0305 | 0.763801 |
| rs183023730 | T | G | 0.7898 | 0.1677 | 2.471E-06 | 22.1734096 | 0.1344 | 0.729001 |
| rs586802 | A | G | 0.0882 | 0.0187 | 2.398E-06 | 22.2405058 | 0.0195 | 0.5278 |
| rs6740648 | T | C | 0.0818 | 0.0172 | 0.0000019 | 22.6120749 | 0.018 | 0.7466 |
| rs74148555 | T | C | -0.3771 | 0.0753 | 5.591E-07 | 25.0734338 | 0.0595 | 0.4798 |
| rs76287671 | T | C | 0.0894 | 0.0189 | 2.191E-06 | 22.3687714 | 0.0197 | 0.8819 |
| **GROa** | | | | | | | | |
| rs114991247 | T | C | -0.2202 | 0.0463 | 1.971E-06 | 22.6061961 | 0.0282 | 0.0002256 |
| rs115214168 | T | C | 0.4528 | 0.0828 | 4.479E-08 | 29.8886987 | 0.0584 | 0.623799 |
| rs1361829 | A | G | -0.1106 | 0.0241 | 4.576E-06 | 21.0489779 | 0.0169 | 0.1969 |
| rs140734053 | A | G | 0.7333 | 0.1545 | 2.069E-06 | 22.514445 | 0.0899 | 0.5978 |
| rs150194856 | T | C | -0.4223 | 0.0914 | 3.857E-06 | 21.3355879 | 0.0646 | 0.5433 |
| rs17171245 | T | G | 0.2446 | 0.053 | 3.932E-06 | 21.287076 | 0.0393 | 0.2539 |
| rs185768063 | A | G | 0.4038 | 0.076 | 1.055E-07 | 28.2137159 | 0.0519 | 0.2546 |
| rs188345231 | T | C | 0.6177 | 0.1322 | 2.968E-06 | 21.8196176 | 0.0967 | 0.9224 |
| rs3026943 | A | C | -0.1246 | 0.0256 | 1.084E-06 | 23.6761454 | 0.0179 | 0.01376 |
| rs62024303 | A | G | -0.3013 | 0.066 | 4.908E-06 | 20.8288473 | 0.0422 | 0.7272 |
| rs76215157 | C | G | -0.7398 | 0.1564 | 2.226E-06 | 22.3619669 | 0.0857 | 0.9528 |
| rs76390238 | C | G | 0.6223 | 0.1352 | 4.141E-06 | 21.17391 | 0.0896 | 0.3433 |
| rs79454658 | T | C | 0.2784 | 0.0596 | 3.017E-06 | 21.8072442 | 0.0413 | 0.6233 |
| **HGF** | | | | | | | | |
| rs11060254 | A | G | -0.0765 | 0.0166 | 3.974E-06 | 21.2325661 | 0.0181 | 0.798 |
| rs11129909 | T | C | -0.0738 | 0.0161 | 4.457E-06 | 21.0066482 | 0.0174 | 0.4066 |
| rs13412535 | A | G | -0.1043 | 0.0213 | 9.671E-07 | 23.9720514 | 0.0211 | 0.272 |
| rs2003620 | T | C | 0.2277 | 0.0487 | 2.978E-06 | 21.8556602 | 0.0506 | 0.77 |
| rs362307 | T | C | 0.1511 | 0.0328 | 0.00000421 | 21.2166607 | 0.0335 | 0.5343 |
| rs4245058 | T | C | -0.1552 | 0.0331 | 2.683E-06 | 21.9797747 | 0.035 | 0.549 |
| rs57146176 | A | G | -0.0987 | 0.0208 | 2.184E-06 | 22.5114477 | 0.0334 | 0.003046 |
| rs5745687 | T | C | -0.3008 | 0.0404 | 9.922E-14 | 55.4228333 | 0.0419 | 0.0555699 |
| rs80051150 | T | C | 0.198 | 0.0413 | 1.684E-06 | 22.9787324 | 0.0464 | 0.3381 |
| **IFN-G** | | | | | | | | |
| rs113399544 | A | G | -0.0849 | 0.0183 | 3.323E-06 | 21.5179571 | 0.0187 | 0.223 |
| rs113600793 | A | C | 0.1871 | 0.0371 | 4.426E-07 | 25.4265591 | 0.0362 | 0.0243097 |
| rs115729819 | A | G | 0.2511 | 0.0514 | 1.045E-06 | 23.8591531 | 0.0525 | 0.5384 |
| rs117046255 | T | C | -0.0968 | 0.0207 | 2.787E-06 | 21.8624013 | 0.0216 | 0.7377 |
| rs11843756 | T | G | 0.1812 | 0.0391 | 3.622E-06 | 21.4709227 | 0.0411 | 0.753999 |
| rs12420286 | T | C | 0.2357 | 0.05 | 2.452E-06 | 22.2160576 | 0.0517 | 0.8592 |
| rs147378920 | A | G | -0.384 | 0.0751 | 3.195E-07 | 26.1378831 | 0.0588 | 0.0106299 |
| rs1867282 | T | C | 0.0781 | 0.0166 | 2.478E-06 | 22.1296084 | 0.0171 | 0.524901 |
| rs2073438 | A | G | 0.092 | 0.0188 | 9.551E-07 | 23.9413036 | 0.0192 | 0.745 |
| rs7088799 | T | G | -0.0805 | 0.0166 | 1.274E-06 | 23.5105843 | 0.0173 | 0.0006974 |
| rs73479333 | C | G | -0.1123 | 0.024 | 2.816E-06 | 21.8889468 | 0.025 | 0.3773 |
| rs74148555 | T | C | -0.3771 | 0.077 | 9.858E-07 | 23.9783586 | 0.0595 | 0.4798 |
| **IL-1B** | | | | | | | | |
| rs143319329 | T | C | 0.4357 | 0.093 | 2.835E-06 | 21.9356097 | 0.1038 | 0.5282 |
| rs4786740 | A | C | 0.1264 | 0.0265 | 0.00000182 | 22.7375092 | 0.0172 | 0.723199 |
| rs61335305 | A | C | 0.4333 | 0.0928 | 3.015E-06 | 21.7882281 | 0.0631 | 0.0333903 |
| **IL-1RA** | | | | | | | | |
| rs1054402 | T | C | 0.1325 | 0.0269 | 8.201E-07 | 24.2488368 | 0.0194 | 0.5757 |
| rs117181659 | A | G | -0.2204 | 0.0478 | 0.00000392 | 21.2486381 | 0.0344 | 0.667 |
| rs11869294 | C | G | -0.2286 | 0.047 | 1.128E-06 | 23.6439727 | 0.0314 | 0.7362 |
| rs13343438 | A | G | 0.2771 | 0.0607 | 4.973E-06 | 20.8285666 | 0.0441 | 0.8712 |
| rs35590641 | C | G | -0.1167 | 0.025 | 3.041E-06 | 21.7783718 | 0.0178 | 0.366 |
| rs3876037 | A | G | 0.1234 | 0.027 | 4.733E-06 | 20.8769237 | 0.0175 | 0.3222 |
| rs56134659 | A | G | -0.1109 | 0.0236 | 2.564E-06 | 22.0700237 | 0.0168 | 0.2385 |
| rs61335305 | A | C | 0.4315 | 0.0904 | 1.812E-06 | 22.7713329 | 0.0631 | 0.0333903 |
| rs6699436 | A | G | -0.1858 | 0.0404 | 4.365E-06 | 21.1393876 | 0.0271 | 0.8206 |
| rs9985296 | T | C | 0.1053 | 0.0231 | 4.952E-06 | 20.7680871 | 0.0169 | 0.3799 |
| **IL-2** | | | | | | | | |
| rs13412535 | A | G | 0.174 | 0.0331 | 1.447E-07 | 27.6181929 | 0.0211 | 0.272 |
| rs16836080 | A | G | 0.1158 | 0.0253 | 4.841E-06 | 20.9376929 | 0.018 | 0.1633 |
| rs2690020 | A | G | 0.1158 | 0.0245 | 2.273E-06 | 22.3273767 | 0.0169 | 0.2427 |
| rs4479767 | A | G | 0.1821 | 0.0392 | 3.444E-06 | 21.5675348 | 0.0289 | 0.9194 |
| rs4634519 | A | G | -0.1249 | 0.0268 | 0.00000318 | 21.7074115 | 0.0189 | 0.535 |
| rs61335305 | A | C | 0.4439 | 0.0913 | 1.157E-06 | 23.6255259 | 0.0631 | 0.0333903 |
| rs62124990 | T | G | -0.7013 | 0.149 | 2.502E-06 | 22.1405238 | 0.0623 | 0.4081 |
| rs7615304 | A | G | -0.1139 | 0.024 | 2.161E-06 | 22.5101151 | 0.0169 | 0.5093 |
| **IL-2RA** | | | | | | | | |
| rs11241559 | T | G | -0.124 | 0.0264 | 0.00000275 | 22.0496505 | 0.0193 | 0.2166 |
| rs117244812 | A | G | -0.7187 | 0.1493 | 1.474E-06 | 23.1601767 | 0.1014 | 0.7972 |
| rs12789243 | T | C | 0.1263 | 0.0276 | 4.608E-06 | 20.9293013 | 0.0196 | 0.6519 |
| rs17147986 | A | C | -0.2976 | 0.0337 | 1.069E-18 | 77.9421257 | 0.024 | 0.00729004 |
| rs17624670 | A | G | -0.125 | 0.0273 | 4.639E-06 | 20.9537099 | 0.0194 | 0.293 |
| rs34037190 | A | G | 0.4784 | 0.0935 | 3.134E-07 | 26.1652756 | 0.0516 | 0.9534 |
| rs56213152 | T | C | 0.1269 | 0.0271 | 2.937E-06 | 21.9154734 | 0.0196 | 0.1498 |
| rs759244 | A | T | -0.1094 | 0.0238 | 4.257E-06 | 21.117715 | 0.0172 | 0.4516 |
| rs79100208 | C | G | 0.8345 | 0.1758 | 2.052E-06 | 22.5206611 | 0.1064 | 0.3731 |
| **IL-4** | | | | | | | | |
| rs116705532 | T | G | -0.4675 | 0.0978 | 1.727E-06 | 22.844369 | 0.1028 | 0.67 |
| rs117146485 | T | C | -0.2856 | 0.0625 | 4.945E-06 | 20.8761312 | 0.0626 | 0.04635 |
| rs12238729 | T | C | 0.5271 | 0.1096 | 1.505E-06 | 23.116432 | 0.1355 | 0.8481 |
| rs12640583 | T | G | -0.1104 | 0.0214 | 2.451E-07 | 26.6075108 | 0.0226 | 0.691399 |
| rs17713451 | A | G | 0.1255 | 0.0252 | 6.41E-07 | 24.795908 | 0.0266 | 0.3028 |
| rs1867282 | T | C | 0.0808 | 0.0162 | 5.822E-07 | 24.8706044 | 0.0171 | 0.524901 |
| rs2073438 | A | G | 0.0847 | 0.0183 | 3.725E-06 | 21.4169828 | 0.0192 | 0.745 |
| rs2346020 | A | G | 0.079 | 0.0169 | 2.843E-06 | 21.8461253 | 0.018 | 0.5357 |
| rs2708586 | T | C | -0.0767 | 0.0166 | 3.588E-06 | 21.3436258 | 0.0177 | 0.5814 |
| rs56408830 | A | G | -0.1794 | 0.0365 | 9.075E-07 | 24.151983 | 0.0407 | 0.1518 |
| rs7613691 | A | G | 0.1787 | 0.0382 | 2.962E-06 | 21.8784235 | 0.039 | 0.841 |
| rs79597994 | T | C | -0.5855 | 0.1271 | 4.056E-06 | 21.2156501 | 0.1028 | 0.003271 |
| rs9506111 | A | G | -0.1446 | 0.0314 | 4.081E-06 | 21.2017123 | 0.0333 | 0.3623 |
| rs9941733 | A | G | 0.1156 | 0.0229 | 4.331E-07 | 25.4764171 | 0.0223 | 0.5055 |
| **IL-5** | | | | | | | | |
| rs10178043 | T | G | 0.2579 | 0.0553 | 3.126E-06 | 21.7368748 | 0.0379 | 0.4047 |
| rs148634917 | A | G | -0.517 | 0.1087 | 1.974E-06 | 22.6082361 | 0.0623 | 0.8977 |
| rs28793375 | T | C | 0.1697 | 0.0362 | 2.746E-06 | 21.962978 | 0.0248 | 0.579001 |
| rs72831687 | A | G | -0.5337 | 0.1104 | 1.324E-06 | 23.3561331 | 0.0671 | 0.673701 |
| rs73040118 | T | C | 0.2294 | 0.049 | 2.903E-06 | 21.9048029 | 0.0348 | 0.5684 |
| rs74811276 | A | G | 0.217 | 0.0471 | 4.082E-06 | 21.2139889 | 0.0306 | 0.4404 |
| rs7739450 | A | G | -0.1295 | 0.0256 | 4.05E-07 | 25.5743314 | 0.017 | 0.652501 |
| rs9309063 | T | G | -0.1119 | 0.0245 | 4.866E-06 | 20.8483979 | 0.017 | 0.3735 |
| **IL-6** | | | | | | | | |
| rs10910395 | A | T | -0.108 | 0.0235 | 4.348E-06 | 21.1157384 | 0.0246 | 0.3908 |
| rs10982193 | A | G | -0.0793 | 0.0174 | 4.816E-06 | 20.7654986 | 0.0188 | 0.2382 |
| rs113098456 | A | G | -0.1553 | 0.0339 | 4.641E-06 | 20.9815709 | 0.0326 | 0.7327 |
| rs113600793 | A | C | 0.1736 | 0.0359 | 1.291E-06 | 23.3778749 | 0.0362 | 0.0243097 |
| rs114373846 | T | C | 0.4196 | 0.0905 | 3.568E-06 | 21.4915771 | 0.098 | 0.7428 |
| rs11732981 | A | C | 0.0722 | 0.0156 | 3.793E-06 | 21.4150792 | 0.0168 | 0.7314 |
| rs1333040 | T | C | 0.0747 | 0.0157 | 1.993E-06 | 22.632701 | 0.0168 | 0.1756 |
| rs13412535 | A | G | -0.1186 | 0.0214 | 3.141E-08 | 30.7069242 | 0.0211 | 0.272 |
| rs4684700 | T | C | -0.0747 | 0.0162 | 3.912E-06 | 21.2571805 | 0.017 | 0.5767 |
| rs73273528 | T | C | 0.268 | 0.0553 | 0.00000125 | 23.4808499 | 0.0555 | 0.9324 |
| rs76856708 | T | C | 0.336 | 0.0697 | 1.427E-06 | 23.2331173 | 0.0746 | 0.104 |
| **IL-7** | | | | | | | | |
| rs117509142 | T | C | -0.3213 | 0.0684 | 2.599E-06 | 22.0524712 | 0.0464 | 0.2326 |
| rs11757972 | T | C | 0.121 | 0.0257 | 2.529E-06 | 22.1540183 | 0.017 | 0.6195 |
| rs1374279 | A | T | 0.1625 | 0.0347 | 2.792E-06 | 21.9177413 | 0.0238 | 0.7051 |
| rs142397827 | A | C | 0.4592 | 0.0994 | 3.822E-06 | 21.329418 | 0.0593 | 0.4853 |
| rs17091524 | T | C | 0.5092 | 0.1015 | 5.244E-07 | 25.1531697 | 0.0655 | 0.403 |
| rs2006957 | T | C | 0.2557 | 0.0262 | 1.434E-22 | 95.1934111 | 0.0179 | 0.5104 |
| rs218238 | A | T | 0.1319 | 0.0284 | 3.277E-06 | 21.5576257 | 0.0201 | 0.2056 |
| rs28793375 | T | C | 0.1644 | 0.036 | 4.866E-06 | 20.8423479 | 0.0248 | 0.579001 |
| rs62006410 | T | C | -0.1492 | 0.0302 | 7.588E-07 | 24.3933685 | 0.0197 | 0.1585 |
| rs7155170 | A | T | -0.1236 | 0.027 | 4.787E-06 | 20.9438939 | 0.0188 | 0.7972 |
| rs77318030 | T | C | -0.2966 | 0.0631 | 2.639E-06 | 22.0816535 | 0.042 | 0.4308 |
| rs77981494 | T | C | -0.5201 | 0.1055 | 8.225E-07 | 24.289401 | 0.0606 | 0.0854791 |
| **IL-8** | | | | | | | | |
| rs113487695 | A | C | -0.6129 | 0.1292 | 2.092E-06 | 22.49109 | 0.0936 | 0.6582 |
| rs116726256 | T | C | -0.2247 | 0.0489 | 4.261E-06 | 21.1030251 | 0.0359 | 0.8073 |
| rs12075 | A | G | 0.1148 | 0.0235 | 9.969E-07 | 23.8508763 | 0.0169 | 0.1113 |
| rs12912642 | A | G | 0.1168 | 0.0251 | 3.212E-06 | 21.6418574 | 0.0177 | 0.325 |
| rs183628733 | T | C | 0.6547 | 0.1417 | 3.821E-06 | 21.3354236 | 0.0824 | 0.7973 |
| rs2673604 | A | C | -0.118 | 0.0254 | 3.289E-06 | 21.5701353 | 0.0181 | 0.4833 |
| rs3786107 | A | G | 0.2463 | 0.0517 | 1.935E-06 | 22.6831845 | 0.0324 | 0.906 |
| rs75840288 | A | C | 0.5125 | 0.1121 | 4.846E-06 | 20.8897288 | 0.0719 | 0.1319 |
| **IL-9** | | | | | | | | |
| rs117807175 | C | G | -0.5225 | 0.1106 | 2.327E-06 | 22.3062103 | 0.0756 | 0.651 |
| rs1259728 | A | G | -0.2381 | 0.0507 | 2.599E-06 | 22.0427781 | 0.0362 | 0.542099 |
| rs3736858 | C | G | -0.1351 | 0.0291 | 3.373E-06 | 21.5421069 | 0.0209 | 0.3736 |
| rs41294750 | T | C | 0.3442 | 0.0736 | 2.916E-06 | 21.8589632 | 0.0506 | 0.1058 |
| rs4880409 | T | C | -0.3552 | 0.0716 | 6.952E-07 | 24.5970671 | 0.0838 | 0.3768 |
| rs73443903 | A | C | 0.2162 | 0.046 | 2.569E-06 | 22.0779717 | 0.0335 | 0.252 |
| **IL-10** | | | | | | | | |
| rs10457128 | A | G | -0.0854 | 0.0172 | 6.956E-07 | 24.6459963 | 0.0175 | 0.6106 |
| rs10493718 | A | C | -0.1081 | 0.0222 | 1.068E-06 | 23.7046186 | 0.0231 | 0.5634 |
| rs13412535 | A | G | -0.1347 | 0.0224 | 1.798E-09 | 36.1515304 | 0.0211 | 0.272 |
| rs1530455 | T | C | 0.082 | 0.0174 | 2.527E-06 | 22.2032598 | 0.0176 | 0.0683298 |
| rs2086656 | T | C | -0.08 | 0.017 | 2.589E-06 | 22.1395946 | 0.0177 | 0.8654 |
| rs3002131 | C | G | 0.1191 | 0.026 | 4.592E-06 | 20.9780134 | 0.0246 | 0.0713904 |
| rs3025021 | T | C | 0.0913 | 0.0194 | 2.609E-06 | 22.1424477 | 0.018 | 0.1506 |
| rs383684 | A | G | 0.092 | 0.0197 | 3.168E-06 | 21.8037269 | 0.0293 | 0.3393 |
| rs4741748 | A | G | -0.0788 | 0.0169 | 3.202E-06 | 21.735346 | 0.0172 | 0.4515 |
| rs6054847 | T | C | 0.0971 | 0.0207 | 2.752E-06 | 21.9981065 | 0.0214 | 0.113 |
| rs6680918 | T | C | -0.1202 | 0.025 | 1.591E-06 | 23.1108783 | 0.0257 | 0.6511 |
| rs7088799 | T | G | -0.0815 | 0.0166 | 9.352E-07 | 24.0983093 | 0.0173 | 0.0006974 |
| rs73192842 | A | G | 0.0949 | 0.0206 | 4.031E-06 | 21.2170752 | 0.0215 | 0.6806 |
| rs7747448 | A | G | -0.1061 | 0.0189 | 1.998E-08 | 31.5061032 | 0.0192 | 0.813 |
| rs9472173 | T | C | -0.2004 | 0.0174 | 1.256E-30 | 132.612502 | 0.0173 | 0.8461 |
| **IL-12p70** |  |  |  |  |  |  |  |  |
| rs113600793 | A | C | 0.1832 | 0.0359 | 3.351E-07 | 26.0349984 | 0.0362 | 0.0243097 |
| rs12969892 | T | C | 0.1227 | 0.0267 | 4.194E-06 | 21.1135916 | 0.0279 | 0.3103 |
| rs2123852 | T | C | 0.0942 | 0.0204 | 0.00000373 | 21.317535 | 0.0212 | 0.6924 |
| rs273702 | A | G | -0.127 | 0.027 | 2.522E-06 | 22.1195062 | 0.0281 | 0.9004 |
| rs282258 | T | C | 0.0726 | 0.0156 | 3.282E-06 | 21.6530739 | 0.0169 | 0.464 |
| rs34322762 | T | C | 0.0953 | 0.0199 | 1.708E-06 | 22.9267052 | 0.0176 | 0.00672094 |
| rs34826779 | T | G | -0.0884 | 0.019 | 3.327E-06 | 21.6417733 | 0.0204 | 0.2237 |
| rs41282644 | A | G | 0.1401 | 0.0303 | 3.737E-06 | 21.3740356 | 0.031 | 0.3366 |
| rs4530855 | T | G | 0.0861 | 0.0184 | 2.872E-06 | 21.8910287 | 0.0186 | 0.6618 |
| rs4741748 | A | G | -0.0799 | 0.0163 | 9.162E-07 | 24.0222601 | 0.0172 | 0.4515 |
| rs6532374 | T | C | -0.1033 | 0.0226 | 4.613E-06 | 20.8871545 | 0.0239 | 0.8759 |
| rs7754905 | A | G | -0.1005 | 0.019 | 1.14E-07 | 27.9718014 | 0.0201 | 0.0408498 |
| rs782111 | A | C | -0.0765 | 0.0156 | 9.247E-07 | 24.0419222 | 0.0168 | 0.8167 |
| rs865585 | A | C | -0.1654 | 0.0237 | 2.732E-12 | 48.69337 | 0.0238 | 0.814 |
| rs9381249 | T | C | -0.1788 | 0.0367 | 1.125E-06 | 23.730037 | 0.0416 | 0.3741 |
| **IL-13** | | | | | | | | |
| rs10995604 | A | G | -0.1571 | 0.0343 | 4.482E-06 | 20.966335 | 0.0237 | 0.8111 |
| rs117795020 | A | G | -0.3584 | 0.0716 | 5.479E-07 | 25.0419617 | 0.0496 | 0.6734 |
| rs12623722 | A | G | -0.1189 | 0.0257 | 3.614E-06 | 21.3922198 | 0.0183 | 0.8058 |
| rs138854806 | A | G | -0.4204 | 0.0839 | 5.449E-07 | 25.0934216 | 0.0504 | 0.8403 |
| rs139083458 | T | C | 0.9995 | 0.211 | 2.165E-06 | 22.4263748 | 0.1433 | 0.9522 |
| rs147747784 | C | G | 0.369 | 0.0765 | 1.435E-06 | 23.2534958 | 0.0397 | 0.803 |
| rs150836197 | T | C | 0.3283 | 0.0713 | 4.136E-06 | 21.1895189 | 0.043 | 0.0770602 |
| rs27949 | T | C | -0.1144 | 0.025 | 4.827E-06 | 20.9281299 | 0.018 | 0.1442 |
| rs28442067 | A | G | -0.1379 | 0.0286 | 1.413E-06 | 23.2356516 | 0.0205 | 0.9974 |
| rs7073807 | T | C | 0.1618 | 0.0354 | 0.00000477 | 20.8789618 | 0.0249 | 0.9487 |
| rs75383097 | C | G | -0.5369 | 0.116 | 3.702E-06 | 21.4106188 | 0.0683 | 0.1378 |
| rs76339001 | A | T | -0.4375 | 0.0886 | 7.915E-07 | 24.3695248 | 0.0506 | 0.1543 |
| rs76975337 | T | C | -0.1211 | 0.0265 | 4.921E-06 | 20.8715608 | 0.0191 | 0.514301 |
| rs77955971 | A | C | 0.4408 | 0.0868 | 3.756E-07 | 25.775204 | 0.0469 | 0.4334 |
| **IL-16** | | | | | | | | |
| rs117217798 | T | C | -0.2064 | 0.044 | 2.772E-06 | 21.992129 | 0.0303 | 0.3458 |
| rs12577604 | T | C | 0.4335 | 0.0941 | 4.083E-06 | 21.2105631 | 0.0651 | 0.9863 |
| rs142034902 | A | G | -0.4367 | 0.0925 | 2.327E-06 | 22.2759462 | 0.0663 | 0.1041 |
| rs142332135 | A | G | -0.7646 | 0.1082 | 1.581E-12 | 49.9076715 | 0.0644 | 0.659601 |
| rs144691581 | A | G | 0.4929 | 0.0958 | 2.668E-07 | 26.4569551 | 0.0575 | 0.2325 |
| rs35834666 | T | C | -0.1729 | 0.0348 | 6.57E-07 | 24.6708857 | 0.0239 | 0.3667 |
| rs4778640 | A | G | 0.7189 | 0.0983 | 2.552E-13 | 53.4543648 | 0.0675 | 0.5324 |
| rs4976691 | C | G | 0.1254 | 0.026 | 1.473E-06 | 23.2488577 | 0.018 | 0.2956 |
| rs7097884 | T | C | -0.1193 | 0.0243 | 8.809E-07 | 24.0891559 | 0.017 | 0.4821 |
| rs78042619 | A | G | 0.55 | 0.1158 | 2.023E-06 | 22.5455847 | 0.0749 | 0.6421 |
| **IL-17** | | | | | | | | |
| rs11985957 | A | G | 0.1511 | 0.0329 | 4.363E-06 | 21.0875351 | 0.0363 | 0.3591 |
| rs12735700 | T | G | -0.0943 | 0.0206 | 0.0000045 | 20.9496914 | 0.0206 | 0.7151 |
| rs145006174 | C | G | -0.2266 | 0.0473 | 1.645E-06 | 22.9449024 | 0.0482 | 0.8833 |
| rs17282552 | T | C | -0.2026 | 0.0403 | 4.876E-07 | 25.2672208 | 0.0383 | 0.7992 |
| rs3792369 | A | G | 0.0941 | 0.0166 | 1.46E-08 | 32.1256376 | 0.0173 | 0.0479999 |
| rs61990749 | C | G | 0.1124 | 0.0226 | 6.569E-07 | 24.728879 | 0.0234 | 0.1348 |
| rs78296352 | T | G | 0.2949 | 0.0645 | 4.809E-06 | 20.8986774 | 0.0721 | 0.2266 |
| rs9519328 | A | G | 0.5256 | 0.1101 | 0.00000179 | 22.7767495 | 0.0555 | 0.6783 |
| **IL-18** | | | | | | | | |
| rs10409850 | A | G | 0.1791 | 0.0347 | 2.44E-07 | 26.6253795 | 0.0248 | 0.2508 |
| rs11214093 | T | C | 0.1143 | 0.0238 | 1.544E-06 | 23.0516561 | 0.0169 | 0.6181 |
| rs117266781 | T | C | 0.7051 | 0.1436 | 9.176E-07 | 24.0966203 | 0.0916 | 0.4711 |
| rs117371668 | T | G | 0.3712 | 0.0799 | 3.357E-06 | 21.571779 | 0.0578 | 0.9691 |
| rs139468359 | T | C | 0.5101 | 0.1088 | 2.737E-06 | 21.9693112 | 0.076 | 0.593999 |
| rs1979967 | T | C | 0.14 | 0.0285 | 8.719E-07 | 24.1173694 | 0.0205 | 0.771401 |
| rs4952239 | A | T | -0.1156 | 0.0242 | 1.809E-06 | 22.8059686 | 0.0174 | 0.0140501 |
| rs58701153 | A | T | -0.1265 | 0.0242 | 1.807E-07 | 27.3095098 | 0.0176 | 0.01258 |
| rs62312914 | T | C | -0.1265 | 0.025 | 4.215E-07 | 25.5896661 | 0.0174 | 0.0750793 |
| rs764078 | A | T | 0.1283 | 0.0278 | 4.075E-06 | 21.2876296 | 0.0199 | 0.855 |
| rs77187209 | T | C | -0.4859 | 0.1041 | 3.082E-06 | 21.7748877 | 0.071 | 0.636 |
| rs78623212 | T | C | 0.8322 | 0.1676 | 6.82E-07 | 24.6417178 | 0.0954 | 7.97E-05 |
| rs78716465 | A | G | 0.3173 | 0.0679 | 2.981E-06 | 21.8254989 | 0.0454 | 0.9818 |
| **IP-10** | | | | | | | | |
| rs113183470 | A | T | -0.2414 | 0.0524 | 4.147E-06 | 21.2118552 | 0.0357 | 0.5623 |
| rs12714300 | A | T | -0.1573 | 0.0338 | 3.297E-06 | 21.6466523 | 0.0241 | 0.1382 |
| rs143799975 | A | G | -0.7551 | 0.1638 | 4.012E-06 | 21.2396738 | 0.1027 | 0.8091 |
| rs34383175 | T | C | -0.3196 | 0.0653 | 9.904E-07 | 23.9416388 | 0.0466 | 0.759299 |
| rs397816 | T | C | 0.1211 | 0.0248 | 1.026E-06 | 23.8315133 | 0.0175 | 0.5628 |
| rs4859940 | C | G | -0.1204 | 0.0258 | 3.228E-06 | 21.7660819 | 0.0184 | 0.513499 |
| rs4862110 | T | C | -0.1453 | 0.0318 | 4.909E-06 | 20.8662153 | 0.0195 | 0.2713 |
| rs75970138 | A | G | -0.4845 | 0.1037 | 2.994E-06 | 21.8170861 | 0.0756 | 0.1366 |
| rs7645625 | T | G | -0.1116 | 0.0236 | 2.192E-06 | 22.3496682 | 0.0172 | 0.4101 |
| rs78077394 | T | C | -0.3486 | 0.0701 | 6.474E-07 | 24.716412 | 0.0462 | 0.2057 |
| rs79848609 | A | C | 0.2514 | 0.0535 | 2.637E-06 | 22.069357 | 0.039 | 0.146 |
| rs8112618 | A | G | 0.1388 | 0.0297 | 3.047E-06 | 21.8289442 | 0.0211 | 0.9656 |
| **M-CSF** | | | | | | | | |
| rs116274860 | T | G | 0.8262 | 0.1739 | 2.029E-06 | 22.553989 | 0.0751 | 0.3217 |
| rs116887628 | A | G | -0.2741 | 0.0598 | 4.626E-06 | 20.9926783 | 0.0331 | 0.1104 |
| rs117867915 | T | C | 0.5224 | 0.1096 | 1.874E-06 | 22.700602 | 0.0612 | 0.692901 |
| rs11963606 | C | G | -0.5353 | 0.117 | 4.731E-06 | 20.9158207 | 0.0673 | 0.1481 |
| rs12962919 | T | C | 0.3025 | 0.0659 | 4.394E-06 | 21.0538767 | 0.0334 | 0.1248 |
| rs139457375 | A | C | -0.4047 | 0.0854 | 2.142E-06 | 22.4389646 | 0.0483 | 0.8746 |
| rs147378920 | A | G | -0.6064 | 0.1318 | 4.177E-06 | 21.1514289 | 0.0588 | 0.0106299 |
| rs34089869 | T | C | 0.2194 | 0.0462 | 2.078E-06 | 22.5341636 | 0.027 | 0.0918502 |
| rs62294910 | A | G | 0.3472 | 0.0687 | 4.378E-07 | 25.5210246 | 0.0348 | 0.1239 |
| rs72723242 | T | G | -0.4969 | 0.1083 | 4.434E-06 | 21.0345501 | 0.0667 | 0.1719 |
| rs9387100 | T | C | -0.135 | 0.029 | 3.341E-06 | 21.6532798 | 0.017 | 0.3417 |
| rs9626985 | T | C | 0.2277 | 0.0496 | 4.482E-06 | 21.0578901 | 0.0286 | 0.7345 |
| **MCP-1** | | | | | | | | |
| rs111995966 | T | G | 0.1428 | 0.0309 | 3.788E-06 | 21.3518377 | 0.0316 | 0.551099 |
| rs11920996 | T | C | 0.1805 | 0.0376 | 1.604E-06 | 23.0395783 | 0.0398 | 0.0287303 |
| rs12062235 | T | G | 0.1477 | 0.032 | 3.836E-06 | 21.2988834 | 0.0345 | 0.4276 |
| rs143815843 | A | G | -0.2049 | 0.0447 | 4.609E-06 | 21.0070308 | 0.0492 | 0.757399 |
| rs16837903 | A | G | -0.1104 | 0.0238 | 3.352E-06 | 21.5119627 | 0.0248 | 0.2772 |
| rs2201150 | T | C | 0.0916 | 0.016 | 1.044E-08 | 32.7677623 | 0.0174 | 0.0356903 |
| rs2229593 | T | C | 0.2624 | 0.0405 | 9.246E-11 | 41.9675308 | 0.0431 | 0.955 |
| rs56212190 | T | C | 0.1799 | 0.0372 | 1.318E-06 | 23.3814936 | 0.0392 | 0.9498 |
| rs62245103 | T | G | 0.2433 | 0.0416 | 4.989E-09 | 34.1974213 | 0.0415 | 0.9623 |
| rs7197349 | A | G | 0.0971 | 0.0206 | 2.399E-06 | 22.2126218 | 0.0212 | 0.4847 |
| rs72705803 | A | G | -0.2188 | 0.047 | 3.222E-06 | 21.6667974 | 0.053 | 0.6516 |
| rs7978037 | A | T | 0.0746 | 0.016 | 3.043E-06 | 21.7336912 | 0.0173 | 0.4422 |
| rs79939301 | A | G | 0.1449 | 0.0255 | 1.356E-08 | 32.281389 | 0.0265 | 0.5573 |
| rs856100 | A | G | 0.0899 | 0.0191 | 2.545E-06 | 22.1487108 | 0.0205 | 0.4639 |
| rs862990 | T | C | -0.089 | 0.0183 | 1.153E-06 | 23.6468685 | 0.0194 | 0.7402 |
| rs9317045 | A | C | 0.1157 | 0.0235 | 8.425E-07 | 24.2340944 | 0.0238 | 0.9711 |
| **MCP-3** | | | | | | | | |
| rs10892381 | T | C | 0.2432 | 0.0473 | 2.693E-07 | 26.3884447 | 0.0179 | 0.733199 |
| rs117286643 | A | G | 0.6934 | 0.1474 | 2.542E-06 | 22.0892842 | 0.0574 | 0.1391 |
| rs28394764 | A | T | 0.597 | 0.1282 | 3.194E-06 | 21.646194 | 0.0483 | 0.2769 |
| rs3129806 | T | C | -0.1975 | 0.0433 | 4.978E-06 | 20.766693 | 0.017 | 0.3314 |
| rs6993671 | T | C | 0.2041 | 0.0443 | 4.061E-06 | 21.1878794 | 0.0172 | 0.1824 |
| rs7275485 | T | C | -0.2218 | 0.0481 | 3.986E-06 | 21.2247149 | 0.0188 | 0.5203 |
| **MIF** | | | | | | | | |
| rs1007888 | T | C | -0.1275 | 0.0245 | 1.915E-07 | 27.0671345 | 0.0173 | 0.9493 |
| rs113218956 | A | G | -0.8789 | 0.1876 | 2.815E-06 | 21.9365002 | 0.1302 | 0.1134 |
| rs11551183 | C | G | 0.3666 | 0.0795 | 3.999E-06 | 21.2522416 | 0.0574 | 0.2345 |
| rs12594190 | A | G | 0.1321 | 0.0266 | 6.85E-07 | 24.6488375 | 0.0184 | 0.4884 |
| rs141009259 | T | C | -0.6194 | 0.1285 | 1.444E-06 | 23.2214977 | 0.0797 | 0.0581393 |
| rs2294689 | C | G | -0.1338 | 0.0287 | 3.043E-06 | 21.7221353 | 0.0696 | 0.9806 |
| rs35792361 | A | G | -0.2586 | 0.0527 | 9.003E-07 | 24.065223 | 0.0359 | 0.3173 |
| rs35890933 | T | G | 0.1676 | 0.0365 | 4.458E-06 | 21.0725154 | 0.024 | 0.4421 |
| rs3814097 | A | G | -0.1163 | 0.0251 | 3.548E-06 | 21.456855 | 0.0171 | 0.8194 |
| rs78098071 | T | C | -0.4583 | 0.0915 | 5.509E-07 | 25.0733063 | 0.0656 | 0.624499 |
| **MIG** |  |  |  |  |  |  |  |  |
| rs10266753 | T | C | -0.2016 | 0.0397 | 3.769E-07 | 25.7731047 | 0.0302 | 0.4546 |
| rs111607343 | A | G | -0.5235 | 0.1119 | 2.928E-06 | 21.8745887 | 0.0723 | 0.3251 |
| rs11177248 | A | G | 0.3157 | 0.0667 | 2.222E-06 | 22.3905207 | 0.0434 | 0.8308 |
| rs113302091 | T | C | 0.2537 | 0.0553 | 4.402E-06 | 21.0357194 | 0.04 | 0.0245002 |
| rs13143163 | C | G | 0.2735 | 0.0582 | 2.622E-06 | 22.0716799 | 0.0369 | 0.3738 |
| rs139010077 | T | C | 0.4337 | 0.0943 | 4.193E-06 | 21.1408359 | 0.065 | 0.7338 |
| rs191555775 | A | T | 0.2279 | 0.0412 | 3.278E-08 | 30.5816501 | 0.028 | 0.7366 |
| rs192433162 | A | G | -0.8045 | 0.1676 | 1.594E-06 | 23.0287712 | 0.1128 | 0.2944 |
| rs3733233 | T | C | 0.1223 | 0.025 | 1.049E-06 | 23.9188113 | 0.0176 | 0.6656 |
| rs62562991 | A | G | 0.6239 | 0.1259 | 7.237E-07 | 24.5439924 | 0.0711 | 0.1349 |
| rs6679677 | A | C | 0.1628 | 0.0327 | 6.514E-07 | 24.7730793 | 0.0237 | 0.8046 |
| rs8127917 | T | G | 0.2382 | 0.0492 | 1.278E-06 | 23.4271795 | 0.0354 | 0.824 |
| rs816960 | T | C | -0.1179 | 0.0242 | 0.00000115 | 23.7226704 | 0.0175 | 0.1731 |
| **MIP-1A** | | | | | | | | |
| rs117506943 | T | C | 0.0108 | 0.0682 | 4.484E-06 | 21.0242617 | 0.0429 | 0.0791498 |
| rs12159394 | A | G | 0.0008 | 0.0366 | 3.112E-06 | 21.7655465 | 0.0264 | 0.3881 |
| rs57786342 | A | G | 0.0149 | 0.0283 | 8.909E-07 | 24.1108624 | 0.0223 | 0.5771 |
| rs6956239 | T | C | -0.0088 | 0.026 | 4.583E-06 | 20.9364595 | 0.0185 | 0.2551 |
| **MIP-1B** | | | | | | | | |
| rs111721971 | T | G | -0.227 | 0.047 | 1.387E-06 | 23.321215 | 0.0464 | 0.7322 |
| rs116237296 | A | G | 0.5284 | 0.1115 | 2.153E-06 | 22.4528284 | 0.1199 | 0.1901 |
| rs11651720 | T | C | -0.1189 | 0.0219 | 5.688E-08 | 29.4693566 | 0.0234 | 0.2007 |
| rs11716293 | C | G | 0.0986 | 0.0189 | 1.689E-07 | 27.2098029 | 0.0197 | 0.1046 |
| rs117657747 | A | G | 0.2089 | 0.0453 | 4.013E-06 | 21.2589533 | 0.0369 | 0.5361 |
| rs145526037 | T | G | -0.1863 | 0.0406 | 4.473E-06 | 21.0508102 | 0.0435 | 0.9859 |
| rs17138331 | A | G | -0.1434 | 0.0295 | 1.125E-06 | 23.623783 | 0.0304 | 0.9345 |
| rs17661219 | C | G | 0.0872 | 0.0172 | 3.888E-07 | 25.6963388 | 0.0179 | 0.2061 |
| rs2314809 | T | C | -0.0735 | 0.0157 | 2.904E-06 | 21.9114212 | 0.0168 | 0.5017 |
| rs2742396 | T | C | 0.1071 | 0.0168 | 1.832E-10 | 40.6308167 | 0.0172 | 0.0754501 |
| rs281728 | A | C | -0.079 | 0.0171 | 3.891E-06 | 21.3381683 | 0.0183 | 0.2584 |
| rs28393318 | A | G | -0.1076 | 0.0235 | 4.616E-06 | 20.9596484 | 0.0253 | 0.2263 |
| rs57893487 | C | G | -0.1075 | 0.0214 | 4.916E-07 | 25.2281007 | 0.0207 | 0.1375 |
| rs6802288 | A | G | -0.1623 | 0.0173 | 6.574E-21 | 87.9913554 | 0.0188 | 0.4774 |
| rs6806860 | A | C | -0.1008 | 0.0188 | 8.575E-08 | 28.7409116 | 0.0202 | 0.4004 |
| rs6908843 | A | G | 0.0997 | 0.0209 | 1.779E-06 | 22.7506033 | 0.0224 | 0.3663 |
| rs72791296 | T | C | 0.2364 | 0.0466 | 3.968E-07 | 25.7287262 | 0.0466 | 0.1221 |
| rs72799710 | T | C | -0.1037 | 0.0217 | 1.792E-06 | 22.8314355 | 0.0233 | 0.0297399 |
| rs74979864 | A | T | -0.3184 | 0.0613 | 2.025E-07 | 26.9724467 | 0.0644 | 0.9695 |
| rs76582507 | A | G | 0.3259 | 0.0676 | 1.421E-06 | 23.2364888 | 0.0942 | 0.1867 |
| rs772112 | A | T | -0.142 | 0.0216 | 5.007E-11 | 43.2080195 | 0.0215 | 0.156 |
| rs9916627 | T | C | -0.096 | 0.0198 | 1.244E-06 | 23.5021319 | 0.0214 | 0.549201 |
| **PDGF-BB** | | | | | | | | |
| rs10512952 | T | C | -0.2816 | 0.0587 | 1.636E-06 | 23.0083196 | 0.0622 | 0.4345 |
| rs116154010 | T | C | 0.3225 | 0.0662 | 1.113E-06 | 23.726805 | 0.073 | 0.763901 |
| rs11766649 | A | G | 0.0902 | 0.0196 | 3.964E-06 | 21.173699 | 0.0208 | 0.173 |
| rs12289510 | A | G | -0.0772 | 0.0158 | 1.001E-06 | 23.868011 | 0.0168 | 0.3525 |
| rs12615784 | T | C | -0.1003 | 0.0193 | 1.986E-07 | 27.0011991 | 0.0206 | 0.8585 |
| rs147862316 | T | C | 0.2279 | 0.0411 | 2.987E-08 | 30.7397838 | 0.0458 | 0.3371 |
| rs2643354 | A | G | 0.1251 | 0.0261 | 1.633E-06 | 22.9683294 | 0.0283 | 0.8699 |
| rs35859699 | A | G | -0.3854 | 0.0838 | 4.223E-06 | 21.146144 | 0.0802 | 0.1015 |
| rs62191444 | T | G | -0.112 | 0.0239 | 2.678E-06 | 21.9551317 | 0.0235 | 0.8652 |
| rs6756793 | T | C | 0.0876 | 0.0157 | 2.684E-08 | 31.1246668 | 0.017 | 0.7346 |
| rs6910518 | T | G | 0.0806 | 0.0162 | 6.005E-07 | 24.7477578 | 0.0173 | 0.00447095 |
| rs72972467 | C | G | -0.1616 | 0.0328 | 8.286E-07 | 24.2678235 | 0.0334 | 0.004797 |
| rs73162807 | A | C | -0.2313 | 0.0499 | 3.548E-06 | 21.4805787 | 0.0541 | 0.2492 |
| rs9924851 | C | G | 0.0767 | 0.0163 | 2.702E-06 | 22.1366206 | 0.0175 | 0.790999 |
| **RANTES** | | | | | | | | |
| rs10505135 | T | C | 0.035 | 0.0252 | 1.899E-07 | 27.2144071 | 0.0175 | 0.1889 |
| rs118096511 | T | C | -0.0676 | 0.0709 | 1.965E-06 | 22.6332093 | 0.0499 | 0.3744 |
| rs11873385 | A | G | 0.0096 | 0.0552 | 2.927E-06 | 21.8666762 | 0.0371 | 0.624001 |
| rs148526102 | T | C | -0.002 | 0.083 | 4.793E-06 | 20.9267695 | 0.0579 | 0.295 |
| rs2731672 | T | C | -0.0476 | 0.0272 | 4.827E-06 | 20.8378946 | 0.0193 | 0.1156 |
| rs4795087 | C | G | -0.0065 | 0.0312 | 1.629E-06 | 22.9160869 | 0.0218 | 0.8979 |
| rs62438851 | A | G | -0.024 | 0.0413 | 4.009E-06 | 21.241356 | 0.0279 | 0.7734 |
| rs7170339 | C | G | 0.0645 | 0.0904 | 2.187E-06 | 22.434053 | 0.0597 | 0.4722 |
| rs72793342 | A | G | -0.0251 | 0.0307 | 9.08E-07 | 24.0184348 | 0.0208 | 0.1984 |
| rs78050316 | A | C | -0.0279 | 0.0859 | 9.946E-07 | 23.9152113 | 0.0642 | 0.0516297 |
| **SCF** | | | | | | | | |
| rs10800449 | A | C | 0.0851 | 0.0179 | 1.962E-06 | 22.5968979 | 0.0186 | 0.1457 |
| rs11244035 | T | C | -0.1296 | 0.0279 | 3.501E-06 | 21.5723452 | 0.03 | 0.577301 |
| rs113127926 | A | C | 0.1974 | 0.0418 | 2.337E-06 | 22.2965412 | 0.0434 | 0.4488 |
| rs117721699 | C | G | -0.2392 | 0.0484 | 7.514E-07 | 24.4189729 | 0.0527 | 0.4405 |
| rs12345108 | T | C | -0.0772 | 0.0167 | 3.731E-06 | 21.3647307 | 0.0177 | 0.43 |
| rs13412535 | A | G | -0.1065 | 0.0213 | 5.586E-07 | 24.9940005 | 0.0211 | 0.272 |
| rs138538809 | T | C | -0.5788 | 0.1139 | 3.757E-07 | 25.8169755 | 0.1157 | 0.3051 |
| rs72678285 | A | T | 0.1062 | 0.0231 | 0.00000443 | 21.1310384 | 0.023 | 0.2599 |
| rs78666213 | T | G | -0.2845 | 0.0574 | 7.152E-07 | 24.5604614 | 0.0584 | 0.8369 |
| rs8045376 | A | G | -0.3126 | 0.068 | 4.268E-06 | 21.1278783 | 0.0743 | 0.3074 |
| **SCGF-B** | | | | | | | | |
| rs11111869 | A | G | 0.1621 | 0.0311 | 1.861E-07 | 27.1526211 | 0.0226 | 0.398 |
| rs112346514 | T | C | -0.3261 | 0.0703 | 3.543E-06 | 21.5058918 | 0.0438 | 0.1085 |
| rs1149926 | T | C | -0.3458 | 0.0749 | 3.917E-06 | 21.3035927 | 0.0538 | 0.0322099 |
| rs118003677 | T | C | -0.3654 | 0.0786 | 3.348E-06 | 21.6002363 | 0.0559 | 0.514801 |
| rs12118918 | A | G | -0.1631 | 0.035 | 3.208E-06 | 21.7039281 | 0.024 | 0.2782 |
| rs12480722 | T | C | 0.1654 | 0.0353 | 2.812E-06 | 21.9426011 | 0.025 | 0.8201 |
| rs13287050 | A | T | -0.121 | 0.0263 | 4.118E-06 | 21.1556197 | 0.0187 | 0.00630406 |
| rs13866 | T | C | -0.1647 | 0.028 | 3.773E-09 | 34.5810077 | 0.0193 | 0.9233 |
| rs139413256 | A | G | -0.5174 | 0.1076 | 1.532E-06 | 23.109727 | 0.0662 | 0.8401 |
| rs143829871 | T | C | -0.1866 | 0.0399 | 2.852E-06 | 21.8596898 | 0.0289 | 0.2341 |
| rs144724875 | T | C | 0.5381 | 0.0829 | 8.645E-11 | 42.1098245 | 0.0507 | 0.9215 |
| rs149009264 | A | G | 0.4551 | 0.0985 | 3.793E-06 | 21.3357404 | 0.069 | 0.1295 |
| rs150733161 | T | C | -0.5255 | 0.112 | 2.687E-06 | 22.0026963 | 0.0723 | 0.6732 |
| rs151194174 | A | G | 0.4536 | 0.0941 | 1.454E-06 | 23.2238038 | 0.044 | 0.7274 |
| rs264157 | A | G | 0.1079 | 0.0233 | 3.685E-06 | 21.4337201 | 0.0168 | 0.5247 |
| rs34911860 | A | G | -0.3674 | 0.0787 | 3.002E-06 | 21.7818793 | 0.0641 | 0.4038 |
| rs3817303 | T | G | 0.1362 | 0.0294 | 3.602E-06 | 21.4499391 | 0.0213 | 0.1698 |
| rs4737731 | T | C | 0.1146 | 0.0251 | 4.871E-06 | 20.8347503 | 0.0186 | 0.0422698 |
| rs4976691 | C | G | -0.1484 | 0.0253 | 4.438E-09 | 34.3869192 | 0.018 | 0.2956 |
| rs77954165 | T | C | 0.2631 | 0.0562 | 2.867E-06 | 21.9046124 | 0.0388 | 0.9237 |
| rs78217154 | T | C | 0.3942 | 0.0861 | 4.722E-06 | 20.9504543 | 0.0603 | 0.1324 |
| **SDF-1A** | | | | | | | | |
| rs10474392 | A | G | 0.0934 | 0.0177 | 1.376E-07 | 27.8378865 | 0.0925 | 0.7535 |
| rs10516368 | A | C | -0.4268 | 0.0883 | 1.356E-06 | 23.3569674 | 0.0797 | 0.611699 |
| rs12141941 | T | C | -0.0881 | 0.0186 | 2.263E-06 | 22.4292612 | 0.0213 | 0.0580096 |
| rs149893336 | A | G | -0.494 | 0.1082 | 0.00000493 | 20.8395539 | 0.0192 | 0.0364704 |
| rs1600396 | A | G | -0.0933 | 0.0204 | 4.939E-06 | 20.9118278 | 0.0269 | 0.9843 |
| rs62194946 | T | G | -0.0849 | 0.0185 | 4.552E-06 | 21.0552772 | 0.1009 | 0.2036 |
| rs6586903 | T | C | -0.1264 | 0.0268 | 2.421E-06 | 22.2389153 | 0.0181 | 0.582101 |
| rs76766406 | A | G | 0.4642 | 0.1012 | 4.489E-06 | 21.0347952 | 0.0925 | 0.7535 |
| rs78883416 | C | G | -0.0871 | 0.0182 | 1.755E-06 | 22.8972104 | 0.0797 | 0.611699 |
| **TNF-A** | | | | | | | | |
| rs10767536 | A | G | 0.118 | 0.0253 | 3.146E-06 | 21.7407357 | 0.0178 | 0.7048 |
| rs115018697 | C | G | -0.9542 | 0.197 | 1.273E-06 | 23.4475589 | 0.1294 | 0.8557 |
| rs116736594 | T | C | 0.3407 | 0.0702 | 1.223E-06 | 23.5408048 | 0.0457 | 0.6753 |
| rs79105320 | A | G | 0.5573 | 0.1177 | 2.207E-06 | 22.4066239 | 0.081 | 0.1063 |
| **TNF-B** | | | | | | | | |
| rs10925040 | T | C | 0.1738 | 0.0372 | 2.929E-06 | 21.8001085 | 0.0175 | 0.9411 |
| rs2420873 | T | G | 0.1673 | 0.0365 | 4.513E-06 | 20.9822059 | 0.0172 | 0.424 |
| rs62284710 | A | G | 0.3702 | 0.0782 | 2.183E-06 | 22.3822794 | 0.0354 | 0.2551 |
| rs75240021 | C | G | 0.3713 | 0.0772 | 1.489E-06 | 23.1025697 | 0.0321 | 0.7905 |
| rs76225863 | A | G | 0.7534 | 0.1217 | 5.982E-10 | 38.2749883 | 0.0533 | 0.0400996 |
| **TRAIL** | | | | | | | | |
| rs113057689 | A | G | -0.2625 | 0.0489 | 7.972E-08 | 28.8094751 | 0.0406 | 0.1988 |
| rs11875481 | T | C | -0.0969 | 0.0211 | 4.601E-06 | 21.0851692 | 0.0227 | 0.5913 |
| rs12458564 | A | T | -0.1002 | 0.0175 | 1.1E-08 | 32.7758372 | 0.0186 | 0.3676 |
| rs13115587 | A | C | 0.101 | 0.0217 | 3.244E-06 | 21.657969 | 0.0239 | 0.3529 |
| rs13278062 | T | G | 0.08 | 0.0157 | 3.326E-07 | 25.9582324 | 0.0168 | 0.9992 |
| rs139958028 | A | G | 0.1803 | 0.0395 | 4.992E-06 | 20.830117 | 0.0402 | 0.9986 |
| rs183815186 | A | T | -0.3499 | 0.0602 | 6.341E-09 | 33.7745328 | 0.062 | 0.0304698 |
| rs550057 | T | C | -0.0783 | 0.0169 | 3.707E-06 | 21.4607336 | 0.0183 | 0.0113999 |
| rs558572 | T | C | 0.1351 | 0.0265 | 3.419E-07 | 25.9844422 | 0.0284 | 0.1712 |
| rs57396456 | T | C | -0.5641 | 0.0516 | 7.71E-28 | 119.48331 | 0.0558 | 0.529 |
| rs616114 | T | C | -0.1033 | 0.0162 | 1.715E-10 | 40.6504223 | 0.0173 | 0.0984895 |
| rs62093482 | T | C | 0.9827 | 0.0529 | 6.123E-77 | 345.004703 | 0.0586 | 0.4169 |
| rs747324 | T | C | -0.0826 | 0.0178 | 3.338E-06 | 21.528538 | 0.0187 | 0.572 |
| rs75928541 | A | G | 0.2784 | 0.0591 | 2.442E-06 | 22.1849241 | 0.057 | 0.9396 |
| rs7599203 | T | C | 0.0918 | 0.02 | 4.333E-06 | 21.0629802 | 0.0215 | 0.9441 |
| rs78682108 | A | G | -0.2383 | 0.0394 | 1.435E-09 | 36.572116 | 0.0386 | 0.661899 |
| **VEGF** | | | | | | | | |
| rs10411345 | C | G | -0.1041 | 0.0218 | 1.733E-06 | 22.7964477 | 0.0201 | 0.3627 |
| rs10757514 | C | G | -0.1024 | 0.0222 | 4.169E-06 | 21.2702537 | 0.022 | 0.4109 |
| rs10822118 | T | C | -0.0797 | 0.0168 | 2.211E-06 | 22.499703 | 0.0168 | 0.00197102 |
| rs10934631 | T | C | -0.1132 | 0.0244 | 3.607E-06 | 21.5175047 | 0.0238 | 0.720701 |
| rs114773511 | T | C | 0.2187 | 0.0441 | 6.971E-07 | 24.5866349 | 0.0413 | 0.17 |
| rs12456390 | T | C | -0.0818 | 0.0179 | 4.882E-06 | 20.8775365 | 0.0181 | 0.467 |
| rs1730969 | C | G | -0.7811 | 0.1696 | 4.106E-06 | 21.2027728 | 0.1979 | 0.7552 |
| rs181031888 | A | T | 0.3767 | 0.0498 | 4.014E-14 | 57.2020062 | 0.0463 | 0.745901 |
| rs2039420 | C | G | 0.0975 | 0.0175 | 2.744E-08 | 31.0321481 | 0.0175 | 0.9833 |
| rs56071907 | T | C | 0.126 | 0.027 | 0.00000301 | 21.7716963 | 0.0242 | 0.375 |
| rs60013354 | A | G | -0.2497 | 0.0521 | 1.662E-06 | 22.9636196 | 0.0506 | 0.8911 |
| rs62401205 | A | C | -0.2015 | 0.0412 | 9.946E-07 | 23.9130171 | 0.0341 | 0.7965 |
| rs6496613 | A | C | -0.2359 | 0.0515 | 4.701E-06 | 20.9758771 | 0.0516 | 0.8991 |
| rs7356919 | A | G | -0.1694 | 0.02 | 2.846E-17 | 71.7208662 | 0.0196 | 0.3671 |
| rs73872715 | T | C | -0.6079 | 0.1299 | 2.864E-06 | 21.8939787 | 0.113 | 0.2084 |
| rs76458389 | T | G | -0.1786 | 0.0363 | 8.573E-07 | 24.2007243 | 0.0359 | 0.9679 |
| rs77961527 | A | G | 0.2289 | 0.0457 | 5.525E-07 | 25.0805982 | 0.0396 | 0.4616 |
| rs9381249 | T | C | -0.2414 | 0.0396 | 1.038E-09 | 37.1503462 | 0.0416 | 0.3741 |

**Table S8. MR estimates of 41 inflammatory cytokines on DR.**

|  | **IVW** | | | | **MR-Egger** | | | | **Weighted Median** | | | | **Simple Mode** | | | | **Weighted Mode** | | | |
| --- | --- | --- | --- | --- | --- | --- | --- | --- | --- | --- | --- | --- | --- | --- | --- | --- | --- | --- | --- | --- |
| **Exposures** | **No.of SNPs** | **OR** | **95% CI** | **pval** | **No.of SNPs** | **OR** | **95% CI** | **pval** | **No.of SNPs** | **OR** | **95% CI** | **pval** | **No.of SNPs** | **OR** | **95% CI** | **pval** | **No.of SNPs** | **OR** | **95% CI** | **pval** |
| CTACK | 12 | 1.080 | (0.936-1.245) | 0.292 | 12 | 1.152 | (0.860-1.543) | 0.367 | 12 | 1.078 | (0.899-1.293) | 0.417 | 12 | 1.189 | (0.845-1.672) | 0.341 | 12 | 1.102 | (0.789-1.539) | 0.581 |
| Eotaxin | 17 | 0.973 | (0.850-1.115) | 0.697 | 17 | 1.001 | (0.719-1.394) | 0.993 | 17 | 1.025 | (0.861-1.221) | 0.781 | 17 | 0.950 | (0.687-1.313) | 0.759 | 17 | 1.018 | (0.791-1.309) | 0.893 |
| GROa | 13 | 0.980 | (0.846-1.135) | 0.783 | 13 | 1.145 | (0.896-1.465) | 0.303 | 13 | 0.999 | (0.847-1.178) | 0.989 | 13 | 1.010 | (0.764-1.334) | 0.948 | 13 | 1.048 | (0.813-1.351) | 0.724 |
| IP-10 | 12 | 0.966 | (0.854-1.093) | 0.584 | 12 | 1.105 | (0.834-1.463) | 0.502 | 12 | 1.000 | (0.849-1.178) | 0.998 | 12 | 0.995 | (0.755-1.312) | 0.974 | 12 | 1.007 | (0.770-1.318) | 0.960 |
| MCP-1 | 16 | 1.026 | (0.880-1.195) | 0.744 | 16 | 1.150 | (0.754-1.756) | 0.526 | 16 | 1.053 | (0.862-1.287) | 0.614 | 16 | 1.067 | (0.765-1.488) | 0.709 | 16 | 1.060 | (0.776-1.449) | 0.719 |
| MCP-3 | 6 | 1.035 | (0.939-1.141) | 0.493 | 6 | 0.932 | (0.721-1.206) | 0.622 | 6 | 1.031 | (0.907-1.172) | 0.640 | 6 | 1.028 | (0.851-1.242) | 0.784 | 6 | 1.001 | (0.835-1.201) | 0.988 |
| MIG | 13 | 1.137 | (0.824-1.567) | 0.435 | 13 | 1.003 | (0.500-2.010) | 0.994 | 13 | 0.930 | (0.796-1.087) | 0.363 | 13 | 0.922 | (0.740-1.149) | 0.486 | 13 | 0.913 | (0.739-1.128) | 0.416 |
| MIP-1A | 4 | 0.895 | (0.637-1.259) | 0.525 | 4 | 1.306 | (0.402-4.246) | 0.700 | 4 | 0.935 | (0.697-1.253) | 0.652 | 4 | 0.969 | (0.601-1.562) | 0.905 | 4 | 0.982 | (0.641-1.505) | 0.939 |
| MIP-1B | 22 | 0.971 | (0.859-1.097) | 0.633 | 22 | 0.886 | (0.628-1.250) | 0.499 | 22 | 0.946 | (0.797-1.123) | 0.526 | 22 | 1.096 | (0.805-1.493) | 0.565 | 22 | 0.937 | (0.723-1.214) | 0.629 |
| RANTES | 10 | 1.005 | (0.833-1.214) | 0.955 | 10 | 1.034 | (0.629-1.699) | 0.900 | 10 | 0.979 | (0.813-1.178) | 0.819 | 10 | 0.960 | (0.693-1.331) | 0.813 | 10 | 0.964 | (0.718-1.294) | 0.813 |
| SDF-1A | 9 | 0.916 | (0.691-1.215) | 0.544 | 9 | 0.975 | (0.539-1.762) | 0.935 | 9 | 0.781 | (0.582-1.047) | 0.098 | 9 | 0.703 | (0.424-1.165) | 0.209 | 9 | 0.723 | (0.472-1.109) | 0.176 |
| SCGF-B | 21 | 1.010 | (0.913-1.118) | 0.845 | 21 | 0.971 | (0.782-1.205) | 0.790 | 21 | 0.947 | (0.830-1.081) | 0.421 | 21 | 0.944 | (0.739-1.205) | 0.648 | 21 | 0.925 | (0.758-1.130) | 0.454 |
| B-NGF | 4 | 1.044 | (0.852-1.278) | 0.680 | 4 | 0.983 | (0.273-3.536) | 0.981 | 4 | 1.082 | (0.835-1.403) | 0.552 | 4 | 1.208 | (0.833-1.753) | 0.393 | 4 | 1.208 | (0.801-1.823) | 0.434 |
| FGF2 | 7 | 0.954 | (0.754-1.206) | 0.693 | 7 | 1.619 | (0.814-3.218) | 0.228 | 7 | 0.901 | (0.661-1.226) | 0.506 | 7 | 0.882 | (0.550-1.414) | 0.621 | 7 | 0.875 | (0.555-1.379) | 0.585 |
| G-CSF | 9 | 1.137 | (0.935-1.382) | 0.198 | 9 | 1.316 | (0.940-1.842) | 0.154 | 9 | 1.222 | (0.948-1.574) | 0.122 | 9 | 1.235 | (0.865-1.763) | 0.279 | 9 | 1.235 | (0.896-1.701) | 0.233 |
| HGF | 9 | 1.012 | (0.796-1.288) | 0.922 | 9 | 0.707 | (0.436-1.147) | 0.203 | 9 | 0.834 | (0.625-1.114) | 0.219 | 9 | 0.819 | (0.478-1.404) | 0.489 | 9 | 0.808 | (0.550-1.187) | 0.309 |
| M-CSF | 12 | 1.019 | (0.926-1.122) | 0.701 | 12 | 1.006 | (0.823-1.229) | 0.958 | 12 | 1.015 | (0.893-1.154) | 0.820 | 12 | 0.937 | (0.734-1.197) | 0.612 | 12 | 0.983 | (0.795-1.216) | 0.877 |
| PDGF-BB | 14 | 1.026 | (0.834-1.261) | 0.810 | 14 | 1.421 | (0.891-2.266) | 0.166 | 14 | 0.968 | (0.762-1.231) | 0.792 | 14 | 0.960 | (0.600-1.536) | 0.867 | 14 | 0.969 | (0.617-1.522) | 0.894 |
| SCF | 10 | 0.964 | (0.765-1.215) | 0.758 | 10 | 1.580 | (1.044-2.391) | 0.063 | 10 | 1.028 | (0.778-1.359) | 0.847 | 10 | 1.065 | (0.673-1.684) | 0.795 | 10 | 1.045 | (0.685-1.595) | 0.842 |
| VEGF | 18 | 1.043 | (0.921-1.181) | 0.508 | 18 | 1.285 | (0.982-1.682) | 0.086 | 18 | 1.024 | (0.857-1.224) | 0.793 | 18 | 0.845 | (0.597-1.197) | 0.357 | 18 | 1.060 | (0.798-1.407) | 0.692 |
| IL-10 | 15 | 0.871 | (0.759-0.999) | 0.048 | 15 | 0.930 | (0.596-1.451) | 0.755 | 15 | 0.858 | (0.708-1.040) | 0.119 | 15 | 0.810 | (0.602-1.089) | 0.185 | 15 | 0.852 | (0.690-1.053) | 0.160 |
| IL-12-p70 | 15 | 1.006 | (0.859-1.177) | 0.946 | 15 | 1.513 | (0.857-2.670) | 0.177 | 15 | 0.993 | (0.779-1.236) | 0.953 | 15 | 0.920 | (0.626-1.354) | 0.697 | 15 | 0.911 | (0.616-1.347) | 0.647 |
| IL-13 | 14 | 1.001 | (0.902-1.110） | 0.989 | 14 | 1.075 | (0.885-1.305） | 0.481 | 14 | 1.041 | (0.901-1.204) | 0.584 | 14 | 1.100 | (0.837-1.444) | 0.507 | 14 | 1.076 | (0.859-1.348） | 0.536 |
| IL-16 | 10 | 0.917 | (0.819-1.027) | 0.134 | 10 | 1.060 | (0.882-1.274) | 0.553 | 10 | 0.920 | (0.789-1.073) | 0.289 | 10 | 0.895 | (0.702-1.141) | 0.392 | 10 | 0.952 | (0.755-1.201) | 0.689 |
| IL-17 | 8 | 0.841 | (0.664-1.065) | 0.151 | 8 | 0.894 | (0.560-1.427) | 0.655 | 8 | 0.866 | (0.665-1.130) | 0.290 | 8 | 0.774 | (0.513-1.168) | 0.262 | 8 | 0.845 | (0.636-1.124) | 0.286 |
| IL-18 | 13 | 0.918 | (0.796-1.058) | 0.237 | 13 | 0.918 | (0.688-1.224) | 0.571 | 13 | 0.838 | (0.707-0.993) | 0.041 | 13 | 0.801 | (0.606-1.059) | 0.145 | 13 | 0.804 | (0.609-1.061) | 0.149 |
| IL-1B | 3 | 0.982 | (0.700-1.376) | 0.915 | 3 | 0.703 | (0.391-1.264) | 0.448 | 3 | 1.025 | (0.734-1.430) | 0.884 | 3 | 1.010 | (0.652-1.564) | 0.968 | 3 | 1.057 | (0.731-1.529) | 0.767 |
| IL-1RA | 10 | 1.071 | (0.933-1.229) | 0.333 | 10 | 0.940 | (0.636-1.391) | 0.767 | 10 | 0.961 | (0.800-1.155) | 0.671 | 10 | 0.939 | (0.733-1.203) | 0.631 | 10 | 0.939 | (0.738-1.195) | 0.622 |
| IL-2 | 8 | 1.027 | (0.896-1.176) | 0.703 | 8 | 1.039 | (0.794-1.361) | 0.789 | 8 | 0.985 | (0.824-1.177) | 0.864 | 8 | 0.909 | (0.691-1.194) | 0.513 | 8 | 0.933 | (0.730-1.194) | 0.601 |
| IL-2RA | 9 | 0.905 | (0.770-1.064) | 0.227 | 9 | 0.774 | (0.580-1.033) | 0.126 | 9 | 0.961 | (0.803-1.150) | 0.661 | 9 | 1.076 | (0.781-1.483) | 0.667 | 9 | 1.062 | (0.759-1.485) | 0.735 |
| IL-4 | 14 | 1.035 | (0.874-1.224) | 0.691 | 14 | 0.957 | (0.672-1.364) | 0.812 | 14 | 1.125 | (0.895-1.413) | 0.312 | 14 | 1.144 | (0.792-1.654) | 0.486 | 14 | 1.144 | (0.785-1.668) | 0.495 |
| IL-5 | 8 | 0.945 | (0.820-1.090) | 0.439 | 8 | 1.119 | (0.821-1.525) | 0.504 | 8 | 1.054 | (0.862-1.288) | 0.609 | 8 | 1.072 | (0.757-1.518) | 0.706 | 8 | 1.083 | (0.808-1.450) | 0.610 |
| IL-6 | 11 | 0.922 | (0.762-1.116) | 0.407 | 11 | 0.872 | (0.569-1.336) | 0.545 | 11 | 0.916 | (0.714-1.176) | 0.491 | 11 | 0.933 | (0.633-1.375) | 0.732 | 11 | 0.930 | (0.639-1.354) | 0.714 |
| IL-7 | 12 | 0.985 | (0.887-1.094) | 0.782 | 12 | 1.014 | (0.795-1.294) | 0.913 | 12 | 0.964 | (0.838-1.111) | 0.615 | 12 | 0.975 | (0.777-1.225) | 0.834 | 12 | 0.965 | (0.803-1.159) | 0.710 |
| IL-8 | 8 | 1.082 | (0.929-1.260) | 0.311 | 8 | 0.979 | (0.733-1.309) | 0.892 | 8 | 1.185 | (0.980-1.433) | 0.081 | 8 | 1.233 | (0.914-1.665) | 0.213 | 8 | 1.219 | (0.899-1.653) | 0.243 |
| IL-9 | 6 | 0.904 | (0.749-1.092) | 0.296 | 6 | 0.761 | (0.481-1.202) | 0.306 | 6 | 0.818 | (0.644-1.038) | 0.099 | 6 | 0.804 | (0.556-1.162) | 0.298 | 6 | 0.799 | (0.574-1.113) | 0.242 |
| IFN-G | 12 | 1.056 | (0.879-1.268) | 0.562 | 12 | 0.977 | (0.662-1.442) | 0.911 | 12 | 1.195 | (0.946-1.508) | 0.135 | 12 | 1.288 | (0.904-1.837) | 0.189 | 12 | 1.249 | (0.922-1.691) | 0.179 |
| MIF | 10 | 1.078 | (0.928-1.253） | 0.325 | 10 | 1.057 | (0.772-1.447) | 0.737 | 10 | 1.067 | (0.882-1.292) | 0.505 | 10 | 0.959 | (0.733-1.253) | 0.765 | 10 | 0.967 | (0.742-1.261) | 0.811 |
| TNF-A | 4 | 0.955 | (0.777-1.174) | 0.660 | 4 | 0.967 | (0.699-1.337) | 0.856 | 4 | 0.973 | (0.772-1.228) | 0.819 | 4 | 1.025 | (0.736-1.429) | 0.891 | 4 | 1.033 | (0.726-1.468) | 0.870 |
| TNF-B | 5 | 0.936 | (0.832-1.052) | 0.267 | 5 | 0.985 | (0.776-1.250) | 0.909 | 5 | 0.951 | (0.826-1.095) | 0.483 | 5 | 0.872 | (0.706-1.076) | 0.270 | 5 | 0.999 | (0.822-1.214) | 0.990 |
| TRAIL | 16 | 0.925 | (0.814-1.052) | 0.237 | 16 | 1.002 | (0.832-1.207) | 0.981 | 16 | 0.910 | (0.776-1.067) | 0.247 | 16 | 0.913 | (0.714-1.167) | 0.477 | 16 | 0.930 | (0.792-1.091) | 0.384 |

Abbreviations: DR, diabetic retinopathy; CI, Confidence interval; OR, Odds Ratio; pval, P-value; SNPs, single nucleotide polymorphisms. OR and 95% CI represent the change in the odds ratio of DR per 1 SD increase in inflammatory cytokines.

**Table S9. Heterogeneity and horizontal pleiotropy tests of 41 inflammatory cytokines on DR.**

| **Exposures** | **Q_1_ pval (IVW)** | **Q_2_ pval (MR-Egger)** | **I^2^** | **intercept** | **intercept pval** |
| --- | --- | --- | --- | --- | --- |
| B-NGF | 0.387 | 0.221 | 1.0% | 0.009 | 0.934 |
| CTACK | 0.222 | 0.180 | 22.6% | -0.016 | 0.628 |
| Eotaxin | 0.952 | 0.929 | 0.0% | -0.004 | 0.856 |
| FGF2 | 0.609 | 0.859 | 0.0% | -0.070 | 0.169 |
| G-CSF | 0.705 | 0.734 | 0.0% | -0.023 | 0.330 |
| GROa | 0.054 | 0.102 | 42.3% | -0.047 | 0.158 |
| HGF | 0.211 | 0.346 | 26.2% | 0.051 | 0.147 |
| IFN-G | 0.306 | 0.250 | 14.1% | 0.011 | 0.666 |
| IL-1B | 0.204 | 0.274 | 37.0% | 0.069 | 0.420 |
| IL-1RA | 0.460 | 0.409 | 0.0% | 0.021 | 0.507 |
| IL-2 | 0.661 | 0.546 | 0.0% | -0.002 | 0.922 |
| IL-2RA | 0.089 | 0.131 | 41.7% | 0.035 | 0.247 |
| IL-4 | 0.485 | 0.423 | 0.0% | 0.011 | 0.631 |
| IL-5 | 0.440 | 0.487 | 0.0% | -0.036 | 0.275 |
| IL-6 | 0.860 | 0.803 | 0.0% | 0.007 | 0.779 |
| IL-7 | 0.601 | 0.517 | 0.0% | -0.006 | 0.803 |
| IL-8 | 0.388 | 0.351 | 5.4% | 0.021 | 0.454 |
| IL-9 | 0.649 | 0.615 | 0.0% | 0.043 | 0.461 |
| IL-10 | 0.944 | 0.920 | 0.0% | -0.008 | 0.765 |
| IL-12p70 | 0.815 | 0.897 | 0.0% | -0.044 | 0.166 |
| IL-13 | 0.539 | 0.517 | 0.0% | -0.018 | 0.411 |
| IL-16 | 0.455 | 0.757 | 0.0% | -0.046 | 0.087 |
| IL-17 | 0.127 | 0.085 | 38.0% | -0.011 | 0.771 |
| IL-18 | 0.101 | 0.071 | 35.1% | -4.98E-05 | 0.998 |
| IP-10 | 0.815 | 0.839 | 0.0% | -0.026 | 0.321 |
| M-CSF | 0.526 | 0.438 | 0.0% | 0.005 | 0.886 |
| MCP-1 | 0.827 | 0.794 | 0.0% | -0.015 | 0.578 |
| MCP-3 | 0.472 | 0.430 | 0.0% | 0.029 | 0.440 |
| MIF | 0.305 | 0.228 | 14.9% | 0.004 | 0.890 |
| MIG | 2.92E-15 | 1.80E-15 | 87.5% | 0.031 | 0.695 |
| MIP-1A | 0.068 | 0.054 | 58.0% | -0.064 | 0.575 |
| MIP-1B | 0.554 | 0.510 | 0.0% | 0.012 | 0.584 |
| PDGF-BB | 0.109 | 0.174 | 33.3% | -0.041 | 0.157 |
| RANTES | 0.037 | 0.022 | 49.7% | -0.006 | 0.908 |
| SCF | 0.205 | 0.752 | 25.9% | -0.073 | 0.029 |
| SCGF-B | 0.139 | 0.113 | 25.6% | 0.009 | 0.684 |
| SDF-1A | 0.043 | 0.027 | 49.8% | -0.008 | 0.820 |
| TNF-A | 0.822 | 0.636 | 0.0% | -0.004 | 0.932 |
| TNF-B | 0.769 | 0.663 | 0.0% | -0.016 | 0.661 |
| TRAIL | 0.131 | 0.152 | 29.2% | -0.018 | 0.270 |
| VEGF | 0.537 | 0.683 | 0.0% | -0.035 | 0.105 |

Q1 pval: P-value of Q test from IVW method; Q2 pval: P-value of Q test from MR-Egger method.

Abbreviations: DR, diabetic retinopathy; pval, P-value; Q, Cochran Q statistics; SNPs, single nucleotide polymorphisms; IVW, the inverse variance weighted method.

**Table S10. SNPs information of 41 inflammation cytokines with DR.**

|  | **Inflammatory cytokines (exposure)** | | | | | **DR (outcome)** | | |
| --- | --- | --- | --- | --- | --- | --- | --- | --- |
| **SNP** | **effect allele** | **other allele** | **Beta** | **se** | **pval** | **F** | **se** | **pval** |
| **B-NGF** | | | | | | | | |
| rs28637706 | T | G | -0.1554 | 0.0261 | 2.717E-09 | 35.4305296 | 0.0273 | 0.2856 |
| rs4767014 | T | C | -0.1211 | 0.0264 | 4.518E-06 | 21.029893 | 0.0268 | 0.8244 |
| rs71641308 | T | C | 0.1969 | 0.0429 | 4.424E-06 | 21.0539447 | 0.0429 | 0.2641 |
| rs73472576 | T | C | -0.1146 | 0.0251 | 4.813E-06 | 20.8342764 | 0.0254 | 0.3824 |
| **CTACK** | | | | | | | | |
| rs10854859 | A | G | -0.1498 | 0.0293 | 3.049E-07 | 26.1247203 | 0.0302 | 0.1603 |
| rs116303454 | A | G | 0.3754 | 0.081 | 3.579E-06 | 21.4675143 | 0.0785 | 0.765301 |
| rs116871507 | A | T | -0.2086 | 0.0448 | 0.00000316 | 21.668849 | 0.0493 | 0.28 |
| rs116943377 | A | G | 0.2878 | 0.0611 | 2.496E-06 | 22.1749384 | 0.0652 | 0.1167 |
| rs117932939 | T | C | 0.1969 | 0.0422 | 3.089E-06 | 21.7585421 | 0.0454 | 0.166 |
| rs118084576 | A | G | 0.5675 | 0.1226 | 0.00000366 | 21.4148015 | 0.1427 | 0.4197 |
| rs184329319 | T | G | -0.3069 | 0.0648 | 2.173E-06 | 22.4185246 | 0.0708 | 0.3355 |
| rs55764737 | T | C | 0.5424 | 0.0967 | 2.012E-08 | 31.4448608 | 0.1056 | 0.7993 |
| rs57338032 | A | G | 0.1443 | 0.0316 | 4.831E-06 | 20.8411539 | 0.0336 | 0.1578 |
| rs57789542 | T | C | -0.7687 | 0.1659 | 3.575E-06 | 21.4577535 | 0.1522 | 0.623799 |
| rs60247384 | T | C | 0.1128 | 0.0245 | 4.302E-06 | 21.1860159 | 0.0263 | 0.7453 |
| rs76395525 | A | G | 0.5193 | 0.1081 | 1.553E-06 | 23.0647377 | 0.127 | 0.04385 |
| **Eotaxin** | | | | | | | | |
| rs11087905 | A | C | 0.0954 | 0.0188 | 4.07E-07 | 25.7439435 | 0.0267 | 0.7146 |
| rs112347425 | T | C | 0.1595 | 0.0276 | 7.771E-09 | 33.388525 | 0.0439 | 0.6191 |
| rs11920996 | T | C | 0.2979 | 0.0377 | 2.919E-15 | 62.4241056 | 0.0595 | 0.8453 |
| rs1677588 | T | G | 0.1181 | 0.025 | 2.223E-06 | 22.310731 | 0.0394 | 0.896 |
| rs2024050 | A | G | 0.164 | 0.0302 | 5.467E-08 | 29.4827394 | 0.0488 | 0.7074 |
| rs2027855 | T | C | 0.0743 | 0.0162 | 4.272E-06 | 21.0301137 | 0.0257 | 0.614601 |
| rs2040143 | A | G | -0.0858 | 0.0178 | 1.333E-06 | 23.2288973 | 0.0281 | 0.7258 |
| rs2229593 | T | C | 0.3647 | 0.0406 | 2.838E-19 | 80.6702648 | 0.0645 | 0.8151 |
| rs2249581 | T | C | -0.0899 | 0.018 | 5.912E-07 | 24.9383891 | 0.0288 | 0.175 |
| rs5754733 | A | C | -0.105 | 0.0213 | 8.196E-07 | 24.2948048 | 0.0334 | 0.5553 |
| rs57723662 | C | G | -0.0982 | 0.0213 | 3.878E-06 | 21.2499441 | 0.0332 | 0.2625 |
| rs60075014 | T | C | -0.1688 | 0.0356 | 2.078E-06 | 22.4770293 | 0.0515 | 0.2816 |
| rs7231030 | A | C | 0.0903 | 0.0193 | 2.709E-06 | 21.885421 | 0.0305 | 0.7968 |
| rs73072941 | A | T | -0.1281 | 0.0263 | 1.113E-06 | 23.7181486 | 0.0392 | 0.9542 |
| rs745331 | A | G | -0.0821 | 0.0176 | 3.036E-06 | 21.7547953 | 0.0271 | 0.2738 |
| rs75426604 | A | C | -0.1371 | 0.0291 | 2.397E-06 | 22.1913107 | 0.0434 | 0.5534 |
| rs9317045 | A | C | 0.1172 | 0.0236 | 6.954E-07 | 24.6561487 | 0.0355 | 0.3707 |
| **FGF2** | | | | | | | | |
| rs13412535 | A | G | -0.1129 | 0.0224 | 4.763E-07 | 25.3967228 | 0.0316 | 0.4581 |
| rs147409637 | T | C | 0.201 | 0.0431 | 3.077E-06 | 21.7431973 | 0.0678 | 0.3211 |
| rs17094040 | T | C | 0.1051 | 0.0229 | 4.308E-06 | 21.0581541 | 0.0358 | 0.1749 |
| rs2849358 | A | G | 0.0911 | 0.0193 | 2.321E-06 | 22.2745002 | 0.029 | 0.9688 |
| rs4795091 | A | G | 0.1239 | 0.0266 | 3.055E-06 | 21.6902807 | 0.0403 | 0.7696 |
| rs76253061 | T | C | -0.4811 | 0.1041 | 3.807E-06 | 21.3528125 | 0.1544 | 0.3316 |
| rs78873483 | A | G | 0.1286 | 0.0282 | 0.00000498 | 20.7907211 | 0.0399 | 0.616799 |
| **G-CSF** | | | | | | | | |
| rs10939033 | A | G | -0.0775 | 0.0163 | 2.074E-06 | 22.6005443 | 0.0257 | 0.547299 |
| rs117261691 | T | C | 0.1318 | 0.0288 | 4.669E-06 | 20.9380652 | 0.0454 | 0.2922 |
| rs183023730 | T | G | 0.7898 | 0.1677 | 2.471E-06 | 22.1734096 | 0.1988 | 0.1841 |
| rs586802 | A | G | 0.0882 | 0.0187 | 2.398E-06 | 22.2405058 | 0.0293 | 0.2659 |
| rs6740648 | T | C | 0.0818 | 0.0172 | 0.0000019 | 22.6120749 | 0.0269 | 0.5381 |
| rs74148555 | T | C | -0.3771 | 0.0753 | 5.591E-07 | 25.0734338 | 0.0889 | 0.4084 |
| rs76287671 | T | C | 0.0894 | 0.0189 | 2.191E-06 | 22.3687714 | 0.0294 | 0.673701 |
| rs77318030 | T | C | -0.2031 | 0.0427 | 2.017E-06 | 22.61803121 | 0.0625 | 0.3814 |
| rs78523761 | A | G | 0.5374 | 0.1139 | 2.389E-6 | 22.25421208 | 0.1589 | 0.4186 |
| **GROa** | | | | | | | | |
| rs114991247 | T | C | -0.2202 | 0.0463 | 1.971E-06 | 22.6061961 | 0.0423 | 0.00201502 |
| rs115214168 | T | C | 0.4528 | 0.0828 | 4.479E-08 | 29.8886987 | 0.0873 | 0.582801 |
| rs1361829 | A | G | -0.1106 | 0.0241 | 4.576E-06 | 21.0489779 | 0.0253 | 0.2169 |
| rs140734053 | A | G | 0.7333 | 0.1545 | 2.069E-06 | 22.514445 | 0.1331 | 0.4769 |
| rs150194856 | T | C | -0.4223 | 0.0914 | 3.857E-06 | 21.3355879 | 0.0978 | 0.0503605 |
| rs17171245 | T | G | 0.2446 | 0.053 | 3.932E-06 | 21.287076 | 0.0587 | 0.9756 |
| rs185768063 | A | G | 0.4038 | 0.076 | 1.055E-07 | 28.2137159 | 0.0775 | 0.9821 |
| rs188345231 | T | C | 0.6177 | 0.1322 | 2.968E-06 | 21.8196176 | 0.1416 | 0.9282 |
| rs3026943 | A | C | -0.1246 | 0.0256 | 1.084E-06 | 23.6761454 | 0.0268 | 0.617701 |
| rs62024303 | A | G | -0.3013 | 0.066 | 4.908E-06 | 20.8288473 | 0.0622 | 0.2195 |
| rs76215157 | C | G | -0.7398 | 0.1564 | 2.226E-06 | 22.3619669 | 0.1248 | 0.311 |
| rs76390238 | C | G | 0.6223 | 0.1352 | 4.141E-06 | 21.17391 | 0.1328 | 0.1599 |
| rs79454658 | T | C | 0.2784 | 0.0596 | 3.017E-06 | 21.8072442 | 0.0628 | 0.5045 |
| **HGF** | | | | | | | | |
| rs11060254 | A | G | -0.0765 | 0.0166 | 3.974E-06 | 21.2325661 | 0.0271 | 0.1812 |
| rs11129909 | T | C | -0.0738 | 0.0161 | 4.457E-06 | 21.0066482 | 0.0259 | 0.3389 |
| rs13412535 | A | G | -0.1043 | 0.0213 | 9.671E-07 | 23.9720514 | 0.0316 | 0.4581 |
| rs2003620 | T | C | 0.2277 | 0.0487 | 2.978E-06 | 21.8556602 | 0.075 | 0.745501 |
| rs362307 | T | C | 0.1511 | 0.0328 | 0.00000421 | 21.2166607 | 0.0499 | 0.0690494 |
| rs4245058 | T | C | -0.1552 | 0.0331 | 2.683E-06 | 21.9797747 | 0.0521 | 0.3658 |
| rs57146176 | A | G | -0.0987 | 0.0208 | 2.184E-06 | 22.5114477 | 0.0503 | 0.723601 |
| rs5745687 | T | C | -0.3008 | 0.0404 | 9.922E-14 | 55.4228333 | 0.0625 | 0.2633 |
| rs80051150 | T | C | 0.198 | 0.0413 | 1.684E-06 | 22.9787324 | 0.0681 | 0.1579 |
| **IFN-G** | | | | | | | | |
| rs113399544 | A | G | -0.0849 | 0.0183 | 3.323E-06 | 21.5179571 | 0.0278 | 0.1892 |
| rs113600793 | A | C | 0.1871 | 0.0371 | 4.426E-07 | 25.4265591 | 0.0541 | 0.7206 |
| rs115729819 | A | G | 0.2511 | 0.0514 | 1.045E-06 | 23.8591531 | 0.0781 | 0.1677 |
| rs117046255 | T | C | -0.0968 | 0.0207 | 2.787E-06 | 21.8624013 | 0.0325 | 0.3173 |
| rs11843756 | T | G | 0.1812 | 0.0391 | 3.622E-06 | 21.4709227 | 0.0616 | 0.4303 |
| rs12420286 | T | C | 0.2357 | 0.05 | 2.452E-06 | 22.2160576 | 0.0774 | 0.3293 |
| rs147378920 | A | G | -0.384 | 0.0751 | 3.195E-07 | 26.1378831 | 0.0894 | 0.971 |
| rs1867282 | T | C | 0.0781 | 0.0166 | 2.478E-06 | 22.1296084 | 0.0255 | 0.5299 |
| rs2073438 | A | G | 0.092 | 0.0188 | 9.551E-07 | 23.9413036 | 0.0287 | 0.475501 |
| rs7088799 | T | G | -0.0805 | 0.0166 | 1.274E-06 | 23.5105843 | 0.0258 | 0.903 |
| rs73479333 | C | G | -0.1123 | 0.024 | 2.816E-06 | 21.8889468 | 0.0373 | 0.0220501 |
| rs74148555 | T | C | -0.3771 | 0.077 | 9.858E-07 | 23.9783586 | 0.0889 | 0.4084 |
| **IL-1B** | | | | | | | | |
| rs143319329 | T | C | 0.4357 | 0.093 | 2.835E-06 | 21.9356097 | 0.156 | 0.1397 |
| rs4786740 | A | C | 0.1264 | 0.0265 | 0.00000182 | 22.7375092 | 0.0257 | 0.343 |
| rs61335305 | A | C | 0.4333 | 0.0928 | 3.015E-06 | 21.7882281 | 0.0936 | 0.742299 |
| **IL-1RA** | | | | | | | | |
| rs1054402 | T | C | 0.1325 | 0.0269 | 8.201E-07 | 24.2488368 | 0.029 | 0.00617405 |
| rs117181659 | A | G | -0.2204 | 0.0478 | 0.00000392 | 21.2486381 | 0.0511 | 0.8281 |
| rs11869294 | C | G | -0.2286 | 0.047 | 1.128E-06 | 23.6439727 | 0.0468 | 0.9922 |
| rs13343438 | A | G | 0.2771 | 0.0607 | 4.973E-06 | 20.8285666 | 0.066 | 0.4072 |
| rs35590641 | C | G | -0.1167 | 0.025 | 3.041E-06 | 21.7783718 | 0.0265 | 0.762801 |
| rs3876037 | A | G | 0.1234 | 0.027 | 4.733E-06 | 20.8769237 | 0.0261 | 0.6438 |
| rs56134659 | A | G | -0.1109 | 0.0236 | 2.564E-06 | 22.0700237 | 0.0251 | 0.770999 |
| rs61335305 | A | C | 0.4315 | 0.0904 | 1.812E-06 | 22.7713329 | 0.0936 | 0.742299 |
| rs6699436 | A | G | -0.1858 | 0.0404 | 4.365E-06 | 21.1393876 | 0.0405 | 0.7548 |
| rs9985296 | T | C | 0.1053 | 0.0231 | 4.952E-06 | 20.7680871 | 0.0252 | 0.3579 |
| **IL-2** | | | | | | | | |
| rs13412535 | A | G | 0.174 | 0.0331 | 1.447E-07 | 27.6181929 | 0.0316 | 0.4581 |
| rs16836080 | A | G | 0.1158 | 0.0253 | 4.841E-06 | 20.9376929 | 0.0269 | 0.0827694 |
| rs2690020 | A | G | 0.1158 | 0.0245 | 2.273E-06 | 22.3273767 | 0.0253 | 0.655 |
| rs4479767 | A | G | 0.1821 | 0.0392 | 3.444E-06 | 21.5675348 | 0.0434 | 0.46 |
| rs4634519 | A | G | -0.1249 | 0.0268 | 0.00000318 | 21.7074115 | 0.0282 | 0.8112 |
| rs61335305 | A | C | 0.4439 | 0.0913 | 1.157E-06 | 23.6255259 | 0.0936 | 0.742299 |
| rs62124990 | T | G | -0.7013 | 0.149 | 2.502E-06 | 22.1405238 | 0.0921 | 0.5658 |
| rs7615304 | A | G | -0.1139 | 0.024 | 2.161E-06 | 22.5101151 | 0.0252 | 0.5572 |
| **IL-2RA** | | | | | | | | |
| rs11241559 | T | G | -0.124 | 0.0264 | 0.00000275 | 22.0496505 | 0.0287 | 0.5593 |
| rs117244812 | A | G | -0.7187 | 0.1493 | 1.474E-06 | 23.1601767 | 0.1526 | 0.00496204 |
| rs12789243 | T | C | 0.1263 | 0.0276 | 4.608E-06 | 20.9293013 | 0.0291 | 0.8638 |
| rs17147986 | A | C | -0.2976 | 0.0337 | 1.069E-18 | 77.9421257 | 0.0357 | 0.0189998 |
| rs17624670 | A | G | -0.125 | 0.0273 | 4.639E-06 | 20.9537099 | 0.0291 | 0.3797 |
| rs34037190 | A | G | 0.4784 | 0.0935 | 3.134E-07 | 26.1652756 | 0.0767 | 0.4238 |
| rs56213152 | T | C | 0.1269 | 0.0271 | 2.937E-06 | 21.9154734 | 0.0293 | 0.6413 |
| rs759244 | A | T | -0.1094 | 0.0238 | 4.257E-06 | 21.117715 | 0.0257 | 0.3744 |
| rs79100208 | C | G | 0.8345 | 0.1758 | 2.052E-06 | 22.5206611 | 0.1565 | 0.8294 |
| **IL-4** | | | | | | | | |
| rs116705532 | T | G | -0.4675 | 0.0978 | 1.727E-06 | 22.844369 | 0.1548 | 0.741401 |
| rs117146485 | T | C | -0.2856 | 0.0625 | 4.945E-06 | 20.8761312 | 0.093 | 0.655399 |
| rs12238729 | T | C | 0.5271 | 0.1096 | 1.505E-06 | 23.116432 | 0.2044 | 0.4923 |
| rs12640583 | T | G | -0.1104 | 0.0214 | 2.451E-07 | 26.6075108 | 0.0336 | 0.9884 |
| rs17713451 | A | G | 0.1255 | 0.0252 | 6.41E-07 | 24.795908 | 0.0397 | 0.0696097 |
| rs1867282 | T | C | 0.0808 | 0.0162 | 5.822E-07 | 24.8706044 | 0.0255 | 0.5299 |
| rs2073438 | A | G | 0.0847 | 0.0183 | 3.725E-06 | 21.4169828 | 0.0287 | 0.475501 |
| rs2346020 | A | G | 0.079 | 0.0169 | 2.843E-06 | 21.8461253 | 0.0269 | 0.0978904 |
| rs2708586 | T | C | -0.0767 | 0.0166 | 3.588E-06 | 21.3436258 | 0.0266 | 0.6814 |
| rs56408830 | A | G | -0.1794 | 0.0365 | 9.075E-07 | 24.151983 | 0.0609 | 0.656299 |
| rs7613691 | A | G | 0.1787 | 0.0382 | 2.962E-06 | 21.8784235 | 0.0576 | 0.8215 |
| rs79597994 | T | C | -0.5855 | 0.1271 | 4.056E-06 | 21.2156501 | 0.15 | 0.1501 |
| rs9506111 | A | G | -0.1446 | 0.0314 | 4.081E-06 | 21.2017123 | 0.0503 | 0.1359 |
| rs9941733 | A | G | 0.1156 | 0.0229 | 4.331E-07 | 25.4764171 | 0.0333 | 0.6125 |
| **IL-5** | | | | | | | | |
| rs10178043 | T | G | 0.2579 | 0.0553 | 3.126E-06 | 21.7368748 | 0.0568 | 0.6469 |
| rs148634917 | A | G | -0.517 | 0.1087 | 1.974E-06 | 22.6082361 | 0.0939 | 0.633501 |
| rs28793375 | T | C | 0.1697 | 0.0362 | 2.746E-06 | 21.962978 | 0.0372 | 0.0476705 |
| rs72831687 | A | G | -0.5337 | 0.1104 | 1.324E-06 | 23.3561331 | 0.0982 | 0.4515 |
| rs73040118 | T | C | 0.2294 | 0.049 | 2.903E-06 | 21.9048029 | 0.0522 | 0.6062 |
| rs74811276 | A | G | 0.217 | 0.0471 | 4.082E-06 | 21.2139889 | 0.0455 | 0.1901 |
| rs7739450 | A | G | -0.1295 | 0.0256 | 4.05E-07 | 25.5743314 | 0.0254 | 0.7944 |
| rs9309063 | T | G | -0.1119 | 0.0245 | 4.866E-06 | 20.8483979 | 0.0252 | 0.4702 |
| **IL-6** | | | | | | | | |
| rs10910395 | A | T | -0.108 | 0.0235 | 4.348E-06 | 21.1157384 | 0.0367 | 0.9766 |
| rs10982193 | A | G | -0.0793 | 0.0174 | 4.816E-06 | 20.7654986 | 0.0281 | 0.1791 |
| rs113098456 | A | G | -0.1553 | 0.0339 | 4.641E-06 | 20.9815709 | 0.0482 | 0.3822 |
| rs113600793 | A | C | 0.1736 | 0.0359 | 1.291E-06 | 23.3778749 | 0.0541 | 0.7206 |
| rs114373846 | T | C | 0.4196 | 0.0905 | 3.568E-06 | 21.4915771 | 0.1433 | 0.8178 |
| rs11732981 | A | C | 0.0722 | 0.0156 | 3.793E-06 | 21.4150792 | 0.0252 | 0.584501 |
| rs1333040 | T | C | 0.0747 | 0.0157 | 1.993E-06 | 22.632701 | 0.0251 | 0.1229 |
| rs13412535 | A | G | -0.1186 | 0.0214 | 3.141E-08 | 30.7069242 | 0.0316 | 0.4581 |
| rs4684700 | T | C | -0.0747 | 0.0162 | 3.912E-06 | 21.2571805 | 0.0253 | 0.7627 |
| rs73273528 | T | C | 0.268 | 0.0553 | 0.00000125 | 23.4808499 | 0.0826 | 0.8922 |
| rs76856708 | T | C | 0.336 | 0.0697 | 1.427E-06 | 23.2331173 | 0.1089 | 0.8785 |
| **IL-7** | | | | | | | | |
| rs117509142 | T | C | -0.3213 | 0.0684 | 2.599E-06 | 22.0524712 | 0.0694 | 0.589599 |
| rs11757972 | T | C | 0.121 | 0.0257 | 2.529E-06 | 22.1540183 | 0.0254 | 0.3424 |
| rs1374279 | A | T | 0.1625 | 0.0347 | 2.792E-06 | 21.9177413 | 0.0358 | 0.83 |
| rs142397827 | A | C | 0.4592 | 0.0994 | 3.822E-06 | 21.329418 | 0.0907 | 0.6271 |
| rs17091524 | T | C | 0.5092 | 0.1015 | 5.244E-07 | 25.1531697 | 0.0964 | 0.6665 |
| rs2006957 | T | C | 0.2557 | 0.0262 | 1.434E-22 | 95.1934111 | 0.0267 | 0.7561 |
| rs218238 | A | T | 0.1319 | 0.0284 | 3.277E-06 | 21.5576257 | 0.0299 | 0.7637 |
| rs28793375 | T | C | 0.1644 | 0.036 | 4.866E-06 | 20.8423479 | 0.0372 | 0.0476705 |
| rs62006410 | T | C | -0.1492 | 0.0302 | 7.588E-07 | 24.3933685 | 0.0297 | 0.134 |
| rs7155170 | A | T | -0.1236 | 0.027 | 4.787E-06 | 20.9438939 | 0.0279 | 0.6271 |
| rs77318030 | T | C | -0.2966 | 0.0631 | 2.639E-06 | 22.0816535 | 0.0625 | 0.3814 |
| rs77981494 | T | C | -0.5201 | 0.1055 | 8.225E-07 | 24.289401 | 0.0904 | 0.5848 |
| **IL-8** | | | | | | | | |
| rs113487695 | A | C | -0.6129 | 0.1292 | 2.092E-06 | 22.49109 | 0.1364 | 0.2435 |
| rs116726256 | T | C | -0.2247 | 0.0489 | 4.261E-06 | 21.1030251 | 0.0533 | 0.116 |
| rs12075 | A | G | 0.1148 | 0.0235 | 9.969E-07 | 23.8508763 | 0.0252 | 0.4047 |
| rs12912642 | A | G | 0.1168 | 0.0251 | 3.212E-06 | 21.6418574 | 0.0264 | 0.1968 |
| rs183628733 | T | C | 0.6547 | 0.1417 | 3.821E-06 | 21.3354236 | 0.1225 | 0.3767 |
| rs2673604 | A | C | -0.118 | 0.0254 | 3.289E-06 | 21.5701353 | 0.0271 | 0.3721 |
| rs3786107 | A | G | 0.2463 | 0.0517 | 1.935E-06 | 22.6831845 | 0.0484 | 0.8422 |
| rs75840288 | A | C | 0.5125 | 0.1121 | 4.846E-06 | 20.8897288 | 0.1062 | 0.4118 |
| **IL-9** | | | | | | | | |
| rs117807175 | C | G | -0.5225 | 0.1106 | 2.327E-06 | 22.3062103 | 0.1124 | 0.8894 |
| rs1259728 | A | G | -0.2381 | 0.0507 | 2.599E-06 | 22.0427781 | 0.0541 | 0.3681 |
| rs3736858 | C | G | -0.1351 | 0.0291 | 3.373E-06 | 21.5421069 | 0.0312 | 0.365 |
| rs41294750 | T | C | 0.3442 | 0.0736 | 2.916E-06 | 21.8589632 | 0.0742 | 0.2949 |
| rs4880409 | T | C | -0.3552 | 0.0716 | 6.952E-07 | 24.5970671 | 0.1255 | 0.580301 |
| rs73443903 | A | C | 0.2162 | 0.046 | 2.569E-06 | 22.0779717 | 0.0501 | 0.2426 |
| **IL-10** | | | | | | | | |
| rs10457128 | A | G | -0.0854 | 0.0172 | 6.956E-07 | 24.6459963 | 0.0261 | 0.0662293 |
| rs10493718 | A | C | -0.1081 | 0.0222 | 1.068E-06 | 23.7046186 | 0.0346 | 0.8821 |
| rs13412535 | A | G | -0.1347 | 0.0224 | 1.798E-09 | 36.1515304 | 0.0316 | 0.4581 |
| rs1530455 | T | C | 0.082 | 0.0174 | 2.527E-06 | 22.2032598 | 0.0263 | 0.4435 |
| rs2086656 | T | C | -0.08 | 0.017 | 2.589E-06 | 22.1395946 | 0.0265 | 0.2087 |
| rs3002131 | C | G | 0.1191 | 0.026 | 4.592E-06 | 20.9780134 | 0.0368 | 0.7953 |
| rs3025021 | T | C | 0.0913 | 0.0194 | 2.609E-06 | 22.1424477 | 0.0267 | 0.645199 |
| rs383684 | A | G | 0.092 | 0.0197 | 3.168E-06 | 21.8037269 | 0.0442 | 0.701501 |
| rs4741748 | A | G | -0.0788 | 0.0169 | 3.202E-06 | 21.735346 | 0.0257 | 0.4486 |
| rs6054847 | T | C | 0.0971 | 0.0207 | 2.752E-06 | 21.9981065 | 0.0321 | 0.4368 |
| rs6680918 | T | C | -0.1202 | 0.025 | 1.591E-06 | 23.1108783 | 0.0384 | 0.3822 |
| rs7088799 | T | G | -0.0815 | 0.0166 | 9.352E-07 | 24.0983093 | 0.0258 | 0.903 |
| rs73192842 | A | G | 0.0949 | 0.0206 | 4.031E-06 | 21.2170752 | 0.0322 | 0.3705 |
| rs7747448 | A | G | -0.1061 | 0.0189 | 1.998E-08 | 31.5061032 | 0.0287 | 0.9322 |
| rs9472173 | T | C | -0.2004 | 0.0174 | 1.256E-30 | 132.612502 | 0.0258 | 0.2465 |
| **IL-12p70** |  |  |  |  |  |  |  |  |
| rs113600793 | A | C | 0.1832 | 0.0359 | 3.351E-07 | 26.0349984 | 0.0541 | 0.7206 |
| rs12969892 | T | C | 0.1227 | 0.0267 | 4.194E-06 | 21.1135916 | 0.0418 | 0.9596 |
| rs2123852 | T | C | 0.0942 | 0.0204 | 0.00000373 | 21.317535 | 0.0316 | 0.8701 |
| rs273702 | A | G | -0.127 | 0.027 | 2.522E-06 | 22.1195062 | 0.0423 | 0.9345 |
| rs282258 | T | C | 0.0726 | 0.0156 | 3.282E-06 | 21.6530739 | 0.0254 | 0.5467 |
| rs34322762 | T | C | 0.0953 | 0.0199 | 1.708E-06 | 22.9267052 | 0.0264 | 0.3609 |
| rs34826779 | T | G | -0.0884 | 0.019 | 3.327E-06 | 21.6417733 | 0.0305 | 0.9786 |
| rs41282644 | A | G | 0.1401 | 0.0303 | 3.737E-06 | 21.3740356 | 0.0467 | 0.2797 |
| rs4530855 | T | G | 0.0861 | 0.0184 | 2.872E-06 | 21.8910287 | 0.0278 | 0.2504 |
| rs4741748 | A | G | -0.0799 | 0.0163 | 9.162E-07 | 24.0222601 | 0.0257 | 0.4486 |
| rs6532374 | T | C | -0.1033 | 0.0226 | 4.613E-06 | 20.8871545 | 0.0358 | 0.8753 |
| rs7754905 | A | G | -0.1005 | 0.019 | 1.14E-07 | 27.9718014 | 0.0299 | 0.4873 |
| rs782111 | A | C | -0.0765 | 0.0156 | 9.247E-07 | 24.0419222 | 0.0251 | 0.2621 |
| rs865585 | A | C | -0.1654 | 0.0237 | 2.732E-12 | 48.69337 | 0.0356 | 0.3906 |
| rs9381249 | T | C | -0.1788 | 0.0367 | 1.125E-06 | 23.730037 | 0.0621 | 0.1267 |
| **IL-13** | | | | | | | | |
| rs10995604 | A | G | -0.1571 | 0.0343 | 4.482E-06 | 20.966335 | 0.0355 | 0.5031 |
| rs117795020 | A | G | -0.3584 | 0.0716 | 5.479E-07 | 25.0419617 | 0.0746 | 0.3778 |
| rs12623722 | A | G | -0.1189 | 0.0257 | 3.614E-06 | 21.3922198 | 0.0273 | 0.219 |
| rs138854806 | A | G | -0.4204 | 0.0839 | 5.449E-07 | 25.0934216 | 0.076 | 0.5434 |
| rs139083458 | T | C | 0.9995 | 0.211 | 2.165E-06 | 22.4263748 | 0.2091 | 0.3269 |
| rs147747784 | C | G | 0.369 | 0.0765 | 1.435E-06 | 23.2534958 | 0.0595 | 0.6254 |
| rs150836197 | T | C | 0.3283 | 0.0713 | 4.136E-06 | 21.1895189 | 0.0641 | 0.2067 |
| rs27949 | T | C | -0.1144 | 0.025 | 4.827E-06 | 20.9281299 | 0.0269 | 0.1885 |
| rs28442067 | A | G | -0.1379 | 0.0286 | 1.413E-06 | 23.2356516 | 0.0306 | 0.2615 |
| rs7073807 | T | C | 0.1618 | 0.0354 | 0.00000477 | 20.8789618 | 0.037 | 0.6036 |
| rs75383097 | C | G | -0.5369 | 0.116 | 3.702E-06 | 21.4106188 | 0.1028 | 0.1379 |
| rs76339001 | A | T | -0.4375 | 0.0886 | 7.915E-07 | 24.3695248 | 0.0757 | 0.7866 |
| rs76975337 | T | C | -0.1211 | 0.0265 | 4.921E-06 | 20.8715608 | 0.0285 | 0.510199 |
| rs77955971 | A | C | 0.4408 | 0.0868 | 3.756E-07 | 25.775204 | 0.0701 | 0.9719 |
| **IL-16** | | | | | | | | |
| rs117217798 | T | C | -0.2064 | 0.044 | 2.772E-06 | 21.992129 | 0.0451 | 0.1319 |
| rs12577604 | T | C | 0.4335 | 0.0941 | 4.083E-06 | 21.2105631 | 0.0978 | 0.4959 |
| rs142034902 | A | G | -0.4367 | 0.0925 | 2.327E-06 | 22.2759462 | 0.0993 | 0.7975 |
| rs142332135 | A | G | -0.7646 | 0.1082 | 1.581E-12 | 49.9076715 | 0.0976 | 0.9202 |
| rs144691581 | A | G | 0.4929 | 0.0958 | 2.668E-07 | 26.4569551 | 0.0864 | 0.3976 |
| rs35834666 | T | C | -0.1729 | 0.0348 | 6.57E-07 | 24.6708857 | 0.0358 | 0.665001 |
| rs4778640 | A | G | 0.7189 | 0.0983 | 2.552E-13 | 53.4543648 | 0.0968 | 0.2339 |
| rs4976691 | C | G | 0.1254 | 0.026 | 1.473E-06 | 23.2488577 | 0.0269 | 0.0328299 |
| rs7097884 | T | C | -0.1193 | 0.0243 | 8.809E-07 | 24.0891559 | 0.0254 | 0.309 |
| rs78042619 | A | G | 0.55 | 0.1158 | 2.023E-06 | 22.5455847 | 0.1112 | 0.565599 |
| **IL-17** | | | | | | | | |
| rs11985957 | A | G | 0.1511 | 0.0329 | 4.363E-06 | 21.0875351 | 0.054 | 0.1094 |
| rs12735700 | T | G | -0.0943 | 0.0206 | 0.0000045 | 20.9496914 | 0.0308 | 0.4708 |
| rs145006174 | C | G | -0.2266 | 0.0473 | 1.645E-06 | 22.9449024 | 0.072 | 0.2834 |
| rs17282552 | T | C | -0.2026 | 0.0403 | 4.876E-07 | 25.2672208 | 0.0584 | 0.0145001 |
| rs3792369 | A | G | 0.0941 | 0.0166 | 1.46E-08 | 32.1256376 | 0.0258 | 0.6859 |
| rs61990749 | C | G | 0.1124 | 0.0226 | 6.569E-07 | 24.728879 | 0.035 | 0.0844306 |
| rs78296352 | T | G | 0.2949 | 0.0645 | 4.809E-06 | 20.8986774 | 0.1091 | 0.4063 |
| rs9519328 | A | G | 0.5256 | 0.1101 | 0.00000179 | 22.7767495 | 0.0821 | 0.4461 |
| **IL-18** | | | | | | | | |
| rs10409850 | A | G | 0.1791 | 0.0347 | 2.44E-07 | 26.6253795 | 0.037 | 0.693999 |
| rs11214093 | T | C | 0.1143 | 0.0238 | 1.544E-06 | 23.0516561 | 0.0253 | 0.508 |
| rs117266781 | T | C | 0.7051 | 0.1436 | 9.176E-07 | 24.0966203 | 0.1366 | 0.0232199 |
| rs117371668 | T | G | 0.3712 | 0.0799 | 3.357E-06 | 21.571779 | 0.0859 | 0.0294503 |
| rs139468359 | T | C | 0.5101 | 0.1088 | 2.737E-06 | 21.9693112 | 0.1132 | 0.1574 |
| rs1979967 | T | C | 0.14 | 0.0285 | 8.719E-07 | 24.1173694 | 0.0307 | 0.4092 |
| rs4952239 | A | T | -0.1156 | 0.0242 | 1.809E-06 | 22.8059686 | 0.0261 | 0.3792 |
| rs58701153 | A | T | -0.1265 | 0.0242 | 1.807E-07 | 27.3095098 | 0.0263 | 0.1263 |
| rs62312914 | T | C | -0.1265 | 0.025 | 4.215E-07 | 25.5896661 | 0.026 | 0.8687 |
| rs764078 | A | T | 0.1283 | 0.0278 | 4.075E-06 | 21.2876296 | 0.0298 | 0.214 |
| rs77187209 | T | C | -0.4859 | 0.1041 | 3.082E-06 | 21.7748877 | 0.109 | 0.2879 |
| rs78623212 | T | C | 0.8322 | 0.1676 | 6.82E-07 | 24.6417178 | 0.1447 | 0.2017 |
| rs78716465 | A | G | 0.3173 | 0.0679 | 2.981E-06 | 21.8254989 | 0.0668 | 0.8231 |
| **IP-10** | | | | | | | | |
| rs113183470 | A | T | -0.2414 | 0.0524 | 4.147E-06 | 21.2118552 | 0.0535 | 0.793299 |
| rs12714300 | A | T | -0.1573 | 0.0338 | 3.297E-06 | 21.6466523 | 0.0361 | 0.2826 |
| rs143799975 | A | G | -0.7551 | 0.1638 | 4.012E-06 | 21.2396738 | 0.1535 | 0.6866 |
| rs34383175 | T | C | -0.3196 | 0.0653 | 9.904E-07 | 23.9416388 | 0.0696 | 0.2883 |
| rs397816 | T | C | 0.1211 | 0.0248 | 1.026E-06 | 23.8315133 | 0.026 | 0.0717299 |
| rs4859940 | C | G | -0.1204 | 0.0258 | 3.228E-06 | 21.7660819 | 0.0275 | 0.763001 |
| rs4862110 | T | C | -0.1453 | 0.0318 | 4.909E-06 | 20.8662153 | 0.029 | 0.9169 |
| rs75970138 | A | G | -0.4845 | 0.1037 | 2.994E-06 | 21.8170861 | 0.1162 | 0.4686 |
| rs7645625 | T | G | -0.1116 | 0.0236 | 2.192E-06 | 22.3496682 | 0.0256 | 0.8203 |
| rs78077394 | T | C | -0.3486 | 0.0701 | 6.474E-07 | 24.716412 | 0.0686 | 0.859 |
| rs79848609 | A | C | 0.2514 | 0.0535 | 2.637E-06 | 22.069357 | 0.058 | 0.9263 |
| rs8112618 | A | G | 0.1388 | 0.0297 | 3.047E-06 | 21.8289442 | 0.0317 | 0.4343 |
| **M-CSF** | | | | | | | | |
| rs116274860 | T | G | 0.8262 | 0.1739 | 2.029E-06 | 22.553989 | 0.1113 | 0.868 |
| rs116887628 | A | G | -0.2741 | 0.0598 | 4.626E-06 | 20.9926783 | 0.049 | 0.6688 |
| rs117867915 | T | C | 0.5224 | 0.1096 | 1.874E-06 | 22.700602 | 0.0916 | 0.3782 |
| rs11963606 | C | G | -0.5353 | 0.117 | 4.731E-06 | 20.9158207 | 0.1 | 0.4655 |
| rs12962919 | T | C | 0.3025 | 0.0659 | 4.394E-06 | 21.0538767 | 0.0495 | 0.4183 |
| rs139457375 | A | C | -0.4047 | 0.0854 | 2.142E-06 | 22.4389646 | 0.0737 | 0.1203 |
| rs147378920 | A | G | -0.6064 | 0.1318 | 4.177E-06 | 21.1514289 | 0.0894 | 0.971 |
| rs34089869 | T | C | 0.2194 | 0.0462 | 2.078E-06 | 22.5341636 | 0.0405 | 0.3548 |
| rs62294910 | A | G | 0.3472 | 0.0687 | 4.378E-07 | 25.5210246 | 0.0519 | 0.1327 |
| rs72723242 | T | G | -0.4969 | 0.1083 | 4.434E-06 | 21.0345501 | 0.0991 | 0.171 |
| rs9387100 | T | C | -0.135 | 0.029 | 3.341E-06 | 21.6532798 | 0.0254 | 0.634 |
| rs9626985 | T | C | 0.2277 | 0.0496 | 4.482E-06 | 21.0578901 | 0.0424 | 0.5319 |
| **MCP-1** | | | | | | | | |
| rs111995966 | T | G | 0.1428 | 0.0309 | 3.788E-06 | 21.3518377 | 0.0474 | 0.6643 |
| rs11920996 | T | C | 0.1805 | 0.0376 | 1.604E-06 | 23.0395783 | 0.0595 | 0.8453 |
| rs12062235 | T | G | 0.1477 | 0.032 | 3.836E-06 | 21.2988834 | 0.0516 | 0.8115 |
| rs143815843 | A | G | -0.2049 | 0.0447 | 4.609E-06 | 21.0070308 | 0.0735 | 0.0327703 |
| rs16837903 | A | G | -0.1104 | 0.0238 | 3.352E-06 | 21.5119627 | 0.0372 | 0.555199 |
| rs2201150 | T | C | 0.0916 | 0.016 | 1.044E-08 | 32.7677623 | 0.0259 | 0.7307 |
| rs2229593 | T | C | 0.2624 | 0.0405 | 9.246E-11 | 41.9675308 | 0.0645 | 0.8151 |
| rs56212190 | T | C | 0.1799 | 0.0372 | 1.318E-06 | 23.3814936 | 0.0585 | 0.9601 |
| rs62245103 | T | G | 0.2433 | 0.0416 | 4.989E-09 | 34.1974213 | 0.0618 | 0.9954 |
| rs7197349 | A | G | 0.0971 | 0.0206 | 2.399E-06 | 22.2126218 | 0.0316 | 0.4721 |
| rs72705803 | A | G | -0.2188 | 0.047 | 3.222E-06 | 21.6667974 | 0.0806 | 0.7092 |
| rs7978037 | A | T | 0.0746 | 0.016 | 3.043E-06 | 21.7336912 | 0.0259 | 0.8194 |
| rs79939301 | A | G | 0.1449 | 0.0255 | 1.356E-08 | 32.281389 | 0.0398 | 0.8614 |
| rs856100 | A | G | 0.0899 | 0.0191 | 2.545E-06 | 22.1487108 | 0.0307 | 0.2179 |
| rs862990 | T | C | -0.089 | 0.0183 | 1.153E-06 | 23.6468685 | 0.0289 | 0.2094 |
| rs9317045 | A | C | 0.1157 | 0.0235 | 8.425E-07 | 24.2340944 | 0.0355 | 0.3707 |
| **MCP-3** | | | | | | | | |
| rs10892381 | T | C | 0.2432 | 0.0473 | 2.693E-07 | 26.3884447 | 0.0267 | 0.5109 |
| rs117286643 | A | G | 0.6934 | 0.1474 | 2.542E-06 | 22.0892842 | 0.0858 | 0.5261 |
| rs28394764 | A | T | 0.597 | 0.1282 | 3.194E-06 | 21.646194 | 0.0717 | 0.4953 |
| rs3129806 | T | C | -0.1975 | 0.0433 | 4.978E-06 | 20.766693 | 0.0254 | 0.0697606 |
| rs6993671 | T | C | 0.2041 | 0.0443 | 4.061E-06 | 21.1878794 | 0.0257 | 0.5104 |
| rs7275485 | T | C | -0.2218 | 0.0481 | 3.986E-06 | 21.2247149 | 0.028 | 0.8944 |
| **MIF** | | | | | | | | |
| rs1007888 | T | C | -0.1275 | 0.0245 | 1.915E-07 | 27.0671345 | 0.0258 | 0.5925 |
| rs113218956 | A | G | -0.8789 | 0.1876 | 2.815E-06 | 21.9365002 | 0.1986 | 0.842 |
| rs11551183 | C | G | 0.3666 | 0.0795 | 3.999E-06 | 21.2522416 | 0.0856 | 0.5517 |
| rs12594190 | A | G | 0.1321 | 0.0266 | 6.85E-07 | 24.6488375 | 0.0275 | 0.750699 |
| rs141009259 | T | C | -0.6194 | 0.1285 | 1.444E-06 | 23.2214977 | 0.1207 | 0.2243 |
| rs2294689 | C | G | -0.1338 | 0.0287 | 3.043E-06 | 21.7221353 | 0.1016 | 0.1627 |
| rs35792361 | A | G | -0.2586 | 0.0527 | 9.003E-07 | 24.065223 | 0.0542 | 0.4814 |
| rs35890933 | T | G | 0.1676 | 0.0365 | 4.458E-06 | 21.0725154 | 0.0358 | 0.01033 |
| rs3814097 | A | G | -0.1163 | 0.0251 | 3.548E-06 | 21.456855 | 0.0256 | 0.640801 |
| rs78098071 | T | C | -0.4583 | 0.0915 | 5.509E-07 | 25.0733063 | 0.0991 | 0.6321 |
| **MIG** |  |  |  |  |  |  |  |  |
| rs10266753 | T | C | -0.2016 | 0.0397 | 3.769E-07 | 25.7731047 | 0.0448 | 0.751299 |
| rs111607343 | A | G | -0.5235 | 0.1119 | 2.928E-06 | 21.8745887 | 0.11 | 0.2064 |
| rs11177248 | A | G | 0.3157 | 0.0667 | 2.222E-06 | 22.3905207 | 0.0647 | 0.7229 |
| rs113302091 | T | C | 0.2537 | 0.0553 | 4.402E-06 | 21.0357194 | 0.0603 | 0.7305 |
| rs13143163 | C | G | 0.2735 | 0.0582 | 2.622E-06 | 22.0716799 | 0.0542 | 0.6304 |
| rs139010077 | T | C | 0.4337 | 0.0943 | 4.193E-06 | 21.1408359 | 0.0987 | 0.9604 |
| rs191555775 | A | T | 0.2279 | 0.0412 | 3.278E-08 | 30.5816501 | 0.0417 | 0.4856 |
| rs192433162 | A | G | -0.8045 | 0.1676 | 1.594E-06 | 23.0287712 | 0.1762 | 0.0650205 |
| rs3733233 | T | C | 0.1223 | 0.025 | 1.049E-06 | 23.9188113 | 0.0264 | 0.331 |
| rs62562991 | A | G | 0.6239 | 0.1259 | 7.237E-07 | 24.5439924 | 0.1041 | 0.3103 |
| rs6679677 | A | C | 0.1628 | 0.0327 | 6.514E-07 | 24.7730793 | 0.0357 | 6.19156E-22 |
| rs8127917 | T | G | 0.2382 | 0.0492 | 1.278E-06 | 23.4271795 | 0.0524 | 0.6718 |
| rs816960 | T | C | -0.1179 | 0.0242 | 0.00000115 | 23.7226704 | 0.0262 | 0.7552 |
| **MIP-1A** | | | | | | | | |
| rs117506943 | T | C | 0.0108 | 0.0682 | 4.484E-06 | 21.0242617 | 0.0641 | 0.9058 |
| rs12159394 | A | G | 0.0008 | 0.0366 | 3.112E-06 | 21.7655465 | 0.0391 | 0.356 |
| rs57786342 | A | G | 0.0149 | 0.0283 | 8.909E-07 | 24.1108624 | 0.0333 | 0.2248 |
| rs6956239 | T | C | -0.0088 | 0.026 | 4.583E-06 | 20.9364595 | 0.0277 | 0.0164199 |
| **MIP-1B** | | | | | | | | |
| rs111721971 | T | G | -0.227 | 0.047 | 1.387E-06 | 23.321215 | 0.0694 | 0.774801 |
| rs116237296 | A | G | 0.5284 | 0.1115 | 2.153E-06 | 22.4528284 | 0.181 | 0.1838 |
| rs11651720 | T | C | -0.1189 | 0.0219 | 5.688E-08 | 29.4693566 | 0.0352 | 0.5597 |
| rs11716293 | C | G | 0.0986 | 0.0189 | 1.689E-07 | 27.2098029 | 0.0294 | 0.4263 |
| rs117657747 | A | G | 0.2089 | 0.0453 | 4.013E-06 | 21.2589533 | 0.0549 | 0.646501 |
| rs145526037 | T | G | -0.1863 | 0.0406 | 4.473E-06 | 21.0508102 | 0.0648 | 0.3363 |
| rs17138331 | A | G | -0.1434 | 0.0295 | 1.125E-06 | 23.623783 | 0.0455 | 0.6264 |
| rs17661219 | C | G | 0.0872 | 0.0172 | 3.888E-07 | 25.6963388 | 0.0267 | 0.6157 |
| rs2314809 | T | C | -0.0735 | 0.0157 | 2.904E-06 | 21.9114212 | 0.0252 | 0.8503 |
| rs2742396 | T | C | 0.1071 | 0.0168 | 1.832E-10 | 40.6308167 | 0.0257 | 0.8527 |
| rs281728 | A | C | -0.079 | 0.0171 | 3.891E-06 | 21.3381683 | 0.0273 | 0.5693 |
| rs28393318 | A | G | -0.1076 | 0.0235 | 4.616E-06 | 20.9596484 | 0.0377 | 0.307 |
| rs57893487 | C | G | -0.1075 | 0.0214 | 4.916E-07 | 25.2281007 | 0.0307 | 0.0274701 |
| rs6802288 | A | G | -0.1623 | 0.0173 | 6.574E-21 | 87.9913554 | 0.0283 | 0.3521 |
| rs6806860 | A | C | -0.1008 | 0.0188 | 8.575E-08 | 28.7409116 | 0.0301 | 0.6479 |
| rs6908843 | A | G | 0.0997 | 0.0209 | 1.779E-06 | 22.7506033 | 0.0335 | 0.634501 |
| rs72791296 | T | C | 0.2364 | 0.0466 | 3.968E-07 | 25.7287262 | 0.0699 | 0.2572 |
| rs72799710 | T | C | -0.1037 | 0.0217 | 1.792E-06 | 22.8314355 | 0.035 | 0.3026 |
| rs74979864 | A | T | -0.3184 | 0.0613 | 2.025E-07 | 26.9724467 | 0.0965 | 0.1124 |
| rs76582507 | A | G | 0.3259 | 0.0676 | 1.421E-06 | 23.2364888 | 0.1419 | 0.9496 |
| rs772112 | A | T | -0.142 | 0.0216 | 5.007E-11 | 43.2080195 | 0.0322 | 0.7251 |
| rs9916627 | T | C | -0.096 | 0.0198 | 1.244E-06 | 23.5021319 | 0.032 | 0.1014 |
| **PDGF-BB** | | | | | | | | |
| rs10512952 | T | C | -0.2816 | 0.0587 | 1.636E-06 | 23.0083196 | 0.0927 | 0.692299 |
| rs116154010 | T | C | 0.3225 | 0.0662 | 1.113E-06 | 23.726805 | 0.1097 | 0.6384 |
| rs11766649 | A | G | 0.0902 | 0.0196 | 3.964E-06 | 21.173699 | 0.031 | 0.3704 |
| rs12289510 | A | G | -0.0772 | 0.0158 | 1.001E-06 | 23.868011 | 0.0252 | 0.8261 |
| rs12615784 | T | C | -0.1003 | 0.0193 | 1.986E-07 | 27.0011991 | 0.0307 | 0.0971203 |
| rs147862316 | T | C | 0.2279 | 0.0411 | 2.987E-08 | 30.7397838 | 0.0697 | 0.0141501 |
| rs2643354 | A | G | 0.1251 | 0.0261 | 1.633E-06 | 22.9683294 | 0.0426 | 0.8165 |
| rs35859699 | A | G | -0.3854 | 0.0838 | 4.223E-06 | 21.146144 | 0.119 | 0.0362502 |
| rs62191444 | T | G | -0.112 | 0.0239 | 2.678E-06 | 21.9551317 | 0.0353 | 0.8824 |
| rs6756793 | T | C | 0.0876 | 0.0157 | 2.684E-08 | 31.1246668 | 0.0256 | 0.3795 |
| rs6910518 | T | G | 0.0806 | 0.0162 | 6.005E-07 | 24.7477578 | 0.0258 | 0.3596 |
| rs72972467 | C | G | -0.1616 | 0.0328 | 8.286E-07 | 24.2678235 | 0.0499 | 0.1033 |
| rs73162807 | A | C | -0.2313 | 0.0499 | 3.548E-06 | 21.4805787 | 0.0818 | 0.965 |
| rs9924851 | C | G | 0.0767 | 0.0163 | 2.702E-06 | 22.1366206 | 0.0263 | 0.3579 |
| **RANTES** | | | | | | | | |
| rs10505135 | T | C | 0.035 | 0.0252 | 1.899E-07 | 27.2144071 | 0.0263 | 0.8167 |
| rs118096511 | T | C | -0.0676 | 0.0709 | 1.965E-06 | 22.6332093 | 0.074 | 0.2784 |
| rs11873385 | A | G | 0.0096 | 0.0552 | 2.927E-06 | 21.8666762 | 0.055 | 0.9227 |
| rs148526102 | T | C | -0.002 | 0.083 | 4.793E-06 | 20.9267695 | 0.0862 | 0.2606 |
| rs2731672 | T | C | -0.0476 | 0.0272 | 4.827E-06 | 20.8378946 | 0.0289 | 0.00795591 |
| rs4795087 | C | G | -0.0065 | 0.0312 | 1.629E-06 | 22.9160869 | 0.0324 | 0.5318 |
| rs62438851 | A | G | -0.024 | 0.0413 | 4.009E-06 | 21.241356 | 0.0413 | 0.9639 |
| rs7170339 | C | G | 0.0645 | 0.0904 | 2.187E-06 | 22.434053 | 0.0892 | 0.178 |
| rs72793342 | A | G | -0.0251 | 0.0307 | 9.08E-07 | 24.0184348 | 0.0308 | 0.01366 |
| rs78050316 | A | C | -0.0279 | 0.0859 | 9.946E-07 | 23.9152113 | 0.0946 | 0.7788 |
| **SCF** | | | | | | | | |
| rs10800449 | A | C | 0.0851 | 0.0179 | 1.962E-06 | 22.5968979 | 0.0279 | 0.0404399 |
| rs11244035 | T | C | -0.1296 | 0.0279 | 3.501E-06 | 21.5723452 | 0.0451 | 0.2179 |
| rs113127926 | A | C | 0.1974 | 0.0418 | 2.337E-06 | 22.2965412 | 0.0645 | 0.9135 |
| rs117721699 | C | G | -0.2392 | 0.0484 | 7.514E-07 | 24.4189729 | 0.0778 | 0.6002 |
| rs12345108 | T | C | -0.0772 | 0.0167 | 3.731E-06 | 21.3647307 | 0.0264 | 0.091939 |
| rs13412535 | A | G | -0.1065 | 0.0213 | 5.586E-07 | 24.9940005 | 0.0316 | 0.4581 |
| rs138538809 | T | C | -0.5788 | 0.1139 | 3.757E-07 | 25.8169755 | 0.1672 | 0.9384 |
| rs72678285 | A | T | 0.1062 | 0.0231 | 0.00000443 | 21.1310384 | 0.0342 | 0.5776 |
| rs78666213 | T | G | -0.2845 | 0.0574 | 7.152E-07 | 24.5604614 | 0.0867 | 0.567 |
| rs8045376 | A | G | -0.3126 | 0.068 | 4.268E-06 | 21.1278783 | 0.1099 | 0.1354 |
| **SCGF-B** | | | | | | | | |
| rs11111869 | A | G | 0.1621 | 0.0311 | 1.861E-07 | 27.1526211 | 0.0338 | 0.8715 |
| rs112346514 | T | C | -0.3261 | 0.0703 | 3.543E-06 | 21.5058918 | 0.0659 | 0.3007 |
| rs1149926 | T | C | -0.3458 | 0.0749 | 3.917E-06 | 21.3035927 | 0.0818 | 0.736901 |
| rs118003677 | T | C | -0.3654 | 0.0786 | 3.348E-06 | 21.6002363 | 0.0844 | 0.1341 |
| rs12118918 | A | G | -0.1631 | 0.035 | 3.208E-06 | 21.7039281 | 0.0359 | 0.3702 |
| rs12480722 | T | C | 0.1654 | 0.0353 | 2.812E-06 | 21.9426011 | 0.0376 | 0.1531 |
| rs13287050 | A | T | -0.121 | 0.0263 | 4.118E-06 | 21.1556197 | 0.0281 | 0.8105 |
| rs13866 | T | C | -0.1647 | 0.028 | 3.773E-09 | 34.5810077 | 0.0286 | 0.0315602 |
| rs139413256 | A | G | -0.5174 | 0.1076 | 1.532E-06 | 23.109727 | 0.098 | 0.6193 |
| rs143829871 | T | C | -0.1866 | 0.0399 | 2.852E-06 | 21.8596898 | 0.0432 | 0.19 |
| rs144724875 | T | C | 0.5381 | 0.0829 | 8.645E-11 | 42.1098245 | 0.0761 | 0.5404 |
| rs149009264 | A | G | 0.4551 | 0.0985 | 3.793E-06 | 21.3357404 | 0.1046 | 0.0613705 |
| rs150733161 | T | C | -0.5255 | 0.112 | 2.687E-06 | 22.0026963 | 0.1088 | 0.807 |
| rs151194174 | A | G | 0.4536 | 0.0941 | 1.454E-06 | 23.2238038 | 0.0659 | 0.4201 |
| rs264157 | A | G | 0.1079 | 0.0233 | 3.685E-06 | 21.4337201 | 0.0251 | 0.7471 |
| rs34911860 | A | G | -0.3674 | 0.0787 | 3.002E-06 | 21.7818793 | 0.0998 | 0.9779 |
| rs3817303 | T | G | 0.1362 | 0.0294 | 3.602E-06 | 21.4499391 | 0.0318 | 0.0681899 |
| rs4737731 | T | C | 0.1146 | 0.0251 | 4.871E-06 | 20.8347503 | 0.0276 | 0.593401 |
| rs4976691 | C | G | -0.1484 | 0.0253 | 4.438E-09 | 34.3869192 | 0.0269 | 0.0328299 |
| rs77954165 | T | C | 0.2631 | 0.0562 | 2.867E-06 | 21.9046124 | 0.0574 | 0.9205 |
| rs78217154 | T | C | 0.3942 | 0.0861 | 4.722E-06 | 20.9504543 | 0.0907 | 0.298 |
| **SDF-1A** | | | | | | | | |
| rs10474392 | A | G | 0.0934 | 0.0177 | 1.376E-07 | 27.8378865 | 0.0289 | 0.1108 |
| rs10516368 | A | C | -0.4268 | 0.0883 | 1.356E-06 | 23.3569674 | 0.1361 | 0.3056 |
| rs12141941 | T | C | -0.0881 | 0.0186 | 2.263E-06 | 22.4292612 | 0.028 | 0.0503501 |
| rs149893336 | A | G | -0.494 | 0.1082 | 0.00000493 | 20.8395539 | 0.1157 | 0.2383 |
| rs1600396 | A | G | -0.0933 | 0.0204 | 4.939E-06 | 20.9118278 | 0.0317 | 0.02943 |
| rs62194946 | T | G | -0.0849 | 0.0185 | 4.552E-06 | 21.0552772 | 0.0287 | 0.4888 |
| rs6586903 | T | C | -0.1264 | 0.0268 | 2.421E-06 | 22.2389153 | 0.0401 | 0.2084 |
| rs76766406 | A | G | 0.4642 | 0.1012 | 4.489E-06 | 21.0347952 | 0.1463 | 0.7038 |
| rs78883416 | C | G | -0.0871 | 0.0182 | 1.755E-06 | 22.8972104 | 0.027 | 0.3342 |
| **TNF-A** | | | | | | | | |
| rs10767536 | A | G | 0.118 | 0.0253 | 3.146E-06 | 21.7407357 | 0.0267 | 0.635 |
| rs115018697 | C | G | -0.9542 | 0.197 | 1.273E-06 | 23.4475589 | 0.1963 | 0.3807 |
| rs116736594 | T | C | 0.3407 | 0.0702 | 1.223E-06 | 23.5408048 | 0.0678 | 0.760801 |
| rs79105320 | A | G | 0.5573 | 0.1177 | 2.207E-06 | 22.4066239 | 0.1194 | 0.8882 |
| **TNF-B** | | | | | | | | |
| rs10925040 | T | C | 0.1738 | 0.0372 | 2.929E-06 | 21.8001085 | 0.0262 | 0.2918 |
| rs2420873 | T | G | 0.1673 | 0.0365 | 4.513E-06 | 20.9822059 | 0.0259 | 0.9727 |
| rs62284710 | A | G | 0.3702 | 0.0782 | 2.183E-06 | 22.3822794 | 0.0531 | 0.6011 |
| rs75240021 | C | G | 0.3713 | 0.0772 | 1.489E-06 | 23.1025697 | 0.0484 | 0.2045 |
| rs76225863 | A | G | 0.7534 | 0.1217 | 5.982E-10 | 38.2749883 | 0.0808 | 0.8268 |
| **TRAIL** | | | | | | | | |
| rs113057689 | A | G | -0.2625 | 0.0489 | 7.972E-08 | 28.8094751 | 0.0609 | 0.3844 |
| rs11875481 | T | C | -0.0969 | 0.0211 | 4.601E-06 | 21.0851692 | 0.0337 | 0.0893902 |
| rs12458564 | A | T | -0.1002 | 0.0175 | 1.1E-08 | 32.7758372 | 0.0278 | 0.0157699 |
| rs13115587 | A | C | 0.101 | 0.0217 | 3.244E-06 | 21.657969 | 0.036 | 0.1812 |
| rs13278062 | T | G | 0.08 | 0.0157 | 3.326E-07 | 25.9582324 | 0.0253 | 0.1558 |
| rs139958028 | A | G | 0.1803 | 0.0395 | 4.992E-06 | 20.830117 | 0.0602 | 0.6673 |
| rs183815186 | A | T | -0.3499 | 0.0602 | 6.341E-09 | 33.7745328 | 0.0938 | 0.743901 |
| rs550057 | T | C | -0.0783 | 0.0169 | 3.707E-06 | 21.4607336 | 0.0272 | 0.5146 |
| rs558572 | T | C | 0.1351 | 0.0265 | 3.419E-07 | 25.9844422 | 0.0425 | 0.9605 |
| rs57396456 | T | C | -0.5641 | 0.0516 | 7.71E-28 | 119.48331 | 0.0844 | 0.6541 |
| rs616114 | T | C | -0.1033 | 0.0162 | 1.715E-10 | 40.6504223 | 0.0259 | 0.4641 |
| rs62093482 | T | C | 0.9827 | 0.0529 | 6.123E-77 | 345.004703 | 0.0879 | 0.2746 |
| rs747324 | T | C | -0.0826 | 0.0178 | 3.338E-06 | 21.528538 | 0.0279 | 0.0119201 |
| rs75928541 | A | G | 0.2784 | 0.0591 | 2.442E-06 | 22.1849241 | 0.0843 | 0.4073 |
| rs7599203 | T | C | 0.0918 | 0.02 | 4.333E-06 | 21.0629802 | 0.032 | 0.6445 |
| rs78682108 | A | G | -0.2383 | 0.0394 | 1.435E-09 | 36.572116 | 0.0583 | 0.9165 |
| **VEGF** | | | | | | | | |
| rs10411345 | C | G | -0.1041 | 0.0218 | 1.733E-06 | 22.7964477 | 0.03 | 0.5997 |
| rs10757514 | C | G | -0.1024 | 0.0222 | 4.169E-06 | 21.2702537 | 0.0329 | 0.6827 |
| rs10822118 | T | C | -0.0797 | 0.0168 | 2.211E-06 | 22.499703 | 0.0251 | 0.9271 |
| rs10934631 | T | C | -0.1132 | 0.0244 | 3.607E-06 | 21.5175047 | 0.0355 | 0.6614 |
| rs114773511 | T | C | 0.2187 | 0.0441 | 6.971E-07 | 24.5866349 | 0.0622 | 0.1161 |
| rs12456390 | T | C | -0.0818 | 0.0179 | 4.882E-06 | 20.8775365 | 0.027 | 0.3537 |
| rs1730969 | C | G | -0.7811 | 0.1696 | 4.106E-06 | 21.2027728 | 0.2848 | 0.516901 |
| rs181031888 | A | T | 0.3767 | 0.0498 | 4.014E-14 | 57.2020062 | 0.0696 | 0.4426 |
| rs2039420 | C | G | 0.0975 | 0.0175 | 2.744E-08 | 31.0321481 | 0.0261 | 0.4082 |
| rs56071907 | T | C | 0.126 | 0.027 | 0.00000301 | 21.7716963 | 0.036 | 0.3642 |
| rs60013354 | A | G | -0.2497 | 0.0521 | 1.662E-06 | 22.9636196 | 0.0742 | 0.630199 |
| rs62401205 | A | C | -0.2015 | 0.0412 | 9.946E-07 | 23.9130171 | 0.051 | 0.4876 |
| rs6496613 | A | C | -0.2359 | 0.0515 | 4.701E-06 | 20.9758771 | 0.0783 | 0.5573 |
| rs7356919 | A | G | -0.1694 | 0.02 | 2.846E-17 | 71.7208662 | 0.0293 | 0.9921 |
| rs73872715 | T | C | -0.6079 | 0.1299 | 2.864E-06 | 21.8939787 | 0.1699 | 0.2041 |
| rs76458389 | T | G | -0.1786 | 0.0363 | 8.573E-07 | 24.2007243 | 0.0543 | 0.1944 |
| rs77961527 | A | G | 0.2289 | 0.0457 | 5.525E-07 | 25.0805982 | 0.0591 | 0.0801309 |
| rs9381249 | T | C | -0.2414 | 0.0396 | 1.038E-09 | 37.1503462 | 0.0621 | 0.1267 |

**Table S11. MR estimates of 41 inflammatory cytokines on myopia.**

|  | **IVW** | | | | **MR-Egger** | | | | **Weighted Median** | | | | **Simple Mode** | | | | **Weighted Mode** | | | |
| --- | --- | --- | --- | --- | --- | --- | --- | --- | --- | --- | --- | --- | --- | --- | --- | --- | --- | --- | --- | --- |
| **Exposures** | **No.of SNPs** | **OR** | **95% CI** | **pval** | **No.of SNPs** | **OR** | **95% CI** | **pval** | **No.of SNPs** | **OR** | **95% CI** | **pval** | **No.of SNPs** | **OR** | **95% CI** | **pval** | **No.of SNPs** | **OR** | **95% CI** | **pval** |
| CTACK | 12 | 0.923 | （0.767-1.112） | 0.402 | 12 | 0.924 | (0.629-1.359) | 0.697 | 12 | 0.970 | (0.755-1.246) | 0.810 | 12 | 0.952 | (0.619-1.465) | 0.829 | 12 | 0.948 | (0.605-1.486) | 0.821 |
| Eotaxin | 17 | 1.156 | (0.954-1.400) | 0.140 | 17 | 1.178 | (0.737-1.882) | 0.504 | 17 | 1.105 | (0.842-1.451) | 0.471 | 17 | 1.124 | (0.722-1.749) | 0.613 | 17 | 1.107 | (0.752-1.631) | 0.612 |
| GROa | 13 | 1.230 | (1.046-1.446) | 0.012 | 13 | 1.095 | (0.823-1.458) | 0.546 | 13 | 1.212 | (0.967-1.520) | 0.096 | 13 | 1.365 | (0.927-2.010) | 0.141 | 13 | 1.242 | (0.853-1.810) | 0.281 |
| IP-10 | 12 | 1.086 | (0.904-1.304) | 0.380 | 12 | 0.985 | (0.641-1.513) | 0.946 | 12 | 1.143 | (0.902-1.448) | 0.268 | 12 | 1.169 | (0.800-1.707) | 0.438 | 12 | 1.174 | (0.815-1.691) | 0.408 |
| MCP-1 | 16 | 1.061 | (0.855-1.318) | 0.589 | 16 | 1.291 | (0.711-2.346) | 0.415 | 16 | 1.113 | (0.842-1.473) | 0.452 | 16 | 1.138 | (0.696-1.861) | 0.613 | 16 | 1.138 | (0.713-1.818) | 0.595 |
| MCP-3 | 6 | 0.899 | (0.769-1.050) | 0.180 | 6 | 0.879 | (0.556-1.389) | 0.610 | 6 | 0.908 | (0.756-1.091) | 0.303 | 6 | 0.816 | (0.582-1.145) | 0.292 | 6 | 0.826 | (0.598-1.142) | 0.300 |
| MIG | 13 | 1.055 | (0.899-1.239) | 0.509 | 13 | 1.013 | (0.726-1.413) | 0.942 | 13 | 1.047 | (0.849-1.291) | 0.700 | 13 | 1.009 | (0.720-1.34413) | 0.961 | 13 | 0.985 | (0.704-1.377) | 0.929 |
| MIP-1A | 4 | 1.040 | (0.761-1.422) | 0.804 | 4 | 1.089 | (0.412-2.882) | 0.879 | 4 | 1.051 | (0.728-1.518) | 0.790 | 4 | 1.108 | (0.701-1.752) | 0.689 | 4 | 1.090 | (0.702-1.693) | 0.727 |
| MIP-1B | 22 | 0.932 | (0.759-1.144) | 0.499 | 22 | 1.137 | (0.633-2.042) | 0.671 | 22 | 0.975 | (0.750-1.268) | 0.849 | 22 | 0.737 | (0.442-1.229) | 0.256 | 22 | 1.066 | (0.703-1.618) | 0.765 |
| RANTES | 10 | 0.929 | (0.768-1.124) | 0.448 | 10 | 0.764 | (0.476-1.225) | 0.296 | 10 | 0.939 | (0.743-1.186) | 0.596 | 10 | 0.955 | (0.641-1.424) | 0.828 | 10 | 0.946 | (0.646-1.385) | 0.782 |
| SDF-1A | 9 | 1.130 | (0.850-1.506) | 0.401 | 9 | 1.591 | (0.897-2.823) | 0.157 | 9 | 0.922 | (0.632-1.346) | 0.674 | 9 | 0.839 | (0.455-1.549) | 0.590 | 9 | 0.868 | (0.486-1.330) | 0.645 |
| SCGF-B | 21 | 1.047 | (0.914-1.199) | 0.507 | 21 | 0.852 | (0.651-1.115) | 0.258 | 21 | 0.931 | (0.780-1.111) | 0.429 | 21 | 0.823 | (0.598-1.131) | 0.244 | 21 | 0.840 | (0.634-1.113) | 0.240 |
| B-NGF | 4 | 1.142 | (0.711-1.834) | 0.583 | 4 | 0.718 | (0.039-1.330) | 0.845 | 4 | 0.962 | (0.6501.423) | 0.846 | 4 | 0.855 | (0.463-1.579) | 0.651 | 4 | 0.841 | (0.523-1.353) | 0.527 |
| FGF2 | 7 | 0.968 | (0.655-1.431) | 0.871 | 7 | 0.445 | (0.157-1.258) | 0.187 | 7 | 1.029 | (0.649-1.633) | 0.903 | 7 | 1.298 | (0.643-2.619) | 0.495 | 7 | 1.264 | (0.662-2.411) | 0.504 |
| G-CSF | 9 | 0.963 | (0.651-1.424) | 0.849 | 9 | 0.700 | (0.357-1.372) | 0.333 | 9 | 1.000 | (0.658-1.519) | 1.000 | 9 | 1.043 | (0.485-2.245) | 0.916 | 9 | 1.083 | (0.487-2.406) | 0.850 |
| HGF | 9 | 0.968 | (0.721-1.299) | 0.829 | 9 | 1.680 | (0.876-3.224) | 0.162 | 9 | 0.862 | (0.569-1.304) | 0.481 | 9 | 0.821 | (0.424-1.588) | 0.574 | 9 | 0.828 | (0.403-1.701) | 0.621 |
| M-CSF | 12 | 0.945 | (0.825-1.082) | 0.803 | 12 | 0.964 | (0.726-1.279) | 0.803 | 12 | 0.997 | (0.826-1.205) | 0.978 | 12 | 1.073 | (0.758-1.519) | 0.699 | 12 | 1.093 | (0.804-1.485) | 0.583 |
| PDGF-BB | 14 | 1.084 | (0.822-1.430) | 0.568 | 14 | 1.323 | (0.676-2.590) | 0.429 | 14 | 1.003 | (0.703-1.432) | 0.986 | 14 | 1.019 | (0.530-1.962) | 0.955 | 14 | 0.989 | (0.462-1.743) | 0.755 |
| SCF | 10 | 1.140 | (0.859-1.513) | 0.364 | 10 | 1.110 | (0.615-2.003) | 0.738 | 10 | 1.065 | (0.730-1.551) | 0.745 | 10 | 1.492 | (0.779-2.860) | 0.259 | 10 | 0.992 | (0.557-1.767) | 0.979 |
| VEGF | 18 | 1.025 | (0.860-1.221) | 0.783 | 18 | 0.900 | (0.615-1.316) | 0.594 | 18 | 1.014 | (0.802-1.283) | 0.906 | 18 | 1.026 | (0.701-1.502) | 0.895 | 18 | 1.008 | (0.712-1.429) | 0.964 |
| IL-10 | 15 | 0.928 | (0.764-1.127) | 0.451 | 15 | 0.654 | (0.349-1.228) | 0.209 | 15 | 0.950 | (0.724-1.247) | 0.712 | 15 | 1.048 | (0.706-1.556) | 0.820 | 15 | 0.948 | (0.700-1.283) | 0.733 |
| IL-12-p70 | 15 | 1.189 | (0.951-1.486) | 0.129 | 15 | 0.966 | (0.432-2.160) | 0.935 | 15 | 1.164 | (0.856-1.582) | 0.333 | 15 | 1.158 | (0.684-1.960) | 0.594 | 15 | 1.163 | (0.721-1.875) | 0.546 |
| IL-13 | 14 | 1.060 | (0.896-1.254) | 0.496 | 14 | 1.172 | (0.850-1.615) | 0.352 | 14 | 1.102 | (0.885-1.371) | 0.386 | 14 | 1.102 | (0.761-1.594) | 0.616 | 14 | 1.121 | (0.788-1.596) | 0.536 |
| IL-16 | 10 | 1.051 | (0.895-1.235) | 0.545 | 10 | 1.164 | (0.895-1.514) | 0.290 | 10 | 1.095 | (0.873-1.374) | 0.432 | 10 | 0.897 | (0.603-1.334) | 0.604 | 10 | 1.231 | (0.882-1.718) | 0.253 |
| IL-17 | 8 | 1.018 | (0.781-1.326) | 0.894 | 8 | 1.115 | (0.682-1.822) | 0.862 | 8 | 1.030 | (0.739-1.436) | 0.862 | 8 | 1.028 | (0.635-1.665) | 0.913 | 8 | 1.131 | (0.761-1.681) | 0.561 |
| IL-18 | 13 | 0.934 | (0.794-1.099) | 0.409 | 13 | 0.819 | (0.599-1.121) | 0.238 | 13 | 0.893 | (0.715-1.156) | 0.319 | 13 | 0.879 | (0.616-1.255) | 0.492 | 13 | 0.879 | (0.623-1.241) | 0.478 |
| IL-1B | 3 | 0.796 | (0.545-1.164) | 0.239 | 3 | 0.752 | (0.306-2.848) | 0.646 | 3 | 0.859 | (0.540-1.164) | 0.521 | 3 | 0.878 | (0.505-1.525) | 0.688 | 3 | 0.875 | (0.529-1.448) | 0.655 |
| IL-1RA | 10 | 0.940 | (0.761-1.161) | 0.568 | 10 | 1.293 | (0.725-2.304) | 0.409 | 10 | 0.925 | (0.711-1.202) | 0.557 | 10 | 0.306 | (0.603-1.360) | 0.645 | 10 | 0.902 | (0.590-1.379) | 0.645 |
| IL-2 | 8 | 0.978 | (0.808-1.188) | 0.835 | 8 | 1.303 | (0.889-1.912) | 0.224 | 8 | 0.985 | (0.760-1.276) | 0.907 | 8 | 0.936 | (0.616-1.423) | 0.766 | 8 | 1.011 | (0.677-1.509) | 0.959 |
| IL-2RA | 9 | 0.897 | (0.736-1.095) | 0.287 | 9 | 1.095 | (0.770-1.558) | 0.628 | 9 | 0.896 | (0.695-1.157) | 0.401 | 9 | 0.873 | (0.526-1.448) | 0.613 | 9 | 0.700 | (0.442-1.106) | 0.165 |
| IL-4 | 14 | 0.903 | (0.689-1.183) | 0.459 | 14 | 0.853 | (0.475-1.533) | 0.605 | 14 | 0.960 | (0.684-1.349) | 0.815 | 14 | 1.012 | (0.555-1.843) | 0.970 | 14 | 1.012 | (0.585-1.748) | 0.967 |
| IL-5 | 8 | 0.969 | (0.768-1.223) | 0.793 | 8 | 0.943 | (0.546-1.629) | 0.840 | 8 | 0.845 | (0.638-1.119) | 0.240 | 8 | 0.781 | (0.480-1.271) | 0.354 | 8 | 0.775 | (0.488-1.231) | 0.316 |
| IL-6 | 11 | 0.850 | (0.647-1.115) | 0.240 | 11 | 0.706 | (0.382-1.305) | 0.295 | 11 | 0.949 | (0.659-1.365) | 0.777 | 11 | 0.954 | (0.544-1.674) | 0.874 | 11 | 0.954 | (0.534-1.705) | 0.878 |
| IL-7 | 12 | 1.000 | (0.842-1.183) | 0.982 | 12 | 0.736 | (0.515-1.053) | 0.125 | 12 | 1.005 | (0.813-1.242) | 0.963 | 12 | 1.027 | (0.693-1.522) | 0.895 | 12 | 0.958 | (0.726-1.262) | 0.764 |
| IL-8 | 8 | 1.012 | (0.797-1.286) | 0.920 | 8 | 0.861 | (0.546-1.359) | 0.545 | 8 | 0.926 | (0.703-1.221) | 0.588 | 8 | 0.903 | (0.645-1.265) | 0.573 | 8 | 0.918 | (0.646-1.304) | 0.647 |
| IL-9 | 6 | 1.252 | (0.957-1.638) | 0.101 | 6 | 1.223 | (0.637-2.347) | 0.578 | 6 | 1.183 | (0.843-1.659) | 0.330 | 6 | 1.125 | (0.691-1.832) | 0.656 | 6 | 1.129 | (0.699-1.825) | 0.641 |
| IFN-G | 12 | 0.845 | (0.629-1.137) | 0.151 | 12 | 0.803 | (0.427-1.511) | 0.512 | 12 | 1.000 | (0.693-1.442) | 0.998 | 12 | 1.116 | (0.618-2.014) | 0.724 | 12 | 1.143 | (0.627-2.085) | 0.671 |
| MIF | 10 | 0.899 | (0.739-1.093) | 0.285 | 10 | 1.014 | (0.690-1.489) | 0.947 | 10 | 0.875 | (0.683-1.120) | 0.288 | 10 | 0.860 | (0.592-1.249) | 0.448 | 10 | 0.864 | (0.621-1.203) | 0.410 |
| TNF-A | 4 | 1.121 | (0.837-1.500) | 0.444 | 4 | 0.991 | (0.628-1.563) | 0.972 | 4 | 1.170 | (0.834-1.642) | 0.363 | 4 | 1.310 | (0.801-2.142) | 0.361 | 4 | 1.296 | (0.770-2.181) | 0.400 |
| TNF-B | 5 | 1.092 | (0.926-1.289) | 0.295 | 5 | 0.925 | (0.661-1.295) | 0.682 | 5 | 1.105 | (0.895-1.365) | 0.352 | 5 | 1.161 | (0.870-1.548) | 0.368 | 5 | 1.130 | (0.849-1.505) | 0.449 |
| TRAIL | 16 | 1.019 | (0.867-1.198) | 0.818 | 16 | 1.060 | (0.831-1.353) | 0.646 | 16 | 1.024 | (0.827-1.238) | 0.828 | 16 | 1.051 | (0.722-1.531) | 0.797 | 16 | 1.058 | (0.867-1.291) | 0.589 |

Abbreviations: CI, Confidence interval; OR, Odds Ratio; pval, P-value; SNPs, single nucleotide polymorphisms. OR and 95% CI represent the change in the odds ratio of myopia per 1 SD increase in inflammatory cytokines.

**Table S12. Heterogeneity and horizontal pleiotropy tests of 41 inflammatory cytokines on myopia.**

| **Exposures** | **Q_1_ pval (IVW)** | **Q_2_ pval (MR-Egger)** | **I^2^** | **intercept** | **intercept pval** |
| --- | --- | --- | --- | --- | --- |
| B-NGF | 0.041 | 0.020 | 63.6% | 0.066 | 0.781 |
| CTACK | 0.354 | 0.276 | 9.3% | -0.000 | 0.996 |
| Eotaxin | 0.677 | 0.607 | 0.0% | -0.003 | 0.931 |
| FGF2 | 0.223 | 0.355 | 27.0% | 0.103 | 0.180 |
| G-CSF | 0.047 | 0.065 | 49.1% | 0.049 | 0.295 |
| GROa | 0.415 | 0.408 | 3.1% | 0.035 | 0.356 |
| HGF | 0.563 | 0.856 | 0.0% | -0.077 | 0.105 |
| IFN-G | 0.115 | 0.081 | 34.4% | 0.007 | 0.859 |
| IL-1B | 0.485 | 0.234 | 0.0% | 0.012 | 0.908 |
| IL-1RA | 0.314 | 0.345 | 14.1% | -0.051 | 0.281 |
| IL-2 | 0.549 | 0.800 | 0.0% | -0.055 | 0.142 |
| IL-2RA | 0.250 | 0.316 | 21.7% | -0.044 | 0.229 |
| IL-4 | 0.217 | 0.167 | 21.8% | 0.008 | 0.833 |
| IL-5 | 0.232 | 0.159 | 24.7% | 0.006 | 0.915 |
| IL-6 | 0.568 | 0.515 | 0.0% | 0.023 | 0.526 |
| IL-7 | 0.210 | 0.375 | 23.8% | 0.067 | 0.095 |
| IL-8 | 0.253 | 0.232 | 22.2% | 0.034 | 0.442 |
| IL-9 | 0.612 | 0.467 | 0.0% | 0.006 | 0.941 |
| IL-10 | 0.662 | 0.695 | 0.0% | 0.040 | 0.273 |
| IL-12p70 | 0.536 | 0.478 | 0.0% | 0.022 | 0.608 |
| IL-13 | 0.205 | 0.183 | 23.0% | -0.025 | 0.483 |
| IL-16 | 0.536 | 0.530 | 0.0% | -0.032 | 0.364 |
| IL-17 | 0.977 | 0.963 | 0.0% | -0.017 | 0.684 |
| IL-18 | 0.453 | 0.445 | 0.0% | 0.027 | 0.358 |
| IP-10 | 0.362 | 0.303 | 8.5% | 0.019 | 0.631 |
| M-CSF | 0.718 | 0.636 | 0.0% | -0.007 | 0.880 |
| MCP-1 | 0.903 | 0.889 | 0.0% | -0.026 | 0.501 |
| MCP-3 | 0.273 | 0.175 | 21.3% | 0.060 | 0.922 |
| MIF | 0.974 | 0.974 | 0.0% | -0.026 | 0.496 |
| MIG | 0.970 | 0.952 | 0.0% | 0.010 | 0.787 |
| MIP-1A | 0.957 | 0.857 | 0.0% | -0.008 | 0.931 |
| MIP-1B | 0.102 | 0.092 | 28.9% | -0.026 | 0.483 |
| PDGF-BB | 0.181 | 0.156 | 25.4% | -0.025 | 0.533 |
| RANTES | 0.812 | 0.813 | 0.0% | 0.040 | 0.401 |
| SCF | 0.631 | 0.531 | 0.0% | 0.004 | 0.922 |
| SCGF-B | 0.240 | 0.346 | 16.8% | 0.047 | 0.103 |
| SDF-1A | 0.461 | 0.551 | 0.0% | -0.046 | 0.220 |
| TNF-A | 0.564 | 0.457 | 0.0% | 0.037 | 0.562 |
| TNF-B | 0.419 | 0.445 | 0.0% | 0.051 | 0.348 |
| TRAIL | 0.337 | 0.285 | 10.2% | -0.009 | 0.671 |
| VEGF | 0.551 | 0.521 | 0.0% | 0.022 | 0.460 |

Q1 pval: P-value of Q test from IVW method; Q2 pval: P-value of Q test from MR-Egger method.

Abbreviations: pval, P-value; Q, Cochran Q statistics; SNPs, single nucleotide polymorphisms; IVW, the inverse variance weighted method.

**Table S13. SNPs information of 41 inflammation cytokines with myopia.**

|  | **Inflammatory cytokines (exposure)** | | | | | **myopia (outcome)** | | |
| --- | --- | --- | --- | --- | --- | --- | --- | --- |
| **SNP** | **effect allele** | **other allele** | **Beta** | **se** | **pval** | **F** | **se** | **pval** |
| **B-NGF** | | | | | | | | |
| rs28637706 | T | G | -0.1554 | 0.0261 | 2.717E-09 | 35.4305296 | 0.0387 | 0.412 |
| rs4767014 | T | C | -0.1211 | 0.0264 | 4.518E-06 | 21.029893 | 0.038 | 0.4701 |
| rs71641308 | T | C | 0.1969 | 0.0429 | 4.424E-06 | 21.0539447 | 0.0605 | 0.2626 |
| rs73472576 | T | C | -0.1146 | 0.0251 | 4.813E-06 | 20.8342764 | 0.036 | 0.01008 |
| **CTACK** | | | | | | | | |
| rs10854859 | A | G | -0.1498 | 0.0293 | 3.049E-07 | 26.1247203 | 0.0427 | 0.3749 |
| rs116303454 | A | G | 0.3754 | 0.081 | 3.579E-06 | 21.4675143 | 0.1118 | 0.8974 |
| rs116871507 | A | T | -0.2086 | 0.0448 | 0.00000316 | 21.668849 | 0.07 | 0.4839 |
| rs116943377 | A | G | 0.2878 | 0.0611 | 2.496E-06 | 22.1749384 | 0.0922 | 0.9136 |
| rs117932939 | T | C | 0.1969 | 0.0422 | 3.089E-06 | 21.7585421 | 0.0644 | 0.9418 |
| rs118084576 | A | G | 0.5675 | 0.1226 | 0.00000366 | 21.4148015 | 0.1977 | 0.4931 |
| rs184329319 | T | G | -0.3069 | 0.0648 | 2.173E-06 | 22.4185246 | 0.1 | 0.2707 |
| rs55764737 | T | C | 0.5424 | 0.0967 | 2.012E-08 | 31.4448608 | 0.1472 | 0.0190498 |
| rs57338032 | A | G | 0.1443 | 0.0316 | 4.831E-06 | 20.8411539 | 0.0477 | 0.9396 |
| rs57789542 | T | C | -0.7687 | 0.1659 | 3.575E-06 | 21.4577535 | 0.2198 | 0.0615106 |
| rs60247384 | T | C | 0.1128 | 0.0245 | 4.302E-06 | 21.1860159 | 0.0372 | 0.6455 |
| rs76395525 | A | G | 0.5193 | 0.1081 | 1.553E-06 | 23.0647377 | 0.1731 | 0.4033 |
| **Eotaxin** | | | | | | | | |
| rs11087905 | A | C | 0.0954 | 0.0188 | 4.07E-07 | 25.7439435 | 0.0377 | 0.5533 |
| rs112347425 | T | C | 0.1595 | 0.0276 | 7.771E-09 | 33.388525 | 0.0622 | 0.8487 |
| rs11920996 | T | C | 0.2979 | 0.0377 | 2.919E-15 | 62.4241056 | 0.0844 | 0.2905 |
| rs1677588 | T | G | 0.1181 | 0.025 | 2.223E-06 | 22.310731 | 0.0559 | 0.2191 |
| rs2024050 | A | G | 0.164 | 0.0302 | 5.467E-08 | 29.4827394 | 0.069 | 0.7449 |
| rs2027855 | T | C | 0.0743 | 0.0162 | 4.272E-06 | 21.0301137 | 0.0365 | 0.7121 |
| rs2040143 | A | G | -0.0858 | 0.0178 | 1.333E-06 | 23.2288973 | 0.0397 | 0.2406 |
| rs2229593 | T | C | 0.3647 | 0.0406 | 2.838E-19 | 80.6702648 | 0.0912 | 0.905 |
| rs2249581 | T | C | -0.0899 | 0.018 | 5.912E-07 | 24.9383891 | 0.0409 | 0.1348 |
| rs5754733 | A | C | -0.105 | 0.0213 | 8.196E-07 | 24.2948048 | 0.0473 | 0.8426 |
| rs57723662 | C | G | -0.0982 | 0.0213 | 3.878E-06 | 21.2499441 | 0.0471 | 0.326 |
| rs60075014 | T | C | -0.1688 | 0.0356 | 2.078E-06 | 22.4770293 | 0.0727 | 0.679499 |
| rs7231030 | A | C | 0.0903 | 0.0193 | 2.709E-06 | 21.885421 | 0.0434 | 0.654699 |
| rs73072941 | A | T | -0.1281 | 0.0263 | 1.113E-06 | 23.7181486 | 0.0556 | 0.0350598 |
| rs745331 | A | G | -0.0821 | 0.0176 | 3.036E-06 | 21.7547953 | 0.0384 | 0.725401 |
| rs75426604 | A | C | -0.1371 | 0.0291 | 2.397E-06 | 22.1913107 | 0.0615 | 0.1326 |
| rs9317045 | A | C | 0.1172 | 0.0236 | 6.954E-07 | 24.6561487 | 0.0506 | 0.8681 |
| **FGF2** | | | | | | | | |
| rs13412535 | A | G | -0.1129 | 0.0224 | 4.763E-07 | 25.3967228 | 0.0449 | 0.9225 |
| rs147409637 | T | C | 0.201 | 0.0431 | 3.077E-06 | 21.7431973 | 0.0957 | 0.4061 |
| rs17094040 | T | C | 0.1051 | 0.0229 | 4.308E-06 | 21.0581541 | 0.0508 | 0.9468 |
| rs2849358 | A | G | 0.0911 | 0.0193 | 2.321E-06 | 22.2745002 | 0.041 | 0.2627 |
| rs4795091 | A | G | 0.1239 | 0.0266 | 3.055E-06 | 21.6902807 | 0.0575 | 0.1861 |
| rs76253061 | T | C | -0.4811 | 0.1041 | 3.807E-06 | 21.3528125 | 0.2241 | 0.0485702 |
| rs78873483 | A | G | 0.1286 | 0.0282 | 0.00000498 | 20.7907211 | 0.0564 | 0.419 |
| **G-CSF** | | | | | | | | |
| rs10939033 | A | G | -0.0775 | 0.0163 | 2.074E-06 | 22.6005443 | 0.0364 | 0.701501 |
| rs117261691 | T | C | 0.1318 | 0.0288 | 4.669E-06 | 20.9380652 | 0.0643 | 0.0502701 |
| rs183023730 | T | G | 0.7898 | 0.1677 | 2.471E-06 | 22.1734096 | 0.301 | 0.3971 |
| rs586802 | A | G | 0.0882 | 0.0187 | 2.398E-06 | 22.2405058 | 0.0414 | 0.1218 |
| rs6740648 | T | C | 0.0818 | 0.0172 | 0.0000019 | 22.6120749 | 0.0381 | 0.7548 |
| rs74148555 | T | C | -0.3771 | 0.0753 | 5.591E-07 | 25.0734338 | 0.1249 | 0.02683 |
| rs76287671 | T | C | 0.0894 | 0.0189 | 2.191E-06 | 22.3687714 | 0.0416 | 0.68 |
| rs77318030 | T | C | -0.2031 | 0.0427 | 0.000002 | 22.618031 | 0.0889 | 0.9067 |
| rs78523761 | A | G | 0.5374 | 0,1139 | 0.0000024 | 22.254212 | 0.2296 | 0.0605899 |
| **GROa** | | | | | | | | |
| rs114991247 | T | C | -0.2202 | 0.0463 | 1.971E-06 | 22.6061961 | 0.0598 | 0.2494 |
| rs115214168 | T | C | 0.4528 | 0.0828 | 4.479E-08 | 29.8886987 | 0.1232 | 0.2873 |
| rs1361829 | A | G | -0.1106 | 0.0241 | 4.576E-06 | 21.0489779 | 0.0358 | 0.0620098 |
| rs140734053 | A | G | 0.7333 | 0.1545 | 2.069E-06 | 22.514445 | 0.1912 | 0.5034 |
| rs150194856 | T | C | -0.4223 | 0.0914 | 3.857E-06 | 21.3355879 | 0.1368 | 0.0492198 |
| rs17171245 | T | G | 0.2446 | 0.053 | 3.932E-06 | 21.287076 | 0.083 | 0.6584 |
| rs185768063 | A | G | 0.4038 | 0.076 | 1.055E-07 | 28.2137159 | 0.111 | 0.0417503 |
| rs188345231 | T | C | 0.6177 | 0.1322 | 2.968E-06 | 21.8196176 | 0.209 | 0.726599 |
| rs3026943 | A | C | -0.1246 | 0.0256 | 1.084E-06 | 23.6761454 | 0.0378 | 0.2796 |
| rs62024303 | A | G | -0.3013 | 0.066 | 4.908E-06 | 20.8288473 | 0.0886 | 0.9881 |
| rs76215157 | C | G | -0.7398 | 0.1564 | 2.226E-06 | 22.3619669 | 0.1785 | 0.4158 |
| rs76390238 | C | G | 0.6223 | 0.1352 | 4.141E-06 | 21.17391 | 0.1905 | 0.1276 |
| rs79454658 | T | C | 0.2784 | 0.0596 | 3.017E-06 | 21.8072442 | 0.0881 | 0.9302 |
| **HGF** | | | | | | | | |
| rs11060254 | A | G | -0.0765 | 0.0166 | 3.974E-06 | 21.2325661 | 0.0382 | 0.1687 |
| rs11129909 | T | C | -0.0738 | 0.0161 | 4.457E-06 | 21.0066482 | 0.0368 | 0.6151 |
| rs13412535 | A | G | -0.1043 | 0.0213 | 9.671E-07 | 23.9720514 | 0.0449 | 0.9225 |
| rs2003620 | T | C | 0.2277 | 0.0487 | 2.978E-06 | 21.8556602 | 0.1063 | 0.6203 |
| rs362307 | T | C | 0.1511 | 0.0328 | 0.00000421 | 21.2166607 | 0.0707 | 0.6848 |
| rs4245058 | T | C | -0.1552 | 0.0331 | 2.683E-06 | 21.9797747 | 0.0735 | 0.6089 |
| rs57146176 | A | G | -0.0987 | 0.0208 | 2.184E-06 | 22.5114477 | 0.0711 | 0.3694 |
| rs5745687 | T | C | -0.3008 | 0.0404 | 9.922E-14 | 55.4228333 | 0.0898 | 0.08179 |
| rs80051150 | T | C | 0.198 | 0.0413 | 1.684E-06 | 22.9787324 | 0.0968 | 0.714 |
| **IFN-G** | | | | | | | | |
| rs113399544 | A | G | -0.0849 | 0.0183 | 3.323E-06 | 21.5179571 | 0.0395 | 0.398 |
| rs113600793 | A | C | 0.1871 | 0.0371 | 4.426E-07 | 25.4265591 | 0.077 | 0.696 |
| rs115729819 | A | G | 0.2511 | 0.0514 | 1.045E-06 | 23.8591531 | 0.11 | 0.8116 |
| rs117046255 | T | C | -0.0968 | 0.0207 | 2.787E-06 | 21.8624013 | 0.0459 | 0.1986 |
| rs11843756 | T | G | 0.1812 | 0.0391 | 3.622E-06 | 21.4709227 | 0.0872 | 0.9752 |
| rs12420286 | T | C | 0.2357 | 0.05 | 2.452E-06 | 22.2160576 | 0.1101 | 0.705801 |
| rs147378920 | A | G | -0.384 | 0.0751 | 3.195E-07 | 26.1378831 | 0.1257 | 0.5079 |
| rs1867282 | T | C | 0.0781 | 0.0166 | 2.478E-06 | 22.1296084 | 0.0362 | 0.4443 |
| rs2073438 | A | G | 0.092 | 0.0188 | 9.551E-07 | 23.9413036 | 0.0407 | 0.0190401 |
| rs7088799 | T | G | -0.0805 | 0.0166 | 1.274E-06 | 23.5105843 | 0.0367 | 0.6623 |
| rs73479333 | C | G | -0.1123 | 0.024 | 2.816E-06 | 21.8889468 | 0.0529 | 0.0379997 |
| rs74148555 | T | C | -0.3771 | 0.077 | 9.858E-07 | 23.9783586 | 0.1249 | 0.02683 |
| **IL-1B** | | | | | | | | |
| rs143319329 | T | C | 0.4357 | 0.093 | 2.835E-06 | 21.9356097 | 0.2199 | 0.1259 |
| rs4786740 | A | C | 0.1264 | 0.0265 | 0.00000182 | 22.7375092 | 0.0366 | 0.5097 |
| rs61335305 | A | C | 0.4333 | 0.0928 | 3.015E-06 | 21.7882281 | 0.1314 | 0.8132 |
| **IL-1RA** | | | | | | | | |
| rs1054402 | T | C | 0.1325 | 0.0269 | 8.201E-07 | 24.2488368 | 0.0411 | 0.7133 |
| rs117181659 | A | G | -0.2204 | 0.0478 | 0.00000392 | 21.2486381 | 0.0727 | 0.4542 |
| rs11869294 | C | G | -0.2286 | 0.047 | 1.128E-06 | 23.6439727 | 0.0667 | 0.4649 |
| rs13343438 | A | G | 0.2771 | 0.0607 | 4.973E-06 | 20.8285666 | 0.094 | 0.9503 |
| rs35590641 | C | G | -0.1167 | 0.025 | 3.041E-06 | 21.7783718 | 0.0377 | 0.3243 |
| rs3876037 | A | G | 0.1234 | 0.027 | 4.733E-06 | 20.8769237 | 0.0371 | 0.3705 |
| rs56134659 | A | G | -0.1109 | 0.0236 | 2.564E-06 | 22.0700237 | 0.0357 | 0.7873 |
| rs61335305 | A | C | 0.4315 | 0.0904 | 1.812E-06 | 22.7713329 | 0.1314 | 0.8132 |
| rs6699436 | A | G | -0.1858 | 0.0404 | 4.365E-06 | 21.1393876 | 0.0572 | 0.2115 |
| rs9985296 | T | C | 0.1053 | 0.0231 | 4.952E-06 | 20.7680871 | 0.0357 | 0.0129799 |
| **IL-2** | | | | | | | | |
| rs13412535 | A | G | 0.174 | 0.0331 | 1.447E-07 | 27.6181929 | 0.0449 | 0.9225 |
| rs16836080 | A | G | 0.1158 | 0.0253 | 4.841E-06 | 20.9376929 | 0.0381 | 0.6406 |
| rs2690020 | A | G | 0.1158 | 0.0245 | 2.273E-06 | 22.3273767 | 0.0359 | 0.6675 |
| rs4479767 | A | G | 0.1821 | 0.0392 | 3.444E-06 | 21.5675348 | 0.0614 | 0.713099 |
| rs4634519 | A | G | -0.1249 | 0.0268 | 0.00000318 | 21.7074115 | 0.0399 | 0.1543 |
| rs61335305 | A | C | 0.4439 | 0.0913 | 1.157E-06 | 23.6255259 | 0.1314 | 0.8132 |
| rs62124990 | T | G | -0.7013 | 0.149 | 2.502E-06 | 22.1405238 | 0.1312 | 0.1567 |
| rs7615304 | A | G | -0.1139 | 0.024 | 2.161E-06 | 22.5101151 | 0.0357 | 0.25 |
| **IL-2RA** | | | | | | | | |
| rs11241559 | T | G | -0.124 | 0.0264 | 0.00000275 | 22.0496505 | 0.0408 | 0.6201 |
| rs117244812 | A | G | -0.7187 | 0.1493 | 1.474E-06 | 23.1601767 | 0.2173 | 0.3868 |
| rs12789243 | T | C | 0.1263 | 0.0276 | 4.608E-06 | 20.9293013 | 0.0413 | 0.9094 |
| rs17147986 | A | C | -0.2976 | 0.0337 | 1.069E-18 | 77.9421257 | 0.0509 | 0.0136399 |
| rs17624670 | A | G | -0.125 | 0.0273 | 4.639E-06 | 20.9537099 | 0.0411 | 0.8057 |
| rs34037190 | A | G | 0.4784 | 0.0935 | 3.134E-07 | 26.1652756 | 0.1088 | 0.1532 |
| rs56213152 | T | C | 0.1269 | 0.0271 | 2.937E-06 | 21.9154734 | 0.0416 | 0.263 |
| rs759244 | A | T | -0.1094 | 0.0238 | 4.257E-06 | 21.117715 | 0.0365 | 0.2818 |
| rs79100208 | C | G | 0.8345 | 0.1758 | 2.052E-06 | 22.5206611 | 0.2234 | 0.7787 |
| **IL-4** | | | | | | | | |
| rs116705532 | T | G | -0.4675 | 0.0978 | 1.727E-06 | 22.844369 | 0.209 | 0.730801 |
| rs117146485 | T | C | -0.2856 | 0.0625 | 4.945E-06 | 20.8761312 | 0.1326 | 0.0370502 |
| rs12238729 | T | C | 0.5271 | 0.1096 | 1.505E-06 | 23.116432 | 0.2864 | 0.7877 |
| rs12640583 | T | G | -0.1104 | 0.0214 | 2.451E-07 | 26.6075108 | 0.0478 | 0.9252 |
| rs17713451 | A | G | 0.1255 | 0.0252 | 6.41E-07 | 24.795908 | 0.0562 | 0.1224 |
| rs1867282 | T | C | 0.0808 | 0.0162 | 5.822E-07 | 24.8706044 | 0.0362 | 0.4443 |
| rs2073438 | A | G | 0.0847 | 0.0183 | 3.725E-06 | 21.4169828 | 0.0407 | 0.0190401 |
| rs2346020 | A | G | 0.079 | 0.0169 | 2.843E-06 | 21.8461253 | 0.038 | 0.8663 |
| rs2708586 | T | C | -0.0767 | 0.0166 | 3.588E-06 | 21.3436258 | 0.0377 | 0.4677 |
| rs56408830 | A | G | -0.1794 | 0.0365 | 9.075E-07 | 24.151983 | 0.0864 | 0.2821 |
| rs7613691 | A | G | 0.1787 | 0.0382 | 2.962E-06 | 21.8784235 | 0.0827 | 0.3195 |
| rs79597994 | T | C | -0.5855 | 0.1271 | 4.056E-06 | 21.2156501 | 0.2233 | 0.9226 |
| rs9506111 | A | G | -0.1446 | 0.0314 | 4.081E-06 | 21.2017123 | 0.0708 | 0.8223 |
| rs9941733 | A | G | 0.1156 | 0.0229 | 4.331E-07 | 25.4764171 | 0.0472 | 0.215 |
| **IL-5** | | | | | | | | |
| rs10178043 | T | G | 0.2579 | 0.0553 | 3.126E-06 | 21.7368748 | 0.0809 | 0.8233 |
| rs148634917 | A | G | -0.517 | 0.1087 | 1.974E-06 | 22.6082361 | 0.1323 | 0.2152 |
| rs28793375 | T | C | 0.1697 | 0.0362 | 2.746E-06 | 21.962978 | 0.0524 | 0.525801 |
| rs72831687 | A | G | -0.5337 | 0.1104 | 1.324E-06 | 23.3561331 | 0.1406 | 0.1385 |
| rs73040118 | T | C | 0.2294 | 0.049 | 2.903E-06 | 21.9048029 | 0.0735 | 0.4122 |
| rs74811276 | A | G | 0.217 | 0.0471 | 4.082E-06 | 21.2139889 | 0.0644 | 0.1841 |
| rs7739450 | A | G | -0.1295 | 0.0256 | 4.05E-07 | 25.5743314 | 0.036 | 0.3513 |
| rs9309063 | T | G | -0.1119 | 0.0245 | 4.866E-06 | 20.8483979 | 0.0357 | 0.1684 |
| **IL-6** | | | | | | | | |
| rs10910395 | A | T | -0.108 | 0.0235 | 4.348E-06 | 21.1157384 | 0.0516 | 0.706999 |
| rs10982193 | A | G | -0.0793 | 0.0174 | 4.816E-06 | 20.7654986 | 0.0399 | 0.9151 |
| rs113098456 | A | G | -0.1553 | 0.0339 | 4.641E-06 | 20.9815709 | 0.0688 | 0.6035 |
| rs113600793 | A | C | 0.1736 | 0.0359 | 1.291E-06 | 23.3778749 | 0.077 | 0.696 |
| rs114373846 | T | C | 0.4196 | 0.0905 | 3.568E-06 | 21.4915771 | 0.2121 | 0.9099 |
| rs11732981 | A | C | 0.0722 | 0.0156 | 3.793E-06 | 21.4150792 | 0.0356 | 0.0435201 |
| rs1333040 | T | C | 0.0747 | 0.0157 | 1.993E-06 | 22.632701 | 0.0357 | 0.1904 |
| rs13412535 | A | G | -0.1186 | 0.0214 | 3.141E-08 | 30.7069242 | 0.0449 | 0.9225 |
| rs4684700 | T | C | -0.0747 | 0.0162 | 3.912E-06 | 21.2571805 | 0.0358 | 0.9828 |
| rs73273528 | T | C | 0.268 | 0.0553 | 0.00000125 | 23.4808499 | 0.1179 | 0.0906108 |
| rs76856708 | T | C | 0.336 | 0.0697 | 1.427E-06 | 23.2331173 | 0.1569 | 0.3806 |
| **IL-7** | | | | | | | | |
| rs117509142 | T | C | -0.3213 | 0.0684 | 2.599E-06 | 22.0524712 | 0.0984 | 0.2546 |
| rs11757972 | T | C | 0.121 | 0.0257 | 2.529E-06 | 22.1540183 | 0.0359 | 0.0695905 |
| rs1374279 | A | T | 0.1625 | 0.0347 | 2.792E-06 | 21.9177413 | 0.0505 | 0.2003 |
| rs142397827 | A | C | 0.4592 | 0.0994 | 3.822E-06 | 21.329418 | 0.1253 | 0.2246 |
| rs17091524 | T | C | 0.5092 | 0.1015 | 5.244E-07 | 25.1531697 | 0.1371 | 0.8754 |
| rs2006957 | T | C | 0.2557 | 0.0262 | 1.434E-22 | 95.1934111 | 0.0378 | 0.4556 |
| rs218238 | A | T | 0.1319 | 0.0284 | 3.277E-06 | 21.5576257 | 0.0424 | 0.8673 |
| rs28793375 | T | C | 0.1644 | 0.036 | 4.866E-06 | 20.8423479 | 0.0524 | 0.525801 |
| rs62006410 | T | C | -0.1492 | 0.0302 | 7.588E-07 | 24.3933685 | 0.0418 | 0.3696 |
| rs7155170 | A | T | -0.1236 | 0.027 | 4.787E-06 | 20.9438939 | 0.0398 | 0.9887 |
| rs77318030 | T | C | -0.2966 | 0.0631 | 2.639E-06 | 22.0816535 | 0.0889 | 0.9067 |
| rs77981494 | T | C | -0.5201 | 0.1055 | 8.225E-07 | 24.289401 | 0.1281 | 0.0266097 |
| **IL-8** | | | | | | | | |
| rs113487695 | A | C | -0.6129 | 0.1292 | 2.092E-06 | 22.49109 | 0.2004 | 0.9571 |
| rs116726256 | T | C | -0.2247 | 0.0489 | 4.261E-06 | 21.1030251 | 0.0753 | 0.4738 |
| rs12075 | A | G | 0.1148 | 0.0235 | 9.969E-07 | 23.8508763 | 0.0357 | 0.0058341 |
| rs12912642 | A | G | 0.1168 | 0.0251 | 3.212E-06 | 21.6418574 | 0.0376 | 0.528901 |
| rs183628733 | T | C | 0.6547 | 0.1417 | 3.821E-06 | 21.3354236 | 0.1736 | 0.737901 |
| rs2673604 | A | C | -0.118 | 0.0254 | 3.289E-06 | 21.5701353 | 0.0384 | 0.579101 |
| rs3786107 | A | G | 0.2463 | 0.0517 | 1.935E-06 | 22.6831845 | 0.0685 | 0.8156 |
| rs75840288 | A | C | 0.5125 | 0.1121 | 4.846E-06 | 20.8897288 | 0.1516 | 0.9402 |
| **IL-9** | | | | | | | | |
| rs117807175 | C | G | -0.5225 | 0.1106 | 2.327E-06 | 22.3062103 | 0.1613 | 0.8805 |
| rs1259728 | A | G | -0.2381 | 0.0507 | 2.599E-06 | 22.0427781 | 0.0769 | 0.1891 |
| rs3736858 | C | G | -0.1351 | 0.0291 | 3.373E-06 | 21.5421069 | 0.0442 | 0.506001 |
| rs41294750 | T | C | 0.3442 | 0.0736 | 2.916E-06 | 21.8589632 | 0.1057 | 0.0527704 |
| rs4880409 | T | C | -0.3552 | 0.0716 | 6.952E-07 | 24.5970671 | 0.1773 | 0.5677 |
| rs73443903 | A | C | 0.2162 | 0.046 | 2.569E-06 | 22.0779717 | 0.0713 | 0.9398 |
| **IL-10** | | | | | | | | |
| rs10457128 | A | G | -0.0854 | 0.0172 | 6.956E-07 | 24.6459963 | 0.0371 | 0.3625 |
| rs10493718 | A | C | -0.1081 | 0.0222 | 1.068E-06 | 23.7046186 | 0.0489 | 0.1473 |
| rs13412535 | A | G | -0.1347 | 0.0224 | 1.798E-09 | 36.1515304 | 0.0449 | 0.9225 |
| rs1530455 | T | C | 0.082 | 0.0174 | 2.527E-06 | 22.2032598 | 0.0373 | 0.973 |
| rs2086656 | T | C | -0.08 | 0.017 | 2.589E-06 | 22.1395946 | 0.0376 | 0.9964 |
| rs3002131 | C | G | 0.1191 | 0.026 | 4.592E-06 | 20.9780134 | 0.0523 | 0.2415 |
| rs3025021 | T | C | 0.0913 | 0.0194 | 2.609E-06 | 22.1424477 | 0.0378 | 0.9315 |
| rs383684 | A | G | 0.092 | 0.0197 | 3.168E-06 | 21.8037269 | 0.0629 | 0.0699294 |
| rs4741748 | A | G | -0.0788 | 0.0169 | 3.202E-06 | 21.735346 | 0.0366 | 0.847 |
| rs6054847 | T | C | 0.0971 | 0.0207 | 2.752E-06 | 21.9981065 | 0.0454 | 0.6268 |
| rs6680918 | T | C | -0.1202 | 0.025 | 1.591E-06 | 23.1108783 | 0.0546 | 0.116 |
| rs7088799 | T | G | -0.0815 | 0.0166 | 9.352E-07 | 24.0983093 | 0.0367 | 0.6623 |
| rs73192842 | A | G | 0.0949 | 0.0206 | 4.031E-06 | 21.2170752 | 0.0456 | 0.655101 |
| rs7747448 | A | G | -0.1061 | 0.0189 | 1.998E-08 | 31.5061032 | 0.0407 | 0.4069 |
| rs9472173 | T | C | -0.2004 | 0.0174 | 1.256E-30 | 132.612502 | 0.0365 | 0.5049 |
| **IL-12p70** |  |  |  |  |  |  |  |  |
| rs113600793 | A | C | 0.1832 | 0.0359 | 3.351E-07 | 26.0349984 | 0.077 | 0.696 |
| rs12969892 | T | C | 0.1227 | 0.0267 | 4.194E-06 | 21.1135916 | 0.0592 | 0.776599 |
| rs2123852 | T | C | 0.0942 | 0.0204 | 0.00000373 | 21.317535 | 0.0447 | 0.6801 |
| rs273702 | A | G | -0.127 | 0.027 | 2.522E-06 | 22.1195062 | 0.0595 | 0.3101 |
| rs282258 | T | C | 0.0726 | 0.0156 | 3.282E-06 | 21.6530739 | 0.036 | 0.1744 |
| rs34322762 | T | C | 0.0953 | 0.0199 | 1.708E-06 | 22.9267052 | 0.0373 | 0.0199499 |
| rs34826779 | T | G | -0.0884 | 0.019 | 3.327E-06 | 21.6417733 | 0.0432 | 0.346 |
| rs41282644 | A | G | 0.1401 | 0.0303 | 3.737E-06 | 21.3740356 | 0.0657 | 0.9398 |
| rs4530855 | T | G | 0.0861 | 0.0184 | 2.872E-06 | 21.8910287 | 0.0393 | 0.0360296 |
| rs4741748 | A | G | -0.0799 | 0.0163 | 9.162E-07 | 24.0222601 | 0.0366 | 0.847 |
| rs6532374 | T | C | -0.1033 | 0.0226 | 4.613E-06 | 20.8871545 | 0.0507 | 0.673 |
| rs7754905 | A | G | -0.1005 | 0.019 | 1.14E-07 | 27.9718014 | 0.0425 | 0.783401 |
| rs782111 | A | C | -0.0765 | 0.0156 | 9.247E-07 | 24.0419222 | 0.0356 | 0.571 |
| rs865585 | A | C | -0.1654 | 0.0237 | 2.732E-12 | 48.69337 | 0.0505 | 0.5532 |
| rs9381249 | T | C | -0.1788 | 0.0367 | 1.125E-06 | 23.730037 | 0.0874 | 0.6214 |
| **IL-13** | | | | | | | | |
| rs10995604 | A | G | -0.1571 | 0.0343 | 4.482E-06 | 20.966335 | 0.0503 | 0.8909 |
| rs117795020 | A | G | -0.3584 | 0.0716 | 5.479E-07 | 25.0419617 | 0.1055 | 0.574101 |
| rs12623722 | A | G | -0.1189 | 0.0257 | 3.614E-06 | 21.3922198 | 0.0385 | 0.792899 |
| rs138854806 | A | G | -0.4204 | 0.0839 | 5.449E-07 | 25.0934216 | 0.1058 | 0.3835 |
| rs139083458 | T | C | 0.9995 | 0.211 | 2.165E-06 | 22.4263748 | 0.3083 | 0.2988 |
| rs147747784 | C | G | 0.369 | 0.0765 | 1.435E-06 | 23.2534958 | 0.0846 | 0.4946 |
| rs150836197 | T | C | 0.3283 | 0.0713 | 4.136E-06 | 21.1895189 | 0.0896 | 0.1634 |
| rs27949 | T | C | -0.1144 | 0.025 | 4.827E-06 | 20.9281299 | 0.0381 | 0.9773 |
| rs28442067 | A | G | -0.1379 | 0.0286 | 1.413E-06 | 23.2356516 | 0.0434 | 0.7304 |
| rs7073807 | T | C | 0.1618 | 0.0354 | 0.00000477 | 20.8789618 | 0.0527 | 0.1918 |
| rs75383097 | C | G | -0.5369 | 0.116 | 3.702E-06 | 21.4106188 | 0.146 | 0.0069019 |
| rs76339001 | A | T | -0.4375 | 0.0886 | 7.915E-07 | 24.3695248 | 0.1079 | 0.0716193 |
| rs76975337 | T | C | -0.1211 | 0.0265 | 4.921E-06 | 20.8715608 | 0.0403 | 0.752101 |
| rs77955971 | A | C | 0.4408 | 0.0868 | 3.756E-07 | 25.775204 | 0.0993 | 0.5475 |
| **IL-16** | | | | | | | | |
| rs117217798 | T | C | -0.2064 | 0.044 | 2.772E-06 | 21.992129 | 0.0644 | 0.2092 |
| rs12577604 | T | C | 0.4335 | 0.0941 | 4.083E-06 | 21.2105631 | 0.1369 | 0.7451 |
| rs142034902 | A | G | -0.4367 | 0.0925 | 2.327E-06 | 22.2759462 | 0.1408 | 0.3992 |
| rs142332135 | A | G | -0.7646 | 0.1082 | 1.581E-12 | 49.9076715 | 0.1375 | 0.1173 |
| rs144691581 | A | G | 0.4929 | 0.0958 | 2.668E-07 | 26.4569551 | 0.123 | 0.8408 |
| rs35834666 | T | C | -0.1729 | 0.0348 | 6.57E-07 | 24.6708857 | 0.0504 | 0.1901 |
| rs4778640 | A | G | 0.7189 | 0.0983 | 2.552E-13 | 53.4543648 | 0.1429 | 0.3139 |
| rs4976691 | C | G | 0.1254 | 0.026 | 1.473E-06 | 23.2488577 | 0.0381 | 0.48 |
| rs7097884 | T | C | -0.1193 | 0.0243 | 8.809E-07 | 24.0891559 | 0.0361 | 0.6569 |
| rs78042619 | A | G | 0.55 | 0.1158 | 2.023E-06 | 22.5455847 | 0.1561 | 0.8609 |
| **IL-17** | | | | | | | | |
| rs11985957 | A | G | 0.1511 | 0.0329 | 4.363E-06 | 21.0875351 | 0.0763 | 0.6468 |
| rs12735700 | T | G | -0.0943 | 0.0206 | 0.0000045 | 20.9496914 | 0.0436 | 0.9428 |
| rs145006174 | C | G | -0.2266 | 0.0473 | 1.645E-06 | 22.9449024 | 0.1028 | 0.6393 |
| rs17282552 | T | C | -0.2026 | 0.0403 | 4.876E-07 | 25.2672208 | 0.0814 | 0.4686 |
| rs3792369 | A | G | 0.0941 | 0.0166 | 1.46E-08 | 32.1256376 | 0.0366 | 0.9965 |
| rs61990749 | C | G | 0.1124 | 0.0226 | 6.569E-07 | 24.728879 | 0.0496 | 0.9789 |
| rs78296352 | T | G | 0.2949 | 0.0645 | 4.809E-06 | 20.8986774 | 0.1549 | 0.705801 |
| rs9519328 | A | G | 0.5256 | 0.1101 | 0.00000179 | 22.7767495 | 0.1179 | 0.4591 |
| **IL-18** | | | | | | | | |
| rs10409850 | A | G | 0.1791 | 0.0347 | 2.44E-07 | 26.6253795 | 0.0522 | 0.7524 |
| rs11214093 | T | C | 0.1143 | 0.0238 | 1.544E-06 | 23.0516561 | 0.0359 | 0.08127 |
| rs117266781 | T | C | 0.7051 | 0.1436 | 9.176E-07 | 24.0966203 | 0.1947 | 0.1982 |
| rs117371668 | T | G | 0.3712 | 0.0799 | 3.357E-06 | 21.571779 | 0.1209 | 0.632501 |
| rs139468359 | T | C | 0.5101 | 0.1088 | 2.737E-06 | 21.9693112 | 0.1603 | 0.2883 |
| rs1979967 | T | C | 0.14 | 0.0285 | 8.719E-07 | 24.1173694 | 0.0433 | 0.593401 |
| rs4952239 | A | T | -0.1156 | 0.0242 | 1.809E-06 | 22.8059686 | 0.0369 | 0.7504 |
| rs58701153 | A | T | -0.1265 | 0.0242 | 1.807E-07 | 27.3095098 | 0.0373 | 0.9498 |
| rs62312914 | T | C | -0.1265 | 0.025 | 4.215E-07 | 25.5896661 | 0.0369 | 0.0680707 |
| rs764078 | A | T | 0.1283 | 0.0278 | 4.075E-06 | 21.2876296 | 0.0422 | 0.2343 |
| rs77187209 | T | C | -0.4859 | 0.1041 | 3.082E-06 | 21.7748877 | 0.1506 | 0.4019 |
| rs78623212 | T | C | 0.8322 | 0.1676 | 6.82E-07 | 24.6417178 | 0.2027 | 0.4739 |
| rs78716465 | A | G | 0.3173 | 0.0679 | 2.981E-06 | 21.8254989 | 0.0964 | 0.774 |
| **IP-10** | | | | | | | | |
| rs113183470 | A | T | -0.2414 | 0.0524 | 4.147E-06 | 21.2118552 | 0.0755 | 0.7624 |
| rs12714300 | A | T | -0.1573 | 0.0338 | 3.297E-06 | 21.6466523 | 0.0512 | 0.0518096 |
| rs143799975 | A | G | -0.7551 | 0.1638 | 4.012E-06 | 21.2396738 | 0.2167 | 0.697199 |
| rs34383175 | T | C | -0.3196 | 0.0653 | 9.904E-07 | 23.9416388 | 0.0979 | 0.0316701 |
| rs397816 | T | C | 0.1211 | 0.0248 | 1.026E-06 | 23.8315133 | 0.0371 | 0.2669 |
| rs4859940 | C | G | -0.1204 | 0.0258 | 3.228E-06 | 21.7660819 | 0.039 | 0.4341 |
| rs4862110 | T | C | -0.1453 | 0.0318 | 4.909E-06 | 20.8662153 | 0.0413 | 0.6107 |
| rs75970138 | A | G | -0.4845 | 0.1037 | 2.994E-06 | 21.8170861 | 0.1629 | 0.7175 |
| rs7645625 | T | G | -0.1116 | 0.0236 | 2.192E-06 | 22.3496682 | 0.0363 | 0.5115 |
| rs78077394 | T | C | -0.3486 | 0.0701 | 6.474E-07 | 24.716412 | 0.0973 | 0.2581 |
| rs79848609 | A | C | 0.2514 | 0.0535 | 2.637E-06 | 22.069357 | 0.0825 | 0.628001 |
| rs8112618 | A | G | 0.1388 | 0.0297 | 3.047E-06 | 21.8289442 | 0.0449 | 0.8207 |
| **M-CSF** | | | | | | | | |
| rs116274860 | T | G | 0.8262 | 0.1739 | 2.029E-06 | 22.553989 | 0.1566 | 0.1008 |
| rs116887628 | A | G | -0.2741 | 0.0598 | 4.626E-06 | 20.9926783 | 0.0698 | 0.997 |
| rs117867915 | T | C | 0.5224 | 0.1096 | 1.874E-06 | 22.700602 | 0.1302 | 0.508899 |
| rs11963606 | C | G | -0.5353 | 0.117 | 4.731E-06 | 20.9158207 | 0.1433 | 0.5977 |
| rs12962919 | T | C | 0.3025 | 0.0659 | 4.394E-06 | 21.0538767 | 0.0704 | 0.8138 |
| rs139457375 | A | C | -0.4047 | 0.0854 | 2.142E-06 | 22.4389646 | 0.1035 | 0.3598 |
| rs147378920 | A | G | -0.6064 | 0.1318 | 4.177E-06 | 21.1514289 | 0.1257 | 0.5079 |
| rs34089869 | T | C | 0.2194 | 0.0462 | 2.078E-06 | 22.5341636 | 0.0575 | 0.6878 |
| rs62294910 | A | G | 0.3472 | 0.0687 | 4.378E-07 | 25.5210246 | 0.0737 | 0.578599 |
| rs72723242 | T | G | -0.4969 | 0.1083 | 4.434E-06 | 21.0345501 | 0.1399 | 0.6612 |
| rs9387100 | T | C | -0.135 | 0.029 | 3.341E-06 | 21.6532798 | 0.036 | 0.4547 |
| rs9626985 | T | C | 0.2277 | 0.0496 | 4.482E-06 | 21.0578901 | 0.0606 | 0.1023 |
| **MCP-1** | | | | | | | | |
| rs111995966 | T | G | 0.1428 | 0.0309 | 3.788E-06 | 21.3518377 | 0.067 | 0.6067 |
| rs11920996 | T | C | 0.1805 | 0.0376 | 1.604E-06 | 23.0395783 | 0.0844 | 0.2905 |
| rs12062235 | T | G | 0.1477 | 0.032 | 3.836E-06 | 21.2988834 | 0.0728 | 0.547599 |
| rs143815843 | A | G | -0.2049 | 0.0447 | 4.609E-06 | 21.0070308 | 0.1042 | 0.4732 |
| rs16837903 | A | G | -0.1104 | 0.0238 | 3.352E-06 | 21.5119627 | 0.0523 | 0.3678 |
| rs2201150 | T | C | 0.0916 | 0.016 | 1.044E-08 | 32.7677623 | 0.0368 | 0.1239 |
| rs2229593 | T | C | 0.2624 | 0.0405 | 9.246E-11 | 41.9675308 | 0.0912 | 0.905 |
| rs56212190 | T | C | 0.1799 | 0.0372 | 1.318E-06 | 23.3814936 | 0.0824 | 0.5952 |
| rs62245103 | T | G | 0.2433 | 0.0416 | 4.989E-09 | 34.1974213 | 0.0872 | 0.533301 |
| rs7197349 | A | G | 0.0971 | 0.0206 | 2.399E-06 | 22.2126218 | 0.0448 | 0.1647 |
| rs72705803 | A | G | -0.2188 | 0.047 | 3.222E-06 | 21.6667974 | 0.1116 | 0.5011 |
| rs7978037 | A | T | 0.0746 | 0.016 | 3.043E-06 | 21.7336912 | 0.0367 | 0.9788 |
| rs79939301 | A | G | 0.1449 | 0.0255 | 1.356E-08 | 32.281389 | 0.056 | 0.687299 |
| rs856100 | A | G | 0.0899 | 0.0191 | 2.545E-06 | 22.1487108 | 0.0433 | 0.9762 |
| rs862990 | T | C | -0.089 | 0.0183 | 1.153E-06 | 23.6468685 | 0.041 | 0.779301 |
| rs9317045 | A | C | 0.1157 | 0.0235 | 8.425E-07 | 24.2340944 | 0.0506 | 0.8681 |
| **MCP-3** | | | | | | | | |
| rs10892381 | T | C | 0.2432 | 0.0473 | 2.693E-07 | 26.3884447 | 0.0379 | 0.0335498 |
| rs117286643 | A | G | 0.6934 | 0.1474 | 2.542E-06 | 22.0892842 | 0.1202 | 0.3184 |
| rs28394764 | A | T | 0.597 | 0.1282 | 3.194E-06 | 21.646194 | 0.1019 | 0.913 |
| rs3129806 | T | C | -0.1975 | 0.0433 | 4.978E-06 | 20.766693 | 0.0361 | 0.148 |
| rs6993671 | T | C | 0.2041 | 0.0443 | 4.061E-06 | 21.1878794 | 0.0366 | 0.3158 |
| rs7275485 | T | C | -0.2218 | 0.0481 | 3.986E-06 | 21.2247149 | 0.0397 | 0.8713 |
| **MIF** | | | | | | | | |
| rs1007888 | T | C | -0.1275 | 0.0245 | 1.915E-07 | 27.0671345 | 0.0366 | 0.2055 |
| rs113218956 | A | G | -0.8789 | 0.1876 | 2.815E-06 | 21.9365002 | 0.2785 | 0.5615 |
| rs11551183 | C | G | 0.3666 | 0.0795 | 3.999E-06 | 21.2522416 | 0.1218 | 0.569 |
| rs12594190 | A | G | 0.1321 | 0.0266 | 6.85E-07 | 24.6488375 | 0.039 | 0.5206 |
| rs141009259 | T | C | -0.6194 | 0.1285 | 1.444E-06 | 23.2214977 | 0.1687 | 0.6441 |
| rs2294689 | C | G | -0.1338 | 0.0287 | 3.043E-06 | 21.7221353 | 0.1491 | 0.4759 |
| rs35792361 | A | G | -0.2586 | 0.0527 | 9.003E-07 | 24.065223 | 0.0763 | 0.8561 |
| rs35890933 | T | G | 0.1676 | 0.0365 | 4.458E-06 | 21.0725154 | 0.0508 | 0.7001 |
| rs3814097 | A | G | -0.1163 | 0.0251 | 3.548E-06 | 21.456855 | 0.0363 | 0.632501 |
| rs78098071 | T | C | -0.4583 | 0.0915 | 5.509E-07 | 25.0733063 | 0.1397 | 0.8167 |
| **MIG** |  |  |  |  |  |  |  |  |
| rs10266753 | T | C | -0.2016 | 0.0397 | 3.769E-07 | 25.7731047 | 0.0635 | 0.8324 |
| rs111607343 | A | G | -0.5235 | 0.1119 | 2.928E-06 | 21.8745887 | 0.1547 | 0.7895 |
| rs11177248 | A | G | 0.3157 | 0.0667 | 2.222E-06 | 22.3905207 | 0.0915 | 0.5399 |
| rs113302091 | T | C | 0.2537 | 0.0553 | 4.402E-06 | 21.0357194 | 0.085 | 0.3716 |
| rs13143163 | C | G | 0.2735 | 0.0582 | 2.622E-06 | 22.0716799 | 0.0775 | 0.7149 |
| rs139010077 | T | C | 0.4337 | 0.0943 | 4.193E-06 | 21.1408359 | 0.1394 | 0.1568 |
| rs191555775 | A | T | 0.2279 | 0.0412 | 3.278E-08 | 30.5816501 | 0.0593 | 0.8951 |
| rs192433162 | A | G | -0.8045 | 0.1676 | 1.594E-06 | 23.0287712 | 0.2423 | 0.9072 |
| rs3733233 | T | C | 0.1223 | 0.025 | 1.049E-06 | 23.9188113 | 0.0374 | 0.5632 |
| rs62562991 | A | G | 0.6239 | 0.1259 | 7.237E-07 | 24.5439924 | 0.1476 | 0.5904 |
| rs6679677 | A | C | 0.1628 | 0.0327 | 6.514E-07 | 24.7730793 | 0.0499 | 0.792 |
| rs8127917 | T | G | 0.2382 | 0.0492 | 1.278E-06 | 23.4271795 | 0.0754 | 0.3895 |
| rs816960 | T | C | -0.1179 | 0.0242 | 0.00000115 | 23.7226704 | 0.037 | 0.6934 |
| **MIP-1A** | | | | | | | | |
| rs117506943 | T | C | 0.0108 | 0.0682 | 4.484E-06 | 21.0242617 | 0.0905 | 0.9812 |
| rs12159394 | A | G | 0.0008 | 0.0366 | 3.112E-06 | 21.7655465 | 0.0556 | 0.6538 |
| rs57786342 | A | G | 0.0149 | 0.0283 | 8.909E-07 | 24.1108624 | 0.0473 | 0.749 |
| rs6956239 | T | C | -0.0088 | 0.026 | 4.583E-06 | 20.9364595 | 0.0391 | 0.7813 |
| **MIP-1B** | | | | | | | | |
| rs111721971 | T | G | -0.227 | 0.047 | 1.387E-06 | 23.321215 | 0.0984 | 0.4654 |
| rs116237296 | A | G | 0.5284 | 0.1115 | 2.153E-06 | 22.4528284 | 0.2593 | 0.5743 |
| rs11651720 | T | C | -0.1189 | 0.0219 | 5.688E-08 | 29.4693566 | 0.0496 | 0.1861 |
| rs11716293 | C | G | 0.0986 | 0.0189 | 1.689E-07 | 27.2098029 | 0.0416 | 0.1971 |
| rs117657747 | A | G | 0.2089 | 0.0453 | 4.013E-06 | 21.2589533 | 0.0781 | 0.0351399 |
| rs145526037 | T | G | -0.1863 | 0.0406 | 4.473E-06 | 21.0508102 | 0.0927 | 0.1078 |
| rs17138331 | A | G | -0.1434 | 0.0295 | 1.125E-06 | 23.623783 | 0.0645 | 0.4505 |
| rs17661219 | C | G | 0.0872 | 0.0172 | 3.888E-07 | 25.6963388 | 0.0378 | 0.6376 |
| rs2314809 | T | C | -0.0735 | 0.0157 | 2.904E-06 | 21.9114212 | 0.0357 | 0.9547 |
| rs2742396 | T | C | 0.1071 | 0.0168 | 1.832E-10 | 40.6308167 | 0.0364 | 0.2827 |
| rs281728 | A | C | -0.079 | 0.0171 | 3.891E-06 | 21.3381683 | 0.0388 | 0.1291 |
| rs28393318 | A | G | -0.1076 | 0.0235 | 4.616E-06 | 20.9596484 | 0.0538 | 0.0220999 |
| rs57893487 | C | G | -0.1075 | 0.0214 | 4.916E-07 | 25.2281007 | 0.9436 | 0.2848 |
| rs6802288 | A | G | -0.1623 | 0.0173 | 6.574E-21 | 87.9913554 | 0.0401 | 0.720701 |
| rs6806860 | A | C | -0.1008 | 0.0188 | 8.575E-08 | 28.7409116 | 0.0429 | 0.9684 |
| rs6908843 | A | G | 0.0997 | 0.0209 | 1.779E-06 | 22.7506033 | 0.0475 | 0.3168 |
| rs72791296 | T | C | 0.2364 | 0.0466 | 3.968E-07 | 25.7287262 | 0.099 | 0.8857 |
| rs72799710 | T | C | -0.1037 | 0.0217 | 1.792E-06 | 22.8314355 | 0.0494 | 0.5554 |
| rs74979864 | A | T | -0.3184 | 0.0613 | 2.025E-07 | 26.9724467 | 0.1351 | 0.0227798 |
| rs76582507 | A | G | 0.3259 | 0.0676 | 1.421E-06 | 23.2364888 | 0.199 | 0.4423 |
| rs772112 | A | T | -0.142 | 0.0216 | 5.007E-11 | 43.2080195 | 0.0456 | 0.3312 |
| rs9916627 | T | C | -0.096 | 0.0198 | 1.244E-06 | 23.5021319 | 0.0455 | 0.8133 |
| **PDGF-BB** | | | | | | | | |
| rs10512952 | T | C | -0.2816 | 0.0587 | 1.636E-06 | 23.0083196 | 0.1343 | 0.8761 |
| rs116154010 | T | C | 0.3225 | 0.0662 | 1.113E-06 | 23.726805 | 0.1548 | 0.5408 |
| rs11766649 | A | G | 0.0902 | 0.0196 | 3.964E-06 | 21.173699 | 0.044 | 0.5935 |
| rs12289510 | A | G | -0.0772 | 0.0158 | 1.001E-06 | 23.868011 | 0.0357 | 0.6486 |
| rs12615784 | T | C | -0.1003 | 0.0193 | 1.986E-07 | 27.0011991 | 0.0435 | 0.2012 |
| rs147862316 | T | C | 0.2279 | 0.0411 | 2.987E-08 | 30.7397838 | 0.0982 | 0.976 |
| rs2643354 | A | G | 0.1251 | 0.0261 | 1.633E-06 | 22.9683294 | 0.0601 | 0.9536 |
| rs35859699 | A | G | -0.3854 | 0.0838 | 4.223E-06 | 21.146144 | 0.1684 | 0.103 |
| rs62191444 | T | G | -0.112 | 0.0239 | 2.678E-06 | 21.9551317 | 0.0499 | 0.1555 |
| rs6756793 | T | C | 0.0876 | 0.0157 | 2.684E-08 | 31.1246668 | 0.036 | 0.2523 |
| rs6910518 | T | G | 0.0806 | 0.0162 | 6.005E-07 | 24.7477578 | 0.0366 | 0.0126299 |
| rs72972467 | C | G | -0.1616 | 0.0328 | 8.286E-07 | 24.2678235 | 0.0707 | 0.248 |
| rs73162807 | A | C | -0.2313 | 0.0499 | 3.548E-06 | 21.4805787 | 0.1156 | 0.2995 |
| rs9924851 | C | G | 0.0767 | 0.0163 | 2.702E-06 | 22.1366206 | 0.0372 | 0.3981 |
| **RANTES** | | | | | | | | |
| rs10505135 | T | C | 0.035 | 0.0252 | 1.899E-07 | 27.2144071 | 0.0371 | 0.77 |
| rs118096511 | T | C | -0.0676 | 0.0709 | 1.965E-06 | 22.6332093 | 0.1056 | 0.8564 |
| rs11873385 | A | G | 0.0096 | 0.0552 | 2.927E-06 | 21.8666762 | 0.0782 | 0.6483 |
| rs148526102 | T | C | -0.002 | 0.083 | 4.793E-06 | 20.9267695 | 0.1223 | 0.0695392 |
| rs2731672 | T | C | -0.0476 | 0.0272 | 4.827E-06 | 20.8378946 | 0.0408 | 0.7415 |
| rs4795087 | C | G | -0.0065 | 0.0312 | 1.629E-06 | 22.9160869 | 0.0461 | 0.4552 |
| rs62438851 | A | G | -0.024 | 0.0413 | 4.009E-06 | 21.241356 | 0.059 | 0.3531 |
| rs7170339 | C | G | 0.0645 | 0.0904 | 2.187E-06 | 22.434053 | 0.126 | 0.4563 |
| rs72793342 | A | G | -0.0251 | 0.0307 | 9.08E-07 | 24.0184348 | 0.0439 | 0.744201 |
| rs78050316 | A | C | -0.0279 | 0.0859 | 9.946E-07 | 23.9152113 | 0.1354 | 0.8999 |
| **SCF** | | | | | | | | |
| rs10800449 | A | C | 0.0851 | 0.0179 | 1.962E-06 | 22.5968979 | 0.0395 | 0.3429 |
| rs11244035 | T | C | -0.1296 | 0.0279 | 3.501E-06 | 21.5723452 | 0.0637 | 0.2028 |
| rs113127926 | A | C | 0.1974 | 0.0418 | 2.337E-06 | 22.2965412 | 0.0921 | 0.3193 |
| rs117721699 | C | G | -0.2392 | 0.0484 | 7.514E-07 | 24.4189729 | 0.1106 | 0.2319 |
| rs12345108 | T | C | -0.0772 | 0.0167 | 3.731E-06 | 21.3647307 | 0.0375 | 0.5466 |
| rs13412535 | A | G | -0.1065 | 0.0213 | 5.586E-07 | 24.9940005 | 0.0449 | 0.9225 |
| rs138538809 | T | C | -0.5788 | 0.1139 | 3.757E-07 | 25.8169755 | 0.2395 | 0.757399 |
| rs72678285 | A | T | 0.1062 | 0.0231 | 0.00000443 | 21.1310384 | 0.0486 | 0.9211 |
| rs78666213 | T | G | -0.2845 | 0.0574 | 7.152E-07 | 24.5604614 | 0.1237 | 0.5684 |
| rs8045376 | A | G | -0.3126 | 0.068 | 4.268E-06 | 21.1278783 | 0.1551 | 0.144 |
| **SCGF-B** | | | | | | | | |
| rs11111869 | A | G | 0.1621 | 0.0311 | 1.861E-07 | 27.1526211 | 0.0478 | 0.5398 |
| rs112346514 | T | C | -0.3261 | 0.0703 | 3.543E-06 | 21.5058918 | 0.0935 | 0.279 |
| rs1149926 | T | C | -0.3458 | 0.0749 | 3.917E-06 | 21.3035927 | 0.1147 | 0.5433 |
| rs118003677 | T | C | -0.3654 | 0.0786 | 3.348E-06 | 21.6002363 | 0.121 | 0.4539 |
| rs12118918 | A | G | -0.1631 | 0.035 | 3.208E-06 | 21.7039281 | 0.0506 | 0.1128 |
| rs12480722 | T | C | 0.1654 | 0.0353 | 2.812E-06 | 21.9426011 | 0.0532 | 0.3824 |
| rs13287050 | A | T | -0.121 | 0.0263 | 4.118E-06 | 21.1556197 | 0.0396 | 0.1744 |
| rs13866 | T | C | -0.1647 | 0.028 | 3.773E-09 | 34.5810077 | 0.0406 | 0.8009 |
| rs139413256 | A | G | -0.5174 | 0.1076 | 1.532E-06 | 23.109727 | 0.1407 | 0.2583 |
| rs143829871 | T | C | -0.1866 | 0.0399 | 2.852E-06 | 21.8596898 | 0.0613 | 0.0062581 |
| rs144724875 | T | C | 0.5381 | 0.0829 | 8.645E-11 | 42.1098245 | 0.1068 | 0.6169 |
| rs149009264 | A | G | 0.4551 | 0.0985 | 3.793E-06 | 21.3357404 | 0.1459 | 0.4176 |
| rs150733161 | T | C | -0.5255 | 0.112 | 2.687E-06 | 22.0026963 | 0.154 | 0.702 |
| rs151194174 | A | G | 0.4536 | 0.0941 | 1.454E-06 | 23.2238038 | 0.092 | 0.2795 |
| rs264157 | A | G | 0.1079 | 0.0233 | 3.685E-06 | 21.4337201 | 0.0355 | 0.8362 |
| rs34911860 | A | G | -0.3674 | 0.0787 | 3.002E-06 | 21.7818793 | 0.1383 | 0.3843 |
| rs3817303 | T | G | 0.1362 | 0.0294 | 3.602E-06 | 21.4499391 | 0.045 | 0.2007 |
| rs4737731 | T | C | 0.1146 | 0.0251 | 4.871E-06 | 20.8347503 | 0.0392 | 0.4291 |
| rs4976691 | C | G | -0.1484 | 0.0253 | 4.438E-09 | 34.3869192 | 0.0381 | 0.48 |
| rs77954165 | T | C | 0.2631 | 0.0562 | 2.867E-06 | 21.9046124 | 0.0825 | 0.1807 |
| rs78217154 | T | C | 0.3942 | 0.0861 | 4.722E-06 | 20.9504543 | 0.1285 | 0.4439 |
| **SDF-1A** | | | | | | | | |
| rs10474392 | A | G | 0.0934 | 0.0177 | 1.376E-07 | 27.8378865 | 0.0409 | 0.8415 |
| rs10516368 | A | C | -0.4268 | 0.0883 | 1.356E-06 | 23.3569674 | 0.1957 | 0.1721 |
| rs12141941 | T | C | -0.0881 | 0.0186 | 2.263E-06 | 22.4292612 | 0.0397 | 0.623199 |
| rs149893336 | A | G | -0.494 | 0.1082 | 0.00000493 | 20.8395539 | 0.169 | 0.8134 |
| rs1600396 | A | G | -0.0933 | 0.0204 | 4.939E-06 | 20.9118278 | 0.045 | 0.583201 |
| rs62194946 | T | G | -0.0849 | 0.0185 | 4.552E-06 | 21.0552772 | 0.0406 | 0.4697 |
| rs6586903 | T | C | -0.1264 | 0.0268 | 2.421E-06 | 22.2389153 | 0.0571 | 0.0691895 |
| rs76766406 | A | G | 0.4642 | 0.1012 | 4.489E-06 | 21.0347952 | 0.2152 | 0.1541 |
| rs78883416 | C | G | -0.0871 | 0.0182 | 1.755E-06 | 22.8972104 | 0.0382 | 0.8055 |
| **TNF-A** | | | | | | | | |
| rs10767536 | A | G | 0.118 | 0.0253 | 3.146E-06 | 21.7407357 | 0.0379 | 0.3916 |
| rs115018697 | C | G | -0.9542 | 0.197 | 1.273E-06 | 23.4475589 | 0.2747 | 0.487 |
| rs116736594 | T | C | 0.3407 | 0.0702 | 1.223E-06 | 23.5408048 | 0.0975 | 0.7652 |
| rs79105320 | A | G | 0.5573 | 0.1177 | 2.207E-06 | 22.4066239 | 0.1662 | 0.2501 |
| **TNF-B** | | | | | | | | |
| rs10925040 | T | C | 0.1738 | 0.0372 | 2.929E-06 | 21.8001085 | 0.037 | 0.3146 |
| rs2420873 | T | G | 0.1673 | 0.0365 | 4.513E-06 | 20.9822059 | 0.0365 | 0.1159 |
| rs62284710 | A | G | 0.3702 | 0.0782 | 2.183E-06 | 22.3822794 | 0.0754 | 0.9416 |
| rs75240021 | C | G | 0.3713 | 0.0772 | 1.489E-06 | 23.1025697 | 0.0679 | 0.3484 |
| rs76225863 | A | G | 0.7534 | 0.1217 | 5.982E-10 | 38.2749883 | 0.1143 | 0.4256 |
| **TRAIL** | | | | | | | | |
| rs113057689 | A | G | -0.2625 | 0.0489 | 7.972E-08 | 28.8094751 | 0.0861 | 0.2443 |
| rs11875481 | T | C | -0.0969 | 0.0211 | 4.601E-06 | 21.0851692 | 0.0479 | 0.9753 |
| rs12458564 | A | T | -0.1002 | 0.0175 | 1.1E-08 | 32.7758372 | 0.0395 | 0.4956 |
| rs13115587 | A | C | 0.101 | 0.0217 | 3.244E-06 | 21.657969 | 0.0507 | 0.423 |
| rs13278062 | T | G | 0.08 | 0.0157 | 3.326E-07 | 25.9582324 | 0.0358 | 0.0440301 |
| rs139958028 | A | G | 0.1803 | 0.0395 | 4.992E-06 | 20.830117 | 0.0847 | 0.1916 |
| rs183815186 | A | T | -0.3499 | 0.0602 | 6.341E-09 | 33.7745328 | 0.1332 | 0.9828 |
| rs550057 | T | C | -0.0783 | 0.0169 | 3.707E-06 | 21.4607336 | 0.0386 | 0.0708696 |
| rs558572 | T | C | 0.1351 | 0.0265 | 3.419E-07 | 25.9844422 | 0.0606 | 0.4085 |
| rs57396456 | T | C | -0.5641 | 0.0516 | 7.71E-28 | 119.48331 | 0.1198 | 0.2453 |
| rs616114 | T | C | -0.1033 | 0.0162 | 1.715E-10 | 40.6504223 | 0.0367 | 0.7959 |
| rs62093482 | T | C | 0.9827 | 0.0529 | 6.123E-77 | 345.004703 | 0.1249 | 0.8573 |
| rs747324 | T | C | -0.0826 | 0.0178 | 3.338E-06 | 21.528538 | 0.0397 | 0.5134 |
| rs75928541 | A | G | 0.2784 | 0.0591 | 2.442E-06 | 22.1849241 | 0.1206 | 0.5713 |
| rs7599203 | T | C | 0.0918 | 0.02 | 4.333E-06 | 21.0629802 | 0.0453 | 0.1412 |
| rs78682108 | A | G | -0.2383 | 0.0394 | 1.435E-09 | 36.572116 | 0.0819 | 0.628801 |
| **VEGF** | | | | | | | | |
| rs10411345 | C | G | -0.1041 | 0.0218 | 1.733E-06 | 22.7964477 | 0.0424 | 0.8918 |
| rs10757514 | C | G | -0.1024 | 0.0222 | 4.169E-06 | 21.2702537 | 0.0468 | 0.4942 |
| rs10822118 | T | C | -0.0797 | 0.0168 | 2.211E-06 | 22.499703 | 0.0356 | 0.9505 |
| rs10934631 | T | C | -0.1132 | 0.0244 | 3.607E-06 | 21.5175047 | 0.0503 | 0.9395 |
| rs114773511 | T | C | 0.2187 | 0.0441 | 6.971E-07 | 24.5866349 | 0.0865 | 0.0523504 |
| rs12456390 | T | C | -0.0818 | 0.0179 | 4.882E-06 | 20.8775365 | 0.0384 | 0.4639 |
| rs1730969 | C | G | -0.7811 | 0.1696 | 4.106E-06 | 21.2027728 | 0.4056 | 0.6526 |
| rs181031888 | A | T | 0.3767 | 0.0498 | 4.014E-14 | 57.2020062 | 0.0975 | 0.928 |
| rs2039420 | C | G | 0.0975 | 0.0175 | 2.744E-08 | 31.0321481 | 0.037 | 0.731301 |
| rs56071907 | T | C | 0.126 | 0.027 | 0.00000301 | 21.7716963 | 0.0511 | 0.2186 |
| rs60013354 | A | G | -0.2497 | 0.0521 | 1.662E-06 | 22.9636196 | 0.1073 | 0.0223301 |
| rs62401205 | A | C | -0.2015 | 0.0412 | 9.946E-07 | 23.9130171 | 0.0717 | 0.7179 |
| rs6496613 | A | C | -0.2359 | 0.0515 | 4.701E-06 | 20.9758771 | 0.1093 | 0.703399 |
| rs7356919 | A | G | -0.1694 | 0.02 | 2.846E-17 | 71.7208662 | 0.0416 | 0.8399 |
| rs73872715 | T | C | -0.6079 | 0.1299 | 2.864E-06 | 21.8939787 | 0.2424 | 0.2465 |
| rs76458389 | T | G | -0.1786 | 0.0363 | 8.573E-07 | 24.2007243 | 0.0757 | 0.8353 |
| rs77961527 | A | G | 0.2289 | 0.0457 | 5.525E-07 | 25.0805982 | 0.0836 | 0.1692 |
| rs9381249 | T | C | -0.2414 | 0.0396 | 1.038E-09 | 37.1503462 | 0.0874 | 0.6214 |

**Table S14. MR estimates of 41 inflammatory cytokines on cataract.**

|  | **IVW** | | | | **MR-Egger** | | | | **Weighted Median** | | | | **Simple Mode** | | | | **Weighted Mode** | | | |
| --- | --- | --- | --- | --- | --- | --- | --- | --- | --- | --- | --- | --- | --- | --- | --- | --- | --- | --- | --- | --- |
| **Exposures** | **No.of SNPs** | **OR** | **95% CI** | **pval** | **No.of SNPs** | **OR** | **95% CI** | **pval** | **No.of SNPs** | **OR** | **95% CI** | **pval** | **No.of SNPs** | **OR** | **95% CI** | **pval** | **No.of SNPs** | **OR** | **95% CI** | **pval** |
| CTACK | 12 | 0.991 | （0.935-1.049） | 0.747 | 12 | 0.952 | （0.849-1.067） | 0.417 | 12 | 1.001 | （0.926-1.083） | 0.973 | 12 | 1.034 | （0.906-1.181） | 0.628 | 12 | 1.034 | （0.911-1.175） | 0.613 |
| EOTAXIN | 17 | 1.089 | （1.018-1.165） | 0.013 | 17 | 1.137 | （0.961-1.345） | 0.154 | 17 | 1.087 | （0.996-1.187） | 0.064 | 17 | 1.067 | （0.939-1.233） | 0.305 | 17 | 1.078 | （0.942-1.234） | 0.305 |
| GROa | 13 | 1.053 | （1.000-1.109） | 0.049 | 13 | 1.053 | （0.962-1.154） | 0.287 | 13 | 1.049 | （0.976-1.128） | 0.194 | 13 | 1.051 | （0.930-1.188） | 0.440 | 13 | 1.049 | （0.976-1.128） | 0.194 |
| IP-10 | 12 | 0.946 | （0.883-1.015） | 0.121 | 12 | 0.918 | （0.781-1.081） | 0.329 | 12 | 1.000 | （0.922-1.084） | 0.999 | 12 | 1.013 | （0.896-1.146） | 0.838 | 12 | 1.016 | （0.908-1.137） | 0.783 |
| MCP-1 | 16 | 1.044 | (0.973-1.120) | 0.231 | 16 | 1.126 | (0.927-1.368) | 0.251 | 16 | 1.030 | (0.941-1.127) | 0.522 | 16 | 1.037 | (0.871-1.233) | 0.689 | 16 | 1.029 | (0.874-1.210) | 0.737 |
| MCP-3 | 6 | 0.983 | （0.940-1.029） | 0.458 | 6 | 0.988 | （0.876-1.114） | 0.854 | 6 | 0.980 | （0.927-1.035） | 0.466 | 6 | 0.975 | （0.898-1.058） | 0.571 | 6 | 0.980 | （0.927-1.035） | 0.466 |
| MIG | 13 | 1.019 | (0.967-1.073) | 0.489 | 13 | 1.010 | (0.906-1.126) | 0.857 | 13 | 1.052 | (0.980-1.129) | 1.162 | 13 | 1.061 | (0.942-1.195) | 0.345 | 13 | 1.058 | (0.939-1.192) | 0.371 |
| MIP-1A | 4 | 0.979 | (0.884-1.083) | 0.680 | 4 | 0.881 | (0.643-1.208) | 0.515 | 4 | 0.984 | (0.877-1.104) | 0.788 | 4 | 0.999 | (0.855-1.168) | 0.992 | 4 | 1.000 | (0.848-1.178) | 0.997 |
| MIP-1B | 22 | 1.031 | (0.970-1.094) | 0.327 | 22 | 0.993 | (0.836-1.180) | 0.938 | 22 | 1.014 | (0.936-1.099) | 0.733 | 22 | 0.992 | (0.861-1.142) | 0.912 | 22 | 1.002 | (0.892-1.126) | 0.969 |
| RANTES | 10 | 0.998 | (0.938-1.062) | 0.945 | 10 | 0.991 | (0.849-1.156) | 0.909 | 10 | 0.969 | (0.897-1.046) | 0.418 | 10 | 0.964 | (0.848-1.096) | 0.590 | 10 | 0.963 | (0.854-1.087) | 0.558 |
| SDF-1A | 9 | 1.003 | (0.914-1.100) | 0.954 | 9 | 0.902 | (0.750-1.085) | 0.309 | 9 | 0.943 | (0.832-1.068) | 0.351 | 9 | 0.911 | (0.739-1.123) | 0.409 | 9 | 0.917 | (0.764-0.101) | 0.380 |
| SCGF-B | 21 | 1.032 | (0.991-1.074) | 0.129 | 21 | 1.014 | (0.933-1.103) | 0.744 | 21 | 1.008 | (0.954-1.066) | 0.768 | 21 | 0.988 | (0.889-1.099) | 0.830 | 21 | 0.991 | (0.897-1.096) | 0.864 |
| B-NGF | 4 | 0.995 | （0.879-1.126） | 0.940 | 4 | 0.902 | （0.417-1.952） | 0.818 | 4 | 1.019 | （0.894-1.161） | 0.784 | 4 | 1.083 | （0.888-1.321） | 0.487 | 4 | 1.080 | （0.860-1.357） | 0.553 |
| FGF2 | 7 | 1.183 | （1.004-1.393） | 0.045 | 7 | 1.602 | （1.031-2.488） | 0.090 | 7 | 1.166 | （1.031-2.488） | 0.090 | 7 | 1.179 | （0.900-1.544） | 0.278 | 7 | 1.166 | （0.992-1.370） | 0.062 |
| G-CSF | 9 | 1.015 | （0.887-1.161） | 0.829 | 9 | 1.022 | （0.799-1.308） | 0.868 | 9 | 0.968 | （0.852-1.099） | 0.612 | 9 | 0.948 | （0.800-1.125） | 0.559 | 9 | 0.968 | （0.852-1.099） | 0.612 |
| HGF | 9 | 1.001 | (0.910-1.101) | 0.986 | 9 | 1.042 | (0.844-1.287) | 0.712 | 9 | 1.055 | (0.928-1.199) | 0.415 | 9 | 1.110 | (0.888-1.387) | 0.386 | 9 | 1.097 | (0.917-1.314) | 0.342 |
| M-CSF | 12 | 1.025 | (0.980-1.071) | 0.284 | 12 | 1.015 | (0.925-1.113) | 0.764 | 12 | 1.018 | (0.961-1.077) | 0.545 | 12 | 1.037 | (0.942-1.140) | 0.475 | 12 | 1.023 | (0.937-1.117) | 0.626 |
| PDGF-BB | 14 | 0.949 | (0.878-1.025) | 0.184 | 14 | 0.874 | (0.727-1.050) | 0.177 | 14 | 0.926 | (0.834-1.028) | 0.147 | 14 | 0.922 | (0.768-1.107) | 0.398 | 14 | 0.925 | (0.783-1.093) | 0.377 |
| SCF | 10 | 1.070 | (0.976-1.174) | 0.150 | 10 | 1.231 | (1.015-1.493) | 0.068 | 10 | 1.070 | (0.945-1.212) | 0.287 | 10 | 1.136 | (0.918-1.406) | 0.270 | 10 | 1.138 | (0.933-1.389) | 0.234 |
| VEGF | 18 | 1.026 | (0.958-1.098) | 0.459 | 18 | 1.065 | (0.916-1.238) | 0.426 | 18 | 1.023 | (0.940-1.112) | 0.601 | 18 | 1.021 | (0.895-1.165) | 0.761 | 18 | 1.017 | (0.898-1.152) | 0.795 |
| IL-10 | 15 | 1.019 | （0.957-1.085） | 0.560 | 15 | 1.235 | （1.006-1.516） | 0.065 | 15 | 1.032 | （0.944-1.127） | 0.489 | 15 | 0.959 | （0.801-1.147） | 0.651 | 15 | 1.032 | （0.944-1.127） | 0.489 |
| IL-12-p70 | 15 | 1.011 | （0.930-1.087） | 0.774 | 15 | 1.115 | （0.858-1.448） | 0.429 | 15 | 0.965 | （0.873-1.067） | 0.487 | 15 | 0.935 | （0.782-1.119） | 0.476 | 15 | 0.932 | （0.794-1.094） | 0.405 |
| IL-13 | 14 | 1.027 | （0.979-1.077） | 0.284 | 14 | 1.011 | （0.924-1.105） | 0.822 | 14 | 1.012 | （0.946-1.082） | 0.723 | 14 | 0.983 | （0.872-1.108） | 0.783 | 14 | 0.976 | （0.873-1.092） | 0.680 |
| IL-16 | 10 | 1.022 | （0.970-1.077） | 0.415 | 10 | 1.011 | （0.929-1.101） | 0.800 | 10 | 1.020 | （0.954-1.091） | 0.553 | 10 | 1.040 | （0.942-1.148） | 0.460 | 10 | 1.022 | （0.930-1.124） | 0.663 |
| IL-17 | 8 | 1.015 | （0.928-1.110） | 0.744 | 8 | 1.040 | （0.870-1.243） | 0.683 | 8 | 1.013 | （0.903-1.137） | 0.827 | 8 | 1.150 | （0.956-1.382） | 0.182 | 8 | 1.017 | （0.888-1.165） | 0.816 |
| IL-18 | 13 | 0.998 | （0.947-1.052） | 0.955 | 13 | 1.010 | （0.913-1.117） | 0.854 | 13 | 1.004 | （0.934-1.079） | 0.916 | 13 | 0.957 | （0.857-1.068） | 0.446 | 13 | 1.016 | （0.906-1.140） | 0.793 |
| IL-1B | 3 | 0.975 | （0.862-1.105） | 0.699 | 3 | 0.873 | （0.680-1.120） | 0.478 | 3 | 0.994 | （0.857-1.154） | 0.938 | 3 | 0.995 | （0.819-1.208） | 0.963 | 3 | 1.006 | （0.853-1.186） | 0.954 |
| IL-1RA | 10 | 0.932 | （0.874-0.993） | 0.030 | 10 | 0.819 | （0.685-0.979） | 0.060 | 10 | 0.966 | （0.888-1.051） | 0.424 | 10 | 0.990 | （0.863-1.134） | 0.884 | 10 | 0.989 | （0.864-1.131） | 0.871 |
| IL-2 | 8 | 1.000 | （0.939-1.065） | 0.994 | 8 | 1.016 | （0.891-1.158） | 0.824 | 8 | 0.979 | （0.900-1.066） | 0.627 | 8 | 0.971 | （0.849-0.110） | 0.675 | 8 | 0.985 | （0.876-1.108） | 0.806 |
| IL-2RA | 9 | 0.970 | （0.909-1.035） | 0.351 | 9 | 0.967 | （0.851-1.099） | 0.622 | 9 | 0.971 | （0.901-1.046） | 0.432 | 9 | 0.977 | （0.888-1.075） | 0.645 | 9 | 0.969 | （0.883-1.063） | 0.524 |
| IL-4 | 14 | 1.001 | （0.926-1.082） | 0.984 | 14 | 1.016 | （0.862-1.198） | 0.851 | 14 | 1.044 | （0.935-1.166） | 0.446 | 14 | 1.063 | （0.869-1.302） | 0.562 | 14 | 1.073 | （0.885-1.300） | 0.487 |
| IL-5 | 8 | 0.987 | （0.924-1.054） | 0.701 | 8 | 0.923 | （0.799-1.066） | 0.923 | 8 | 1.018 | （0.934-1.110） | 0.684 | 8 | 1.039 | （0.907-1.191） | 0.596 | 8 | 1.032 | （0.902-1.181） | 0.660 |
| IL-6 | 11 | 1.073 | （0.959-1.201） | 0.220 | 11 | 0.966 | （0.748-1.247） | 0.796 | 11 | 1.077 | （0.951-1.221） | 0.244 | 11 | 1.088 | （0.904-1.309） | 0.392 | 11 | 1.095 | （0.931-1.288） | 0.296 |
| IL-7 | 12 | 1.025 | （0.976-1.076） | 0.321 | 12 | 0.999 | （0.890-1.122） | 0.989 | 12 | 1.040 | （0.972-1.113） | 0.252 | 12 | 1.067 | （0.961-1.184） | 0.250 | 12 | 1.049 | （0.964-1.141） | 0.293 |
| IL-8 | 8 | 1.021 | （0.935-1.115） | 0.641 | 8 | 1.101 | （0.938-1.292） | 0.284 | 8 | 0.999 | （0.904-1.105） | 0.988 | 8 | 0.973 | （0.834-1.134） | 0.734 | 8 | 0.980 | （0.844-1.137） | 0.799 |
| IL-9 | 6 | 0.994 | （0.911-1.084） | 0.891 | 6 | 0.972 | （0.777-1.217） | 0.819 | 6 | 0.990 | （0.888-1.104） | 0.857 | 6 | 0.979 | （0.843-1.137） | 0.795 | 6 | 0.980 | （0.852-1.127） | 0.793 |
| IFN-G | 12 | 0.996 | （0.921-1.077） | 0.925 | 12 | 0.997 | （0.850-1.169） | 0.967 | 12 | 0.992 | （0.891-1.105） | 0.883 | 12 | 1.012 | （0.861-1.189） | 0.886 | 12 | 1.007 | （0.866-1.170） | 0.929 |
| MIF | 10 | 0.933 | （0.864-1.009） | 0.082 | 10 | 0.847 | （0.735-0.976） | 0.050 | 10 | 0.929 | （0.847-1.018） | 0.116 | 10 | 0.918 | （0.775-1.088） | 0.351 | 10 | 0.921 | （0.791-1.074） | 0.324 |
| TNF-A | 4 | 0.945 | （0.860-1.039） | 0.246 | 4 | 0.997 | （0.860-1.157） | 0.974 | 4 | 0.955 | （0.853-1.070） | 0.428 | 4 | 0.963 | （0.829-1.119） | 0.657 | 4 | 0.967 | （0.831-1.125） | 0.692 |
| TNF-B | 5 | 0.994 | （0.937-1.054） | 0.835 | 5 | 1.051 | （0.935-1.181） | 0.466 | 5 | 1.020 | （0.953-1.091） | 0.564 | 5 | 1.024 | （0.945-1.109） | 0.596 | 5 | 1.022 | （0.948-1.101） | 0.600 |
| TRAIL | 16 | 1.040 | （0.989-1.092） | 0.124 | 16 | 1.121 | （1.043-1.206） | 0.008 | 16 | 1.062 | （0.990-1.139） | 0.094 | 16 | 0.998 | （0.868-1.148） | 0.982 | 16 | 1.076 | （1.005-1.152） | 0.054 |

CI, Confidence interval; OR, Odds Ratio; pval, P-value; SNPs, single nucleotide polymorphisms. OR and 95% CI represent the change in the odds ratio of cataract per 1 SD increase in inflammatory cytokines.

**Table S15. Heterogeneity and horizontal pleiotropy tests of 41 inflammatory cytokines on cataract.**

| **Exposures** | **Q_1_ pval (IVW)** | **Q_2_ pval (MR-Egger)** | **I^2^** | **intercept** | **intercept pval** |
| --- | --- | --- | --- | --- | --- |
| B-NGF | 0.152 | 0.077 | 43.3% | 0.014 | 0.822 |
| CTACK | 0.490 | 0.456 | 0.0% | 0.010 | 0.447 |
| Eotaxin | 0.292 | 0.456 | 13.8% | 0.010 | 0.588 |
| FGF2 | 0.034 | 0.086 | 55.9% | 0.086 | 0.210 |
| G-CSF | 0.023 | 0.013 | 55.1% | -0.001 | 0.947 |
| GROa | 0.676 | 0.593 | 0.0% | 0.000 | 0.999 |
| HGF | 0.571 | 0.482 | 0.0% | -0.006 | 0.685 |
| IFN-G | 0.708 | 0.623 | 0.0% | 0.000 | 0.997 |
| IL-1B | 0.403 | 0.373 | 0.0% | 0.023 | 0.496 |
| IL-1RA | 0.539 | 0.688 | 0.0% | 0.021 | 0.167 |
| IL-2 | 0.460 | 0.357 | 0.0% | -0.003 | 0.800 |
| IL-2RA | 0.243 | 0.172 | 22.5% | 0.001 | 0.961 |
| IL-4 | 0.485 | 0.407 | 0.0% | -0.002 | 0.839 |
| IL-5 | 0.547 | 0.557 | 0.0% | 0.014 | 0.346 |
| IL-6 | 0.091 | 0.092 | 38.7% | 0.013 | 0.391 |
| IL-7 | 0.463 | 0.395 | 0.0% | 0.006 | 0.645 |
| IL-8 | 0.117 | 0.141 | 39.4% | -0.016 | 0.316 |
| IL-9 | 0.470 | 0.340 | 0.0% | 0.005 | 0.844 |
| IL-10 | 0.480 | 0.704 | 0.0% | -0.022 | 0.076 |
| IL-12p70 | 0.600 | 0.569 | 0.0% | -0.011 | 0.457 |
| IL-13 | 0.795 | 0.742 | 0.0% | 0.004 | 0.691 |
| IL-16 | 0.866 | 0.806 | 0.0% | 0.003 | 0.771 |
| IL-17 | 0.367 | 0.277 | 8.1% | -0.005 | 0.765 |
| IL-18 | 0.930 | 0.895 | 0.0% | -0.002 | 0.803 |
| IP-10 | 0.124 | 0.093 | 33.3% | 0.006 | 0.696 |
| M-CSF | 0.934 | 0.898 | 0.0% | 0.003 | 0.821 |
| MCP-1 | 0.668 | 0.648 | 0.0% | -0.010 | 0.427 |
| MCP-3 | 0.711 | 0.571 | 0.0% | -0.001 | 0.933 |
| MIF | 0.146 | 0.248 | 32.7% | 0.021 | 0.157 |
| MIG | 0.511 | 0.428 | 0.0% | 0.002 | 0.870 |
| MIP-1A | 0.897 | 0.941 | 0.0% | 0.018 | 0.562 |
| MIP-1B | 0.293 | 0.253 | 12.5% | 0.005 | 0.657 |
| PDGF-BB | 0.445 | 0.438 | 0.2% | 0.010 | 0.354 |
| RANTES | 0.879 | 0.815 | 0.0% | 0.001 | 0.923 |
| SCF | 0.503 | 0.683 | 0.0% | -0.021 | 0.143 |
| SCGF-B | 0.931 | 0.912 | 0.0% | 0.004 | 0.654 |
| SDF-1A | 0.654 | 0.751 | 0.0% | 0.014 | 0.234 |
| TNF-A | 0.706 | 0.754 | 0.0% | -0.016 | 0.458 |
| TNF-B | 0.308 | 0.326 | 16.7% | -0.017 | 0.360 |
| TRAIL | 0.630 | 0.989 | 0.0% | -0.018 | 0.014 |
| VEGF | 0.117 | 0.097 | 29.5% | -0.006 | 0.594 |

Q1 pval: P-value of Q test from IVW method; Q2 pval: P-value of Q test from MR-Egger method.

Abbreviations: pval, P-value; Q, Cochran Q statistics; SNPs, single nucleotide polymorphisms; IVW, the inverse variance weighted method.

**Table S16. SNPs information of 41 inflammation cytokines with cataract.**

|  | **Inflammatory cytokines (exposure)** | | | | | **cataract(outcome)** | | |
| --- | --- | --- | --- | --- | --- | --- | --- | --- |
| **SNP** | **effect allele** | **other allele** | **Beta** | **se** | **pval** | **F** | **se** | **pval** |
| **B-NGF** | | | | | | | | |
| rs28637706 | T | G | -0.1554 | 0.0261 | 2.717E-09 | 35.4305296 | 0.0126 | 0.0751294 |
| rs4767014 | T | C | -0.1211 | 0.0264 | 4.518E-06 | 21.029893 | 0.0124 | 0.222 |
| rs71641308 | T | C | 0.1969 | 0.0429 | 4.424E-06 | 21.0539447 | 0.0199 | 0.4182 |
| rs73472576 | T | C | -0.1146 | 0.0251 | 4.813E-06 | 20.8342764 | 0.0117 | 0.9954 |
| **CTACK** | | | | | | | | |
| rs10854859 | A | G | -0.1498 | 0.0293 | 3.049E-07 | 26.1247203 | 0.014 | 0.2218 |
| rs116303454 | A | G | 0.3754 | 0.081 | 3.579E-06 | 21.4675143 | 0.0366 | 0.7365 |
| rs116871507 | A | T | -0.2086 | 0.0448 | 0.00000316 | 21.668849 | 0.0227 | 0.757501 |
| rs116943377 | A | G | 0.2878 | 0.0611 | 2.496E-06 | 22.1749384 | 0.0304 | 0.846 |
| rs117932939 | T | C | 0.1969 | 0.0422 | 3.089E-06 | 21.7585421 | 0.021 | 0.208 |
| rs118084576 | A | G | 0.5675 | 0.1226 | 0.00000366 | 21.4148015 | 0.0652 | 0.6603 |
| rs184329319 | T | G | -0.3069 | 0.0648 | 2.173E-06 | 22.4185246 | 0.0328 | 0.3079 |
| rs55764737 | T | C | 0.5424 | 0.0967 | 2.012E-08 | 31.4448608 | 0.0476 | 0.504001 |
| rs57338032 | A | G | 0.1443 | 0.0316 | 4.831E-06 | 20.8411539 | 0.0155 | 0.703701 |
| rs57789542 | T | C | -0.7687 | 0.1659 | 3.575E-06 | 21.4577535 | 0.0702 | 0.787 |
| rs60247384 | T | C | 0.1128 | 0.0245 | 4.302E-06 | 21.1860159 | 0.0122 | 0.0493901 |
| rs76395525 | A | G | 0.5193 | 0.1081 | 1.553E-06 | 23.0647377 | 0.0573 | 0.217 |
| **Eotaxin** | | | | | | | | |
| rs11087905 | A | C | 0.0954 | 0.0188 | 4.07E-07 | 25.7439435 | 0.0123 | 0.7217 |
| rs112347425 | T | C | 0.1595 | 0.0276 | 7.771E-09 | 33.388525 | 0.0203 | 0.955 |
| rs11920996 | T | C | 0.2979 | 0.0377 | 2.919E-15 | 62.4241056 | 0.0275 | 0.9564 |
| rs1677588 | T | G | 0.1181 | 0.025 | 2.223E-06 | 22.310731 | 0.0182 | 0.3115 |
| rs2024050 | A | G | 0.164 | 0.0302 | 5.467E-08 | 29.4827394 | 0.0224 | 0.4281 |
| rs2027855 | T | C | 0.0743 | 0.0162 | 4.272E-06 | 21.0301137 | 0.0119 | 0.835 |
| rs2040143 | A | G | -0.0858 | 0.0178 | 1.333E-06 | 23.2288973 | 0.0129 | 0.1382 |
| rs2229593 | T | C | 0.3647 | 0.0406 | 2.838E-19 | 80.6702648 | 0.0297 | 0.0835007 |
| rs2249581 | T | C | -0.0899 | 0.018 | 5.912E-07 | 24.9383891 | 0.0132 | 0.579001 |
| rs5754733 | A | C | -0.105 | 0.0213 | 8.196E-07 | 24.2948048 | 0.0153 | 0.0132099 |
| rs57723662 | C | G | -0.0982 | 0.0213 | 3.878E-06 | 21.2499441 | 0.0153 | 0.6277 |
| rs60075014 | T | C | -0.1688 | 0.0356 | 2.078E-06 | 22.4770293 | 0.0235 | 0.4559 |
| rs7231030 | A | C | 0.0903 | 0.0193 | 2.709E-06 | 21.885421 | 0.0141 | 0.3042 |
| rs73072941 | A | T | -0.1281 | 0.0263 | 1.113E-06 | 23.7181486 | 0.0182 | 0.9419 |
| rs745331 | A | G | -0.0821 | 0.0176 | 3.036E-06 | 21.7547953 | 0.0125 | 0.0606694 |
| rs75426604 | A | C | -0.1371 | 0.0291 | 2.397E-06 | 22.1913107 | 0.02 | 0.2613 |
| rs9317045 | A | C | 0.1172 | 0.0236 | 6.954E-07 | 24.6561487 | 0.0164 | 0.0180401 |
| **FGF2** | | | | | | | | |
| rs13412535 | A | G | -0.1129 | 0.0224 | 4.763E-07 | 25.3967228 | 0.0146 | 0.2223 |
| rs147409637 | T | C | 0.201 | 0.0431 | 3.077E-06 | 21.7431973 | 0.0319 | 0.3638 |
| rs17094040 | T | C | 0.1051 | 0.0229 | 4.308E-06 | 21.0581541 | 0.0166 | 0.1835 |
| rs2849358 | A | G | 0.0911 | 0.0193 | 2.321E-06 | 22.2745002 | 0.0134 | 0.9126 |
| rs4795091 | A | G | 0.1239 | 0.0266 | 3.055E-06 | 21.6902807 | 0.0187 | 0.0132501 |
| rs76253061 | T | C | -0.4811 | 0.1041 | 3.807E-06 | 21.3528125 | 0.0709 | 0.000928603 |
| rs78873483 | A | G | 0.1286 | 0.0282 | 0.00000498 | 20.7907211 | 0.0184 | 0.2109 |
| **G-CSF** | | | | | | | | |
| rs10939033 | A | G | -0.0775 | 0.0163 | 2.074E-06 | 22.6005443 | 0.0118 | 0.9145 |
| rs117261691 | T | C | 0.1318 | 0.0288 | 4.669E-06 | 20.9380652 | 0.0212 | 0.8064 |
| rs183023730 | T | G | 0.7898 | 0.1677 | 2.471E-06 | 22.1734096 | 0.0913 | 0.3051 |
| rs586802 | A | G | 0.0882 | 0.0187 | 2.398E-06 | 22.2405058 | 0.0134 | 0.7815 |
| rs6740648 | T | C | 0.0818 | 0.0172 | 0.0000019 | 22.6120749 | 0.0124 | 0.1072 |
| rs74148555 | T | C | -0.3771 | 0.0753 | 5.591E-07 | 25.0734338 | 0.0411 | 0.781801 |
| rs76287671 | T | C | 0.0894 | 0.0189 | 2.191E-06 | 22.3687714 | 0.0136 | 0.2975 |
| rs77318030 | T | C | -0.2031 | 0.0427 | 0.000002017 | 22.61803121 | 0.029 | 0.1706 |
| rs78523761 | A | G | 0.5374 | 0.1139 | 0.000002389 | 22.25421208 | 0.0725 | 0.000879407 |
| **GROa** | | | | | | | | |
| rs114991247 | T | C | -0.2202 | 0.0463 | 1.971E-06 | 22.6061961 | 0.0197 | 0.499001 |
| rs115214168 | T | C | 0.4528 | 0.0828 | 4.479E-08 | 29.8886987 | 0.04 | 0.3006 |
| rs1361829 | A | G | -0.1106 | 0.0241 | 4.576E-06 | 21.0489779 | 0.0117 | 0.7396 |
| rs140734053 | A | G | 0.7333 | 0.1545 | 2.069E-06 | 22.514445 | 0.0609 | 0.0435402 |
| rs150194856 | T | C | -0.4223 | 0.0914 | 3.857E-06 | 21.3355879 | 0.0452 | 0.832 |
| rs17171245 | T | G | 0.2446 | 0.053 | 3.932E-06 | 21.287076 | 0.0271 | 0.1185 |
| rs185768063 | A | G | 0.4038 | 0.076 | 1.055E-07 | 28.2137159 | 0.0357 | 0.9024 |
| rs188345231 | T | C | 0.6177 | 0.1322 | 2.968E-06 | 21.8196176 | 0.0652 | 0.394 |
| rs3026943 | A | C | -0.1246 | 0.0256 | 1.084E-06 | 23.6761454 | 0.0123 | 0.3193 |
| rs62024303 | A | G | -0.3013 | 0.066 | 4.908E-06 | 20.8288473 | 0.0291 | 0.5782 |
| rs76215157 | C | G | -0.7398 | 0.1564 | 2.226E-06 | 22.3619669 | 0.0588 | 0.6557 |
| rs76390238 | C | G | 0.6223 | 0.1352 | 4.141E-06 | 21.17391 | 0.0623 | 0.1314 |
| rs79454658 | T | C | 0.2784 | 0.0596 | 3.017E-06 | 21.8072442 | 0.0289 | 0.4862 |
| **HGF** | | | | | | | | |
| rs11060254 | A | G | -0.0765 | 0.0166 | 3.974E-06 | 21.2325661 | 0.0124 | 0.2481 |
| rs11129909 | T | C | -0.0738 | 0.0161 | 4.457E-06 | 21.0066482 | 0.012 | 0.2882 |
| rs13412535 | A | G | -0.1043 | 0.0213 | 9.671E-07 | 23.9720514 | 0.0146 | 0.2223 |
| rs2003620 | T | C | 0.2277 | 0.0487 | 2.978E-06 | 21.8556602 | 0.0347 | 0.6069 |
| rs362307 | T | C | 0.1511 | 0.0328 | 0.00000421 | 21.2166607 | 0.023 | 0.6672 |
| rs4245058 | T | C | -0.1552 | 0.0331 | 2.683E-06 | 21.9797747 | 0.0242 | 0.4235 |
| rs57146176 | A | G | -0.0987 | 0.0208 | 2.184E-06 | 22.5114477 | 0.0231 | 0.5009 |
| rs5745687 | T | C | -0.3008 | 0.0404 | 9.922E-14 | 55.4228333 | 0.0288 | 0.2964 |
| rs80051150 | T | C | 0.198 | 0.0413 | 1.684E-06 | 22.9787324 | 0.0317 | 0.749701 |
| **IFN-G** | | | | | | | | |
| rs113399544 | A | G | -0.0849 | 0.0183 | 3.323E-06 | 21.5179571 | 0.0128 | 0.7329 |
| rs113600793 | A | C | 0.1871 | 0.0371 | 4.426E-07 | 25.4265591 | 0.0249 | 0.4469 |
| rs115729819 | A | G | 0.2511 | 0.0514 | 1.045E-06 | 23.8591531 | 0.0363 | 0.1564 |
| rs117046255 | T | C | -0.0968 | 0.0207 | 2.787E-06 | 21.8624013 | 0.0149 | 0.4796 |
| rs11843756 | T | G | 0.1812 | 0.0391 | 3.622E-06 | 21.4709227 | 0.0286 | 0.9026 |
| rs12420286 | T | C | 0.2357 | 0.05 | 2.452E-06 | 22.2160576 | 0.0351 | 0.8289 |
| rs147378920 | A | G | -0.384 | 0.0751 | 3.195E-07 | 26.1378831 | 0.0407 | 0.9276 |
| rs1867282 | T | C | 0.0781 | 0.0166 | 2.478E-06 | 22.1296084 | 0.0118 | 0.3666 |
| rs2073438 | A | G | 0.092 | 0.0188 | 9.551E-07 | 23.9413036 | 0.0133 | 0.2855 |
| rs7088799 | T | G | -0.0805 | 0.0166 | 1.274E-06 | 23.5105843 | 0.0119 | 0.8203 |
| rs73479333 | C | G | -0.1123 | 0.024 | 2.816E-06 | 21.8889468 | 0.0173 | 0.0994306 |
| rs74148555 | T | C | -0.3771 | 0.077 | 9.858E-07 | 23.9783586 | 0.0411 | 0.781801 |
| **IL-1B** | | | | | | | | |
| rs143319329 | T | C | 0.4357 | 0.093 | 2.835E-06 | 21.9356097 | 0.0711 | 0.2051 |
| rs4786740 | A | C | 0.1264 | 0.0265 | 0.00000182 | 22.7375092 | 0.0119 | 0.624701 |
| rs61335305 | A | C | 0.4333 | 0.0928 | 3.015E-06 | 21.7882281 | 0.0437 | 0.722199 |
| **IL-1RA** | | | | | | | | |
| rs1054402 | T | C | 0.1325 | 0.0269 | 8.201E-07 | 24.2488368 | 0.0134 | 0.1549 |
| rs117181659 | A | G | -0.2204 | 0.0478 | 0.00000392 | 21.2486381 | 0.0239 | 0.0925209 |
| rs11869294 | C | G | -0.2286 | 0.047 | 1.128E-06 | 23.6439727 | 0.0216 | 0.372 |
| rs13343438 | A | G | 0.2771 | 0.0607 | 4.973E-06 | 20.8285666 | 0.0303 | 0.00978408 |
| rs35590641 | C | G | -0.1167 | 0.025 | 3.041E-06 | 21.7783718 | 0.0122 | 0.9366 |
| rs3876037 | A | G | 0.1234 | 0.027 | 4.733E-06 | 20.8769237 | 0.0121 | 0.786799 |
| rs56134659 | A | G | -0.1109 | 0.0236 | 2.564E-06 | 22.0700237 | 0.0116 | 0.9361 |
| rs61335305 | A | C | 0.4315 | 0.0904 | 1.812E-06 | 22.7713329 | 0.0437 | 0.722199 |
| rs6699436 | A | G | -0.1858 | 0.0404 | 4.365E-06 | 21.1393876 | 0.0187 | 0.737099 |
| rs9985296 | T | C | 0.1053 | 0.0231 | 4.952E-06 | 20.7680871 | 0.0116 | 0.986 |
| **IL-2** | | | | | | | | |
| rs13412535 | A | G | 0.174 | 0.0331 | 1.447E-07 | 27.6181929 | 0.0146 | 0.2223 |
| rs16836080 | A | G | 0.1158 | 0.0253 | 4.841E-06 | 20.9376929 | 0.0124 | 0.768999 |
| rs2690020 | A | G | 0.1158 | 0.0245 | 2.273E-06 | 22.3273767 | 0.0117 | 0.0616396 |
| rs4479767 | A | G | 0.1821 | 0.0392 | 3.444E-06 | 21.5675348 | 0.02 | 0.8224 |
| rs4634519 | A | G | -0.1249 | 0.0268 | 0.00000318 | 21.7074115 | 0.013 | 0.9958 |
| rs61335305 | A | C | 0.4439 | 0.0913 | 1.157E-06 | 23.6255259 | 0.0437 | 0.722199 |
| rs62124990 | T | G | -0.7013 | 0.149 | 2.502E-06 | 22.1405238 | 0.0424 | 0.4154 |
| rs7615304 | A | G | -0.1139 | 0.024 | 2.161E-06 | 22.5101151 | 0.0116 | 0.3663 |
| **IL-2RA** | | | | | | | | |
| rs11241559 | T | G | -0.124 | 0.0264 | 0.00000275 | 22.0496505 | 0.0133 | 0.886 |
| rs117244812 | A | G | -0.7187 | 0.1493 | 1.474E-06 | 23.1601767 | 0.0682 | 0.7954 |
| rs12789243 | T | C | 0.1263 | 0.0276 | 4.608E-06 | 20.9293013 | 0.0135 | 0.0389601 |
| rs17147986 | A | C | -0.2976 | 0.0337 | 1.069E-18 | 77.9421257 | 0.0165 | 0.382 |
| rs17624670 | A | G | -0.125 | 0.0273 | 4.639E-06 | 20.9537099 | 0.0134 | 0.9291 |
| rs34037190 | A | G | 0.4784 | 0.0935 | 3.134E-07 | 26.1652756 | 0.036 | 0.7984 |
| rs56213152 | T | C | 0.1269 | 0.0271 | 2.937E-06 | 21.9154734 | 0.0135 | 0.0183 |
| rs759244 | A | T | -0.1094 | 0.0238 | 4.257E-06 | 21.117715 | 0.0119 | 0.4514 |
| rs79100208 | C | G | 0.8345 | 0.1758 | 2.052E-06 | 22.5206611 | 0.0723 | 0.7172 |
| **IL-4** | | | | | | | | |
| rs116705532 | T | G | -0.4675 | 0.0978 | 1.727E-06 | 22.844369 | 0.0722 | 0.1658 |
| rs117146485 | T | C | -0.2856 | 0.0625 | 4.945E-06 | 20.8761312 | 0.0434 | 0.1644 |
| rs12238729 | T | C | 0.5271 | 0.1096 | 1.505E-06 | 23.116432 | 0.0923 | 0.2575 |
| rs12640583 | T | G | -0.1104 | 0.0214 | 2.451E-07 | 26.6075108 | 0.0156 | 0.740099 |
| rs17713451 | A | G | 0.1255 | 0.0252 | 6.41E-07 | 24.795908 | 0.0183 | 0.7716 |
| rs1867282 | T | C | 0.0808 | 0.0162 | 5.822E-07 | 24.8706044 | 0.0118 | 0.3666 |
| rs2073438 | A | G | 0.0847 | 0.0183 | 3.725E-06 | 21.4169828 | 0.0133 | 0.2855 |
| rs2346020 | A | G | 0.079 | 0.0169 | 2.843E-06 | 21.8461253 | 0.0124 | 0.4327 |
| rs2708586 | T | C | -0.0767 | 0.0166 | 3.588E-06 | 21.3436258 | 0.0122 | 0.8844 |
| rs56408830 | A | G | -0.1794 | 0.0365 | 9.075E-07 | 24.151983 | 0.0281 | 0.8331 |
| rs7613691 | A | G | 0.1787 | 0.0382 | 2.962E-06 | 21.8784235 | 0.0267 | 0.2235 |
| rs79597994 | T | C | -0.5855 | 0.1271 | 4.056E-06 | 21.2156501 | 0.0686 | 0.4031 |
| rs9506111 | A | G | -0.1446 | 0.0314 | 4.081E-06 | 21.2017123 | 0.0231 | 0.6432 |
| rs9941733 | A | G | 0.1156 | 0.0229 | 4.331E-07 | 25.4764171 | 0.0153 | 0.1394 |
| **IL-5** | | | | | | | | |
| rs10178043 | T | G | 0.2579 | 0.0553 | 3.126E-06 | 21.7368748 | 0.026 | 0.8706 |
| rs148634917 | A | G | -0.517 | 0.1087 | 1.974E-06 | 22.6082361 | 0.0437 | 0.4457 |
| rs28793375 | T | C | 0.1697 | 0.0362 | 2.746E-06 | 21.962978 | 0.0171 | 0.3446 |
| rs72831687 | A | G | -0.5337 | 0.1104 | 1.324E-06 | 23.3561331 | 0.0459 | 0.8209 |
| rs73040118 | T | C | 0.2294 | 0.049 | 2.903E-06 | 21.9048029 | 0.0238 | 0.3313 |
| rs74811276 | A | G | 0.217 | 0.0471 | 4.082E-06 | 21.2139889 | 0.0211 | 0.0845104 |
| rs7739450 | A | G | -0.1295 | 0.0256 | 4.05E-07 | 25.5743314 | 0.0117 | 0.667599 |
| rs9309063 | T | G | -0.1119 | 0.0245 | 4.866E-06 | 20.8483979 | 0.0117 | 0.502 |
| **IL-6** | | | | | | | | |
| rs10910395 | A | T | -0.108 | 0.0235 | 4.348E-06 | 21.1157384 | 0.017 | 0.8408 |
| rs10982193 | A | G | -0.0793 | 0.0174 | 4.816E-06 | 20.7654986 | 0.013 | 0.0694193 |
| rs113098456 | A | G | -0.1553 | 0.0339 | 4.641E-06 | 20.9815709 | 0.0223 | 0.631499 |
| rs113600793 | A | C | 0.1736 | 0.0359 | 1.291E-06 | 23.3778749 | 0.0249 | 0.4469 |
| rs114373846 | T | C | 0.4196 | 0.0905 | 3.568E-06 | 21.4915771 | 0.0667 | 0.5007 |
| rs11732981 | A | C | 0.0722 | 0.0156 | 3.793E-06 | 21.4150792 | 0.0116 | 0.0393296 |
| rs1333040 | T | C | 0.0747 | 0.0157 | 1.993E-06 | 22.632701 | 0.0116 | 0.00616396 |
| rs13412535 | A | G | -0.1186 | 0.0214 | 3.141E-08 | 30.7069242 | 0.0146 | 0.2223 |
| rs4684700 | T | C | -0.0747 | 0.0162 | 3.912E-06 | 21.2571805 | 0.0117 | 0.524499 |
| rs73273528 | T | C | 0.268 | 0.0553 | 0.00000125 | 23.4808499 | 0.038 | 0.6194 |
| rs76856708 | T | C | 0.336 | 0.0697 | 1.427E-06 | 23.2331173 | 0.0512 | 0.5285 |
| **IL-7** | | | | | | | | |
| rs117509142 | T | C | -0.3213 | 0.0684 | 2.599E-06 | 22.0524712 | 0.0326 | 0.3311 |
| rs11757972 | T | C | 0.121 | 0.0257 | 2.529E-06 | 22.1540183 | 0.0117 | 0.0568905 |
| rs1374279 | A | T | 0.1625 | 0.0347 | 2.792E-06 | 21.9177413 | 0.0164 | 0.8902 |
| rs142397827 | A | C | 0.4592 | 0.0994 | 3.822E-06 | 21.329418 | 0.0412 | 0.4939 |
| rs17091524 | T | C | 0.5092 | 0.1015 | 5.244E-07 | 25.1531697 | 0.0449 | 0.5354 |
| rs2006957 | T | C | 0.2557 | 0.0262 | 1.434E-22 | 95.1934111 | 0.0123 | 0.4343 |
| rs218238 | A | T | 0.1319 | 0.0284 | 3.277E-06 | 21.5576257 | 0.0139 | 0.41 |
| rs28793375 | T | C | 0.1644 | 0.036 | 4.866E-06 | 20.8423479 | 0.0171 | 0.3446 |
| rs62006410 | T | C | -0.1492 | 0.0302 | 7.588E-07 | 24.3933685 | 0.0136 | 0.791899 |
| rs7155170 | A | T | -0.1236 | 0.027 | 4.787E-06 | 20.9438939 | 0.0129 | 0.1947 |
| rs77318030 | T | C | -0.2966 | 0.0631 | 2.639E-06 | 22.0816535 | 0.029 | 0.1706 |
| rs77981494 | T | C | -0.5201 | 0.1055 | 8.225E-07 | 24.289401 | 0.0418 | 0.4736 |
| **IL-8** | | | | | | | | |
| rs113487695 | A | C | -0.6129 | 0.1292 | 2.092E-06 | 22.49109 | 0.0626 | 0.5872 |
| rs116726256 | T | C | -0.2247 | 0.0489 | 4.261E-06 | 21.1030251 | 0.0247 | 0.7154 |
| rs12075 | A | G | 0.1148 | 0.0235 | 9.969E-07 | 23.8508763 | 0.0116 | 0.6983 |
| rs12912642 | A | G | 0.1168 | 0.0251 | 3.212E-06 | 21.6418574 | 0.0122 | 0.0610099 |
| rs183628733 | T | C | 0.6547 | 0.1417 | 3.821E-06 | 21.3354236 | 0.0563 | 0.9052 |
| rs2673604 | A | C | -0.118 | 0.0254 | 3.289E-06 | 21.5701353 | 0.0125 | 0.723701 |
| rs3786107 | A | G | 0.2463 | 0.0517 | 1.935E-06 | 22.6831845 | 0.0223 | 0.0342902 |
| rs75840288 | A | C | 0.5125 | 0.1121 | 4.846E-06 | 20.8897288 | 0.0494 | 0.0752298 |
| **IL-9** | | | | | | | | |
| rs117807175 | C | G | -0.5225 | 0.1106 | 2.327E-06 | 22.3062103 | 0.0518 | 0.9609 |
| rs1259728 | A | G | -0.2381 | 0.0507 | 2.599E-06 | 22.0427781 | 0.0248 | 0.727601 |
| rs3736858 | C | G | -0.1351 | 0.0291 | 3.373E-06 | 21.5421069 | 0.0144 | 0.731301 |
| rs41294750 | T | C | 0.3442 | 0.0736 | 2.916E-06 | 21.8589632 | 0.0346 | 0.2532 |
| rs4880409 | T | C | -0.3552 | 0.0716 | 6.952E-07 | 24.5970671 | 0.0578 | 0.0878092 |
| rs73443903 | A | C | 0.2162 | 0.046 | 2.569E-06 | 22.0779717 | 0.023 | 0.721499 |
| **IL-10** | | | | | | | | |
| rs10457128 | A | G | -0.0854 | 0.0172 | 6.956E-07 | 24.6459963 | 0.0121 | 0.3003 |
| rs10493718 | A | C | -0.1081 | 0.0222 | 1.068E-06 | 23.7046186 | 0.0159 | 0.601999 |
| rs13412535 | A | G | -0.1347 | 0.0224 | 1.798E-09 | 36.1515304 | 0.0146 | 0.2223 |
| rs1530455 | T | C | 0.082 | 0.0174 | 2.527E-06 | 22.2032598 | 0.0121 | 0.9955 |
| rs2086656 | T | C | -0.08 | 0.017 | 2.589E-06 | 22.1395946 | 0.0122 | 0.1222 |
| rs3002131 | C | G | 0.1191 | 0.026 | 4.592E-06 | 20.9780134 | 0.0169 | 0.6407 |
| rs3025021 | T | C | 0.0913 | 0.0194 | 2.609E-06 | 22.1424477 | 0.0124 | 0.0375198 |
| rs383684 | A | G | 0.092 | 0.0197 | 3.168E-06 | 21.8037269 | 0.0203 | 0.8174 |
| rs4741748 | A | G | -0.0788 | 0.0169 | 3.202E-06 | 21.735346 | 0.0119 | 0.8799 |
| rs6054847 | T | C | 0.0971 | 0.0207 | 2.752E-06 | 21.9981065 | 0.0147 | 0.5628 |
| rs6680918 | T | C | -0.1202 | 0.025 | 1.591E-06 | 23.1108783 | 0.0177 | 0.767701 |
| rs7088799 | T | G | -0.0815 | 0.0166 | 9.352E-07 | 24.0983093 | 0.0119 | 0.8203 |
| rs73192842 | A | G | 0.0949 | 0.0206 | 4.031E-06 | 21.2170752 | 0.0148 | 0.2829 |
| rs7747448 | A | G | -0.1061 | 0.0189 | 1.998E-08 | 31.5061032 | 0.0133 | 0.8337 |
| rs9472173 | T | C | -0.2004 | 0.0174 | 1.256E-30 | 132.612502 | 0.0119 | 0.119 |
| **IL-12p70** |  |  |  |  |  |  |  |  |
| rs113600793 | A | C | 0.1832 | 0.0359 | 3.351E-07 | 26.0349984 | 0.0249 | 0.4469 |
| rs12969892 | T | C | 0.1227 | 0.0267 | 4.194E-06 | 21.1135916 | 0.0192 | 0.3765 |
| rs2123852 | T | C | 0.0942 | 0.0204 | 0.00000373 | 21.317535 | 0.0146 | 0.0737395 |
| rs273702 | A | G | -0.127 | 0.027 | 2.522E-06 | 22.1195062 | 0.0195 | 0.6509 |
| rs282258 | T | C | 0.0726 | 0.0156 | 3.282E-06 | 21.6530739 | 0.0117 | 0.6053 |
| rs34322762 | T | C | 0.0953 | 0.0199 | 1.708E-06 | 22.9267052 | 0.0122 | 0.4795 |
| rs34826779 | T | G | -0.0884 | 0.019 | 3.327E-06 | 21.6417733 | 0.0141 | 0.1365 |
| rs41282644 | A | G | 0.1401 | 0.0303 | 3.737E-06 | 21.3740356 | 0.0215 | 0.791199 |
| rs4530855 | T | G | 0.0861 | 0.0184 | 2.872E-06 | 21.8910287 | 0.0128 | 0.3482 |
| rs4741748 | A | G | -0.0799 | 0.0163 | 9.162E-07 | 24.0222601 | 0.0119 | 0.8799 |
| rs6532374 | T | C | -0.1033 | 0.0226 | 4.613E-06 | 20.8871545 | 0.0165 | 0.3303 |
| rs7754905 | A | G | -0.1005 | 0.019 | 1.14E-07 | 27.9718014 | 0.0139 | 0.7851 |
| rs782111 | A | C | -0.0765 | 0.0156 | 9.247E-07 | 24.0419222 | 0.0116 | 0.5163 |
| rs865585 | A | C | -0.1654 | 0.0237 | 2.732E-12 | 48.69337 | 0.0164 | 0.4119 |
| rs9381249 | T | C | -0.1788 | 0.0367 | 1.125E-06 | 23.730037 | 0.0285 | 0.2437 |
| **IL-13** | | | | | | | | |
| rs10995604 | A | G | -0.1571 | 0.0343 | 4.482E-06 | 20.966335 | 0.0163 | 0.4137 |
| rs117795020 | A | G | -0.3584 | 0.0716 | 5.479E-07 | 25.0419617 | 0.0343 | 0.1917 |
| rs12623722 | A | G | -0.1189 | 0.0257 | 3.614E-06 | 21.3922198 | 0.0126 | 0.0659493 |
| rs138854806 | A | G | -0.4204 | 0.0839 | 5.449E-07 | 25.0934216 | 0.035 | 0.2848 |
| rs139083458 | T | C | 0.9995 | 0.211 | 2.165E-06 | 22.4263748 | 0.0955 | 0.498799 |
| rs147747784 | C | G | 0.369 | 0.0765 | 1.435E-06 | 23.2534958 | 0.0273 | 0.593401 |
| rs150836197 | T | C | 0.3283 | 0.0713 | 4.136E-06 | 21.1895189 | 0.0298 | 0.726501 |
| rs27949 | T | C | -0.1144 | 0.025 | 4.827E-06 | 20.9281299 | 0.0124 | 0.8796 |
| rs28442067 | A | G | -0.1379 | 0.0286 | 1.413E-06 | 23.2356516 | 0.0141 | 0.8801 |
| rs7073807 | T | C | 0.1618 | 0.0354 | 0.00000477 | 20.8789618 | 0.0171 | 0.7341 |
| rs75383097 | C | G | -0.5369 | 0.116 | 3.702E-06 | 21.4106188 | 0.047 | 0.369 |
| rs76339001 | A | T | -0.4375 | 0.0886 | 7.915E-07 | 24.3695248 | 0.0351 | 0.8478 |
| rs76975337 | T | C | -0.1211 | 0.0265 | 4.921E-06 | 20.8715608 | 0.0131 | 0.744501 |
| rs77955971 | A | C | 0.4408 | 0.0868 | 3.756E-07 | 25.775204 | 0.0322 | 0.3266 |
| **IL-16** | | | | | | | | |
| rs117217798 | T | C | -0.2064 | 0.044 | 2.772E-06 | 21.992129 | 0.0209 | 0.512201 |
| rs12577604 | T | C | 0.4335 | 0.0941 | 4.083E-06 | 21.2105631 | 0.0453 | 0.5057 |
| rs142034902 | A | G | -0.4367 | 0.0925 | 2.327E-06 | 22.2759462 | 0.0457 | 0.413 |
| rs142332135 | A | G | -0.7646 | 0.1082 | 1.581E-12 | 49.9076715 | 0.0445 | 0.724201 |
| rs144691581 | A | G | 0.4929 | 0.0958 | 2.668E-07 | 26.4569551 | 0.0397 | 0.1999 |
| rs35834666 | T | C | -0.1729 | 0.0348 | 6.57E-07 | 24.6708857 | 0.0165 | 0.3103 |
| rs4778640 | A | G | 0.7189 | 0.0983 | 2.552E-13 | 53.4543648 | 0.0458 | 0.9008 |
| rs4976691 | C | G | 0.1254 | 0.026 | 1.473E-06 | 23.2488577 | 0.0124 | 0.9319 |
| rs7097884 | T | C | -0.1193 | 0.0243 | 8.809E-07 | 24.0891559 | 0.0117 | 0.8312 |
| rs78042619 | A | G | 0.55 | 0.1158 | 2.023E-06 | 22.5455847 | 0.0506 | 0.3464 |
| **IL-17** | | | | | | | | |
| rs11985957 | A | G | 0.1511 | 0.0329 | 4.363E-06 | 21.0875351 | 0.0251 | 0.2752 |
| rs12735700 | T | G | -0.0943 | 0.0206 | 0.0000045 | 20.9496914 | 0.0142 | 0.2776 |
| rs145006174 | C | G | -0.2266 | 0.0473 | 1.645E-06 | 22.9449024 | 0.0329 | 0.2242 |
| rs17282552 | T | C | -0.2026 | 0.0403 | 4.876E-07 | 25.2672208 | 0.0266 | 0.5805 |
| rs3792369 | A | G | 0.0941 | 0.0166 | 1.46E-08 | 32.1256376 | 0.0119 | 0.729001 |
| rs61990749 | C | G | 0.1124 | 0.0226 | 6.569E-07 | 24.728879 | 0.0162 | 0.229 |
| rs78296352 | T | G | 0.2949 | 0.0645 | 4.809E-06 | 20.8986774 | 0.0499 | 0.1807 |
| rs9519328 | A | G | 0.5256 | 0.1101 | 0.00000179 | 22.7767495 | 0.0383 | 0.6353 |
| **IL-18** | | | | | | | | |
| rs10409850 | A | G | 0.1791 | 0.0347 | 2.44E-07 | 26.6253795 | 0.017 | 0.5799 |
| rs11214093 | T | C | 0.1143 | 0.0238 | 1.544E-06 | 23.0516561 | 0.0117 | 0.9921 |
| rs117266781 | T | C | 0.7051 | 0.1436 | 9.176E-07 | 24.0966203 | 0.0621 | 0.517299 |
| rs117371668 | T | G | 0.3712 | 0.0799 | 3.357E-06 | 21.571779 | 0.0396 | 0.3624 |
| rs139468359 | T | C | 0.5101 | 0.1088 | 2.737E-06 | 21.9693112 | 0.052 | 0.4818 |
| rs1979967 | T | C | 0.14 | 0.0285 | 8.719E-07 | 24.1173694 | 0.0141 | 0.3164 |
| rs4952239 | A | T | -0.1156 | 0.0242 | 1.809E-06 | 22.8059686 | 0.012 | 0.2717 |
| rs58701153 | A | T | -0.1265 | 0.0242 | 1.807E-07 | 27.3095098 | 0.0122 | 0.3801 |
| rs62312914 | T | C | -0.1265 | 0.025 | 4.215E-07 | 25.5896661 | 0.012 | 0.7289 |
| rs764078 | A | T | 0.1283 | 0.0278 | 4.075E-06 | 21.2876296 | 0.0137 | 0.8463 |
| rs77187209 | T | C | -0.4859 | 0.1041 | 3.082E-06 | 21.7748877 | 0.0495 | 0.5745 |
| rs78623212 | T | C | 0.8322 | 0.1676 | 6.82E-07 | 24.6417178 | 0.0644 | 0.719701 |
| rs78716465 | A | G | 0.3173 | 0.0679 | 2.981E-06 | 21.8254989 | 0.0311 | 0.767701 |
| **IP-10** | | | | | | | | |
| rs113183470 | A | T | -0.2414 | 0.0524 | 4.147E-06 | 21.2118552 | 0.0247 | 0.1193 |
| rs12714300 | A | T | -0.1573 | 0.0338 | 3.297E-06 | 21.6466523 | 0.0166 | 0.9993 |
| rs143799975 | A | G | -0.7551 | 0.1638 | 4.012E-06 | 21.2396738 | 0.0685 | 0.00228602 |
| rs34383175 | T | C | -0.3196 | 0.0653 | 9.904E-07 | 23.9416388 | 0.0321 | 0.7615 |
| rs397816 | T | C | 0.1211 | 0.0248 | 1.026E-06 | 23.8315133 | 0.0121 | 0.9955 |
| rs4859940 | C | G | -0.1204 | 0.0258 | 3.228E-06 | 21.7660819 | 0.0127 | 0.7011 |
| rs4862110 | T | C | -0.1453 | 0.0318 | 4.909E-06 | 20.8662153 | 0.0135 | 0.811 |
| rs75970138 | A | G | -0.4845 | 0.1037 | 2.994E-06 | 21.8170861 | 0.0517 | 0.9484 |
| rs7645625 | T | G | -0.1116 | 0.0236 | 2.192E-06 | 22.3496682 | 0.0118 | 0.7446 |
| rs78077394 | T | C | -0.3486 | 0.0701 | 6.474E-07 | 24.716412 | 0.0321 | 0.536 |
| rs79848609 | A | C | 0.2514 | 0.0535 | 2.637E-06 | 22.069357 | 0.027 | 0.7123 |
| rs8112618 | A | G | 0.1388 | 0.0297 | 3.047E-06 | 21.8289442 | 0.0145 | 0.00610998 |
| **M-CSF** | | | | | | | | |
| rs116274860 | T | G | 0.8262 | 0.1739 | 2.029E-06 | 22.553989 | 0.0522 | 0.1204 |
| rs116887628 | A | G | -0.2741 | 0.0598 | 4.626E-06 | 20.9926783 | 0.0227 | 0.6875 |
| rs117867915 | T | C | 0.5224 | 0.1096 | 1.874E-06 | 22.700602 | 0.0425 | 0.8281 |
| rs11963606 | C | G | -0.5353 | 0.117 | 4.731E-06 | 20.9158207 | 0.0454 | 0.2759 |
| rs12962919 | T | C | 0.3025 | 0.0659 | 4.394E-06 | 21.0538767 | 0.0229 | 0.676799 |
| rs139457375 | A | C | -0.4047 | 0.0854 | 2.142E-06 | 22.4389646 | 0.0334 | 0.8281 |
| rs147378920 | A | G | -0.6064 | 0.1318 | 4.177E-06 | 21.1514289 | 0.0407 | 0.9276 |
| rs34089869 | T | C | 0.2194 | 0.0462 | 2.078E-06 | 22.5341636 | 0.0187 | 0.4219 |
| rs62294910 | A | G | 0.3472 | 0.0687 | 4.378E-07 | 25.5210246 | 0.0239 | 0.8304 |
| rs72723242 | T | G | -0.4969 | 0.1083 | 4.434E-06 | 21.0345501 | 0.046 | 0.4549 |
| rs9387100 | T | C | -0.135 | 0.029 | 3.341E-06 | 21.6532798 | 0.0117 | 0.6137 |
| rs9626985 | T | C | 0.2277 | 0.0496 | 4.482E-06 | 21.0578901 | 0.0197 | 0.451 |
| **MCP-1** | | | | | | | | |
| rs111995966 | T | G | 0.1428 | 0.0309 | 3.788E-06 | 21.3518377 | 0.0218 | 0.8891 |
| rs11920996 | T | C | 0.1805 | 0.0376 | 1.604E-06 | 23.0395783 | 0.0275 | 0.9564 |
| rs12062235 | T | G | 0.1477 | 0.032 | 3.836E-06 | 21.2988834 | 0.0239 | 0.590799 |
| rs143815843 | A | G | -0.2049 | 0.0447 | 4.609E-06 | 21.0070308 | 0.0336 | 0.8248 |
| rs16837903 | A | G | -0.1104 | 0.0238 | 3.352E-06 | 21.5119627 | 0.0171 | 0.9402 |
| rs2201150 | T | C | 0.0916 | 0.016 | 1.044E-08 | 32.7677623 | 0.0119 | 0.8573 |
| rs2229593 | T | C | 0.2624 | 0.0405 | 9.246E-11 | 41.9675308 | 0.0297 | 0.0835007 |
| rs56212190 | T | C | 0.1799 | 0.0372 | 1.318E-06 | 23.3814936 | 0.0272 | 0.6308 |
| rs62245103 | T | G | 0.2433 | 0.0416 | 4.989E-09 | 34.1974213 | 0.0286 | 0.7623 |
| rs7197349 | A | G | 0.0971 | 0.0206 | 2.399E-06 | 22.2126218 | 0.0146 | 0.1605 |
| rs72705803 | A | G | -0.2188 | 0.047 | 3.222E-06 | 21.6667974 | 0.0367 | 0.6208 |
| rs7978037 | A | T | 0.0746 | 0.016 | 3.043E-06 | 21.7336912 | 0.0119 | 0.6369 |
| rs79939301 | A | G | 0.1449 | 0.0255 | 1.356E-08 | 32.281389 | 0.0183 | 0.6941 |
| rs856100 | A | G | 0.0899 | 0.0191 | 2.545E-06 | 22.1487108 | 0.0141 | 0.3966 |
| rs862990 | T | C | -0.089 | 0.0183 | 1.153E-06 | 23.6468685 | 0.0133 | 0.3307 |
| rs9317045 | A | C | 0.1157 | 0.0235 | 8.425E-07 | 24.2340944 | 0.0164 | 0.0180401 |
| **MCP-3** | | | | | | | | |
| rs10892381 | T | C | 0.2432 | 0.0473 | 2.693E-07 | 26.3884447 | 0.0123 | 0.7813 |
| rs117286643 | A | G | 0.6934 | 0.1474 | 2.542E-06 | 22.0892842 | 0.0402 | 0.8652 |
| rs28394764 | A | T | 0.597 | 0.1282 | 3.194E-06 | 21.646194 | 0.0334 | 0.595899 |
| rs3129806 | T | C | -0.1975 | 0.0433 | 4.978E-06 | 20.766693 | 0.0117 | 0.3706 |
| rs6993671 | T | C | 0.2041 | 0.0443 | 4.061E-06 | 21.1878794 | 0.0119 | 0.4543 |
| rs7275485 | T | C | -0.2218 | 0.0481 | 3.986E-06 | 21.2247149 | 0.013 | 0.1878 |
| **MIF** | | | | | | | | |
| rs1007888 | T | C | -0.1275 | 0.0245 | 1.915E-07 | 27.0671345 | 0.0119 | 0.1345 |
| rs113218956 | A | G | -0.8789 | 0.1876 | 2.815E-06 | 21.9365002 | 0.0899 | 0.4397 |
| rs11551183 | C | G | 0.3666 | 0.0795 | 3.999E-06 | 21.2522416 | 0.0395 | 0.3919 |
| rs12594190 | A | G | 0.1321 | 0.0266 | 6.85E-07 | 24.6488375 | 0.0127 | 0.3634 |
| rs141009259 | T | C | -0.6194 | 0.1285 | 1.444E-06 | 23.2214977 | 0.0552 | 0.00219599 |
| rs2294689 | C | G | -0.1338 | 0.0287 | 3.043E-06 | 21.7221353 | 0.0476 | 0.5551 |
| rs35792361 | A | G | -0.2586 | 0.0527 | 9.003E-07 | 24.065223 | 0.0249 | 0.564 |
| rs35890933 | T | G | 0.1676 | 0.0365 | 4.458E-06 | 21.0725154 | 0.0165 | 0.560101 |
| rs3814097 | A | G | -0.1163 | 0.0251 | 3.548E-06 | 21.456855 | 0.0118 | 0.5726 |
| rs78098071 | T | C | -0.4583 | 0.0915 | 5.509E-07 | 25.0733063 | 0.0455 | 0.0947393 |
| **MIG** |  |  |  |  |  |  |  |  |
| rs10266753 | T | C | -0.2016 | 0.0397 | 3.769E-07 | 25.7731047 | 0.0208 | 0.2846 |
| rs111607343 | A | G | -0.5235 | 0.1119 | 2.928E-06 | 21.8745887 | 0.0492 | 0.4772 |
| rs11177248 | A | G | 0.3157 | 0.0667 | 2.222E-06 | 22.3905207 | 0.0296 | 0.4439 |
| rs113302091 | T | C | 0.2537 | 0.0553 | 4.402E-06 | 21.0357194 | 0.028 | 0.0943409 |
| rs13143163 | C | G | 0.2735 | 0.0582 | 2.622E-06 | 22.0716799 | 0.0251 | 0.8001 |
| rs139010077 | T | C | 0.4337 | 0.0943 | 4.193E-06 | 21.1408359 | 0.0456 | 0.763001 |
| rs191555775 | A | T | 0.2279 | 0.0412 | 3.278E-08 | 30.5816501 | 0.0192 | 0.0676893 |
| rs192433162 | A | G | -0.8045 | 0.1676 | 1.594E-06 | 23.0287712 | 0.077 | 0.6441 |
| rs3733233 | T | C | 0.1223 | 0.025 | 1.049E-06 | 23.9188113 | 0.0122 | 0.5225 |
| rs62562991 | A | G | 0.6239 | 0.1259 | 7.237E-07 | 24.5439924 | 0.0483 | 0.505 |
| rs6679677 | A | C | 0.1628 | 0.0327 | 6.514E-07 | 24.7730793 | 0.0164 | 0.3038 |
| rs8127917 | T | G | 0.2382 | 0.0492 | 1.278E-06 | 23.4271795 | 0.0244 | 0.3713 |
| rs816960 | T | C | -0.1179 | 0.0242 | 0.00000115 | 23.7226704 | 0.012 | 0.6185 |
| **MIP-1A** | | | | | | | | |
| rs117506943 | T | C | 0.0108 | 0.0682 | 4.484E-06 | 21.0242617 | 0.0293 | 0.426 |
| rs12159394 | A | G | 0.0008 | 0.0366 | 3.112E-06 | 21.7655465 | 0.0182 | 0.9865 |
| rs57786342 | A | G | 0.0149 | 0.0283 | 8.909E-07 | 24.1108624 | 0.0153 | 0.8111 |
| rs6956239 | T | C | -0.0088 | 0.026 | 4.583E-06 | 20.9364595 | 0.0127 | 0.781801 |
| **MIP-1B** | | | | | | | | |
| rs111721971 | T | G | -0.227 | 0.047 | 1.387E-06 | 23.321215 | 0.0317 | 0.1665 |
| rs116237296 | A | G | 0.5284 | 0.1115 | 2.153E-06 | 22.4528284 | 0.0821 | 0.0175999 |
| rs11651720 | T | C | -0.1189 | 0.0219 | 5.688E-08 | 29.4693566 | 0.0161 | 0.1821 |
| rs11716293 | C | G | 0.0986 | 0.0189 | 1.689E-07 | 27.2098029 | 0.0135 | 0.4259 |
| rs117657747 | A | G | 0.2089 | 0.0453 | 4.013E-06 | 21.2589533 | 0.0254 | 0.8982 |
| rs145526037 | T | G | -0.1863 | 0.0406 | 4.473E-06 | 21.0508102 | 0.0301 | 0.2237 |
| rs17138331 | A | G | -0.1434 | 0.0295 | 1.125E-06 | 23.623783 | 0.0209 | 0.0662903 |
| rs17661219 | C | G | 0.0872 | 0.0172 | 3.888E-07 | 25.6963388 | 0.0123 | 0.8256 |
| rs2314809 | T | C | -0.0735 | 0.0157 | 2.904E-06 | 21.9114212 | 0.0116 | 0.947 |
| rs2742396 | T | C | 0.1071 | 0.0168 | 1.832E-10 | 40.6308167 | 0.0119 | 0.1636 |
| rs281728 | A | C | -0.079 | 0.0171 | 3.891E-06 | 21.3381683 | 0.0126 | 0.7093 |
| rs28393318 | A | G | -0.1076 | 0.0235 | 4.616E-06 | 20.9596484 | 0.0174 | 0.1059 |
| rs57893487 | C | G | -0.1075 | 0.0214 | 4.916E-07 | 25.2281007 | 0.0143 | 0.7902 |
| rs6802288 | A | G | -0.1623 | 0.0173 | 6.574E-21 | 87.9913554 | 0.013 | 0.9095 |
| rs6806860 | A | C | -0.1008 | 0.0188 | 8.575E-08 | 28.7409116 | 0.0139 | 0.671699 |
| rs6908843 | A | G | 0.0997 | 0.0209 | 1.779E-06 | 22.7506033 | 0.0155 | 0.7019 |
| rs72791296 | T | C | 0.2364 | 0.0466 | 3.968E-07 | 25.7287262 | 0.0321 | 0.8794 |
| rs72799710 | T | C | -0.1037 | 0.0217 | 1.792E-06 | 22.8314355 | 0.016 | 0.2211 |
| rs74979864 | A | T | -0.3184 | 0.0613 | 2.025E-07 | 26.9724467 | 0.0447 | 0.8377 |
| rs76582507 | A | G | 0.3259 | 0.0676 | 1.421E-06 | 23.2364888 | 0.066 | 0.1815 |
| rs772112 | A | T | -0.142 | 0.0216 | 5.007E-11 | 43.2080195 | 0.0149 | 0.1851 |
| rs9916627 | T | C | -0.096 | 0.0198 | 1.244E-06 | 23.5021319 | 0.0148 | 0.8671 |
| **PDGF-BB** | | | | | | | | |
| rs10512952 | T | C | -0.2816 | 0.0587 | 1.636E-06 | 23.0083196 | 0.0427 | 0.5939 |
| rs116154010 | T | C | 0.3225 | 0.0662 | 1.113E-06 | 23.726805 | 0.0507 | 0.5449 |
| rs11766649 | A | G | 0.0902 | 0.0196 | 3.964E-06 | 21.173699 | 0.0143 | 0.1311 |
| rs12289510 | A | G | -0.0772 | 0.0158 | 1.001E-06 | 23.868011 | 0.0116 | 0.2765 |
| rs12615784 | T | C | -0.1003 | 0.0193 | 1.986E-07 | 27.0011991 | 0.0142 | 0.5981 |
| rs147862316 | T | C | 0.2279 | 0.0411 | 2.987E-08 | 30.7397838 | 0.032 | 0.535499 |
| rs2643354 | A | G | 0.1251 | 0.0261 | 1.633E-06 | 22.9683294 | 0.0196 | 0.965 |
| rs35859699 | A | G | -0.3854 | 0.0838 | 4.223E-06 | 21.146144 | 0.054 | 0.5555 |
| rs62191444 | T | G | -0.112 | 0.0239 | 2.678E-06 | 21.9551317 | 0.0162 | 0.8871 |
| rs6756793 | T | C | 0.0876 | 0.0157 | 2.684E-08 | 31.1246668 | 0.0117 | 0.7543 |
| rs6910518 | T | G | 0.0806 | 0.0162 | 6.005E-07 | 24.7477578 | 0.0119 | 0.2534 |
| rs72972467 | C | G | -0.1616 | 0.0328 | 8.286E-07 | 24.2678235 | 0.0229 | 0.0104701 |
| rs73162807 | A | C | -0.2313 | 0.0499 | 3.548E-06 | 21.4805787 | 0.0373 | 0.2653 |
| rs9924851 | C | G | 0.0767 | 0.0163 | 2.702E-06 | 22.1366206 | 0.0121 | 0.4957 |
| **RANTES** | | | | | | | | |
| rs10505135 | T | C | 0.035 | 0.0252 | 1.899E-07 | 27.2144071 | 0.0121 | 0.6544 |
| rs118096511 | T | C | -0.0676 | 0.0709 | 1.965E-06 | 22.6332093 | 0.0345 | 0.612999 |
| rs11873385 | A | G | 0.0096 | 0.0552 | 2.927E-06 | 21.8666762 | 0.0256 | 0.6965 |
| rs148526102 | T | C | -0.002 | 0.083 | 4.793E-06 | 20.9267695 | 0.04 | 0.5928 |
| rs2731672 | T | C | -0.0476 | 0.0272 | 4.827E-06 | 20.8378946 | 0.0133 | 0.697 |
| rs4795087 | C | G | -0.0065 | 0.0312 | 1.629E-06 | 22.9160869 | 0.015 | 0.1505 |
| rs62438851 | A | G | -0.024 | 0.0413 | 4.009E-06 | 21.241356 | 0.0191 | 0.804 |
| rs7170339 | C | G | 0.0645 | 0.0904 | 2.187E-06 | 22.434053 | 0.0414 | 0.3024 |
| rs72793342 | A | G | -0.0251 | 0.0307 | 9.08E-07 | 24.0184348 | 0.0143 | 0.6468 |
| rs78050316 | A | C | -0.0279 | 0.0859 | 9.946E-07 | 23.9152113 | 0.0437 | 0.9083 |
| **SCF** | | | | | | | | |
| rs10800449 | A | C | 0.0851 | 0.0179 | 1.962E-06 | 22.5968979 | 0.0128 | 0.490999 |
| rs11244035 | T | C | -0.1296 | 0.0279 | 3.501E-06 | 21.5723452 | 0.0208 | 0.3662 |
| rs113127926 | A | C | 0.1974 | 0.0418 | 2.337E-06 | 22.2965412 | 0.0298 | 0.4097 |
| rs117721699 | C | G | -0.2392 | 0.0484 | 7.514E-07 | 24.4189729 | 0.036 | 0.286 |
| rs12345108 | T | C | -0.0772 | 0.0167 | 3.731E-06 | 21.3647307 | 0.0122 | 0.8245 |
| rs13412535 | A | G | -0.1065 | 0.0213 | 5.586E-07 | 24.9940005 | 0.0146 | 0.2223 |
| rs138538809 | T | C | -0.5788 | 0.1139 | 3.757E-07 | 25.8169755 | 0.0788 | 0.219 |
| rs72678285 | A | T | 0.1062 | 0.0231 | 0.00000443 | 21.1310384 | 0.0158 | 0.9652 |
| rs78666213 | T | G | -0.2845 | 0.0574 | 7.152E-07 | 24.5604614 | 0.0399 | 0.8803 |
| rs8045376 | A | G | -0.3126 | 0.068 | 4.268E-06 | 21.1278783 | 0.0513 | 0.0400701 |
| **SCGF-B** | | | | | | | | |
| rs11111869 | A | G | 0.1621 | 0.0311 | 1.861E-07 | 27.1526211 | 0.0156 | 0.9249 |
| rs112346514 | T | C | -0.3261 | 0.0703 | 3.543E-06 | 21.5058918 | 0.0301 | 0.4265 |
| rs1149926 | T | C | -0.3458 | 0.0749 | 3.917E-06 | 21.3035927 | 0.0373 | 0.7921 |
| rs118003677 | T | C | -0.3654 | 0.0786 | 3.348E-06 | 21.6002363 | 0.0392 | 0.4992 |
| rs12118918 | A | G | -0.1631 | 0.035 | 3.208E-06 | 21.7039281 | 0.0165 | 0.625299 |
| rs12480722 | T | C | 0.1654 | 0.0353 | 2.812E-06 | 21.9426011 | 0.0172 | 0.5754 |
| rs13287050 | A | T | -0.121 | 0.0263 | 4.118E-06 | 21.1556197 | 0.0129 | 0.4568 |
| rs13866 | T | C | -0.1647 | 0.028 | 3.773E-09 | 34.5810077 | 0.0133 | 0.0810103 |
| rs139413256 | A | G | -0.5174 | 0.1076 | 1.532E-06 | 23.109727 | 0.0454 | 0.1959 |
| rs143829871 | T | C | -0.1866 | 0.0399 | 2.852E-06 | 21.8596898 | 0.0199 | 0.9723 |
| rs144724875 | T | C | 0.5381 | 0.0829 | 8.645E-11 | 42.1098245 | 0.035 | 0.2464 |
| rs149009264 | A | G | 0.4551 | 0.0985 | 3.793E-06 | 21.3357404 | 0.0473 | 0.9794 |
| rs150733161 | T | C | -0.5255 | 0.112 | 2.687E-06 | 22.0026963 | 0.0508 | 0.3959 |
| rs151194174 | A | G | 0.4536 | 0.0941 | 1.454E-06 | 23.2238038 | 0.0305 | 0.7833 |
| rs264157 | A | G | 0.1079 | 0.0233 | 3.685E-06 | 21.4337201 | 0.0116 | 0.919 |
| rs34911860 | A | G | -0.3674 | 0.0787 | 3.002E-06 | 21.7818793 | 0.045 | 0.5825 |
| rs3817303 | T | G | 0.1362 | 0.0294 | 3.602E-06 | 21.4499391 | 0.0147 | 0.0440403 |
| rs4737731 | T | C | 0.1146 | 0.0251 | 4.871E-06 | 20.8347503 | 0.0128 | 0.6167 |
| rs4976691 | C | G | -0.1484 | 0.0253 | 4.438E-09 | 34.3869192 | 0.0124 | 0.9319 |
| rs77954165 | T | C | 0.2631 | 0.0562 | 2.867E-06 | 21.9046124 | 0.0266 | 0.9622 |
| rs78217154 | T | C | 0.3942 | 0.0861 | 4.722E-06 | 20.9504543 | 0.0409 | 0.764499 |
| **SDF-1A** | | | | | | | | |
| rs10474392 | A | G | 0.0934 | 0.0177 | 1.376E-07 | 27.8378865 | 0.0133 | 0.3614 |
| rs10516368 | A | C | -0.4268 | 0.0883 | 1.356E-06 | 23.3569674 | 0.0637 | 0.5543 |
| rs12141941 | T | C | -0.0881 | 0.0186 | 2.263E-06 | 22.4292612 | 0.0129 | 0.2758 |
| rs149893336 | A | G | -0.494 | 0.1082 | 0.00000493 | 20.8395539 | 0.0544 | 0.5919 |
| rs1600396 | A | G | -0.0933 | 0.0204 | 4.939E-06 | 20.9118278 | 0.0146 | 0.4445 |
| rs62194946 | T | G | -0.0849 | 0.0185 | 4.552E-06 | 21.0552772 | 0.0133 | 0.3133 |
| rs6586903 | T | C | -0.1264 | 0.0268 | 2.421E-06 | 22.2389153 | 0.0185 | 0.5988 |
| rs76766406 | A | G | 0.4642 | 0.1012 | 4.489E-06 | 21.0347952 | 0.0681 | 0.6006 |
| rs78883416 | C | G | -0.0871 | 0.0182 | 1.755E-06 | 22.8972104 | 0.0125 | 0.2881 |
| **TNF-A** | | | | | | | | |
| rs10767536 | A | G | 0.118 | 0.0253 | 3.146E-06 | 21.7407357 | 0.0122 | 0.1428 |
| rs115018697 | C | G | -0.9542 | 0.197 | 1.273E-06 | 23.4475589 | 0.0889 | 0.6899 |
| rs116736594 | T | C | 0.3407 | 0.0702 | 1.223E-06 | 23.5408048 | 0.0313 | 0.9272 |
| rs79105320 | A | G | 0.5573 | 0.1177 | 2.207E-06 | 22.4066239 | 0.0556 | 0.5163 |
| **TNF-B** | | | | | | | | |
| rs10925040 | T | C | 0.1738 | 0.0372 | 2.929E-06 | 21.8001085 | 0.0121 | 0.6611 |
| rs2420873 | T | G | 0.1673 | 0.0365 | 4.513E-06 | 20.9822059 | 0.0119 | 0.0360504 |
| rs62284710 | A | G | 0.3702 | 0.0782 | 2.183E-06 | 22.3822794 | 0.0244 | 0.7359 |
| rs75240021 | C | G | 0.3713 | 0.0772 | 1.489E-06 | 23.1025697 | 0.0223 | 0.7019 |
| rs76225863 | A | G | 0.7534 | 0.1217 | 5.982E-10 | 38.2749883 | 0.0368 | 0.8714 |
| **TRAIL** | | | | | | | | |
| rs113057689 | A | G | -0.2625 | 0.0489 | 7.972E-08 | 28.8094751 | 0.0279 | 0.7453 |
| rs11875481 | T | C | -0.0969 | 0.0211 | 4.601E-06 | 21.0851692 | 0.0155 | 0.9792 |
| rs12458564 | A | T | -0.1002 | 0.0175 | 1.1E-08 | 32.7758372 | 0.0128 | 0.8335 |
| rs13115587 | A | C | 0.101 | 0.0217 | 3.244E-06 | 21.657969 | 0.0166 | 0.958 |
| rs13278062 | T | G | 0.08 | 0.0157 | 3.326E-07 | 25.9582324 | 0.0116 | 0.6485 |
| rs139958028 | A | G | 0.1803 | 0.0395 | 4.992E-06 | 20.830117 | 0.0277 | 0.4605 |
| rs183815186 | A | T | -0.3499 | 0.0602 | 6.341E-09 | 33.7745328 | 0.043 | 0.3416 |
| rs550057 | T | C | -0.0783 | 0.0169 | 3.707E-06 | 21.4607336 | 0.0126 | 0.2661 |
| rs558572 | T | C | 0.1351 | 0.0265 | 3.419E-07 | 25.9844422 | 0.0194 | 0.3777 |
| rs57396456 | T | C | -0.5641 | 0.0516 | 7.71E-28 | 119.48331 | 0.0386 | 0.0308802 |
| rs616114 | T | C | -0.1033 | 0.0162 | 1.715E-10 | 40.6504223 | 0.0119 | 0.674401 |
| rs62093482 | T | C | 0.9827 | 0.0529 | 6.123E-77 | 345.004703 | 0.0401 | 0.0793707 |
| rs747324 | T | C | -0.0826 | 0.0178 | 3.338E-06 | 21.528538 | 0.0129 | 0.1723 |
| rs75928541 | A | G | 0.2784 | 0.0591 | 2.442E-06 | 22.1849241 | 0.0392 | 0.5034 |
| rs7599203 | T | C | 0.0918 | 0.02 | 4.333E-06 | 21.0629802 | 0.0148 | 0.3248 |
| rs78682108 | A | G | -0.2383 | 0.0394 | 1.435E-09 | 36.572116 | 0.0265 | 0.9702 |
| **VEGF** | | | | | | | | |
| rs10411345 | C | G | -0.1041 | 0.0218 | 1.733E-06 | 22.7964477 | 0.0139 | 0.2189 |
| rs10757514 | C | G | -0.1024 | 0.0222 | 4.169E-06 | 21.2702537 | 0.0152 | 0.3057 |
| rs10822118 | T | C | -0.0797 | 0.0168 | 2.211E-06 | 22.499703 | 0.0116 | 0.1611 |
| rs10934631 | T | C | -0.1132 | 0.0244 | 3.607E-06 | 21.5175047 | 0.0164 | 0.7264 |
| rs114773511 | T | C | 0.2187 | 0.0441 | 6.971E-07 | 24.5866349 | 0.0287 | 0.8535 |
| rs12456390 | T | C | -0.0818 | 0.0179 | 4.882E-06 | 20.8775365 | 0.0125 | 0.763999 |
| rs1730969 | C | G | -0.7811 | 0.1696 | 4.106E-06 | 21.2027728 | 0.136 | 0.06498 |
| rs181031888 | A | T | 0.3767 | 0.0498 | 4.014E-14 | 57.2020062 | 0.032 | 0.4326 |
| rs2039420 | C | G | 0.0975 | 0.0175 | 2.744E-08 | 31.0321481 | 0.012 | 0.769699 |
| rs56071907 | T | C | 0.126 | 0.027 | 0.00000301 | 21.7716963 | 0.0167 | 0.9777 |
| rs60013354 | A | G | -0.2497 | 0.0521 | 1.662E-06 | 22.9636196 | 0.0345 | 0.0303697 |
| rs62401205 | A | C | -0.2015 | 0.0412 | 9.946E-07 | 23.9130171 | 0.0236 | 0.0508803 |
| rs6496613 | A | C | -0.2359 | 0.0515 | 4.701E-06 | 20.9758771 | 0.0356 | 0.557799 |
| rs7356919 | A | G | -0.1694 | 0.02 | 2.846E-17 | 71.7208662 | 0.0135 | 0.706999 |
| rs73872715 | T | C | -0.6079 | 0.1299 | 2.864E-06 | 21.8939787 | 0.0769 | 0.0222998 |
| rs76458389 | T | G | -0.1786 | 0.0363 | 8.573E-07 | 24.2007243 | 0.0249 | 0.5147 |
| rs77961527 | A | G | 0.2289 | 0.0457 | 5.525E-07 | 25.0805982 | 0.0272 | 0.8709 |
| rs9381249 | T | C | -0.2414 | 0.0396 | 1.038E-09 | 37.1503462 | 0.0285 | 0.2437 |

S17. Chemical structure and function of the selected natural active ingredients

| Component | Chemical structure characteristics | Known for the function of the eye |
| --- | --- | --- |
| anthocyanin | Polyphenolic compounds containing the core structure of Benzopyrylium ring | Inhibit VEGF signaling pathway, reduce oxidative stress and retinal vascular leakage [9, 10] |
| procyanidin | Flavan-3-ol polymer containing catechins and epicatechin units | Anti-inflammatory, inhibit NF-κB pathway, protect the integrity of the blood-retinal barrier [11] |
| lutein | Carotenoids, containing conjugated polyene chains and two epoxide groups | Antioxidant, filter blue light, protect photoreceptor cells from light damage [12] |
| quercetin | Flavonols, 3-OH substituents, C2-C3 double bonds and C4 carbonyl groups | Inhibit the release of inflammatory factors (such as TNF-α, IL-6) and reduce the activation of retinal glial cells [13, 14] |
| resveratrol | Polyphenols, containing Trans-stilbene | Activating SIRT1 pathway, inhibiting NLRP3 inflammasome, alleviating oxidative stress-induced lens epithelial cell damage [15, 16] |

S18. Baseline Characteristics Table of the study population

| Variable | AMD（n=3763） | Glaucoma（n=8591） | DR（n=3646） | Myopia（n=1640） | Cataract（n=26758） | Inflammatory cytokines  （n=8239） | |
| --- | --- | --- | --- | --- | --- | --- | --- |
| Age (mean ±SD) | 68.2 ± 8.5 | 65.4 ± 9.3 | 62.1 ± 10.7 | 28.4 ± 12.6 | 70.8 ± 7.9 | 60.1 ±5.3 | 37.0 ± 6.5 |
| Percentage of women（%） | 54% | 58% | 42% | 51% | 52% | 60% | 62% |
| CCI | N.A. | N.A. | N.A. | N.A. | N.A. | N.A. | N.A. |
| Sample collection time point | N.A. | N.A. | N.A. | N.A. | N.A. | N.A. | N.A. |

Figure S1. SNP screening process

Figure S1. SNP screening process

Figure S1. SNP screening process

Figure S1. SNP screening process

Figure S1. SNP screening process

Figure S1. SNP screening process

Figure S1. SNP screening process


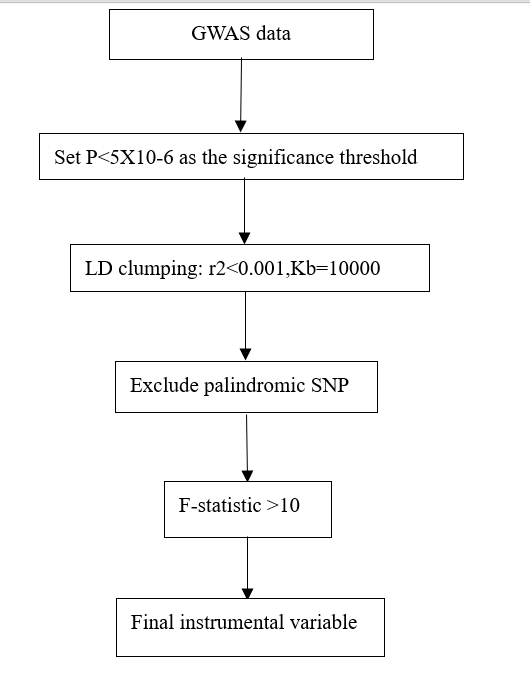


1. Harju N. Regulation of oxidative stress and inflammatory responses in human retinal pigment epithelial cells. Acta Ophthalmol. 2022; 100 Suppl 273:3-59;doi:10.1111/aos.15275.

2. Korhonen E, Piippo N, Hytti M, Kaarniranta K, Kauppinen A. Cis-urocanic acid improves cell viability and suppresses inflammasome activation in human retinal pigment epithelial cells. Biochem Pharmacol. 2023; 216:115790;doi:10.1016/j.bcp.2023.115790.

3. Oribio-Quinto C, Burgos-Blasco B, Pérez-García P, Espino-Paisán L, Sarriá B, Fernández-Vigo JI, García-Feijóo J. Aqueous Humor Cytokine Profile in Primary Congenital Glaucoma. J Clin Med. 2023; 12(9);doi:10.3390/jcm12093142.

4. Lee SJ, Noh SE, Jo DH, Cho CS, Park KS, Kim JH. IL-10-induced modulation of macrophage polarization suppresses outer-blood-retinal barrier disruption in the streptozotocin-induced early diabetic retinopathy mouse model. Faseb j. 2024; 38(9):e23638;doi:10.1096/fj.202400053R.

5. Obasanmi G, Lois N, Armstrong D, Hombrebueno JMR, Lynch A, Chen M, Xu H. Peripheral Blood Mononuclear Cells from Patients with Type 1 Diabetes and Diabetic Retinopathy Produce Higher Levels of IL-17A, IL-10 and IL-6 and Lower Levels of IFN-γ-A Pilot Study. Cells. 2023; 12(3);doi:10.3390/cells12030467.

6. Zhang JS, Wang JD, Zhu GY, Yusufu M, Xiong Y, Li J, Cao K, Jin SS, He HL, Sun XL *et al*. The high expression of the pain-related inflammatory factors in the eyes of cataract patients infected with hepatitis B virus. Cytokine. 2020; 134:155189;doi:10.1016/j.cyto.2020.155189.

7. Hu S, Lin Z, Hu MJ, Tan JS, Guo TT, Huang X, Hua L. Causal relationships of circulating amino acids with cardiovascular disease: a trans-ancestry Mendelian randomization analysis. J Transl Med. 2023; 21(1):699;doi:10.1186/s12967-023-04580-y.

8. Yuan S, Kim JH, Xu P, Wang Z. Causal association between celiac disease and inflammatory bowel disease: A two-sample bidirectional Mendelian randomization study. Front Immunol. 2022; 13:1057253;doi:10.3389/fimmu.2022.1057253.

9. Li R, Ye Z, Yang W, Xu YJ, Tan CP, Liu Y. Blueberry Anthocyanins from Commercial Products: Structure Identification and Potential for Diabetic Retinopathy Amelioration. Molecules. 2022; 27(21);doi:10.3390/molecules27217475.

10. Yacout SM, Gaillard ER. The Anthocyanins, Oenin and Callistephin, Protect RPE Cells Against Oxidative Stress. Photochem Photobiol. 2017; 93(2):590-599;doi:10.1111/php.12683.

11. Liu Y, Liu X, Chen X, Yang Z, Chen J, Zhu W, Li Y, Wen Y, Deng C, Gu C *et al*. Senolytic and senomorphic agent procyanidin C1 alleviates structural and functional decline in the aged retina. Proc Natl Acad Sci U S A. 2024; 121(18):e2311028121;doi:10.1073/pnas.2311028121.

12. Li LH, Lee JC, Leung HH, Lam WC, Fu Z, Lo ACY. Lutein Supplementation for Eye Diseases. Nutrients. 2020; 12(6);doi:10.3390/nu12061721.

13. Zou Y, Jiang J, Li Y, Ding X, Fang F, Chen L. Quercetin Regulates Microglia M1/M2 Polarization and Alleviates Retinal Inflammation via ERK/STAT3 Pathway. Inflammation. 2024; 47(5):1616-1633;doi:10.1007/s10753-024-01997-5.

14. Kumar B, Gupta SK, Nag TC, Srivastava S, Saxena R, Jha KA, Srinivasan BP. Retinal neuroprotective effects of quercetin in streptozotocin-induced diabetic rats. Exp Eye Res. 2014; 125:193-202;doi:10.1016/j.exer.2014.06.009.

15. Nguyen DD, Luo LJ, Yang CJ, Lai JY. Highly Retina-Permeating and Long-Acting Resveratrol/Metformin Nanotherapeutics for Enhanced Treatment of Macular Degeneration. ACS Nano. 2023; 17(1):168-183;doi:10.1021/acsnano.2c05824.

16. Tufekci KU, Eltutan BI, Isci KB, Genc S. Resveratrol Inhibits NLRP3 Inflammasome-Induced Pyroptosis and miR-155 Expression in Microglia Through Sirt1/AMPK Pathway. Neurotox Res. 2021; 39(6):1812-1829;doi:10.1007/s12640-021-00435-w.
